# Breeding for disease resistance in soybean: a global perspective

**DOI:** 10.1007/s00122-022-04101-3

**Published:** 2022-07-05

**Authors:** Feng Lin, Sushil Satish Chhapekar, Caio Canella Vieira, Marcos Paulo Da Silva, Alejandro Rojas, Dongho Lee, Nianxi Liu, Esteban Mariano Pardo, Yi-Chen Lee, Zhimin Dong, Jose Baldin Pinheiro, Leonardo Daniel Ploper, John Rupe, Pengyin Chen, Dechun Wang, Henry T. Nguyen

**Affiliations:** 1 Department of Plant, Soil and Microbial Sciences, Michigan State University, East Lansing, MI 48824 USA; 2 Division of Plant Sciences and National Center for Soybean Biotechnology, University of Missouri-Columbia, Columbia, MO 65211 USA; 3 Fisher Delta Research Center, University of Missouri, Portageville, MO 63873 USA; 4 Department of Entomology and Plant Pathology, University of Arkansas, Fayetteville, AR 72701 USA; 5 Soybean Research Institute, Jilin Academy of Agricultural Sciences, Changchun,, 130033 Jilin China; 6 Instituto de Tecnología Agroindustrial del Noroeste Argentino (ITANOA) [Estación Experimental Agroindustrial Obispo Colombres (EEAOC) – Consejo Nacional de Investigaciones Científicas y Técnicas (CONICET)], Av. William Cross 3150, C.P. T4101XAC, Las Talitas, Tucumán, Argentina; 7 Departamento de Genética, Escola Superior de Agricultura “Luiz de Queiroz” (ESALQ/USP), PO Box 9, Piracicaba, SP 13418-900 Brazil

## Abstract

**Key message:**

**This review provides a comprehensive atlas of QTLs, genes, and alleles conferring resistance to 28 important diseases in all major soybean production regions in the world.**

**Abstract:**

Breeding disease-resistant soybean [*Glycine max* (L.) Merr.] varieties is a common goal for soybean breeding programs to ensure the sustainability and growth of soybean production worldwide. However, due to global climate change, soybean breeders are facing strong challenges to defeat diseases. Marker-assisted selection and genomic selection have been demonstrated to be successful methods in quickly integrating vertical resistance or horizontal resistance into improved soybean varieties, where vertical resistance refers to R genes and major effect QTLs, and horizontal resistance is a combination of major and minor effect genes or QTLs. This review summarized more than 800 resistant loci/alleles and their tightly linked markers for 28 soybean diseases worldwide, caused by nematodes, oomycetes, fungi, bacteria, and viruses. The major breakthroughs in the discovery of disease resistance gene atlas of soybean were also emphasized which include: (1) identification and characterization of vertical resistance genes reside *rhg1* and *Rhg4* for soybean cyst nematode, and exploration of the underlying regulation mechanisms through copy number variation and (2) map-based cloning and characterization of *Rps11* conferring resistance to 80% isolates of *Phytophthora sojae* across the USA. In this review, we also highlight the validated QTLs in overlapping genomic regions from at least two studies and applied a consistent naming nomenclature for these QTLs. Our review provides a comprehensive summary of important resistant genes/QTLs and can be used as a toolbox for soybean improvement. Finally, the summarized genetic knowledge sheds light on future directions of accelerated soybean breeding and translational genomics studies.

**Supplementary Information:**

The online version contains supplementary material available at 10.1007/s00122-022-04101-3.

## Introduction

**Fig. 1:**
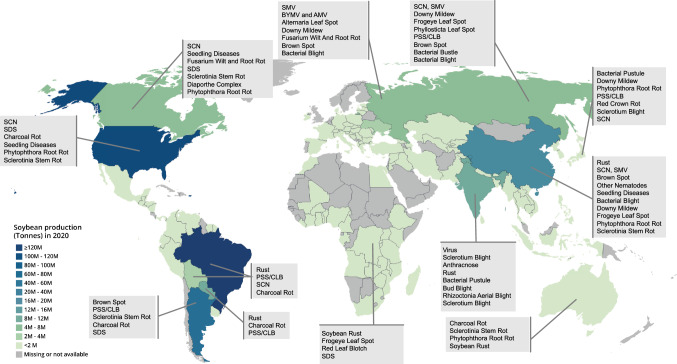
Global soybean yield production in 2020 (data obtained from FAOSTAT) and major diseases in top ten soybean production countries. SCN: soybean cyst nematode; SDS: sudden death syndrome; PSS: Phomopsis seed decay; CLB: Cercospora leaf blight; SMV: soybean mosaic virus; BYMV: bean yellow mosaic virus; AMV: Alfalfa mosaic virus

Soybean [*Glycine max* (L.) Merr.] is one of the most important crops globally. It produced 70.86% of the global supply of plant-based protein meal and 28.88% of the plant-based oil (second only to palm oil) in the 2020/2021 market year (Market View Data Base, Untied Soybean Board 2021. https://marketviewdb.centrec.com/?bi=Global_MealandOil_Consumption_Annual). Total world soybean production in 2020 was 353.5 million metric tons (Mt), and the estimated cultivated area was 127.0 million ha. While cultivated throughout the world, 96.2% of soybean production is concentrated in ten countries: Brazil (121.8 million Mt), the USA (112.5 million Mt), Argentina (48.8 million Mt), China (19.6 million Mt), India (11.2 million Mt), Paraguay (11.0 million Mt), Canada (6.4 million Mt), Russia (4.3 million Mt), Ukraine (2.8 million Mt), and Bolivia (2.8 million Mt) (FAOSTAT 2020; Fig. 1). A major constraint to soybean production is disease loss. Of more than 200 pathogens known to infect soybean, only about 35 are economically important (Hartman et al. 2016). The most prevalent diseases in major soybean production regions of the world are presented in 1. The type and severity of disease and the degree of yield and seed quality loss vary with region and year, depending on the climate and the growing season weather, cultural and disease control practices, and the genetic diversity of the pathogens and the soybean cultivars. Unfortunately, the proportion of global soybean yield loss due to diseases increased from ~ 11% in 1994 to 27% in 2006. In 1994, soybean diseases caused losses of nearly 15 million Mt (10.87% of total production), valued at more than $3 billion across the top ten soybean production countries (Wrather et al. 1997). In 1998, the world soybean yield losses due to diseases were more than 28 million Mt (18.49% of total production), more than doubled the losses in 1994 (> $6 billion) (Wrather et al. 2001). In 2006, a total of 59.9 million Mt of soybean production were reduced in the world, accounting for more than 27% of the total soybean production (220.4 million Mt) (Wrather et al. 2010).

**Table 1:** Soybean diseases in major soybean production regions of the world

	Common disease name	Causal agent
Nematode diseases	Lance nematodes^a^	*Hoplolaimus* spp.
	Lesion nematodes	*Pratylenchus* spp*.*
	Reniform nematode^a^	*Rotylenchulus reniformis*
	Root-knot nematodes^a^	*Meloidogyne* spp.
	Soybean cyst nematode^a^	*Heterodera glycines*
Oomycete diseases	Downy mildew^a^	*Peronospora manshurica*
	Phytophthora root and stem rot^a^	*Phytophthora sojae, P. sanseomeana*
	Pythium damping off and root rot^a^	*Pythium* spp.
Fungal diseases	Alternaria leaf spot	*Alternaria* spp.
	Anthracnose	*Colletotrichum* spp.
	Brown spot	*Septoria glycines*
	Brown stem rot^a^	*Cadophora gregata*
	Cercospora leaf blight and purple seed stain^a^	*Cercospora kikuchii*
	Charcoal rot^a^	*Macrophomina phaseolina*
	Frogeye leaf spot^a^	*Cercospora sojina*
	Fusarium wilt and root rot^a^	*Fusarium* spp*.*
	Phomopsis seed decay^a^	*Phomopsis longicolla*
	Phyllosticta leaf spot	*Pleosphaerulina sojicola*
	Pod and stem blight	*Diaporthe phaseolorum* var. *sojae*
	Powdery mildew	*Erysiphe diffusa*
	Red leaf blotch^a^	*Coniothyrium glycines*
	Rhizoctonia damping-off and root rot^a^	*Rhizoctonia solani*
	Sclerotinia stem rot^a^	*Sclerotinia sclerotiorum*
	Sclerotium blight	*Sclerotium rolfsii*
	Seedling diseases^a^	*Fusarium* spp., *Alternaria* spp., *Pythium* spp. etc
	Soybean rust^a^	*Phakopsora pachyrhizi*
	Stem canker^a^	*Diaporthe phaseolorum* var. *caulivora; Diaporthe aspalathi*
	Sudden death syndrome^a^	*Fusarium virguliforme; F. tucumaniae; F. Brasiliense; F. crassistipitatum*
	Taproot decline^a^	*Xylaria necrophora*
	Target spot and root rot	*Corynespora cassiicola*
	Violet root and lower stem rot	*Rhizoctonia croccorum*
Bacterial diseases	Bacterial blight^a^	*Pseudomonas savastanoi* pv. *glycinea*
	Bacterial pustule^a^	*Xanthomonas axonopodis* pv. *glycines*
	Wildfire	*Pseudomonas syringae* pv. *tabaci*
Virus diseases	Alfalfa mosaic^a^	Alfalfa mosaic virus (AMV)
	Bean pod mottle^a^	Bean pod mottle virus (BPMV)
	Bean yellow mosaic	Bean yellow mosaic virus (BYMV)
	Brazilian bud blight	Tobacco streak virus (TSV)
	Cowpea mild mottle	Cowpea mild mottle virus (CMMV)
	Peanut mottle	Peanut mottle virus (PMV)
	Soybean dwarf^a^	Soybean dwarf virus (SbDV)
	Soybean mosaic^a^	Soybean mosaic virus (SMV)
	Soybean vein necrotic virus^a^	soybean vein necrotic virus (SVNV)

^a^Soybean diseases included in this review

In a recent report of soybean production losses caused by diseases in the USA and Canada from 2010 to 2014, yearly losses ranged from 10.06 to 13.92 million Mt (11.7–14.2% of total soybean production) (Allen et al. 2017). These losses are the result of many diseases caused by a range of fungi, bacteria, phytoplasmas, nematodes, and viruses. Recent meta-analyses of soybean disease losses in the USA over the last 24 years found that the greatest losses across states and years were from soybean cyst nematode (SCN) (*Heterodera glycines* Ichinohe), charcoal rot [*Macrophomina phaseolina* (Tassi) Goid], and seedling diseases (caused by several oomycetes and fungi) (Bandara et al. 2020; Roth et al. 2020). Important intermittent diseases caused by variations in the weather were Phytophthora root and stem rot (*Phytophthora sojae* Kaufmann & Gerdemann), sudden death syndrome (SDS) (*Fusarium virguliforme* O’Donnell and T. Aoki), and Sclerotinia stem rot [*Sclerotinia sclerotiorum* (Lib.)] (Roth et al. 2020). Root-knot nematode (*Meloidogyne* spp.), reniform nematode (*Rotylenchulus reniformis* Linford & Oliveira), and *Diaporthe* diseases were emerging diseases. Disease pressure appears to be increasing as greater yield losses have been observed over time (Bandara et al. 2020).

In Brazil, estimates in 1997 reported that the greatest disease losses were from stem canker (*Diaporthe aspalathi* (E. Jansen, Castl. & Crous) and *D. caulivora* (Athow & Caldwell) J.M. Santos, Vrandecic & A.J.L. Phillips), brown spot (*Septoria glycines* Hemmi), Cercospora leaf blight (CLB)/purple seed stain (PSS) [*Cercospora kikuchii* (Matsumoto & Tomoyasu) M. W. Gardner], and charcoal rot followed by soybean cyst nematode, seedling diseases, and Sclerotinia stem rot (Wrather et al. 1997). However, after soybean rust [*Phakopsora pachyrhizi* (Sydow. & Sydow.)] was introduced in Brazil in 2002, it quickly became the most suppressive soybean pathogen causing yield losses of nearly sixfold greater than CLB/PSS, the second most damaging disease in the country (Wrather et al. 2010). Soybean rust is particularly damaging in Brazil due to the year-round survival of the pathogen in production areas unlike in neighboring Argentina, where the pathogen must be re-introduced each year, therefore resulting in significantly less damage than in Brazil. The major soybean diseases in Argentina include SDS, charcoal rot, Cercospora leaf blight, brown spot, target spot [*Corynespora cassiicola* (Berk. & M.A. Curtis) C.T. Wei], and Sclerotinia stem rot. The most prevalent soybean disease in China is soybean mosaic virus (SMV). Other major diseases in China include frogeye leaf spot (*Cercospora sojina* Hara), SCN, anthracnose (*Colletotrichum* spp.), root rot (*P. sojae*, *Pythium* spp., *Fusarium* spp.), bacterial diseases, Sclerotinia stem rot, downy mildew [*Peronospora manshurica* (Naum.) Syd.], and soybean rust (Wrather et al. 1997, 2001, 2010). Prominent diseases in India include viruses, Sclerotium blight (*Sclerotium rolfsii* Sacc.), anthracnose (*Colletotrichum* spp.), and soybean rust (Wrather et al. 2010).

Russia and Ukraine are the most soybean productive countries in the world. Common soybean diseases in Russia include SCN, SMV, downy mildew, frogeye leaf spot, Phyllosticta leaf spot (*Pleosphaerulina sojicola* Miura, syn. *Phyllosticta sojicola* C. Massal.), CLB/PSS, brown spot, bacterial bustle (*Xanthomonas axonopodis* pv. *glycines*), and bacterial blight (*Pseudomonas syringae* pv. *glycinea* Coerper) (Bushnev et al. 2020; Sinegovskaya 2021). In Ukraine, SMV is a major concern which often infects together with bean yellow mosaic virus (BYMV), and Alfalfa mosaic virus (AMV) in the right-bank region (Kyrychennko et al. 2012; Mishchenko et al. 2017), while in the Forest-Steppe region, Alternaria leaf spot, downy mildew, Fusarium wilt and root rot, brown spot, and bacterial blight are the most prevalent soybean diseases (Sergiienko et al. 2021).

Africa and Australia represent geographical regions with the potential to become major soybean producers in the future (Hartman and Murithi, 2019). Africa produces about 1% of global soybean production (FAOSTAT, 2020). The major soybean diseases in Africa include soybean rust, frogeye leaf spot, red leaf blotch (*Coniothyrium glycines*), and SDS (Murithi et al. 2016; Hartman and Murithi, 2019). Australia produced 17,323 tons of soybean in 2020 (FAOSTAT 2020), and the major soybean diseases include charcoal rot, sclerotinia stem rot, Phytophthora root rot, and soybean rust (Ryley 2013).

In the future, soybean diseases may be continuously severe and difficult to manage, especially with the significant changes in the global climate (Roth et al. 2020). Since 1981, global temperatures have risen 0.18 °C per decade (www.climate.gov) and are expected to rise 6 °C by the next century (Mikhaylov et al. 2020). Temperatures and water precipitation are expected to increase in many areas (Tebaldi et al. 2006; Karl et al. 2009), but the increase in rainfall will be followed by more frequent extreme weather events as well as more frequent and severe droughts, making the overall weather patterns less consistent and predictable (Prein et al. 2021). It is estimated that rising temperatures have hindered agricultural production gains by 21% and made the management of plant diseases increasingly challenging (Jones 2021; Ortiz-Bobea et al. 2021). In the USA, it is predicted that climate changes may reduce average soybean yields by 86–92% by 2050 (Yu et al. 2021). These climate changes may alter the types, severities, and geographical distributions of soybean diseases, especially for the intermittent diseases that are heavily influenced by environmental factors, such as Phytophthora root and stem rot, SDS, and Sclerotinia stem rot (Roth et al. 2020).

Effective soybean disease management includes cultural practices (crop rotation, tillage, clean seed, etc.), chemical applications (foliar, seed, or soil), but the most important component is the deployment of resistant cultivars (Grau et al. 2004). Resistant cultivars can carry either vertical resistance, horizontal resistance, or both. Vertical resistance is contributed by resistance genes (R genes) for specific diseases, such as SCN (*Rhg*), Phytophthora root and stem rot (*Rps*), soybean rust (*Rpp*), frogeye leaf spot (*Rcs*), bacterial blight (*Rpg*), and SMV (*Rsv* and *Rsc*). R genes have been widely deployed conferring complete resistance to some pathotypes of the pathogen. The R genes typically follow a gene-for-gene interaction with the corresponding avirulence (Avr) factors from the pathogen, and resistance occurs only when the R gene and Avr factors both exist (Whitham et al. 2016). Therefore, R genes are pathotype (race)-specific, i.e., they may confer full protection to some pathotypes of the pathogen, while they are completely susceptible to others. R genes are often non-durable, and can be quickly overcome, due to the fast shift of the pathogen populations. For instance, the *Rpp1* and *Rpp3* genes mediated resistance to soybean rust were defeated the following year after the disease first occurred in Brazil in 2001 (Garcia et al. 2008; Langenbach et al. 2016). Another example is the *Rps1k* gene which has been traditionally deployed since the 1990s, can be defeated by most of the newly emerged pathotypes of *Phytophthora sojae* (McCoy et al. 2021). Although there are some exceptions such as *Rcs3* which has provided durable resistance against all known races of frogeye leaf spot in the USA (Boerma and Phillips 1983; Mian et al. 2008), searching for novel sources of resistance genes is a vital task for the deployment of vertical resistance and sustainability of the global soybean value chain.

In contrast, horizontal resistance (sometimes called partial resistance or tolerance) is quantitative and conferred by multiple minor effect genes and/or quantitative trait loci (QTL). Unlike vertical resistance that occurs only to some specific pathogens, horizontal resistance is widely involved in multiple soybean diseases and is known as the only type of resistance to many soybean diseases, including SDS, Sclerotinia stem rot, root-knot nematode, and most *Pythium* species. Horizontal resistance is usually considered pathotype non-specific (Dorrance et al. 2008; St. Clair 2010; Mundt 2014; Nelson et al. 2018; Karhoff et al. 2019), although some isolate specific QTLs have also been identified in soybean (Lee et al. 2014; Stasko et al. 2016; Lin et al. 2021). Therefore, horizontal resistance is considered more durable.
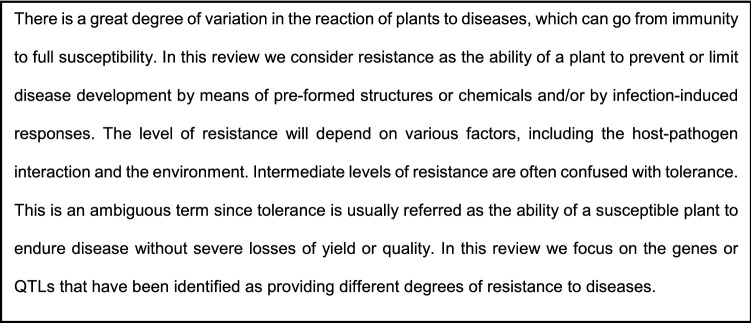


The traditional introgression of resistance genes into resistant cultivars can take more than ten years starting from making crosses between the recurrent parents and the resistance donor parents. Fortunately, with the development of molecular marker technology, especially with the sequencing of the soybean genome and the development of low cost of high-throughput genotyping (such as the BARCSoySNP6K and BARCSoySNP50K iSelect BeadChips), breeders can make selections more efficiently and accurately (Song et al. 2013, 2020). Marker-assisted selection (MAS) has proved to be the most successful approach in the selection of R genes or major QTLs (Ribaut and Hoisington 1998). The markers used for MAS have evolved from the low-efficiency restriction fragment length polymorphism (RFLP) markers to simple sequence repeat (SSR) markers, and currently, to more efficient and cost-friendly SNP markers in modern soybean breeding programs. However, for minor effect QTLs, genomic selection (GS) has been demonstrated to outperform MAS with higher accuracy and efficiency (Bao et al. 2014; Wen et al. 2018). For example, Bao et al. (2014) genotyped 282 soybean accessions for resistance to SCN HG type 0 and discovered that GS using full marker set produced significantly more accurate predictions than MAS using two rhg1-associated DNA markers. In another study for soybean resistance to white mold (Wen et al. 2018), the GS prediction accuracy was estimated at 0.64, which was significantly higher than that of MAS (0.47–0.51), although MAS was still 24–26% higher than using random SNPs. Moreover, with the recent development of new technologies such as GWA studies, numerous SNP markers have been identified for soybean resistance against various diseases and have the potential to be deployed in the future (Wen et al. 2014; Vuong et al. 2015; Zhang et al. 2015a; Chang et al. 2016; Rincker et al. 2016a; Coser et al. 2017; Moellers et al. 2017; Lin et al. 2020). On the other hand, genome-editing technology (such as CRISPR/Cas9) allows plant breeders to fine-tune gene regulation toward the improvement of crop resistance to various diseases (Chen et al. 2019).
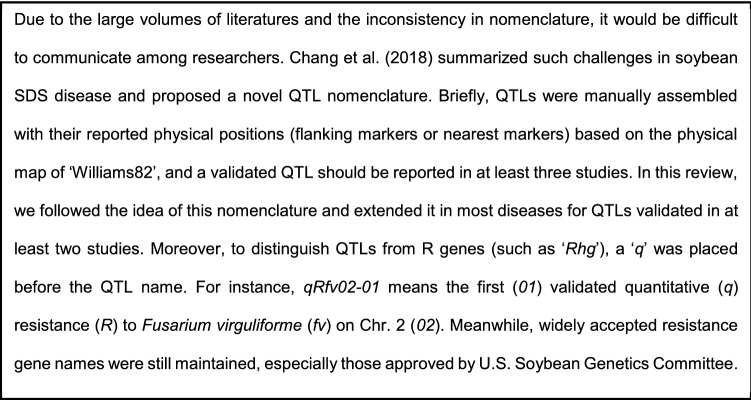


To assist soybean breeders to develop effective breeding strategies under the global climate change, reducing the world soybean yield loss due to diseases and ensure the continuous growth and sustainability of the global soybean production in the next decade, this review aims to: 1. provide comprehensive atlas of soybean genes and QTLs conferring resistance to 28 economically important and emerging diseases, including their donor source, genetic position, tightly linked markers, resistance spectrum, and testing methods; 2. validate high-quality QTLs across different studies based on the overlapping of their genomic positions; and 3. offer comprehensive future perspectives and breeding suggestions for disease-related pipelines. This review may also serve as a guideline and toolbox for soybean breeders around the world.

## Section I. Soybean resistance to nematode diseases

Plant–parasitic nematodes are the major constraints for soybean production worldwide. Nematodes alone are responsible for a projected loss of $78 billion annually worldwide with a 10–15% average yield loss in soybean (Lima et al. 2017). The intensity of yield loss caused by parasitic nematodes are variable and typically depends on several factors including the nematode species, the nematode population density, management practices, the genetic background of soybean varieties, and soil and environmental factors (Bradley et al. 2021). In recent decades, nematode infestation has been spread in most soybean producing countries in the world including the USA, Brazil, Canada, South Africa, Japan, China, and India. Soybean cyst nematode, southern root-knot nematode, reniform, and lance nematodes are the major plant–parasitic nematodes in soybean around the world resulting in losses of as much as 100% (Wrather and Koening 2009; Kim et al. 2016; Bradley et al. 2021). The detailed information of each specific nematode and breeding efforts to enhance the levels of resistance is described below.

## Soybean cyst nematode

Among plant–parasitic nematode species, soybean cyst nematode (SCN, caused by *Heterodera glycines* Ichinohe) is the most destructive sedentary and obligate parasite of soybean causing up to 30% yield loss (Mueller et al. 2016). The annual production losses caused by SCN are more than twice as much as any other diseases in North America, causing projected yearly losses of billions of dollars worldwide. In 1915, Japan reported the first occurrence of SCN, and later in 1954 it was identified in North Carolina, USA (Winstead et al. 1955; Riggs, 2004), and later in Ontario, Canada (Anderson et al. 1988). Subsequently, it spread to most soybean-producing countries causing severe yield losses worldwide. For instance, more than 3.5 million Mt of production losses caused by SCN were reported in 28 states of the USA (Koenning and Wrather 2010; Allen et al. 2017) corresponding to more than $1 billion in value (Liu et al. 2012). Later, SCN infestation was identified in Quebec province, Canada (Mimee et al. 2015) and some of the soybean cultivated provinces in China (Peng et al. 2016).

While crop damage due to SCN is devastating, the symptoms above the ground level are not every time noticeable, and infestations are typically only identified in the advanced phase of infection. At this stage, a significant amount of damage has already taken place. Symptoms include chlorosis, stunting, reduced root development, and decreased nodule formation (Niblack et al. 2006). Several traditional practices including biological, chemical, and physical methods have been attempted to control SCN infestation but were found inadequate for the management of the disease. The development and deployment of resistant cultivars along with crop rotation methods are the preferably efficient practice for the management of SCN (Davis and Tylka 2000).

Breeding for SCN resistance involves the genetic mapping of QTLs/genes associated with the resistant phenotype and understanding the underlying resistance mechanism. The first *Rhg* (resistance to *H. glycines*) locus was reported around the mid-1950s (Ross and Brim 1957) which described plant introductions (PIs) 88788 and ‘Peking’ (PI 548402) as sources of SCN resistance. These two accessions were integrated into the soybean breeding programs through cycles of backcrossing. With the rapid progress in the availability of molecular markers and mapping techniques, numerous SCN-resistance loci have been reported by the soybean research community. Table 2 summarizes the main reported QTLs linked to SCN resistance. In soybean, SCN resistance trait is typically multi-genic and quantitatively inherited (Anand and Rao-Arelli 1989; Guo et al. 2005; Vuong et al. 2010, 2011). The resistance found in Peking was governed by three independent recessive genes (Caldwell et al. 1960). Since then, numerous genes/QTLs conferring SCN resistance have been mapped to date. Among these QTLs, two loci *rhg1* and *Rhg4* found on chromosomes 18 and 8, respectively, which confers resistance to SCN races 1, 2, 3, 4, and 5, have been extensively investigated (Kim et al. 2016). In diverse soybean germplasm lines, the *rhg1* locus has been constantly mapped and identified at a sub-telomeric region on the chromosome (Chr.) 18 (Kim et al. 2016). Using *rhg1*, several markers were developed, of which Satt309 (predicted at about 0.4 cM from *rhg1* locus) has been extensively applied for MAS in soybean research (Cregan et al. 1999; Silva et al. 2007). Another major QTL for SCN resistance showed a total phenotypic variation of about 9–28% to SCN HG types 2.5.7 (race 1) and 0 (race 3) and was described as *Rhg4* gene from different resistant plant accessions (Concibido et al. 2004). Meksem et al. (2001) described that *rhg1* and *Rhg4* equally demonstrated about 98% of phenotypic variation in the ‘Forrest’ cultivar conferring resistance to race 3 of SCN. *Rhg4* mediated resistance is largely associated with race 3 of SCN, in addition to some minor resistance against race 2 (HG types 1.2.5.7), race 1 (2.5.7), and race 14 (1.3.6.7). In Peking and PI 437654 accessions, *rhg1* and *Rhg4* loci are essential to provide complete resistance against some SCN races. QTL mapping in PI 567516C identified two SCN-resistance QTLs on chromosomes 10 and 18, which were not linked to major *rhg1* or *Rhg4* loci (Vuong et al. 2010). These QTLs conferred resistance against races 1, 2, 3, and LY1 of SCN (Young 1998). Interestingly, the QTL detected on Chr. 18 is far away from the *rhg1* locus. Another two QTLs were mapped on chromosomes 10 and 18 in PI 567305 (Kim et al. 2016) and were showing elevated resistance to various SCN HG types, identical with the study demonstrated by Vuong et al. (2010) in PI 567516C. Therefore, these results indicated that both PI 567305 and PI 567516C harbor novel QTLs which can provide SCN resistance. Recently, the genetic analysis of the PI 567305 line through Infinium SoySNP6K BeadChips and genotype-by-sequencing (GBS) revealed major QTLs on chromosomes 10 and 18 (Vuong et al. 2021) conferring resistance to SCN as well as other two important nematode species such as root-knot and reniform nematodes. The unique genetic structure of PI 567305 investigated using haplotype and copy number variation analysis suggested the presence of different resistance mechanisms from PI 88788 or Peking-type.

**Table 2:** Validated genes/loci conferring resistance to soybean cyst nematode disease (caused by *Heterodera glycines*)

Locus/Allele name	MLG (Chr.)	Tightly linked/flanking markers	Marker position (Gmax2.0)	Resistance spectrum^a^	PVE^b^ (%)	Population type (size)	Screening environment	Donor source	References
*Rhg4*	MLG A2 (Chr. 8)	*I* locus	6,638,879–8,684,157	Race 3	–	F6:7 (328)	Greenhouse	PI 437654	Webb et al. (1995)
		Satt632—SIUC-100–8		Race 1	7.3	F2:3 (250)		PI 438489B	Vuong et al. (2011)
		Satt632—SIUC-100–8		Race 3	13.4	F2:3 (250)		PI 438489B	
		–		Race 1	7.8	F2:3 (160)		SS97-6946	Islam et al. (2015)
				Race 3	9.2	F2:3 (160)		SS97-6946	
–	MLG J (Chr.16)	*B032*	33,522,390–37,517,727	Race 3	40.2	F2 (56)	Greenhouse	PI 209332	Concibido et al. (1994)
–	MLG A2 (Chr. 8)	*A085*	4,113,588–5,287,036		21.4				
–	MLG A2 (Chr. 8)	Satt233–Sat_040	1,621,167–6,169,649	Race 2	7	F2:3 (250)	Greenhouse	PI 567516C	Vuong et al. (2010)
*qSCN18*	MLG G (Chr. 18)	Satt612–Satt191 and Sat_210–Sat_403	52,048,435–57,332,1221,496,949–6,332,269	Race 1, 2, 3, 5, 14 and LY1	5.2–10.4	F2:3 (250)	Greenhouse		
*qSCN10*	MLG O (Chr. 10)	Sat_038–Satt592 and Sat_274–Satt592	41,364,845–47,422,630	Race 1,2,3 and LY1	7.9–21.7	F2:3 (250)	Greenhouse		
*qSCN-PL10*	MLG O (Chr. 10)	Marker1015215	42,660,451–42,881,952	HG type 7	7.73	F2:3 (200)	Greenhouse	Pingliang ZDD 11,047	Guo et al. (2020)
*–*	MLG B1 (Chr. 11)	–	–	Race 3	~ 91	F2:3 (200)	Greenhouse	Hartwig	Vierling et al. (1996)
		A006–Satt583	10,325,463–26,020,924	Race 1	16.6	F2:3 (250)	Greenhouse	PI 89772	Yue et al. (2001b)
*–*				Race 2	6.8				
		A006-A118	10,344,240–25,063,291	Race 5	9.5				
*rhg1*	MLG G (Chr. 18)	B053–Satt309	1,696,762–2,011,402	Race 1	26.6				
				Race 3	23.0				
				Race 5	10.0				
	MLG E (Chr. 15)	A135—Satt231	1–36,327,770	Race 3	15.7				
*–*	MLG D2 (Chr. 17)	B132–Satt372	2,703,565–6,607,864	Race 1	9.7				
*qSCN001-01*	MLG M (Chr. 7)	ss107925701–ss107918678	176,243–2,483,443	Race 3	22.4	F6:13	Greenhouse	PI 438489B	Abdelmajid et al. (2014)
*qSCN001-02*	MLG F (Chr. 13)	ss107920816–ss107912529	1,021,174–10,404,020	Race 3	8.9		Greenhouse		
*qSCN001-03*	MLG E (Chr. 11)	ss107913532–ss107930960	11,523,094–15,075,151	Race 3	16.1		Greenhouse		
*qSCN002-01*	MLG A1 (Chr. 5)	ss107921684–ss107919814	1–2,725,084	Race 5	43.0	F6:13	Greenhouse	PI 438489B	
*qSCN002-02*	MLG A2 (Chr. 8)	ss107919498–ss107930668	2,487,792–3,609,192	Race 5	43.3	F6:13	Greenhouse	PI 438489B	
*qSCN002-03*	MLG B1 (Chr. 11b)	ss107920383–ss107922154	9,979,503–11,537,679	Race 5	38.9	F6:13	Greenhouse	PI 438489B	
–	MLG G (Chr. 18)	Satt163–Satt688	883,910–3,341,873	Race 2	14.7	F2:3 (226)	Greenhouse	PI 90763	Guo et al. (2005)
			883,910–3,341,873	Race 3	28.1	F2:3 (226)			
			883,910–3,341,873	Race 5	13.0	F2:3 (226)			
–	MLG B1 (Chr. 11)	Satt453–Satt359	32,411,307–34,173,104	Race 2	6.7	F2:3 (226)	Greenhouse	PI 90763	
				Race 5	11.2	F2:3 (226)			
–	MLG A2 (Chr. 8)	Satt400-Satt424	7,678,989–10,846,818	Race 3	17.7	F2:3 (226)			
–	MLG E (Chr. 15)	Satt573-Satt204	14,438,759–17,330,815	Race 5	12.5	F2:3 (226)			
*rhg1*	MLG G (Chr. 18)	Satt038-Satt309	1,295,211—2,467,798	Race 3	0.61	F7 and F8 (115)		Toyomusume	Ferdous et al. (2006)
*rhg-t1*	MLG B1 (Chr. 11)	Satt453-Sat_331	32,911,928–34,766,867		0.12				
*rhg-t2*	MLG B1 (Chr. 11)	Satt583	26,440,896–30,354,966		0.04				
–	MLG B1 (Chr. 11)	Satt583—Sat_123	27,142,236–33,060,032	Race 1	47.3	F2 (184)		PI 438489B	Yue et al. (2001b)
		Satt583—Sat_123		Race 2	45.8				
		Satt168—A329		Race 3	51.5				
		Satt583—Sat_123		Race 5	34.5				
		–		Race 14	37.2				
*rhg1*	MLG G (Chr. 18)	Satt309–Sat_168	1,661,117–1,785,434	Race 1	26.2	F2:3	Greenhouse	Peking	Concibido et al. (1997)
				Race 3	44.8	F2:3		PI90763	
				Race 3	6.4	F5:6 (739)		Hartwig	Prabhu et al. (1999)
				Race 1	15.0	F2:3 (250)		PI 438489B	Vuong et al. (2011)
				Race 2	8.7	F2:3 (250)		PI 438489B	
				Race 3	27.9	F2:3 (250)		PI 438489B	
				Race 1	18.8	F2:3 (160)		SS97-6946	Islam et al. (2015)
				Race 3	9.5	F2:3 (160)		SS97-6946	
*rhg1*	MLG G (Chr. 18)	–	1,697,102–2,467,798	–	52.7 51.4 40.0 28.8	F2:3 (76)	Greenhouse	PI 90763 PI 20933 PI 88788 Peking	Concibido et al. (1997)
*Rhg1-b*	MLG G (Chr. 18)	–	1,710,006–2,011,402	TN14 (Race 2)	–	F3 (80)	Greenhouse	PI 88788	Brucker et al. (2005)
–	MLG B2 (Chr. 14)	A593_1	45,067,577–47,207,943	Race 1,3,5	14–57.7	F2:3 (200)	Greenhouse	Peking	Qiu et al. (1999)
–	MLG D2 (Chr. 17)	–	17,878,150–27,906,833	Race 14	9.5–41.1	BC3F2:3 (126)	Greenhouse	Hartwig	Schuster et al. (2001)
*cqSCN10*	MLG O (Chr. 10)	Satt592, Satt331, and Sat_274	41,610,215–41,958,155	Race 2	13.5	RILs (242)	Greenhouse	PI 567305	Vuong et al. (2021)
				Race 3	34.5				
				Race 5	5.6				
*cqSCN11*	MLG B1 (Chr. 11)	–	35,925,243–37,749,863	Race 2	3.5	RILs (242)	Greenhouse	PI 567305	
				Race 5	5.1				
*cqSCN18*	MLG G (Chr. 18)	–	1,010,310–2,178,121	Race 2	22.5	RILs (242)	Greenhouse	PI 567305	
				Race 3	7.9				
				Race 5	23.0				

^a^Conversion of races to HG types are: Race 1 (HG type 2.5.7), race 2 (HG type 1.2.5.7), race 3 (HG type 0), race 5 (HG type 2.5.7), race 14 (1.3.5.6.7), race 14 (HG type 1.3.6.7), race LY1 (1.2.3.4.5.6.7)

^b^Phenotypic variations explained by the molecular markers

In addition, three resistance loci for race 3 of SCN were detected in a GWA study of 282 soybean accessions, among which two out of these three were correlated to *rhg1* and earlier mapped, FGAM1, SCN-resistance locus whereas the third one was positioned at Chr. 18 (Zhang et al. 2017). About 8 novel QTLs for resistance to race 3 of SCN was also identified by Vuong et al. (2011). Furthermore, 13 significant SNPs for SCN resistance were also identified in 7 diverse genomic regions by Zhang et al. (2017). Out of these 13, 10 SNPs were novel, whereas the remaining 3 were linked to earlier mapped QTLs including *rhg1* and *Rhg4*. An investigation performed by Zhao et al. (2017) demonstrated the identification of 13 important SNPs (4 novels) on five chromosomes which conferred resistance to SCN race 1. Later, twelve SNPs significantly linked to SCN resistance were identified on chromosomes 7, 8, 10, and 18. Of these twelve, three were positioned close to the *rhg1* locus (Tran et al. 2019). Using these data, multiple candidate genes conferring SCN resistance have been discovered. Liu et al. (2019) described 10 genes having 27 mutations, among which three genes overlapped between the two phenotypic mutants suggesting possible involvement of these genes in nematode resistance.

The copy number of *rhg1* has been categorized into two repeat types such as high (> 6 repeats, as in PI 88788) and low (about 3 repeats, as in Peking) (Cook et al. 2012). Yu et al. (2016) demonstrated that, in the case of *rhg1*, both gene-based polymorphism and copy number variation were significantly important for SCN resistance. It also indicated that *rhg1* resistance sources with a high copy number provided elevated resistance against SCN. Altogether it was proposed that *rhg1* locus may facilitate SCN resistance through copy number variation of numerous genes encoding amino acid transporter (AAT), a WI12 (wound-inducible) protein, and an α-soluble N-ethylmaleimide-sensitive factor (NSF) Attachment Protein (α-SNAP) (Kandoth et al. 2017; Liu et al. 2017). Furthermore, Patil et al. (2019) categorized the *rhg1*-*b* locus into two classes, *rhg1*-*b* (like lines of PI 88788-type) and *rhg1*-*b1* (like lines of Cloud-type) and revealed genetic basis of broad-spectrum resistance through interactions of copy number variation among *rhg1* and *Rhg4* genes. Liu et al. (2012) reported that the resistance at the *Rhg4* locus was provided through the serine hydroxymethyltransferase (*SHMT*) gene, whose encoding protein catalyzes the reversible conversion of tetrahydrofolate and serine to tetrahydrofolate and glycine, respectively. The two polymorphisms in the gene *GmSHMT08* positioned at the first and second exons, 389 G/C and 1165 A/T, results in modification of amino acids such as arginine vs. proline and tyrosine vs. asparagine, respectively, and further alteration of the kinetic properties (Liu et al. 2012). *GmSHMT08* encoded protein shows a multifarious role in addition to essentially being involved in the enzymatic reaction of SCN resistance (Kandoth et al. 2017). It has additional functions including structural stability, ligand binding, and interactions with other proteins (such as GmSNAP18). Kandoth et al. (2017) showed that *rhg1-a* allele is required in Forrest cultivar for SCN resistance although it does not impart any selection pressure on nematodes to shift from HG type 7. However, the nematodes were exposed to EXF67 cv. shifted to HG type 1.3.6.7 indicating the bi-genic phenomenon of resistance and necessity of *Rhg4* in Peking-type facilitating resistance.

Cook et al. (2014) showed the distribution of nonsynonymous SNPs in the *GmSNAP11* gene, its paralogous copy identified as *GmSNAP18*, with novel alleles that participated in SCN resistance, especially α-SNAP is crucial for resistance in soybean varieties derived from PI-88788. Further, Lakhssassi et al. (2017) demonstrate that the predicted protein of α-SNAP corresponds to truncated GmSNAP11 and not to GmSNAP18 (289 amino acids, aa). GmSNAP11 exists in Forrest Pecking type in two different forms such as GmSNAP11-T1 (239 aa) and GmSNAP11-T2 (244 aa). A nonsynonymous SNP known as *map-5149* tightly linked to resistance against race 3 of SCN was identified in *GmSNAP11* (Li et al. 2016a, b, c). Altogether, these results suggest the novel nature of *GmSNAP11* providing SCN resistance in soybean.

Marker-assisted selection (MAS) is an effective and routinely performed strategy to develop SCN resistant soybean lines, representing the most rapid, cost-effective, accurate, and reliable method. Shi et al. (2015) developed functional Kompetitive Allele-Specific PCR (KASP) marker assays (*GSM381* and *GSM383* at *rhg1; GSM191* at *Rhg4*) which were effectively applied for rapid and quick selection of SCN resistance, as well as identification of Peking and PI 88788 types of resistance. Kadam et al. (2016) developed KASPar (KBioscience Competitive Allele-Specific PCR) assays from SNPs at *rhg1*, *Rhg4*, and other novel QTLs. They effectively differentiated the copy number variation at *rhg1* into three groups including (1) high resistant such as PI 88788 type, (2) low copy resistant such as Peking type, and (3) susceptible single copy such as Williams82 type numbers. Tian et al. (2019) developed cleaved amplified polymorphic sequences (CAPS) markers using *GmSNAP11* (minor resistant to SCN) and combined with markers Rhg-389 and rhg1-2 for genotyping a panel consisting of 209 soybean accessions with variable SCN resistance.

The underlying molecular mechanisms of SCN resistance are complex and yet to be unveiled. Some studies suggested that there could be several disease-resistance proteins involved in SCN resistance, comprising Nucleotide-binding site-leucine-rich repeats (NBS-LRR), cytochrome P450s, RING domain proteins, zinc-finger domain proteins, protein kinases, transcription factors such as MYB and WRKY. Kofsky et al. (2021) studied the transcriptome of wild SCN resistant soybean (*Glycine soja*) ecotype, ‘NRS100’, and proposed biochemical mechanisms. This included the downregulation of the jasmonic acid (JA) signaling pathway to permit resistance response led by salicylic acid (SA) signaling-activation and polyamine synthesis which further maintains structural stability of root cell walls.

## Soybean root-knot nematode

Root-knot nematodes (*Meloidogyne* spp.) are considered the most economically important and widely distributed parthenogenic plant–parasitic nematodes in the world (Trudgill and Blok 2001). Southern Root-knot nematode [SRKN, *M. incognita* (Kofold & White) Chitwood] was considered as one of the major plant–parasitic nematodes based on scientific and economic importance (Jones et al. 2013). The observed symptoms of SRKN in soybean are similar with the symptoms of abiotic stresses, including stunted growth, wilting, leaf discoloration, and deformation of the roots. The magnitude of crop losses depends on historical crop rotation and field usage, environmental parameters, initial nematode population density, soil type, and genetic background (Vieira et al. 2021).

SRKN is challenging to control due to its short life cycle and high reproductive rates (Trudgill and Blok 2001). Chemical approaches used to be an effective management option, however, most commercial nematicides and soil fumigants were banned due to toxicity to humans, animals, and environments (Abad et al. 2008). Crop rotation is especially challenging and limited since most flowering plants are hosts to SRKN. The use of genetic resistance becomes the most sustainable—economically, environmentally, and socially—alternative to efficiently control the damage caused by SRKN in soybean (Vieira et al. 2021).

Significant efforts have been taken to identify soybean accessions resistant to SRKN. Luzzi et al. (1987) screened over 2700 soybean accessions from the USDA Soybean Germplasm Collection and found that ‘Amredo’, PI 96354, PI 408088, and PI 417444 showed lower gall indices, fewer eggs per root system, and eggs per gram of root than the resistant check Forrest (PI 548655) (Luzzi et al. 1987). Harris et al. (2002) screened 608 PIs from Southern China and reported that PI 594753A and PI 594775A had similar resistance levels as PI 96354 (Harris et al. 2002). The first report on the genetic control of the resistance to SRKN indicated that reduced galling in the cultivar Forrest was controlled by a single dominant gene designated as *Rmi1* (Luzzi et al. 1994a). Hybridizations between PI 96354 × Forrest and Forrest × PI 417444 resulted in individual F_3_ plants and F_3_ populations with higher galling than Forrest, PI 96354, and PI 417444, implying the resistance from Forrest (*Rmi1*) differs from PI 96354 and PI 417444 by at least one gene (Luzzi et al. 1994b).

The first genetic mapping of resistance to SRKN (race 3) in soybean identified two QTLs on chromosomes 10 and 18, accounting for 31% and 14% of phenotypic variation, respectively (Tamulonis et al. 1997). The combination of both resistance QTLs enhanced the levels of resistance to SRKN race 3, the predominant race in the U. S. (Li et al. 2001a). An additional major QTL on Chr. 7 accounting for 62% of the phenotypic variation was reported to confer resistance to SRKN race 2, a predominant race in soybean production areas of South Africa (Fourie et al. 2008). In addition, two minor QTLs on Chr. 8 (7.4% of the phenotypic variation) and 13 (5.6% of the phenotypic variation) were reported to confer resistance to SRKN race 3 (Xu et al. 2013) (Table 3).

**Table 3:** Soybean loci conferring high resistance to southern root-knot nematode (caused by *Meloidogyne* spp.)

MLG (Chr.)	Locus/allele name^a^	Tightly linked/flanking markers	Marker position (Gmax2.0)	Testing methods/Resistance spectrum	PVE^b^	Population type (size)	Donor source	References
MLG C2 (Chr. 6)	–	Satt286 and Satt365	16,200,000–19,600,000	Greenhouse test/Race *3*	–	F2:4 (35)	PI 96354	Shearin et al. (2009)
MLG M (Chr.7)	*–*	Satt201 and Satt590	1,301,315–2,025,244	Greenhouse test/Race *2*	62.4%	F2:3 (69)	LS 5995	Fourie et al. (2008)
MLG A2 (Chr. 8)	*–*	BARC-051847–11,270 and BARC-039273–07,476	22,048,168–35,856,368	Greenhouse test/Race *3*	6.4%	F8:9 (246)	PI 438489B	Xu et al. (2013)
MLG O (Chr. 10)	*qRmi10-01*	G248A-1	1,018,664–1,881,027	Greenhouse test/Race *3*	31%	F2:3 (110)	PI 96354	Tamulonis et al. (1997)
		Satt492 and Satt358	1,018,664–1,881,027	Greenhouse test/Race *3*	55.8%	F2:3 (110)	PI 96354	Li et al. (2001a)
		Satt500 and Satt358	1,018,500–1,395,790	Greenhouse test/Race *2*	31.7%	F2:3 (69)	LS 5995	Fourie et al. (2008)
		BARC-065469–11,494 and BARC-018101–02,517	1,571,105–2,067,005	Greenhouse test/Race *3*	23.6%	F8:9 (246)	PI 438489B	Xu et al. (2013)
		BARCSOYSSR-10–0090 and BARCSOYSSR-10–0105	1,470,000–1,640,000	Greenhouse test/Race *3*	50%	F5:6 (269)	PI 96354	Pham et al. (2013)
		ss715605654	1,507,123–1,519,325	Greenhouse test/Race *3*	–	PI Panel (193)	PI 96354	Passianotto et al. (2017)
MLG F (Chr. 13)	*–*	BARC-010501–00,676 and Sct-033	28,826,405–30,078,140	Greenhouse test/Race *3*	4.8%	F8:9 (246)	PI 438489B	Xu et al. (2013)
MLG G (Chr.18)	*qRmi18-01*	K493h-1 and Cs008D-1	47,201,155–50,158,095	Greenhouse test/Race *3*	14.4%	F2:3 (110)	PI 96354	Tamulonis et al. (1997)
		Satt012 and Satt505	47,201,155–50,158,095	Greenhouse test/Race *3*	17.7%	F2:3 (110)	PI 96354	Li et al. (2001a)
		ss715631954	47,201,155–50,158,095	Greenhouse test/Race *3*	5%	F5:6 (269)	PI 96354	Pham et al. (2013)

^a^Locus name given in this study, if the physical positions of QTLs overlap each other in at least two independent studies. *qRmi10-01* means the 1st (01) quantitative (q) resistance (R) to *M. incognita (mi)* on Chr. 10 (10)

^b^Phenotypic variations explained by the molecular markers

To better understand the mechanisms of soybean resistance to root-knot nematode, fine-mapping analyses were conducted for the major QTL on Chr. 10. Pham et al. (2013) identified three candidate genes with cell wall modification-related functions, including *Glyma.10g016600* (Extensin 1 encoding function), *Glyma.10g016700* (Extensin 2 encoding function), and *Glyma.10g017100* (Pectinesterase 1 encoding function). In another independent study, five candidate genes were identified, including *Glyma.10g017100, Glyma10g02150, Glyma.10g017200, Glyma.10g017300,* and *Glyma.10g017400*, all with pectinesterase encoding-related functions (Xu et al. 2013). Moreover, a GWA study using a panel of diverse soybean accessions narrowed down this QTL to a 12-kb region with five significant single nucleotide polymorphisms (SNPs) located within *Glyma.10g017100* accounting for 25 to 40% of phenotypic variations (Passianotto et al. 2017).

Multiple reports have shown that SRKN resistant soybean genotypes can sustain yield under variable levels of nematode infection. Yield suppression can reach as much as 97% in susceptible genotypes while resistant genotypes may show less than 1% (Herman et al. 1990). Kinloch et al. (1984) reported a negative correlation between yield and number of galls under high pressure, which translated in resistant cultivars yielding as much as 5 times greater than highly susceptible cultivars (Kinloch et al. 1984). Vieira et al. (2021) evaluated the yield performance of 202 elite soybean lines in field conditions with variable distributions of SRKN and reported resistant lines yielding on average 20% higher than susceptible lines. The presence of the major resistance allele on Chr. 10 reduced yield losses by approximately sixfold in comparison to the susceptible group (1.1% and 6.2% per 1000 SRKN second-stage juveniles in 100 cm^−3^, respectively), which provided significant yield protection under high SRKN pressure (Vieira et al. 2021). However, because of the high concentration and wide distributions of SRKN, the limited and narrow base of genetic resistance, and lack of alternative management options, a resistance-breaking population in soybean could result in devastating yield losses (Vieira et al. 2021). Consequently, further work is needed to unveil and stack novel sources of resistance resulting in enhanced and more durable resistance in the future (Vieira et al. 2021).

## Reniform nematode and Lance nematode

Reniform nematode (*Rotylenchulus reniformis* Linford & Oliveira) (RN), a sedentary semi-endoparasite, first emerged in Hawaii on cowpeas [*Vigna unguiculata* (L.) Walp.] in 1931 and was identified in Georgia, USA, in 1940 (Linford and Oliveira 1940; Smith 1940; Gavilano et al. 2013). It has now become a major yield-limiting parasitic nematode species in soybean growing areas in southern and southeastern states of the USA, due to its wide range of hosts (over 300 plant species), and the ability of surviving in broad soil range and dry soil for an extended period (Herald and Thames 1982; Herald and Robinson 1990; Wrather et al. 1995; Robinson et al. 1997; Robbins et al. 1999; Koenning and Wrather 2010). The infestation on the roots of the host is initiated by the vermiform female adults, which is different from common sedentary endo-parasitic nematode genera (*Heterodera, Globodera*, and *Meloidogyne*). Female RN establish feeding sites known as syncytium and eventually become sedentary. The common name of RN refers to its kidney shape characteristics. The male RN are involved in mating but do not feed (Linford and Oliveira 1940; Gaur and Perry 1991; Ganji et al. 2013; Robbins 2013). Typical symptoms of RN infection include root decay, stunting, and foliar chlorosis (Cook et al. 1997; Kinloch 1998; Rivera and Thiessen 2020). Annual soybean yield losses of up to 33% were reported in soybean cultivars that were partially or not resistant to RN, resulting in an average loss of 28,000 Mt in southern USA in 2019 (Kim et al. 2016; Allen et al. 2020). Like other nematode pests, deployment of resistant varieties has been the most effective and economical strategy to control RN in soybean field (Kim et al. 2016).

The relationship between SCN and RN has drawn interest since they both establish syncytium as their feeding sites. Early literatures reported that there were common sources of resistances for SCN and RN (Rebois et al. 1970). Field and greenhouse screening assays were subsequently conducted, and the studies indicated that soybean cultivars that derived their resistance from PI 88788 were resistant to SCN but susceptible to RN whereas cultivars that derived their resistance from Peking and PI 437654 were resistant to both SCN and RN (Robbins et al. 1994a, 1994b, 1999; Robbins and Rake 1996). Greenhouse screening assays were commonly used to evaluate RN resistance for soybean. Disease screening protocol for RN was well-established by Robbins et al. (1999), in which the reproductive index (RI) was calculated based on the number of nematodes at test termination (Pf) and initial infestation density (Pi) (RI = Pf/Pi). High level of RN resistance has been reported in soybean cultivars including Peking, ‘Dyer’, ‘Custer’, Pickett’, Forrest, ‘Hartwig’, and ‘Anand’ (Rebois et al. 1968; Robbins et al. 1994b; Davis et al. 1996). Lee et al. (2015) also reported RN resistance in PI 404198A, PI 438498, PI 467327, PI 468915. PI 494182, PI 507470, PI 507471, PI 507476, and PI 567516, all showing similar or less RI than the resistant check Anand.

Three QTLs conferring RN resistance in soybean have been identified on chromosomes 11, 18, and 19, respectively, from PI 437564 (Ha et al. 2007). Other studies have reported and confirmed resistant loci on chromosomes 8 (Lee 2021), 11 (Jiao et al. 2015; Wilkes et al. 2020; Usovsky et al 2021), 12 (Lee et al. 2016), 13 (Lee 2021), 15 (Lee 2021), and 18 (Jiao et al. 2015; Lee et al. 2016; Wilkes et al. 2020; Lee 2021; Usovsky et al. 2021). Recently, Usovsky et al. (2021) discovered the pleiotropic effect of two genes [*GmSNAP18* (*rhg1-a, rhg1-b, and rhg1-b1* allele) and *GmSNAP11* (*qSCN11* locus)], conferring resistances to both SCN and RN in PI 438489B using universal soybean linkage panel (USLP 1.0) and next-generation whole-genome resequencing (WGRS) technology (Table 4).

**Table 4:** Soybean loci conferring resistance to reniform nematode (caused by *Rotylenchulus reniformis*)

MLG (Chr.)	Locus/allele name^a^	Other name	Tightly linked/flanking markers	Marker position cM (bp)^b^	Testing methods/Resistance spectrum	Population type (size)	PVE^c^	Donor source	References
MLG B1 (Chr. 11)	*qRrr11-01*	GmSNP11	Sat_123 and BARC-018869–03,031	(32,194,583–33,581,636)	Partial Greenhouse assay	F8 (247)	11.3%	PI 438489B	Usovsky et al. (2021)
		–	Satt359	102.55 cM* (32,411,307)	Partial Greenhouse assay	F6 derived RILs (228)	16%	PI 437654	Ha et al. (2007)
	–	–	BARC-021459–04,106	120.13 cM* (38,902,736)	Partial Greenhouse assay	F6:9 (247)	8.9%	PI 567516C	Jiao et al. (2015)
MLG H (Chr. 12)	–	–	Satt353	8.48 cM* (1,687,387)	Partial Greenhouse assay	F5:16 (92)	10%	Hartwig (PI 437654)	Lee et al. (2016)
MLG G (Chr. 18)	–	–	Satt163	0 cM*	Partial Greenhouse assay	F5:16 (92)	13.5%	Hartwig (PI 437654)	Lee et al. (2016)
	–	–	Satt275	2.2 cM*	Partial Greenhouse assay	F5:16 (92)	10%	Hartwig (PI 437654)	Lee et al. (2016)
	*qRrr18-01*	GmSNP18	BARC-055551–13,421 and BARC-048275–10,534	(1,308,798–1,705,500)	Partial Greenhouse assay	F8 (247)	7.3%	PI 438489B	Usovsky et al. (2021)
		–	BARC-012237–01,756	(1,685,571)	Partial Greenhouse assay	F6:9 (247)	7.5%	PI 567516C	Jiao et al. (2015)
	–	–	Sat_168	3.9 cM* (1,706,200)	Partial Greenhouse assay	F6 derived RILs (228)	8%	PI 437654	Ha et al. (2007)
	–	–	Satt309	4.53 cM* (1,736,692)	Partial Greenhouse assay	F5:16 (92)	13.2%	Hartwig (PI 437654)	Lee et al. (2016)
MLG L (Chr. 19)	–	–	Satt513	106.37 cM* (49,223,526)	Partial Greenhouse assay	F6 derived RILs (228)	21%	PI 437654	Ha et al. (2007)

*GmComposite2003 genetic position (www.soybase.org)

^a^Locus name given in this study, if the physical positions of QTLs overlap each other in at least two independent studies. For example, *qRrr11-01* means the 1st (01) validated quantitative (q) resistance (R) to *Rotylenchulus reniformis (rr)* on Chr. 11 (11)

^b^Marker position (bp) based on the *Glycine max* genome assembly version Glyma.Wm82.a2 (Gmax2.0), only starting position is shown for SSR markers

^c^Phenotypic variations explained by the molecular markers

Lance nematodes (*Hoplolaimus* spp.) (LN) are migratory ecto-endo plant–parasitic nematodes that are widespread throughout the USA (Sher 1963; Astudillo and Birchfield 1980; Yan et al. 2016). A total of seven species have been identified and reported in the southeastern USA, including *Hoplolaimus galeatus* Thorne, 1935; *H. columbus* Sher, 1963; *H. magnistylus* Robbins, 1982; *H. stephanus* Sher, 1963; *H. seinhorsti* Luc, 1958; *H. tylenchiformis* von Daday, 1905; and *H. concaudajuvencus* Golden and Minton, 1970 (Lewis and Fassuliotis 1982; Robbins 1982; Koenning et al. 1999). However, only three species (*H. columbus*, *H. galeatus*, and *H. magnistylus*) have been considered economically important lance nematodes in soybean production in the USA (Holguin et al. 2016). The outbreak of *H. columbus* was first detected in South Carolina and predominantly prevailed in South Carolina, North Carolina, and Georgia while *H. galeatus* and *H. magnistylus* were commonly reported in soybean production areas in Alabama, Arkansas, Mississippi, and Tennessee (Lewis and Fassuliotis 1982; Robbins 1982; Koenning et al. 1999). These nematodes primarily damage the structures of the epidermis and cortex in the root (Lewis and Fassuliotis 1982; Lewis, 1989) and cause root stunting/shedding, foliar chlorosis, as well as severely limiting lateral root growth under heavy infestations (Kinloch 1998; Timper 2009). Soybean yield losses from the infestation of these LN species can be as high as 70% (Mueller and Sanders 1987; Noe 1993). Although the resistance of host plants is the most effective way to control plant–parasitic nematodes, efforts to identify genetic resistance for LN have been limited. Therefore, the application of field sanitation and crop rotation with non-host crops is helpful to control LN populations and reduce LN damage in soybean production areas.

## Section II. Soybean resistance to oomycete diseases

Crop germination and stand are key factors for a successful cropping season for soybean growers. During seed establishment, seedlings are subject to attack by several soilborne pathogens, resulting in lack of germination, damping-off or plant death. Poor plant stands due to diseases result in replanting and increased costs. Among the soilborne pathogens impacting soybean are the oomycetes, which include *Phytophthora*, *Pythium,* and *Phytopythium.* The impact of these soilborne diseases is not only limited to the beginning of the season, as root infections can occur at later stages, often reducing yield without significant above ground symptoms. In 2005, losses to soybean seedling diseases in the USA were estimated at 0.89 million Mt (Wrather and Koenning 2009). From 2006 to 2009, soybean yield losses due to seedling diseases have increased considerably ranking second only to soybean cyst nematode (Koenning and Wrather 2010). There are also oomycete diseases that occur in the canopy, like downy mildew caused by [*Peronospora manshurica* (Naum.) Sdy.], which under conducive conditions could affect seed quality and yield (Dunleavy 1987). Key species have been recognized as major contributors in disease development and most breeding efforts have focused on minimizing impacts by *Phytophthora* and *Pythium* (Dorrance et al. 2009; Rupe et al. 2011). Recent efforts have expanded the knowledge of oomycete species causing disease on soybean, but the range of this potential species varies with the locations (Rojas et al. 2017), and among those, some species are considered emerging such as *Phytophthora sansomeana* E.M. Hansen & Reeser (McCoy et al. 2018).

## Phytophthora root and stem rot

Phytophthora root and stem rot (PRSR) of soybean is one of the most prevalent and widely distributed soybean diseases, causing reduced yield and worldwide losses of 2.3 million Mt per year (Erwin and Ribeiro 1996; Koenning and Wrather 2010; Allen et al. 2017). *Phytophthora sojae* Kaufmann & Gerdemann, the main causal agent of this disease, was initially reported in the mid-1950s in the Midwest region of the USA (Kaufmann and Gerdemann 1958) and has since become a major concern for soybean production causing annual losses of approximately 1.2 million Mt in the USA (Wrather et al. 2010). *P. sojae* is an oomycete pathogen that survives in the soil as oospores. Under optimal conditions, oospores germinate and infect seeds and roots causing seed rot and damping-off of seedlings. *P. sojae* may also cause root and stem rot that results in wilting and plant death. While the typical brown to purple water-soaked lesions on the stem appear mid-late season on infected plants, early-season infection may also result in an uneven plant stand and possibly need of replanting (Bienapfl et al. 2011; Dorrance et al. 2016).

Screening of *P. sojae* for race identification and soybean line resistance has been based on the use of hypocotyl inoculations (Dorrance et al. 2008; Stewart and Robertson 2012; Lin et al. 2014). For *P. sojae*, *Rps 1a*, *1b*, *1c*, *1 k*, *3a*, *3b*, *3c*, *4*, *6*, *7*, or *8* are part of the set of differentials, and recent surveys have tested isolates identifying emerging races. Of those, *Rps1a-1 k*, *Rps3a*, *Rps6* and *Rps8* are deployed through resistant cultivars. However, there are reports of resistance breakdown of *Rps1* in soybean-producing states in the Midwest of USA (Dorrance et al. 2016; Matthiesen et al. 2021; McCoy et al. 2021). In lower frequency, *Rps3a* and *Rps6* were also defeated by some isolates in the Midwest. Since not all identified resistance genes have been deployed, it is important to monitor races for future breeding efforts as some of the remaining resistance genes have also been overcome by a few field isolates (Dorrance et al. 2016; McCoy et al. 2021).

Fortunately, novel *Rps* genes or alleles have been identified conferring broad-spectrum resistance to *P. sojae* races. To date, more than 40 *Rps* genes or alleles have been reported worldwide (Table 5). Intriguingly, the *Rps* genes/alleles were not evenly distributed but were clustered on some specific chromosomes. For instance, more than half of the *Rps* genes/alleles (*Rps1a-1 k*, *Rps7*, *Rps9*, *RpsUN1*, *RpsYD25*, *RpsYD29*, *RpsHN*, *RpsQ*, *RpsWA*, *RpsWY*, *RpsHC18*, *RpsX*, *RpsGZ*, *RpsDA*, *RpsT1*, *RpsT2*, *RpsT3*, and *Rps14*) were mapped in a nucleotide-binding site-leucine-rich repeat (NBS-LRR) gene enriched region on Chr. 3; Six *Rps* genes/alleles were located on chromosomes 13 (*Rps3a*, *Rps3b*, *Rps3c*, *Rps8*, *RpsSN10* and *RpsCD*) and 18 (*Rps4*, *Rps5*, *Rps6*, *RpsJS*, *Rps12*, and *Rps13*), respectively. The rest of *Rps* genes were located at chromosomes 2 (*RpsZS18*), 7 (*Rps11*), 10 (*RpsSu*), 16 (*Rps2* and *RpsUN2*), 17 (*Rps10*), and 19 (*RpsYB30*) (Table 5).

**Table 5:** Soybean resistance genes/alleles (*Rps*) and validated QTL/loci conferring resistance to Phytophthora root and stem rot (caused by *P. sojae* and other *Phytophthora* spp.)

Causal agent	MLG (Chr.)	Locus/allele name	Other name	Tightly linked/flanking markers	Marker position cM (bp)^a^	Testing methods/Resistance spectrum	Population type (size)	PVE^b^	Candidate genes	Donor source/allele	References
*P. sojae*	MLG D1b (Chr. 2)	*RpsZS18*	–	Indelwz1, Indelwz2, Indelwz3, Indelwz4, and Indelwz5	(43,411,141,/43,423,820/43,425,227/43,429,008/43,434,948 a2)	Hypocotyl inoculation/*PsRace1, PsRace3, PsRace4, PsRace5, PsUSAR2, Ps41-1, PsNKI, PsFJ2, PsFJ3, Ps6497*	F2:3 (232)	R gene	Glyma.02g245700, Glyma.02g245800, and Glyma.02g246300	Zaoshu18	Yao et al. (2010), Zhong et al. (2018b)
	MLG N (Chr. 3)	*Rps1a*	–	Satt159 (BARCSOYSSR_03_0180) and Satt009 (BARCSOYSSR_03_0226)	(3,197,998—3,932,012 a2)	Hypocotyl inoculation/Races *1, 2, 11, 13, 14, 15, 16, 17, 18, 26, 27, 31, 32, 36, 48, 50, 51, 52, 54, 55,* and isolates *PsHJL1, PsAH4, PsFJ, PsAH1, PsAM1, PsTA3, PsJS9, PsJS8, Ps52, PsJN4, pmg(1)-3, pmg(10)-1, pmg(13)-1, pmg(17)-1, OHSS03WayneBerry5, OHSS04WayneTBH62xx, OH8only,*	F2:3 (81), F2:3 (84)	R gene	–	Mukden, Harlon, Harsoy12xx, L59-731, Union, L88-8470	Bernard et al. (1957), Diers et al. (1992), Weng et al. (2001), Dorrance et al. (2004), Sugimoto et al. (2012), Gunadi (2012), Zhang et al. (2013a), Lin et al. (2013), Jang and Lee (2020)
		*Rps1b*	–	Satt152 (BARCSOYSSR_03_0192) and Satt530	(3,366,546—5,669,644 a2)	Hypocotyl inoculation/Races *1, 3, 4, 5, 6, 7,8, 9, 11, 13, 14, 15, 18, 21, 22, 24, 26, 27, 34, 36, 37, 40, 42, 43, 44, 46, 48, 49, 50, 51, 52, 54, 55,* and isolates *PsJL1-1, PsJMS3, PsHJL1, PsAH4, PsFJ, PsAH3, PsXJ, PsJS9, Ps41-1, PsJS7, PsJS8, PsMC1, PsHY33-1, pmg(1)-3, 94–14-432(2), 94-13p-197, pmg(5)-3, 95–11-117(4), pmg(8)-3, pmg(13)-1, ISA 71D-1, OH199915.5.2.1, OH2000Wood25, OH2000Sandusky74, OHSS03HenryCo3, OHSS04WayneTBH62xx, PS03-113*	F2:3 (160–274), F2:3 (113)	R gene	–	PI 172901, Haro 13xx, L77-1863	Mueller et al. (1978), Ploper et al. (1985), Demirbas et al (2001), Dorrance et al (2004), Sugimoto et al (2012), Gunadi (2012), Zhang et al (2013a), Lin et al (2013), Jang and Lee (2020)
		*Rps1c*	–	Satt152 (BARCSOYSSR_03_0192) and Satt584 (BARCSOYSSR_03_0442)	(3,366,546—9,228,056 a2)	Hypocotyl inoculation / Races *1, 2, 3, 6, 7, 8, 9, 11, 13, 15, 17, 21, 23, 24, 26, 28, 29, 30, 32, 34, 36, 41, 42, 44, 48, 50, 52, 54, 55,* and isolates *PsJMS3, PsHJL1, PsAH4, PsFJ, PsAH3, PsXJ, PsBr1, PsTA3, PsJS9, Ps41-1, PsJS7, PsJS8, Ps52, Ps53, PsAH5, PsHY33-1, pmg(1)-3, 94–14-432(2), 95–11-117(4), pmg(8)-3, pmg(10)-1, pmg(13)-1, pmg(17)-1, 96-13S-106A.1, ISA 19A-1, ISA 19B-2, ButMu4S2, M1-1–1, M1-1–3, M3-3–1, M6-3–1, OH199915.5.2.1, OHSS03HenryCo3, OHSS04WayneTBH62xx, PS04-257, PS04-323*	F2:3 (95)	R gene	–	L75-3735, Williams79, L77-1727, L85-129	Mueller et al. (1978), Demirbas et al. (2001), Dorrance et al. (2004), Sugimoto et al. (2012), Gunadi (2012), Zhang et al. (2013a), Lin et al. (2013), Jang and Lee (2020)
		*Rps1d*	–	Satt152 (BARCSOYSSR_03_0192) and Sat_186 (BARCSOYSSR_03_0204)	(3,366,546 a2 and 3,465,436 a1)	Hypocotyl inoculation/Races *1, 2, 3, 4, 5, 7, 9, 11, 13, 14, 15, 16, 18, 20, 21, 22, 23, 24, 25, 27, 28, 29, 30, 32, 34, 36, 37, 39, 45, 46, 47, 48, 49, 50, 51, 52, 53, 55,* and isolates *PsJL1-1, PsJMS3, PsHJL1, PsAH4, PsJL4-1, PsJL3-2, PsFJ, PsAH3, PsAH1, PsBr1, PsTA3, PsMC1, PsHY33-1, pmg(1)-3, 94–14-432(2), pmg(5)-3, 95–11-117(4), pmg(10)-1, pmg(13)-1, 95–15-15, pmg(25)-1, 96-13S-106A.1, ISA 19A-1, ISA 19B-2, ISA 71D-1, M6-3–1, OH2000Sandusky74, OHSS03HenryCo3, OHSS03WayneBerry11, OHSS03WayneBerry5, OHSS04WayneTBH62xx, OH8only, PS03-113, PS04-323*	F2:3 (47)	R gene	–	Haro 16, PI 103091, L99-3312	Buzzell and Andrson (1992), Demirbas et al. (2001), Dorrance et al. (2004), Sugimoto et al. (2007), Sugimoto et al. (2012), Gunadi (2012), Zhang et al. (2013a), Lin et al. (2013), Jang and Lee (2020)
		*Rps1k*	–	CG1 and TC1	–	Hypocotyl inoculation/Races *1, 2, 3, 4, 5, 6, 7, 8, 9, 11, 13, 14, 15, 17, 18, 20, 21, 22, 23, 24, 26, 36, 37, 42, 43, 44, 46, 47, 48, 49, 50, 51, 52, 53, 54, 55,* and isolates *PsJL1-1, PsJMS3, PsHJL1, PsAH4, PsFJ, PsXJ, PsAH1, PsBr1, PsTA3, PsJS9, Ps41-1, PsJS7, PsJL5, PsJS8, Ps52, PsJN4, PsAH5, PsHY33-1, pmg(1)-3, 94–14-432(2), 94-13p-197, pmg(5)-3, 95–11-117(4), pmg(8)-3, pmg(10)-1, pmg(13)-1, pmg(17)-1, ISA 71–1, M1-1–1, M1-1–3, M3-3–1, M6-3–1, OH199915.5.2.1, OH2000Sandusky74, OH2000Wood25, OHSS03HenryCo3, OHSS04WayneTBH62xx, PS03-113*	F2:3 (111)	R gene	AY963292 and AY963293	Williams82, L77-1794	Bernard and Cremeens (1981), Kasuga et al. (1997), Demirbas et al. (2001), Dorrance et al. (2004), Gao et al. (2005), Bhattacharyya et al. (2005), Gao and Bhattacharyya (2008), Sugimoto et al. (2012), Gunadi (2012), Zhang et al. (2013a), Lin et al. (2013)
		*Rps7*	–	Satt009 (BARCSOYSSR_03_0226) and Satt125 (BARCSOYSSR_03_0564)	(3,932,012—18,415,620 a2)	Hypocotyl inoculation/Races *10, 11, 12, 16, 18, 19, 35, 36,* and isolates *PsJL1-1, PsJM3, PsHJL1, PsAH4, PsJL4-1, PsAH1, PsBr1, PsJS9, PsJS7, M1-1–3, M3-3–1, M6-3–1*	F2:3 (81)	R gene	–	OX281, Harosoy, L93-3258	Anderson and Buzzell (1992), Lohnes and Schmitthenner (1997), Weng et al. (2001), Dorrance et al. (2004), Sugimoto et al. (2012), Gunadi (2012), Zhang et al. (2013a), Jang and Lee (2020)
		*Rps9*	–	Satt631 (BARCSOYSSR_03_0162) and Sat_186 (BARCSOYSSR_03_0204)	(2,943,932 a2)	Hypocotyl inoculation/isolates *PNJ1, PNJ4, Pm2, Pm28, Pmg*	F1 (38, 35) and F2:3 (199, 259),	R gene	–	Ludou4	Wu et al. (2011a)
		*RpsUN1*	–	(BARCSOYSSR_03_0233 and BARCSOYSSR_03_0246)	(4,020,587—4,171,359 a2)	Hypocotyl inoculation/races *1, 3, 5, 7, 8, 10, 13, 17, 24, 28,* and isolates *ISA 19A-1* and *ISA 19B-2*	F2:3 (826)	R gene	Glyma.03g034600	PI 567139B	Lin et al. (2013), Li et al. (2016a, b, c)
		*RpsYu25*/*RpsYD25*	–	SSRYZ35, SSRYZ37, SSRYZ40, SSRYZ42, and BARCSOYSSR_03_0247	(4,150,768/4,158,266/4,161,570/4,204,277/4,205,985 a2)	Hypocotyl inoculation/isolates *PsMC1, Pm2, PNJ1, PNJ3, PNJ4, 6497, 7063, Pm28, Pm14, Pm31, H15, AH, PsJL1-1, PsJMS3, PsAH4, PsJL4-1, PsJL3-2, PsSX1, PsAH3, PsXJ, PsAH1, PsTA3, PsJS9, Ps41-1, PsJS7, PsJL5, PsJS8, PsJN4*	F2:3 (1127)	R gene	Glyma.03g034700, Glyma.03g034800 and Glyma.03g034900	Yudou 25	Fan et al. (2009), Sun et al. (2011), Zhang et al. (2013a), Zhong et al. (2020)
		*RpsYD29*	–	SattWM82-50 and Satt1k4b	(3,857,715 and 4,062,474 a1)	Hypocotyl inoculation / isolates *PsHLJ5, PsHLJ3, PsJMS3, PsJL1-1, PsHLJ1, PsHLJ4, PsAH4, PsJL4-3, PsGZ2, PsJL3-2, PsSX1, PsJS4, PsJL4-1, Ps41-1, PsAH3, PsAH1, PsJS9, PsJS7, PsJS8*, *PsMC1*, *PsJN4*	F2:3 (214)	R gene	Glyma03g04030 and Glyma03g04080	Yudou 29	Zhang et al. (2013a)
		*RpsHN*	–	SSRSOYN-25 and SSRSOYN-44	(4,227,863–4,506,526 a1)	Hypocotyl inoculation/pathotype *HeN08-35*	F6:8 (103), F6:8 (130), F2:3 (159)	R gene	Glyma.03g04260, Glyma.03g04300, and Glyma.03g04340	Meng8206	Niu et al. (2017)
		*RpsQ*	–	Insert11, Insert144, SNP276	(2,997,143/2,997,106/3,031,924 a1)	Hypocotyl inoculation/isolates *PsJL1-2, PsGZ-2, PsFJ, PsJS7, Ps13-2, PsUSAR2, Ps13-1, PsAH, Ps13-4, PsAH4, PsHLJ5, Ps41-1, PsGS8, PsJS12, PsJMS2, PsAH1, PsSX1, PsAH5, Ps13-14, Ps13-5, PsJA08-1, Ps13-3, PsJA08-3, PsMC1, PsAH3, PsAH6, PsJL5, Ps13-12, PsJS6, PsJS10*	F2 (207), F2:3 (207)	R gene	Glyma.03g27200	Qichadou1	Li et al. (2017c)
		*RpsWA*^$^	–	Satt009 (BARCSOYSSR_03_0226) and T0003044871	0.9 -1.6 cM (3,919,203—4,486,048 a1)	Hypocotyl inoculation / isolates *PJ-H42, PJ-H67, R1, R4, R17, R25*	F2 (98), F7:8 (94)	R gene	–	Waseshiroge	Sugimoto et al. (2011)
		*RpsWY*	–	Satt631—Satt152, bin401	(4,466,230—4,502,773 a2)	Hypocotyl inoculation / isolates *Pm14, Pm28, PNJ1, P6497*	F2 (191), F7:8 (196)	R gene	–	Wayao	Cheng et al. (2017b)
		*RpsHC18*	–	BARCSOYSSR_03_0267 and BARCSOYSSR_03_0269	(4,152,795–4,919,481 a1)	Hypocotyl inoculation/*PsRace1, PsRace3, PsRace4, PsRace5, PsUSAR2, Ps41-1, PsMC1, PsAH4, PsNKI, PsFJ2, PsFJ3, PsJS2*	F2:3 (177)	R gene	Glyma03G04560 and Glyma03G04590	Huachuan 18	Zhong et al. (2018a)
		*RpsX (RpsQ?)*	–	BARCSOYSSR_03_0161, BARCSOYSSR_03_0165, BARCSOYSSR_03_0167, and Insert144	(2,910,913–3,153,254, 2,997,106 a2)	Hypocotyl inoculation/*PsRace1, PsRace3, PsRace4, PsRace5, PsUSAR2, Ps41-1, PsAH4, PsMC1, PsNKI, PsFJ2, PsFJ3, PsJS2, Ps6497, Ps7063*	F2:3 (137)	R gene	Glyma.03g027200	Xiu94-11	Zhong et al. (2019)
		*RpsGZ*	–	–	(4,003,401 and 4,370,772 a2)	Hypocotyl inoculation / *PNJ4, PNJ1*	F8:11 (228)	R gene	Glyma.03 g05300	Guizao1	Jiang et al. (2020)
		*RpsDA* ^$^	–	–	(3,893,390–4,752,969 a2)	Hypocotyl inoculation / isolate *2457*	F5 derived (59)	66.4%	–	Daewon	Jang et al. (2020)
		*RpsT1, RpsT2, RpsT3*	–	BARCSOYSSR_03_0209 and BARCSOYSSR_03_0385	(3,606,810–7,614,961 a2)	Hypocotyl inoculation / isolate *Ps060626-4–1, Ps060726-2–1, Pm6, Ps74, Ps060619-1–2, Ps060619-1–1, Ps060619-1–3, Ps080626-1–7, Ps070702-4–1, Ps060710-5–1, Ps060629-4–1, Ps060626-71–1, Ps060828-1–1, Ps060626-6–1, Ps080623-1–4, Ps070702-1–1, S5 Ps070726-3–4, Ps060710-3–1, Ps070621-6–1*	F2:3 (105), F4:5 (165)	R gene	–	Tosan231	Matsuoka et al. (2021)
		*Rps14*	–	InDel4033 and InDel4263	(4,033,638—4,263,083 a2)	Hypocotyl inoculation / *Race 1, 2, 3, 4, 6, 7, 8, 9, 17, 25, ISA19A-1, ISA71D-1, MIN12001.01.05, MIN12004.01.01, MIN12004.03.01*, and *MIN12005.07.02*	F2 (167), F2:3 (110)	R gene	–	PI 340029	Chen et al. (2021a, b)
	MLG M (Chr. 7)	*Rps11*	–	BARCSOYSSR_07_0295	(5,523,128 a2)	Hypocotyl inoculation/Races *1, 3, 7, 10, 13, 17, 25, 28*, and isolates *ISA 19A-1, ISA71D-1, ISA 33O-8,* and 127 additional isolates (see Wang et al. 2021)	F2 (58), F2:3 (209), F4 (17,050)	R gene	a 27.7 kb NBS-LRR gene	PI 594527	Ping et al. (2016), Wang et al. (2021)
	MLG O (Chr. 10)	*RpsSu*	–	Satt358—Sat_242 (BARCSOYSSR_10_1104)	3.5–7.4 cM (1,018,481—39,392,879 a2)	Hypocotyl inoculation / isolate *Pm14*	RIL (176)	R gene	–	Su88-M21	Wu et al. (2011b)
	MLG F (Chr. 13)	*Rps3a*	–	Satt510 (BARCSOYSSR_13_1219) and Satt335 (BARCSOYSSR_13_1271)	(31,802,616—32,721,481 a2)	Hypocotyl inoculation/races *1, 2, 3, 4, 5, 8, 9, 11, 13, 14, 16, 18, 23, 25, 27, 28, 29, 31, 32, 33, 34, 35, 40, 41, 43, 44, 45, 47, 48, 49, 50, 51, 52, 54* and isolates *PsJL1-1, PsJMS3, PsHJL1, PsJL4-1, PsJL3-2, PsFJ, PsSX1, PsXJ, PsBr1, PsAM1, Ps41-1, PsJL5, Ps52, Ps53, PsMC1, PsJN4, 2010DPH39-02–6, OH2000Wood25, OHSS03HenryCo3, OHSS03WayneBerry11, OHSS03WayneBerry5, OH25 2011, PS03-036, PS03-113, PS04-139, PS04-257, PS04-323, PTO4C2Res11*	F2:3 (89)	R gene	–	PI 171442, L83-570	Mueller et al. (1978), Dorrance et al. (2004), Gordon et al. (2007), Sugimoto et al. (2012), Gunadi (2012), Zhang et al. (2013a)
		*Rps3b*	–	–	–	Hypocotyl inoculation / races *1, 2, 3, 4, 5, 7, 9* and isolates *PsJL1-1, PsJMS3, PsHJL1, PsAH4, PsJL4-1, PsJL3-2, PsFJ, PsSX1, PsAH3, PsAH1, PsBr1, PsAM1, PsTA3, Ps52, PsAH5, 2010DPH39-02–1, OHSS03HenryCo3, OHSS03HenryCo7, PS03-06, PS03-113*	F2:3 (160–274)	R gene	–	PI 172901, PRX146-36, L91-8347, L89-1541	Ploper et al. (1985), Dorrance et al. (2004), Gunadi (2012), Zhang et al. (2013a)
		*Rps3c*	–	–	–	Hypocotyl inoculation / races *1, 2, 3, 4,* and isolates *PsJL1-1, PsJMS3, PsAH4, PsJL4-1, PsJL3-2, PsFJ, PsSX1, PsAH3, PsAH1, PsBr1, PsAM1, 2010DP39-02–1, 2010DPH39-02–1, 2010W137-135–1, ButMu4S2, M1-1–2, OH2000Wood25, OHSS03WayneBerry5, OH25 2011, PS03-036, PS03-113, PS04-132, PS04-139, PS04-257, PS04-323, PTO4C2Res11*	F2 (1650, 1708, 1182, 1517, 1692, 1208, 1687, 1452)	R gene	–	PI 340046, PRX145-48, L92-7857	Athow et al. (1986), Dorrance et al. (2004), Sugimoto et al. (2012), Gunadi (2012)
		*Rps8*	–	Satt425 (BARCSOYSSR_13_0784) and Satt114 (BARCSOYSSR_13_1055), Sat_154 and Sat_120	(24,361,239—28,912,878 a2)	Hypocotyl inoculation/race *1, 25*, isolates *PsJL1-1, PsJMS3, PsHJL1, PsAH4, PsJL4-1, PsJL3-2, PsFJ, PsSX1, PsAH3, PsAM1, PsJS7, Ps53, 2010DPH39-02–1, OH2000Sandusky74, OHSS03HenryCo3, OHSS03HenryCo7, OHSS03WayneBerry11, OHSS03WayneBerry5, OHSS04WayneTBH62xx, OH25 2011, PS03-036, PS03-113, PS04-132, PS04-139, PS04-257, PS04-323, PTO4C2Res11*	F2:3 (208, 202)/F2:3 (138)	R gene	–	PI 399073	Burnham et al. (2003a), Sandhu et al. (2005), Gordon et al. (2006), Gunadi (2012), Zhang et al. (2013a), Jang et al. (2020)
		*RpsSN10*	–	Satt423 (BARCSOYSSR_13_0264) and Satt149 (BARCSOYSSR_13_0245)	(16,600,532—16,855,132 a2)	Hypocotyl inoculation/race *1*	F2 (124)	R gene	–	Suinong 10	Yu et al. (2010)
		*RpsCD*	–	–	–	Detached trifoliate leaf disease screening assay/effector *Avh180*, and isolates *Iso1005 and Iso2004*	F2:3 and RIL (142)	R gene	–	PI 408132	Davis (2017)
	MLG J (Chr. 16)	*Rps2*	–	Satt547 (BARCSOYSSR_16_1165)	(34,035,215 a2)	Hypocotyl inoculation/isolates *PsJL1-1, PsHJL1, PsJL4-1, PsJL3-2, PsFJ, PsSX1, PsAH3, PsBr1, PsTA3, PsJS9, PsJS7, PsJS8, Ps52, pmg(1)-3, 94–14-432(2), pmg(25)-1, 96-13S-106A.1, ISA 19A-1, ISA 19B-2, ISA 71D-1*	F2:3 (115)	R gene	–	CNS', L82-1449, L76-1988	Kilen et al. (1974), Polzin et al. (1994), Demirbas et al. (2001), Dorrance et al. (2004), Zhang et al. (2013a), Lin et al. (2013), Jang et al. (2020)
		*RpsUN2*	–	CAPS6 and SNP2	(37,239,148—37,275,206 a2)	Hypocotyl inoculation / races *1,3,4,5,10,13,24,25, 28*, and isolates *ISA 19A-1, ISA 19B-2, ISA 71D-1, ISA 33O-8*	F2:3 (826)	R gene	Glyma.16g215200 and Glyma.16g214900	PI 567139B	Lin et al. (2013), Li et al. (2016a, b, c)
	MLG D2 (Chr. 17)	*Rps10*	–	Sattwd15-28, Sattwd15-32	(30,964,476/30,983,669 a1)	Hypocotyl inoculation/isolate *PsMC1*	F2:3 (102)	R gene	Glyma17g28950 and Glyma17g28970	Wandou15	Zhang et al. (2013b)
	MLG G (Chr. 18)	*Rps4*	–	Satt191 (BARCSOYSSR_18_1750) Sat_064 (BARCSOYSSR_18_1858)	(56,333,740 a2)	Hypocotyl inoculation / isolates *PsJL1-1, PsJMS3, PsHJL1, PsJL4-1, PsJL3-2, PsSX1, PsXJ, PsAM1, Ps41-1, PsJL5, Ps53, ButMu4S2, OH2000Wood25, OHSS03WayneBerry11, OHSS03WayneBerry5, OH25 2011, PS02-014, PS03-036, PS03-113, PS04-132, PS04-139, PS04-257, PS04-323, PTO4C2Res11*	F2:3 (100)	R gene	–	L85-2352	Athow et al. (1980), Demirbas et al. (2001), Dorrance et al. (2004), Sandhu et al. (2004), Gunadi (2012), Zhang et al. (2013a)
		*Rps5*	–	Satt472 (BARCSOYSSR_18_1708)	(53,866,606 a2)	Hypocotyl inoculation/isolates *PsJL1-1, PsJMS3, PsHJL1, PsJL4-1, PsJL3-2, PsSX1, PsXJ, PsAM1, PsJL5, OH2000Wood25, OHSS03WayneBerry5, OHSS04WayneTBH62xx, PS03-036, PS03-113, PS04-139, PS04-323, PTO4C2Res11*	F2:3 (122)	R gene	–	L62-904, L85-3059	Buzzell and Anderson (1981), Demirbas et al. (2001), Dorrance et al. (2004), Gunadi (2012), Zhang et al. (2013a), Sahoo et al. (2017)
		*Rps6*	–	Satt191 (BARCSOYSSR_18_1750) and Sat_372	(54,450,956 a2)	Hypocotyl inoculation/races *1, 2, 3, 4, 10, 12, 14, 15, 16, 18, 19, 20, 21, 25, 28, 33, 34, 35, 40, 41, 42, 43, 44, 46, 47, 48, 53, 54* and isolates *PsJL1-1, PsJMS3, PsHJL1, PsAH4, PsJL4-1, PsJL3-2, PsSX1, PsXJ, Ps41-1, PsJL5, Ps53, 2010DP39-02–6, 2010W137-135–1, ButMu4S2, M1-1–3, M3-3–1, M6-3–1, OH2000Wood25, OHSS03WayneBerry11, OHSS03WayneBerry5, OH25 2011, PS02-014, PS03-036, PS03-113, PS04-139, PS04-257, PS04-323, PTO4C2Res11*	F2:3 (89)	R gene	–	Haro 62xx, L89-1581	Athow and Laviolette (1982), Demirbas et al. (2001), Dorrance et al. (2004), Gordon et al. (2007), Sugimoto et al. (2012), Gunadi 2012; Zhang et al. (2013a)
		*RpsJS*	–	BARCSOYSSR_18_1861, SSRG60684K, and SSRG60718K	(60,613,262—60,752,225 a1)	Hypocotyl inoculation / isolates *JS08-12, P6497, P7063, S16, S2, PNJ1, Pmg, Pm28, Pm2, HeN08-35, HLJ08-17, H15, AH, P7071, Pm31*	F2:3 (231)	R gene	–	Nannong10-1	Sun et al. (2014a)
		*Rps12*	–	BARCSOYSSR_18_1840 and Sat_064, 4 cM from *Rps13*	(55,962,037—56,333,703 a2)	Hypocotyl inoculation/isolates *R17, Val12-11, P7074, 1005–2.9, III5.2b, IV5.2, IV10, IV12.2a, IV13.4a, IV23.3, VI5.2b, VI12.1a, VI15, VI17, VI23.3b, S5-5, PR1, PR6, 1005–2.9* + *VI23.3b* + *R17*	F7 derived (290)	R gene	–	PI 399036	Sahoo et al. (2017), Sahoo et al. (2021)
		*Rps13*	–	Sat_064, BARCSOYSSR_18_1859 and BARCSOYSSR_18_1860	(56,333,703—56,341,167 a2)	Hypocotyl inoculation / isolate *V13, R17* + *Val12-11*	F8 derived (120)	R gene	–	PI 399036	Sahoo et al. (2021)
	MLG L (Chr. 19)	*RpsYB30*	–	Satt497 (BARCSOYSSR_19_0760) Satt313 (BARCSOYSSR_19_0788)	(33,865,280—34,753,167 a2)	Hypocotyl inoculation/isolates *USAR1, PsBX1, PsXJ1, PsMC1, PsZLT1, PsFJ1, PsXJ2, Ps41-1*	F4 (57)	R gene	–	Youbian30	Zhu et al. (2007)
	MLG D1a (Chr. 1)	*qRps01-01*^$^	–	BARC_2.0_Gm01_50164447 and BARC_2.0_Gm01_50295635	(50,164,447—50,295,635 a2)	Tray test / isolates *1.S.1.1*	F9:11 (316)	4.5%	–	Conrad	Stasko et al. (2016)
			–	BARC_2.0_Gm01_50206347 and BARC_2.0_Gm01_50287274	(50,206,347—50,287,274 a2)	Tray test / isolates *OH25*	F9:11 (316)	8.2%	–	Conrad	Stasko et al. (2016)
			–	BARC_2.0_Gm01_50572171 and BARC_2.0_Gm01_50797061	(50,572,171 -50,797,061 a2)	Tray test / isolates *PT2004C2.S1*	F9:11 (316)	7.6%	–	Conrad	Stasko et al. (2016)
	MLG D1b (Chr. 2)	*qRps02-01*^$^	–	Satt579 (BARCSOYSSR_02_0855) and Satt600 (BARCSOYSSR_02_1048)	(19,688,108—29,355,267 a2)	Slant board test / isolate OH25	F4:6 (66), F4:6 (79)	10.6–20.7%	–	–	Burnham et al. (2003b)
			*QPRR-3*	Satt579 (BARCSOYSSR_02_0855) and Sat_089 (BARCSOYSSR_02_1152)	(19,688,108—34,875,449 a2)	Field inoculation and greenhouse test (China and Canada)	F2:6 (140)	5.5–28.0%	–	–	Li et al. (2010d)
			*QPRR-2*	Satt005 (BARCSOYSSR_02_0998) and Satt600 (BARCSOYSSR_02_1048)	(27,699,285—29,355,267 a2)	Field inoculation and greenhouse test (China and Canada)	F2:6 (140)	11.3–22.0%	–	–	Li et al. (2010d)
	MLG N (Chr. 3)	*qRps03-01*^$^	*qHO3-1*	ss715585371	(3,607,392 a2)	Hypocotyl inoculation / isolate *OH.12108.6.3 (OH.121)*	Germplasm (429)	–	–	–	Van et al. (2020)
			*C2-03–3*	ss715585633	(3,691,222–3,895,958 a2)	Layer test/isolate *C2.S1*	Germplasm (495)	4.0% IRRS^c^	–	–	Rolling et al. (2020)
			*QTL 3–1*	ss715585712 and ss715585728	(3,852,888 and 3,865,730 a2)	Tray test / isolates *OH121* and *C2S1*	PI lines (800)	3.2%Root rot score	–	–	Schneider et al. (2016)
		*qRps03-02*^$^	*Qprr3-2*	Chr03-3,904,775 and Chr03-4,404,630	(3,904,775 and 4,404,630 a2)	Hypocotyl inoculation / race *1*	F5:15 (109)	56.1%	Glyma.03G033800 and Glyma.03G033700	DongongL-28	Zhao et al. (2020)
			*Rprr-3–1*	–	(3,993,736 a2)	Hypocotyl inoculation / race *1*	Germplasm and cultivars (225)	33.6%	–	G	Zhao et al. (2020)
			*QTL 3–2*	ss715586320 and ss715586321	(4,276,534 and 4,277,380 a2)	Tray test / isolates *OH121* and *C2S1*	PI lines (800)	3.1% Root rot score	–	–	Schneider et al. (2016)
			*qHO3-2*	ss715586333	(4,289,618 a2)	Hypocotyl inoculation/isolate *OH.12108.6.3 (OH.121)*	Germplasm (429)	–	–	–	Van et al. (2020)
			*QTL 3–3*	ss715586376	(4,315,512 a2)	Tray test / isolates *OH121* and *C2S1*	PI lines (800)	3.9% Root rot score	–	–	Schneider et al. (2016)
	MLG C1 (Chr. 4)	*qRps04-01*^$^		BARC_2.0_Gm04_45977762 and BARC_2.0_Gm04_46204517	(45,977,762—46,204,517 a2)	Tray test / isolates *1.S.1.1*	F9:11 (316)	3.2%	–	Sloan	Stasko et al. (2016)
				BARC_2.0_Gm04_46096228 and BARC_2.0_Gm04_46536196	(46,096,228–46,536,196 a2)	Tray test/isolates *PT2004C2.S1*	F9:11 (316)	3.2%	–	Sloan	Stasko et al. (2016)
	MLG C2 (Chr. 6)	*qRps06-01*^$^	*qPR-6–3*	Satt520 (BARCSOYSSR_06_0386) and Satt557 (BARCSOYSSR_06_1041)	(7,023,397 a1/20,218,893 a2)	Modified slant board assay/race *2*	F7:11 (176)	4.3%	–	Su88-M21	Wu et al. (2011c)
				Gm06_11776489_C_A	(11,725,151- 11,728,261 a2)	Tray test/isolate *Win371*	SoyNAM RIL (91)	17.00%	–	IA3023	Scott et al. (2019)
			*QPRR-7*	Satt277 (BARCSOYSSR_06_0920) and Satt365	(17,218,677 a2)	Field inoculation and greenhouse test (China and Canada)	F2:6 (140)	9.3–21.8%	–	–	Li et al. (2010d)
		*qRps06-02*^$^	–	BARC-062515–17,881 and BARC-040475–07,751	(21,986,774—46,596,066 a2)	Rice-based method/isolates *PT2004C2.S1, 1005–2.9, R7-2a*	F5:7 (232), F5:7 (277)	5.5% %DRL^d^, 4.7% %DSA^e^	–	AR2	Abeysekara et al. (2016)
			*QPRR-6*	Satt489 (BARCSOYSSR_06_1129) and Satt100 (BARCSOYSSR_06_1202)	(23,848,501—31,490,622 a2)	Field inoculation and greenhouse test (China and Canada)	F2:6 (140)	5.4–21.8%	–	–	Li et al. (2010d)
			*qHO6-1*	ss715594028	(26,103,041 a2)	Hypocotyl inoculation / isolate *OH.12108.6.3 (OH.121)*	Germplasm (429)	–	–	–	Van et al. (2020)
	MLG O (Chr. 10)	*qRps10-01*^$^	*qPR-10–2*	Satt420 (BARCSOYSSR_10_0507) and Sat_274 (BARCSOYSSR_10_1353)	(10,091,607—43,788,256 a2)	Modified slant board assay/race *2*	F7:11 (176)	7.7–8.3%	–	Su88-M21	Wu et al. (2011c)
			*OH-10–2*	ss715606258	(33,174,697—33,499,135 a2)	Layer test/isolate *OH.121*	Germplasm (478)	4.5% ISW^f^	–	–	Rolling et al. (2020)
			*qHC10-1*	ss715607061	(41,316,525 a2)	Hypocotyl inoculation / isolate *PT2004 C2.S1*	Germplasm (460)	–	–	–	Van et al. (2020)
	MLG H (Chr. 12)	*qRps12-01*^$^	–	BARC-019775–04,370 and BARC-025943–05,179	(7,533,328—9,804,252 a2)	Rice-based method/isolates *PT2004C2.S1, 1005–2.9, R7-2a*	F5:7 (232), F5:7 (277)	5.7% CDSW^g^	–	AR2	Abeysekara et al. (2016)
			*qHM12-1*	ss715613620	(8,854,648 a2)	Hypocotyl inoculation/isolates *PT2004 C2.S1, R7-2a, 1005–2.9*	*G. soja* (520)	–	–	–	Van et al. (2020)
	MLG F (Chr. 13)	*qRps13-01*^$^	*QGP1*	Satt509 (BARCSOYSSR_11_0342) and Satt030 (BARCSOYSSR_13_0445)	(6,216,988—13,134,055 a2)	Field inoculation and greenhouse test (China and Canada)	F2:7 (112)	6.7–13.2%	–	–	Han et al. (2008)
			*QPRR-1*	Satt325 (BARCSOYSSR_13_0639) and Satt343 (BARCSOYSSR_13_0518)	(8,587,948—10,392,903 a2)	Field inoculation and greenhouse test (China and Canada)	F2:6 (140)	9.2–10.2%	–	–	Li et al. (2010d)
			*QGP2*	Satt343 (BARCSOYSSR_13_0518) and OPG16_600_	(10,392,903 a2)	Field inoculation and greenhouse test (China and Canada)	F2:7 (112)	2.4–8.2%	–	–	Han et al. (2008)
		*qRps13-02*^$^	*OH-13–1*	ss715614516	(27,874,365—27,896,769 a2)	Layer test/isolate *OH.121*	Germplasm (478)	6.75% IRRS	–	–	Rolling et al. (2020)
			*C2-13–5*	ss715614543	(28,001,686—28,051,574 a2)	Layer test/isolate *C2.S1*	Germplasm (495)	2.07% IPH^h^	–	–	Rolling et al. (2020)
			*QTL-13*	Chr13:28,842,184 and Chr13:30,776,191	(28,842,184–30,776,191 a2)	Hydroponic assay/mixed inoculum (pathotypes 1a, 1b, 1c, 1d, 1 k, 3a, 6, and 7)	F5:6 (147)	17.6% CDW^i^	Glyma.13G190400	PI 449459	de Ronne et al. (2020)
			*QTL-13*	BARCSOYSSR_13_1103	51-52 cM (29,647,017 a2)	Tray test and layer test/isolates *C.2.S.1, OH25, OH7, 1.S.1.1, OH30*	F7:8 (305)	8.7%-16.1%	–	PI 398841	Lee et al. (2013a), Lee et al. (2014)
			*qHC13-2*	ss715614840	(29,698,315 a2)	Hypocotyl inoculation/isolate *PT2004 C2.S1*	Germplasm (460)	–	–	–	Van et al. (2020)
			*OH-13–2*	ss715614895	(29,971,253—30,065,880 a2)	Layer test/isolate *OH.121*	Germplasm (478)	2.6% IRW^j^	–	–	Rolling et al. (2020)
			*OH-13–3*	ss715614914	(30,086,805—30,144,416 a2)	Layer test/isolate *OH.121*	Germplasm (478)	8.0% IRW	–	–	Rolling et al. (2020)
				Gm13_29043806_T_C	(30,125,163- 30,154,255 a2)	Tray test/isolate *Win371*	SoyNAM RIL (122)	42.2%	–	HS6-3976	Scott et al. (2019)
			*OH-13–4*	ss715614952	(30,291,675—30,301,385 a2)	Layer test/isolate *OH.121*	Germplasm (478)	2.6% IRRS, 2.3% IRW	–	–	Rolling et al. (2020)
			*C2-13–6*	ss715614993	(30,502,735—30,618,405 a2)	Layer test/isolate *C2.S1*	Germplasm (495)	3.5% IRW	–	–	Rolling et al. (2020)
			*qHM13-1*	ss715615005	(30,628,076 a2)	Hypocotyl inoculation/isolates *PT2004 C2.S1, R7-2a, 1005–2.9*	Germplasm (448)	–	–	–	Van et al. (2020)
			*C2-13–7*	ss715615007	(30,646,059—30,654,291 a2)	Layer test/isolate *C2.S1*	Germplasm (495)	11.2% IRW	–	–	Rolling et al. (2020)
			*OH-13–5*	ss715615020	(30,667,266—30,700,217 a2)	Layer test/isolate *OH.121*	Germplasm (478)	3.1% ΔRW^k^	–	–	Rolling et al. (2020)
				Sct_033 (BARCSOYSSR_13_1230)	20.6—31.7 cM (30,739,608 a2)	Tray test/isolate *1.S.1.1*	F4:6 (375)	5.4%	–	–	Wang et al. (2010)
				Sct_033	34 cM (30,739,608 a1)	Hypocotyl inoculation/isolate *OH17*, race *2*	F7:8 and F7:9 (188)	20.1–35.8%	–	PI 408105A	Nguyen et al. (2012)
			*QTL 13–1*	ss715615031	(30,766,058 a2)	Tray test/isolates *OH121* and *C2S1*	PI lines (800)	2.5% Root weight	–	–	Schneider et al. (2016)
			*–*	SNP 35,123,596	(35,123,596 a1)	Slant board assay/isolate *P7076*	Germplasm (279)	–	Glyma13g32980, Glyma13g33900, Glyma13g33512	–	Li et al. (2016a, b, c)
				Gm13_39560450_G_A	(40,233,656- 42,919,730 a2)	Tray test/isolate *Win371*	SoyNAM RIL (122)	7.2%	–	HS6-3976	Scott et al. (2019)
			–	php2385	44-50 cM	Tray test/isolate C2S1	F10 and F11 (298)	7%	–	V71-370	Tucker et al. (2010)
			*QTL-13*	–	52-54 cM	Tray test and layer test/isolates *C.2.S.1, OH25, OH7, 1.S.1.1, OH30*	F7:8 (305)	13.2%-15.1%	–	PI 398841	Lee et al. (2014)
			*QTL-13*	BARC-051883–11,286 to BARC-042715–08,379	49-57 cM (24,848,378—44,053,323 a1)	Tray test/isolate *OH25*	F7:8 (157)	8.6%	–	PI 407861A	Lee et al. (2013b)
	MLG E (Chr. 15)	*qRps15-01*^$^	*QTL-15*	BARC-055329–13,210 to BARCSOYSSR_15_0160	16-19 cM (2,740,854–3,563,138 a1)	Tray test/isolate *OH25*	F7:8 (157)	7.2%	–	OX20-8	Lee et al. (2013b)
			*C2-15–1*	ss715621545	(2,952,387—3,182,673 a2)	Layer test/isolate *C2.S1*	Germplasm (495)	1.0% IRRS	–	–	Rolling et al. (2020)
		*qRps15-02*^$^	*qPR-15–1*	Satt651 (BARCSOYSSR_15_0306) and Satt598 (BARCSOYSSR_15_0645)	(6,823,519—13,653,981 a2)	Modified slant board assay/race *2*	F7:11 (176)	14.0–15.9%	–	Su88-M21	Wu et al. (2011c)
			*–*	Gm15_11496274	(11,496,274 a1)	Layer test/isolate ? (vir 1d, 2, 3b, 3c, 4, 5, 6, 7)	Cultivars (169)	11.7%	Glyma15g15030	–	Ludke et al. (2019)
	MLG J (Chr. 16)	*qRps16-01*^$^	*16–2*	BARC_2.0_Gm16_3124736 and BARC_2.0_Gm16_3362395	(3,124,736–3,362,395 a2)	Tray test/isolates *1.S.1.1,*	F9:11 (316)	5.4%	–	Sloan	Stasko et al. (2016)
			*16–2*	BARC_2.0_Gm16_3124736 and BARC_2.0_Gm16_3362395	(3,124,736–3,362,395 a2)	Tray test/isolates *PT2004C2.S1*	F9:11 (316)	3.5%	–	Sloan	Stasko et al. (2016)
	MLG D2 (Chr. 17)	*qRps17-01*^$^	*C2-17–1*	ss715626781	(33,515,060—33,574,931 a2)	Layer test/isolate *C2.S1*	Germplasm (495)	4.5% ΔRW	–	–	Rolling et al. (2020)
			*qHC17-1*	ss715626781	(33,549,403 a2)	Hypocotyl inoculation/isolate *PT2004 C2.S1*	Germplasm (460)	–	–	–	Van et al. (2020)
		*qRps17-02*^$^	*C2-17–2*	ss715627019	(36,411,792–36,398,362 a2)	Layer test/isolate *C2.S1*	Germplasm (495)	2.4% IRW	–	–	Rolling et al. (2020)
			–	Satt301-190	(36,718,722 a2)	Slant board assay/race *2*	China mini core collection (175)	5.2%	–	ZDD10252	Sun et al. (2014a, b)
	MLG G (Chr. 18)	*qRps18-01*^$^	*QDRL-18*	BARC-020839–03,962, BARC025777-05,064, and BARC-047665–10,370	13-16 cM (981,868/2,843,515 a2)	Tray test, layer test, and field test (OH, US)/isolates *OH7, OH7-8, OH25, OH12108, C2.S1, OH2010.739, OH2010.001*	F7:8 (367), F7:8 (338)	20.4%, 24.7%	–	PI 427105B and PI 427106	Lee et al. (2014), Karhoff et al. (2019)
			*OH-18–1*	ss715629906	(2,128,180–2,155,661 a2)	Layer test/isolate *OH.121*	Germplasm (478)	7.2% ISW	–	–	Rolling et al. (2020)
		*qRps18-02*^$^	*–*	BARC_2.0_Gm18_56710850 and BARC_2.0_Gm18_56766936	(56,710,850–56,766,936 a2)	Tray test/isolates *1.S.1.1*	F9:11 (316)	5.3%	–	Conrad	Stasko et al. (2016)
			*–*	BARC_2.0_Gm18_56710850 and BARC_2.0_Gm18_56876857	(56,710,850–56,876,857 a2)	Tray test/isolates *OH25*	F9:11 (316)	13.6%	–	Conrad	Stasko et al. (2016)
	MLG L (Chr. 19)	*qRps19-01*^$^	*19–1*	BARC-047496–12,943 and BARC_2.0_Gm19_46116996	(42,821,735 a1/46,116,996 a2)	Tray test/isolates *PT2004C2.S1*	F9:11 (316)	4.6%	–	Conrad	Stasko et al. (2016)
			*19–1*	BARCSOYSSR_19_1243 and BARCSOYSSR_19_1286	(43,533,689–44,370,710 a2)	Tray test/isolates *1.S.1.1,*	F9:11 (316)	3.1%	–	Conrad	Stasko et al. (2016)
			*19–1*	BARCSOYSSR_19_1286 and BARC_2.0_Gm19_46116996	(44,370,710–46,116,996 a2)	Tray test/isolates *OH25*	F9:11 (316)	9.1%	–	Conrad	Stasko et al. (2016)
		*qRps19-02*^$^	*19–2*	BARCSOYSSR_19_1452 and Glyma.19G226100	(47,528,116–47,787,869 a2)	Tray test/isolates *PT2004C2.S1*	F9:11 (316)	4.1%	–	Conrad	Stasko et al. (2016)
			*19–2*	BARCSOYSSR_19_1452 and Glyma.19G226100	(47,528,116–47,787,869 a2)	Tray test/isolates *OH25*	F9:11 (316)	7.8%	–	Conrad	Stasko et al. (2016)
		*qRps19-03*^$^	*19–3*	BARC_2.0_Gm19_50305134 and BARC-014385–01,342	(50,305,134 a2/50,222,676 a1)	Tray test / isolates *OH25*	F9:11 (316)	6.6%	–	Conrad	Stasko et al. (2016)
			*QTL 19–3*	ss715636056, ss715636059, ss715636064, ss715636073, ss715636076, ss715636077, ss715636083, ss715636084	(50,544,363–50,681,263 a2)	Tray test/isolates *OH121* and *C2S1*	PI lines (800)	2.5% Root rot score	–	–	Schneider et al. (2016)
			*QTL-19*	Chr19:50,040,258 and Chr19:50,556,102	(50,040,258–50,556,102 a2)	Hydroponic assay/mixed inoculum (pathotypes 1a, 1b, 1c, 1d, 1 k, 3a, 6, and 7)	F5:6 (147)	13.1% CDW	Glyma.19G262700	PI 449459	de Ronne et al. (2020)
*P. sansomeana*	MLG A1 (Chr.5)	–	*qPsan5.1*	Gm05_32565157_T_C and Gm05_32327497_T_C	54.71 cM (32,832,462–32,594,828 a2)	Modified layer test / *MPS17-22, V-NESO2 5–45, V-KSSO2 3–6, MPS17-24*(in combination with *qPsan16.1*), *C-NESO2 5–12*(in combination with *qPsan16.1*)	F4:5 (218)	6%	–	E13390	Lin et al. (2021)
	MLG J (Chr.16)	–	*qPsan16.1*	Gm16_35700223_G_T and Gm16_35933600_A_G/ Gm16_35816475_T_C	39.01 cM (36,203,537–36,436,443 a2)	Modified layer test / *MPS17-22, V-NESO2 5–45, V-KSSO2 3–6, MPS17-24*(in combination with *qPsan5.1*), *C-NESO2 5–12* (in combination with *qPsan5.1*), *C-IASO2 6–15, and MICO3-28*	F4:5 (218)	5.5%	–	E13901	Lin et al. (2021)

^$^Locus name given in this study

^a^Marker position (bp) based on the *Glycine max* genome assembly version *Gmax1.01* (a1), or *Gmax2.0* (a2), only starting position is shown for SSR markers

^b^Phenotypic variations explained by the molecular markers

^c^IRRS: inoculated root rot score

^d^%DRL: percentage of diseased root length

^e^%DSA: percentage of diseased root surface area

^f^ISW: Inoculated shoot weight

^g^CDSW: Corrected dry shoot weight

^h^IPH: inoculated plant height

^i^CDW: corrected dry weight

^j^IRW: inoculated shoot weight

^k^ΔRW: change in root weight

Fine mapping studies toward map-based cloning of *Rps* genes have also been reported. The first cloned *Rps* gene is the *Rps1k* from Williams82, from which three highly similar coiled coil (CC)-NBS-LRR genes were identified and verified through transgenic progenies (Gao et al. 2005). Unfortunately, none of these genes can be identified in any versions/sources of the Williams82 genome assemblies including unassembled contigs (Wang et al 2021). In another study, *RpsUN1* and *RpsUN2* were further narrowed to a 151 kb and 36 kb genomic regions using 826 F2:3 families. Expression analyses via reverse-transcription (RT)-PCR and RNA-seq suggested that *Glyma.03g034600* and *Glyma.16g215200*/*Glyma.16g214900* were high-confidence candidate genes for *RpsUN1* and *RpsUN2*, respectively (Li et al. 2016a, b, c). Most recently, a map-based cloning study revealed that the *Rps11* gene encoded a 27.7 kb NBS-LRR gene, and is derived from rounds of unequal recombination events, which resulted in promoter fusion and LRR expansion that contributed to the broad-spectrum resistance (Wang et al. 2021). More importantly, *Rps11* alone can defeat 127 isolates (80% of all tested isolates) widely distributed across the USA (Ping et al. 2016; Wang et al. 2021). It is expected that commercial soybean varieties carrying the *Rps11* gene will soon be available in the market.

In *Phytophthora* studies, pathogen inoculation methods to assess populations could also influence the outcome; For instance, hypocotyl inoculation has been a standard method to detect vertical resistance and is a premier step to exclude the influence of potential R genes before detecting horizontal resistance (Dorrance et al. 2018). On the other hand, the most commonly used methods to detect horizontal resistance to *P. sojae* are layer test and tray test which were based on colonized substrate to deliver the pathogen to the plant tissue (Dorrance et al. 2008; Wang et al. 2012). More recently, a hydroponic assay was developed that can detect both vertical and horizontal resistance through infection of soybean root system with zoospores (Lebreton et al. 2018). Different phenotypic traits can be collected including lesion size, root mass, shoot biomass, root scores, and corrected dry weight (CDW) (Dorrance et al. 2008; de Ronne et al. 2020, 2021). Twenty-one validated QTLs were stably identified in at least two independent studies (Table 5). These QTLs were distributed on 13 soybean chromosomes and may be of high priority to develop soybean varieties with horizontal resistance against *P. sojae*. Notably, *qRps18-01* (formerly named *QDRL-18* or *OH-18–1*), a major QTL conferring more than 20% of horizontal resistance (Lee et al. 2014; Karhoff et al. 2019; Rolling et al. 2020), as well as other newly identified QTLs, are being integrated into future soybean varieties through collaborated efforts. Moreover, more than 130 additional QTLs were also reported which provided diverse options for soybean breeders (Supplementary Table 1).

With respect to other *Phytophthora* species, *P. sansomeana* E.M. Hansen & Reeser is an emergent pathogen in soybean-producing areas and causes root rot diseases. Lin et al. (2021) identified and validated two QTLs that contributed horizontal resistance to this pathogen from improved soybean varieties developed at the Michigan State University soybean breeding program (Table 5). Marker-assisted resistance spectrum analysis indicated five patterns of interactions between QTLs and *P. sansomeana* isolates. The validated QTLs can be efficiently integrated into future soybean varieties using MAS with low linkage drag of undesirable agronomic traits, since both donor parents are improved soybean varieties.

## Pythium damping-off and root rot

The genus *Pythium* is typically linked with early-season diseases, such as seedling root rot and damping-off, and multiple species have been implicated (Zhang et al. 1998; Zhang and Yang. 2000). Among the most damaging species, *P. aphanidermatum*, *P. ultimum*, *P. irregulare*, and *P. sylvaticum* have been used to screen potential sources of resistance for breeding efforts to reduce the impact of these pathogens (Ellis et al. 2013b; Scott et al. 2019; Lin et al. 2020; Clevinger et al. 2021). Horizontal resistance is currently the only type of resistance identified for most *Pythium* species, except *Rpa1*, which was identified from cv. ‘Archer’ as a single dominant resistance gene against *P. aphanidermatum* (Table 6) (Cianzio et al. 1991; Kirkpatrick et al. 2006; Bates et al. 2008; Rosso et al. 2008)*.* The *Rpa1* gene is located on Chr. 13 (molecular linkage group F, MLG F), 10.6 cM and 26.6 cM from the SSR markers Satt510 and Satt114, respectively (Rosso et al. 2008)*.* In addition to *Rpa1*, two QTLs were identified for *P. aphanidermatum* from Archer, which were located on chromosomes 4 and 7, and accounting for 8.29–13.85% and 4.5–13.85% of phenotypic variations, respectively (Urrea et al. 2017). Moreover, Archer also confers resistance to seed rot and root discoloration caused by *P. ultimum* and other species of *Pythium* including *Phytopythium. vexans* (formerly *Pythium vexans*)*, P. irregulare*, and hyphal swelling (HS) group (Bates et al. 2004, 2008; Kirkpatrick et al. 2006; Rupe et al. 2011), yet the genes/QTLs conferring those resistances in Archer are unclear.

**Table 6:** Soybean loci associated with resistance to Pythium damping-off and root rot (caused by *Pythium* spp.)

Causal agent	MLG (Chr.)	Locus name	Tightly linked/flanking markers	Marker position cM (bp)^a^	Testing methods/Resistance spectrum	Population type (size)	PVE^b^	Donor source	References
*Pythium aphanidermatum*	MLG (Chr. 4)	*–*	ss715589319	49.00–51.00 cM (7,868,252 a2)	Seed plate assay and an infested vermiculite assay/isolate *64*	F2:6 (84)	8.3–13.8%	Archer	Urrea et al. (2017)
	MLG (Chr. 7)	*–*	ss715598762	121.20 cM (8,151,504 a2)	Seed plate assay and an infested vermiculite assay/isolate *64*	F2:6 (84)	4.5–13.9%	Archer	Urrea et al. (2017)
	MLG F (Chr. 13)	*Rpa1*	Satt510 and Satt114	(28,912,864–31,802,559 a2)	Hypocotyl inoculation/isolate *64*	F2:4 (86)	R gene	Archer	Rosso et al. (2008)
*Pythium irregulare*	MLG D1a (Chr. 1)	-	Satt515	(37,027,518 a2)	Greenhouse test/isolates *Br2-3–5, Cler1-4–1*	F2:3 (192)	14.0% Weight, 17.7% Root rot	PI424354	Ellis et al. (2013b)
		*–*	Gm01_49641478_A_G	(50,295,199–50,583,510 a2)	Tray test/isolate *Br2-3–5*	SoyNAM RIL (116)	6.9%	LG00-3372	Scott et al. (2019)
		*–*	Gm01_52253980_C_T	(53,141,084 a2)	Modified seed rot assay	F9 (307)	6.6%SRS^c^, 7.4%ROTS^d^	–	Clevinger et al. (2021)
	MLG D1b (Chr. 2)	*–*	Gm02_5035934_C_A	(5,054,610–5,321,601 a2)	Tray test/isolate *Br2-3–5*	SoyNAM RIL (91)	9.6%	LD02-9050	Scott et al. (2019)
		*–*	Gm02_6529620_G_A	(6,572,325–7,031,201 a2)	Tray test/isolate *Br2-3–5*	SoyNAM RIL (91)	7.8%	LD02-9050	Scott et al. (2019)
	MLG N (Chr. 3)	*–*	*–*	(31,912,038–33,553,037 a2)	Tray test/isolate *Br2-3–5*	SoyNAM RIL (116)	12.3%	LG00-3372	Scott et al. (2019)
		*–*	Gm03_45516951_G_A	(43,485,660–43,849,572 a2)	Tray test/isolate *Br2-3–5*	SoyNAM RIL (116)	6.9%	IA3023	Scott et al. (2019)
	MLG C1 (Chr. 4)	*–*	*–*	(5,314,249–5,903,949 a2)	Tray test/isolate *Br2-3–5*	SoyNAM RIL (91)	20.0%	IA3023	Scott et al. (2019)
		–	Gm04_5837752_G_A	(5,314,249–6,972,200 a2)	Tray test/isolate *Br2-3–5*	SoyNAM RIL (91)	12.2%	IA3023	Scott et al. (2019)
	MLG A1 (Chr. 5)	–	Gm05_389226_T_C	(2,220,637 a2)	Tray test/isolate *Br2-3–5*	SoyNAM RIL (91)	12.2%	IA3023	Scott et al. (2019)
		–	BARC_050697_09840	(31,837,726 a1)	Greenhouse test/isolates *Br2-3–5, Cler1-4–1*	F2:3 (192)	6.0%	PI424354	Ellis et al. (2013b)
		*–*	Gm05_40791973_A_G	(39,546,900 a2)	Modified seed rot assay	F9 (307)	5.5%SRS, 4.8%ROTS	–	Clevinger et al. (2021)
	MLG C2 (Chr. 6)	–	BARC_013837_01254	(14,247,105 a1)	Greenhouse test/isolates *Br2-3–5, Cler1-4–1*	F2:3 (192)	15.4%Weight, 14.9% Root rot	PI424354	Ellis et al. (2013b)
		–	Gm06_31863080_C_T	(32,783,474 a2)	Modified seed rot assay	F7 (198)	26.6%SRS, 6.1%ROTS	–	Clevinger et al. (2021)
	MLG A2 (Chr. 8)	–	BARC_032503_08989	(7,774,531 a1)	Greenhouse test/isolates *Br2-3–5, Cler1-4–1*	F2:3 (127)	12.6% Weight	PI424354	Ellis et al. (2013b)
		–	Gm08_8695745_A_C	(8,725,772 a2)	Modified seed rot assay	F9 (307)	16.7%SRS, 24.1%ROTS	–	Clevinger et al. (2021)
		–	BARC_041561_08032	(12,458,945 a1)	Greenhouse test/isolates *Br2-3–5, Cler1-4–1*	F2:3 (127)	10.3% Root rot	PI424354	Ellis et al. (2013b)
		–	BARC_021577_04150	(18,143,273 a1)	Greenhouse test/isolates *Br2-3–5, Cler1-4–1*	F2:3 (192)	8.8% Weight, 7.0% Root rot	PI424354	Ellis et al. (2013b)
	MLG O (Chr. 10)	–	BARC_018101_02517	(1,571,105 a1)	Greenhouse test/isolates *Br2-3–5, Cler1-4–1*	F2:3 (127)	14.7% Weight	PI424354	Ellis et al. (2013b)
		–	Gm10_43821942_T_C	(44,218,338 a2)	Tray test/isolate *Br2-3–5*	SoyNAM RIL (116)	10.0–10.9%	LG00-3372	Scott et al. (2019)
	MLG B1 (Chr. 11)	–	BARC_053481_11881	(3,675,507 a1)	Greenhouse test/isolates *Br2-3–5, Cler1-4–1*	F2:3 (127)	4.4% Weight	PI424354	Ellis et al. (2013b)
		–	Gm11_15558504_T_C	(25,067,208 a2)	Modified seed rot assay	F7 (198)	7.9%SRS	–	Clevinger et al. (2021)
		*qRRW11*	Gm11_36581897_A_G	(32,115,772 a2)	Greenhouse test/isolate *CMISO2-5–14*	F4:7 (79)	15.4%RRW^e^	E09088	Lin et al. (2018)
		–	Gm11_38289103_C_T	(34,177,149–34,296,488 a2)	Tray test/isolate *Br2-3–5*	SoyNAM RIL (91)	10.2%	LD02-9050	Scott et al. (2019)
	MLG F (Chr. 13)	–	BARC_900926_00961	(685,173 a1)	Greenhouse test/isolates *Br2-3–5, Cler1-4–1*	F2:3 (127)	8.2% Weight, 17.6% Root rot	PI424354	Ellis et al. 92013b)
		–	BARC_062009_17616	(19,330,554 a1)	Greenhouse test/isolates *Br2-3–5, Cler1-4–1*	F2:3 (192)	7.1% Weight, 6.3% Root rot	PI424354	Ellis et al. (2013b)
		–	–	(22,901,190–25,230,180 a2)	Tray test/isolate *Br2-3–5*	SoyNAM RIL (91)	12.4%	IA3023	Scott et al. (2019)
	MLG B2 (Chr. 14)	–	BARC_2.0_Gm14_2013931	(2,013,931 a2)	Greenhouse test/isolate *Brown 2–3-5*	F9:11 (316)	6.6%	Sloan	Stasko et al. (2016)
		–	BARC_065411_19443	(2,250,656 a1)	Greenhouse test/isolates *Br2-3–5, Cler1-4–1*	F2:3 (192)	7.4% Root rot	PI424354	Ellis et al. (2013b)
		–	BARC_015539_02002	(5,429,258 a1)	Greenhouse test/isolates *Br2-3–5, Cler1-4–1*	F2:3 (192)	8.3% Weight	PI424354	Ellis et al. (2013b)
	MLG J (Chr. 16)	–	Gm16_2780183_T_C	(1,034,335–3,225,680 a2)	Tray test/isolate *Br2-3–5*	SoyNAM RIL (116)	8.3%	LG00-3372	Scott et al. (2019)
		–	Gm16_27322120_C_T	(28,348,383 a2)	Tray test/isolate *Br2-3–5*	SoyNAM RIL (91)	12.4%	LD02-9050	Scott et al. (2019)
	MLG D2 (Chr. 17)	–	–	(4,610,230–6,517,544 a2)	Tray test/isolate *Br2-3–5*	SoyNAM RIL (116)	11.0%	IA3023	Scott et al. (2019)
		–	–	(6,517,544–7,100,289 a2)	Tray test/isolate *Br2-3–5*	SoyNAM RIL (91)	13.6%	IA3023	Scott et al. (2019)
	MLG G (Chr. 18)	–	–	(9,205,527–10,045,551 a2)	Tray test/isolate *Br2-3–5*	SoyNAM RIL (116)	9.3%	LG00-3372	Scott et al. (2019)
	MLG L (Chr. 19)	*19–2*	BARC_2.0_Gm19_47784141	(47,784,141 a2)	Greenhouse test/isolate *Brown 2–3-5*	F9:11 (316)	5.5%	Sloan	Stasko et al. (2016)
	MLG I (Chr. 20)	*qRRW20*	Gm20_1348454_T_G	(1,344,091 a2)	Greenhouse test/isolate *CMISO2-5–14*	F4:7 (113)	12.7%-13.3% RRW	E05226-T	Lin et al. (2018)
		–	BARC_052017_11314	(2,109,173 a1)	Greenhouse test/isolates *Br2-3–5, Cler1-4–1*	F2:3 (192)	8.3% Weight, 6.0% Root rot	PI424354	Ellis et al. (2013b)
*Pythium oopapillum*	MLG D1b (Chr. 2)	–	Gm02_47515175_G_A	(44,427,664 a2)	Modified seed rot assay	F8 (137)	8.9%SRS, 10.7%ROTS	–	Clevinger et al. (2021)
	MLG J (Chr. 16)	–	Gm16_6496577_A_C	(6,643,454 a2)	Modified seed rot assay	F7 (169)	8.6%SRS, 6.9%ROTS	–	Clevinger et al. (2021)
*Pythium sylvaticum*	MLG D1a (Chr. 1)	–	Gm01_52253980_C_T	(53,141,084 a2)	Modified seed rot assay	F9 (307)	5.7%ROTS	–	Clevinger et al. (2021)
	MLG C2 (Chr. 6)	–	Gm06_31863080_C_T	(32,783,474 a2)	Modified seed rot assay	F7 (198)	26.9%SRS, 26.2%ROTS	–	Clevinger et al. (2021)
	MLG A2 (Chr. 8)	–	Gm08_8695745_A_C	(8,725,772 a2)	Modified seed rot assay	F9 (307)	4.9%SRS, 21.4%ROTS	–	Clevinger et al. (2021)
	MLG O (Chr. 10)	–	Gm10_43004105_A_C	(43,489,645 a2)	Greenhouse test/isolate *CMISO2 2–30*	Germplasm (115), improved lines (99)	9.8%RRW	–	Lin et al. (2020)
		*q10.1*	Gm10_42975806_T_C	(43,517,944 a2)	Greenhouse test/isolate *CMISO2 2–30*	Germplasm (115), improved lines (99)	9.8%RRW	–	Lin et al. (2020)
			Gm10_42965189_G_T	(43,528,561 a2)	Greenhouse test/isolate *CMISO2 2–30*	Germplasm (115), improved lines (99)	9.8%RRW	–	Lin et al. (2020)
			Gm10_42963703_G_A and Gm10_43178809_G_T	109.21 cM (43,530,047–43,757,485 a2)	Greenhouse test/isolate *CMISO2 2–30*	F4:7 (113)	11.2%RRW	E05226-T	Lin et al. (2020)
		*q10.2*	Gm10_44563220_A_G and Gm10_44744804_A_C	123.61 cM (45,141,190–45,322,752 a2)	Greenhouse test/isolate *CMISO2 2–30*	F4:7 (113)	13.7%RRW	E05226-T	Lin et al. (2020)
		–	Gm10_48769298_A_G	(49,366,501 a2)	Greenhouse test/isolate *CMISO2 2–30*	Germplasm (115), improved lines (99)	10.2%RRW	–	Lin et al. (2020)
	MLG B2 (Chr. 14)	–	Gm14_4770786_C_T	(4,856,342 a2)	Modified seed rot assay	F7 (198)	12.2%SRS, 13.1%ROTS	–	Clevinger et al. (2021)
	MLG G (Chr. 18)	*q18.1*	Gm18_6584445_C_T Gm18_6636054_G_T	8.51 cM (6,609,920—6,661,172a2)	Greenhouse test/isolate *CMISO2 2–30*	F4:7 (113)	9.5%RRW	E05226-T	Lin et al. (2020)
		*q18.2*	Gm18_7898429_A_C	(7,920,476 a2)	Greenhouse test/isolate *CMISO2 2–30*	Germplasm (115), improved lines (99)	9.3%RRW	–	Lin et al. (2020)
			Gm18_7895324_G_A and Gm18_8851746_A_G	68.41 cM (7,917,371—8,886,122 a2)	Greenhouse test/isolate *CMISO2 2–30*	F4:7 (80)	11.3%RRW	E09088	Lin et al. (2020)
		–	Gm18_51777072_A_G	(47,496,041 a2)	Greenhouse test/isolate *CMISO2 2–30*	Germplasm (115), improved lines (99)	9.6%RRW	–	Lin et al. (2020)
		–	Gm18_57517100_C_T	(53,247,366 a2)	Modified seed rot assay	F8 (137)	12.0%SRS, 10.9%ROTS	–	Clevinger et al. (2021)
	MLG I (Chr. 20)	–	Gm20_2245263_G_A	(2,239,157 a2)	Greenhouse test/isolate *CMISO2 2–30*	Germplasm (115), improved lines (99)	8.1%RRW	–	Lin et al. (2020)
		*q20.1*	Gm20_36002148_T_C and Gm20_36095037_G_A	13.91 cM (37,097,315—37,190,252 a2)	Greenhouse test/isolate *CMISO2 2–30*	F4:7 (80)	16.5%RRW	E05226-T	Lin et al. (2020)
*Pythium torulosum*	MLG M (Chr. 7)	–	Gm07_16031010_C_T	(16,121,771 a2)	Modified seed rot assay	F7 (169)	7.9%SRS	–	Clevinger et al. (2021)
	MLG A2 (Chr. 8)	–	Gm08_8695745_A_C	(8,725,711 a2)	Modified seed rot assay	F9 (307)	66.6%SRS	–	Clevinger et al. (2021)
*Pythium ultimum* var*. sporangiiferum*	MLG N (Chr. 3)	–	Gm03_140242_G_A	(8,046–172,048 a2)	Tray test/isolate *Will1.6.7*	SoyNAM RIL (123)	9.7%	IA3023	Scott et al. (2019)
		–	Gm03_511376_C_T	(425,209–587,640 a2)	Tray test/isolate *Will1.6.7*	SoyNAM RIL (123)	15.9%	IA3023	Scott et al. (2019)
		–	–	(425,209–510,431 a2)	Tray test/isolate *Will1.6.7*	SoyNAM RIL (123)	10.1%	IA3023	Scott et al. (2019)
	MLG A1 (Chr. 5)	–	Gm05_41540078_C_A	(38,639,960–38,838,119 a2)	Tray test/isolate *Will1.6.7*	SoyNAM RIL (122)	10.2%	IA3023	Scott et al. (2019)
	MLG B1 (Chr. 11)	–	Gm11_36517294_T_C	(32,006,970 a2)	Tray test/isolate *Will1.6.7*	SoyNAM RIL (122)	9.2%	IA3023	Scott et al. (2019)
	MLG D2 (Chr. 17)	–	–	(37,799,552–38,965,056 a2)	Tray test/isolate *Will1.6.7*	SoyNAM RIL (122)	9.8%	HS6-3976	Scott et al. (2019)
*Pythium ultimum* var. *ultimum*	MLG D1a (Chr. 1)	–	–	(8,388,480–10,556,016 a2)	Tray test/isolate *Miami1-3–7*	SoyNAM RIL (94)	12.1%	IA3023	Scott et al. (2019)
	MLG D1b (Chr. 2)	*-*	-	(13,877,996–14,102,777 a2)	Tray test/isolate *Miami1-3–7, N201.2.2*	SoyNAM RIL (122)	9.2%	HS6-3976	Scott et al. (2019)
		*-*	Gm02_13904897_A_G	(13,877,996–14,206,854 a2)	Tray test/isolate *Miami1-3–7, N201.2.2*	SoyNAM RIL (122)	12.8%	HS6-3976	Scott et al. (2019)
	MLG N (Chr. 3)	*-*	Gm03_588585_C_T	(510,431–853,885 a2)	Tray test/isolate *Miami1-3–7*	SoyNAM RIL (94)	16.8%	IA3023	Scott et al. (2019)
	MLG A1 (Chr. 5)	*-*	Gm05_41903142_C_T	(38,388,163–38,446,748 a2)	Tray test/isolate *Miami1-3–7, N201.2.2*	SoyNAM RIL (75)	18.5%	IA3023	Scott et al. (2019)
	MLG C2 (Chr. 6)	–	BARC-055889–13,824 and BARC-064115–18,558	(17,236,088–17,966,360 a2)	Greenhouse cup assay/isolate *Miami 1–3-7*	F7:8 (247)	13.9–11.4% PS ^f^, 8.5–12.2%RW ^g^, 11.8–12.6%RRS ^h^	Magellan	Klepadlo et al. (2019)
		–	bin90–bin91	(17,234,790–17,585,393 a2)	Greenhouse cup assay/isolate *Miami 1–3-7*	F7:8 (247)	8.8%PS, 7.5–8.4%, 7.8–8.8%RRS	Magellan	Klepadlo et al. (2019)
	MLG M (Chr. 7)	–	Gm07_36955973_T_C	(30,099,305–36,907,555 a2)	Tray test/isolate *Miami1-3–7, N201.2.2*	SoyNAM RIL (75)	16.3%	S06-13,640	Scott et al. (2019)
	MLG A2 (Chr. 8)	–	bin36–bin37	(8,767,341–9,027,146 a2)	Greenhouse cup assay/isolate *Miami 1–3-7*	F7:8 (247)	8.8–12.3%PS, 11.4–16.8%RW, 7.8–11.4%RRS	Magellan	Klepadlo et al. (2019)
		–	BARC-010097–00,518 and Satt187	(8,937,354–9,192,645 a2)	Greenhouse cup assay/isolate *Miami 1–3-7*	F7:8 (247)	7.3% PS, 11.6% RW, 6.4% RRS	Magellan	Klepadlo et al. (2019)
		–	BARC-010097–00,518 and BARC 050,171–09,440	(8,937,354–9,457,315 a2)	Greenhouse cup assay/isolate *Miami 1–3-7*	F7:8 (247)	10.1% PS, 11.6% RW, 10.5% RRS	Magellan	Klepadlo et al. (2019)
	MLG F (Chr. 13)	–	–	(25,230,180–26,955,004 a2)	Tray test/isolate *Miami1-3–7*	SoyNAM RIL (94)	17.2%	IA3023	Scott et al. (2019)
		–	Gm13_40441579_G_T	(40,935,278–41,953,362 a2)	Tray test/isolate *Miami1-3–7*	SoyNAM RIL (94)	12.7%	IA3023	Scott et al. (2019)
	MLG D2 (Chr. 17)	–	–	(4,949,843–6,517,544 a2)	Tray test/isolate *Miami1-3–7, N201.2.2*	SoyNAM RIL (75)	24.4%	IA3023	Scott et al. (2019)
		–	Gm17_41060022_G_A	(40,457,644–40,876,232 a2)	Tray test/isolate *Miami1-3–7, N201.2.2*	SoyNAM RIL (122)	12.2%	HS6-3976	Scott et al. (2019)

^a^Marker position (bp) based on the *Glycine max* genome assembly version *Gmax1.01* (a1), or *Gmax2.0* (a2), only starting position is shown for SSR markers

^b^Phenotypic variations explained by the molecular markers

^c^SRS: seed rot severity

^d^ROTS: percent rotted seeds in inoculated plates

^e^RRW: ratio of fresh root weight

^f^PS: plant stand

^g^RW: fresh root weight

^h^RRS: root rot score

Horizontal resistance was also identified for other *Pythium* spp. Lin et al. (2020) identified and validated two QTLs for *P. sylvaticum* using QTL mapping and GWA methods. The two QTLs were located on chromosomes 10 and 18 and explained 9.8–11.2% and 9.3–11.3% of phenotypic variations, respectively. Remarkably, pleiotropic QTLs have been frequently identified for resistance to several *Pythium* species or varieties. For example, Scott et al. (2019) identified one QTL on Chr. 3 for resistance to *P. ultimum* var. *ultimum* and *P.ultimum* var. *sporangiiferum*, and other two QTLs (on chromosomes 13 and 17, respectively) that both confer resistance to *P. irregulare* and *P. ultimum* var. *ultimum*. In a more recent study, a major QTL was identified (nearest marker Gm08_8695745_A_C) conferring resistance to *P. irregulare* (16.7–24.1% of phenotypic variations), *P. sylvaticum* (4.9–21.4%), and *P. torulosum* (66.6%), and another large effect QTL (nearest marker Gm06_31863080_C_T) for resistance to *P. sylvaticum* (26.2–26.9%) and *P. irregulare* (6.1–26.6%) (Clevinger et al. 2021). In the future, these validated and pleiotropic QTLs will be of high priority in MAS to develop soybean varieties with tolerance to different *Pythium* pathogens.

## Downy mildew

Soybean downy mildew, caused by *Peronospora manshurica* (Naum.) Sdy., is a common leaf disease throughout the world (Lim et al. 1989). Although severe yield loss is rarely reported, soybean downy mildew can reduce the size and quality of soybean seeds (Palmer et al. 2004; Taguchi-Shiobara et al. 2019). Three resistance genes, *Rpm1*, *Rpm2*, and *Rpmx,* have been reported from soybean varieties ‘Kanrich’, ‘Fayette’, and PI 88788, and ‘AGS129’, respectively, although the genetic and physical location of the resistance genes remain unclear (Geeseman et al. 1950ab, Bernard and Cremeens 1971; Lim et al. 1984; Lim 1989; Chowdhury et al. 2002). Recently, quantitative resistance to soybean downy mildew was first reported in Japan (Taguchi-Shiobara et al. 2019). Remarkably, *QRpm3-1* and *QRpm7-1* were identified and confirmed in several mapping populations across multiple years, each explaining 18–72% and 28–91% of the observed phenotypes (Table 7).

**Table 7:** Soybean loci associated with resistance to downy mildew (caused by *Peronospora manshurica*)

MLG (Chr.)	Locus name	Tightly linked/flanking markers	Marker position	Testing methods/Resistance spectrum	Population type/size	PVE^a^	Donor source	References
*–*	*Rpm1*	*–*	*–*	Greenhouse test/races *1–32*		R gene	Kanrich	Geesman et al. ([Bibr CR163][Bibr CR164]), Bernard and Cremeens (1971), Lim et al. (1984)
*–*	*Rpm2*	*–*	*–*	Greenhouse test/races *2, 33*	F2/98–242, F3/55–106	R gene	Fayette and PI 88788	Lim et al. (1984), Lim (1989)
*–*	*Rpmx*	OPH-02_1250_ and OPP-10_831_	*–*	Field test (Thailand)	F2/102	R gene	AGS129	Chowdhury et al. (2002)
MLG D1b (Chr. 2)	*QRpm2-1*	WGSP02_0160-WGSP02_0170	Around 50 Mb	Field test (Japan)	F6 and F7(112)	8–10%	Harosoy	Taguchi-Shiobara et al. (2019)
MLG N (Chr. 3)	*QRpm3-1*	WGSP03_0040-WGSP03_0070	5-30 Mb	Field test (Japan)	F6 and F7(155), F5 and F6(190), F6 and F7(112)	18–72%	Fukuibuki, Kinusayaka, Harosoy	Taguchi-Shiobara et al. (2019)
MLG C1 (Chr. 4)	*QRpm4-1*	WGSP04_0120-WGSP04_0140	*–*	Field test (Japan)	F6 and F7(155)	4%	Satonohohoemi	Taguchi-Shiobara et al. (2019)
MLG C2 (Chr. 6)	*QRpm6-1*	WGSP06_0200-WGSP06_0210	*–*	Field test (Japan)	F6 and F7(155)	8%	Fukuibuki	Taguchi-Shiobara et al. (2019)
MLG M (Chr. 7)	*QRpm7-1*	WGSP07_0060-WGSP07_0070	Around 5 Mb	Field test (Japan)	F5 and F6 (189), F9 and F10(231), (F5 and F6(190)	6–91%	Tachinagaha, Suzumaru, COL/Akita2009/TARC/1	Taguchi-Shiobara et al. (2019)
	*QRpm7-2*	WGSP07_0080-WGSP07_0130	*–*	Field test (Japan)	F9 and F10(231)	47–91%	Suzumaru	Taguchi-Shiobara et al. (2019)
MLG A2 (Chr. 8)	*QRpm8-1*	WGSP08_0110-WGSP08_0130	Around 20 Mb	Field test (Japan)	F6 and F7(155)	13–24%	Fukuibuki	Taguchi-Shiobara et al. (2019)
MLG B1 (Chr. 11)	*QRpm11-1*	WGSP11_0100-WGSP11_0120	*–*	Field test (Japan)	F9 and F10(231)	4%	Suzumaru	Taguchi-Shiobara et al. (2019)
MLG H (Chr. 12)	*QRpm12-1*	WGSP12_0120-WGSP12_0130	Around 35 Mb	Field test (Japan)	F6 and F7(112)	6–8%	Harosoy	Taguchi-Shiobara et al. (2019)
MLG F (Chr. 13)	*QRpm13-1*	WGSP13_0080-WGSP13_0120	*–*	Field test (Japan)	F5 and F6 (189)	3%	Tachinagaha	Taguchi-Shiobara et al. (2019)
MLG B2 (Chr. 14)	*QRpm14-1*	WGSP14_0050-WGSP14_0060	*–*	Field test (Japan)	F5 and F6 (189)	4%	Tachinagaha	Taguchi-Shiobara et al. (2019)
MLG E (Chr. 15)	*QRpm15-1*	WGSP15_0130-WGSP15_0140	*–*	Field test (Japan)	F5 and F6(190)	3%	Kinusayaka	Taguchi-Shiobara et al. (2019)
MLG J (Chr. 16)	*QRpm16-1*	WGSP16_0090-WGSP16_0100	*–*	Field test (Japan)	F5 and F6(190)	3%	COL/Akita2009/TARC/1	Taguchi-Shiobara et al. (2019)
MLG G (Chr. 18)	*QRpm18-1*	WGSP18_0150-WGSP18_0160	Around 50-60 Mb	Field test (Japan)	F5 and F6 (189)	11–16%	Tachinagaha	Taguchi-Shiobara et al. (2019)
MLG L (Chr. 19)	*QRpm19-1*	WGSP19_0150-WGSP19_0170	*–*	Field test (Japan)	F6 and F7(155)	7%	Fukuibuki	Taguchi-Shiobara et al. (2019)
MLG I (Chr. 20)	*QRpm20-1*	WGSP20_0100-WGSP20_0130	*–*	Field test (Japan)	F5 and F6 (189)	4%	Tachinagaha	Taguchi-Shiobara et al. (2019)
	*QRpm20-2*	WGSP20_0090-WGSP20_0100	*–*	Field test (Japan)	F6 and F7(112)	5%	Harosoy	Taguchi-Shiobara et al. (2019)

^a^Phenotypic variations explained by the molecular markers

## Section III. Soybean resistance to fungal diseases

### Sudden death syndrome and Fusarium wilt and root rot

In the USA, Sudden Death Syndrome (SDS) was initially detected in the State of Arkansas in 1971 (Rupe and Weidemann 1986; Rupe 1989) and has since spread to the majority of soybean producing states (Hartman et al. 2016). In recent years, SDS has been detected in South Dakota (Tande et al. 2014), New York (Cummings et al. 2018), and North Dakota (Nelson et al. 2018). In Brazil, it was first observed in 1981/82 in the State of Minas Gerais (Nakajima et al. 1996). It received the name of red root rot (PVR), as it is still known in that country. This important disease also occurs in Argentina (Ploper 1993), Canada (Anderson and Tenuta 1998), Bolivia (Yorinori 1999), Paraguay (Yorinori 2002), and Uruguay (Ploper et al. 2003).

The major causal agent of SDS identified in the USA is the fungus *Fusarium virguliforme* O’Donnell and T. Aoki (formerly *F. solani* (Mart.) Sacc. f. sp. *glycines*) (Aoki et al. 2003), although a recent study reported that *F. Brasiliense* also causes SDS in the USA (Wang et al. 2019). SDS and *F. virguliforme* were also reported in Malaysia (Chehri et al. 2014) and South Africa (Tewoldemedhin et al. 2014). In Brazil, four fungi have been reported to cause SDS, including *F. virguliforme*, *F. brasiliense*, *F. crassistipitatum*, and *F. tucumaniae.* In addition, *F. brasiliense, F. crassistipitatum, and F. tucumaniae* have been reported to cause SDS in other countries in South America (Aoki et al. 2003, 2005, 2012).

Significant yield losses can occur due to SDS (Aoki et al. 2003). SDS favors cool and wet environment. The symptoms of SDS can be observed on the roots and the aboveground foliage. The fungus initiates its infestation by colonizing the soybean roots, causing root rot and necrosis, which leads to the loss of root mass and root nodules. The fungus may sporulate on the roots producing clusters of conidia that appear to be blue. The aboveground symptom of SDS is caused by the translocation of phytotoxin, the symptoms include interveinal chlorosis and necrosis; leaf abscission at the top of the petiole rather than the base; and eventually, early plant death. Foliage symptoms are generally observed in the later reproductive stages after flowering but may develop earlier (Roy et al. 1997; Aoki et al. 2003; Hartman et al. 2016; Chang et al. 2018).

Cultural practices and planting resistant varieties are the most common methods used to manage SDS (Wrather et al. 1995; Luckew et al. 2012). The soybean community has devoted substantial effort to identifying QTLs that underlie SDS resistance. To date, more than 200 resistance-associated markers have been identified (Table 8 and Supplementary Table 2). After mapping a resistance locus, it is important to confirm and incorporate it into multiple genetic backgrounds to determine whether it will maintain its effect and be useful in a breeding program. Based on the classification of Chang et al.(2018) as well as the studies thereafter, twenty-five confirmed QTLs have been identified from at least two independent studies (Table 8), including a single locus on chromosomes 2, 4, 5, 8, 9, 14, 16, and 19, two on chromosomes 3, 13, 15 and 17, and three on chromosomes 6, 18, and 20. Most of these loci were confirmed in at least one field study, except *qRfv06-03*, which was confirmed in three greenhouse studies (Abdelmajid et al. 2012; Bao et al. 2015; Luckew et al. 2017), and *qRfv20-03*, which was validated in a greenhouse study and a growth chamber study (Swaminathan et al. 2016; de Farias Neto et al. 2007). Notably, *qRfv05-01* confers resistance to both *F. virguliformes* and *F. tucumaniae*, a causal agent of SDS in South America*.* Ninety additional loci were also reported and may need confirmation in future studies (Supplementary Table 2). The confirmed QTLs can be pyramided into elite cultivars with high confidence for durable resistance. There are no reports on genetic mechanisms of the genes but, stacking the two distinct SDS resistance mechanisms, resistance to root rot and leaf scorch is the better strategy to increase resistance (Wang et al. 2016).

**Table 8:** Validated loci associated with resistance to soybean sudden death syndrome (SDS) disease (caused by *Fusarium virguliformes*)

Locus name^a^	MLG (Chr.)	Other names	Tightly linked/flanking markers	Marker position cM (bp)^b^	Testing methods/Resistance spectrum	Population type (size)	PVE^c^	Donor source	References
*qRfv02-01*	MLG D1b (Chr. 2)	*SDS13-5, qFDS003-02*	ss107920774—ss107912689	30.0–36.0 cM	Greenhouse test/isolate *Mont1*	F6:13 (50)	5.2% FDS^d^	PI 438489B	Abdelmajid et al. (2012)
		*–*	ss244884978	(49,773,810 a1)	Field test (MI, US)	Advanced breeding lines (300)	6.4% DI^e^	*–*	Wen et al. (2014)
		*SDS15-4*	BARC-041581–08,046—BARC-046084–10,230	93.341–02.59 cM (41,337,886–43,414,601 a2)	Growth chamber/isolates *Clinton 1B, Scott F2II 1a* and *Scott B2*	F7 derived RIL (200)	8.4% Stem cut	LS94-3207	Swaminathan et al. (2016)
*qRfv03-01*	MLG N (Chr. 3)	*SDS2-7, SDS QTL 1 N, qRfs6*	OC01_650_	*–*	Field test (IL, US)	F5:11 (100)	16% DI	Forrest	Chang et al. (1996)
		*SDS2-8, SDS QTL 1 N*	OF04_1600_	*–*	Field test (IL, US)	F5:11 (100)	10% DI	Forrest	Chang et al. (1996), Chang et al. (2018)
		*SDS1-2, SDS1-4*	OC01_650_	*–*	Field test (IL, US)	F5:9 (100)	30% DI	Forrest	Hnetkovsky et al. (1996), Chang et al. (2018)
		*–*	*ss715586494_C_T*	(44,251,912 a2)	Greenhouse test	Germplasm (214)	9% DAI29^f^	*–*	Zhang et al. (2015a), Chang et al. (2018)
*qRfv03-02*	MLG N (Chr. 3)	*SDS13-10, qRRS001-01*	ss107912585—ss107920575	38.3–42.6 cM	Greenhouse test/isolate *Mont1*	F6:13 (50)	9.9% RRS^g^	PI 438489B	Abdelmajid et al. (2012), Chang et al. (2018)
		*qDX004*	ss245025977—ss245026227	15.90–16.10 cM	Field test (IL, US)	F5:7 (94)	0.1% DX ^h^	*–*	Anderson et al. (2015), Chang et al. (2018)
		*SDS14-2, qDX003*	ss245026358—ss245025977	15.70–15.90 cM	Field test (IL, US)	F5:7 (94)	0.8% DX	*–*	Anderson et al. (2015), Chang et al. (2018)
		*SDS8-3, qRfs6*	Satt080	*–*	Field test (IL, US)	F6 derived (90); F2:3 (321)	15.6% DI	Pyramid	Njiti et al. (2002), Luckew et al. (2013), Chang et al. (2018)
		*Di1, qRfs6*	Satt080—Satt387	51.61 cM	Field test (IL, US)	RIL (94); F2:3 (321)	15.9% DI	Forrest	Kassem et al. (2006), Luckew et al. (2013), Chang et al. (2018)
		*ds2, qRfs6*	Satt080—Satt387	51.61 cM	Field test (IL, US)	RIL (94); F2:3 (321)	14.2% DS^i^	Forrest	Kassem et al. (2006), Luckew et al. (2013), Chang et al. (2018)
		*dx1, qRfs6*	Satt080—Satt387	49.61 cM	Field test (IL, US)	RIL (94); F2:3 (321)	17.3% DX	Forrest	Kassem et al. (2006), Luckew et al. (2013), Chang et al. (2018)
		*di8, QRfs7*	Satt080—Satt387	51.61 cM	Field test (IL, US)	RIL (100)	15.9%		Abdelmajid et al. (2007), Chang et al. (2018)
		*-*	Satt387	(34,554,705 a2)	Field test (IL, US)	F6 derived (90)	10.2% DI	Pyramid	Njiti et al. (2002)
*qRfv04-01*	MLG C1 (Chr. 4)	*SDS13-15, qFDS004-03*	ss107924445—ss107918378	57.3–83.9 cM	Greenhouse test/isolate *Mont1*	F6:13 (50)	4.8%	PI 438489B	Abdelmajid et al. (2012)
		*SDS9-3*	A063_1	*–*	Greenhouse test/Strain ST-90	F7:14 (284)	5% DS	Noir1'	Njiti and Lightfoot (2006)
		*SDS disease incidence 20–1, SDS disease index 20–1, qSDS-4*	ss245526764—ss245561373	48.56–83.86 cM (43.8 Mb–47.3 Mb a1)	Field test (MI, US)	F4 derived (129)	3.7–5.3% DI, DX	GD2422	Tan et al. (2018)
		*qPY4-1*	ss245560843—ss245567348	(47.29 Mb48.08 Mb a2)	Field test (MI, US)	F4 derived (153)	6.36% plot yield	E07080	Tan et al. (2019)
*qRfv05-01*	MLG A1 (Chr. 5)	*SDS14-4, qDX005*	ss245747167-ss245786667	9.20–10.00 cM/8.50–11.70 cM	Field test (IL, US)	F5:7 (94)	0.01–0.04% DX	-	Anderson et al. (2015)
		*SDS15-8*	BARC-059081–15,595 to BARC-065229–19,273	57.79–78.44 cM (35,367,094–37,815,203 a2)	Growth chamber/isolates *Clinton 1B, Scott F2II 1a* and *Scott B2*	F7 derived RIL (200)	7.0% Root feeding	LS94-3207	Swaminathan et al. (2016)
		*RSDS3*	Satt545	(36,463,225 a2)	Greenhouse test/*F. tucumaniae* sp. nov. MJ161	F8 derived (156)	9.3% DX	Moshidou Gong 503	Yamanaka et al. (2006)
*qRfv06-01*	MLG C2 (Chr. 6)	*di5, qRfs4*	Satt371	4.4 cM (49,760,138 a2)	Field test (IL, US)	RIL (100); F2:3 (321)	12.1% DI	Essex	Abdelmajid et al. (2007), Luckew et al. (2013), Chang et al. (2018)
		*SDS14-6, qDX007*	ss246087580-ss246092064	7.20–7.50 cM	Field test (IL, US)	F5:7 (94)	0.6% DX	*–*	Anderson et al. (2015), Chang et al. (2018)
		*qDX008*	ss246091245-ss246092064	7.20–7.30 cM	Field test (IL, US)	F5:7 (94)	0.5% DX	*–*	Anderson et al. (2015), Chang et al. (2018)
		*SDS2-5, SDS QTL 1C2*	OO05_250_	*–*	Field test (IL, US)	F5:11 (100)	13% DI	Essex	Chang et al. (1996), Chang et al. (2018)
		*SDS2-6, SDS QTL 1C2*	K455D-1	*–*	Field test (IL, US)	F5:11 (100)	16% DI	Essex	Chang et al. (1996), Chang et al. (2018)
		*SDS1-1*	OO05_250_	*–*	Field test (IL, US)	F5:9 (100)	26% DI	Essex	Hnetkovsky et al. (1996), Chang et al. (2018)
		*SDS7-5, qRfs4*	Satt371	(49,760,138 a2)	Field test (IL, US)	F5:13 (100); F2:3 (321)	12.0% DI	Essex	Iqbal. et al. (2001), Luckew et al. (2013), Chang et al. (2018)
		*SDS8-2*	Satt307	*–*	Field test (IL, US)	F6 derived (90)	13.6% DI	Douglas	Njiti et al. (2002), Chang et al. (2018)
		SDS16-5	BARC-010457–00,640—BARC-025767–05,060	121.26–126.23 cM (45,851,263–48,305,238 a2)	Growth chamber/isolates *Clinton 1B, Scott F2II 1a* and *Scott B2*	F7 derived RIL (200)	8.6% Root feeding	A95-684043	Swaminathan et al. (2016), Chang et al. (2018)
		*SDS14-5, qDX006*	ss246091245—ss246092064	7.20–7.30 cM	Field test (IL, US)	F5:7 (94)	0.9% DX	*–*	Anderson et al. (2015), Chang et al. (2018)
		*SDS4-2*	K455	*–*	Field test (IL, US)	F5:13 (100)	2%—9% DX, IS	Essex'	Njiti et al. (1998)
		*–*	ss246038868	*–*	Field test (MI, US)	Advanced breeding lines (300)	5.7% DX	*–*	Wen et al. (2014)
		*qSDS6-6*	ss246084690—ss246086447	(46.11–46.25 a2)	Field test (MI, US)	F4 derived (153)	8.8%	E07080	Tan et al. (2019)
		*qSDS6-7*	ss246098726—ss246102570	(47.27–57.59 a2)	Field test (MI, US)	F4 derived (153)	10.8%	E07080	Tan et al. (2019)
*qRfv06-02*	MLG C2 (Chr. 6)	*ds6, QRfs5*	Satt489—Satt286	99.21 cM (16,221,044–23,848,501 a2)	Field test (IL, US)	RIL (100)	12.1% DI	Essex	Abdelmajid et al. (2007), Chang et al. (2018)
		*ds1*	Satt489—Satt286	99.21 cM (16,221,044–23,848,501 a2)	Field test (IL, US)	RIL (94)	15.4% DS	Essex	Kassem et al. (2006), Chang et al. (2018)
		*SDS11-1, cqRfs4*	Satt277, Satt079	(17,218,677, 44,503,658 a2)	Field test (IL, US)	F5:14 (92)	8.2%-24.1% DX	Flyer	Kazi et al. (2008), Chang et al. (2018)
		*SDS16-6*	BARC-021735–04,194—BARC-062515–17,881	97.83–121.26 cM (16,029,425–46,596,066 a2)	Growth chamber/isolates *Clinton 1B, Scott F2II 1a* and *Scott B2*	F7 derived RIL (200)	12% Root feeding	A95-684043	Swaminathan et al. (2016), Chang et al. (2018)
		*qSDS6-3*	ss245888974—ss245909007	(15.03–16.81 Mb a2)	Field test (MI, US)	F4 derived (153)	7.0–9.5%	E07080	Tan et al. (2019)
		*qSDS6-4*	ss245925990—ss246010254	(18.63*–*39.82 Mb a2)	Field test (MI, US)	F4 derived (153)	9.8%	E07080	Tan et al. (2019)
		*qSDS6-5*	ss246041195—ss246068439	(43.16*–*44.82 Mb a2)	Field test (MI, US)	F4 derived (153)	11.1%	E07080	Tan et al. (2019)
		*qPY6-1*	ss245879277—ss245882767	(14.05*–*14.42 Mb a2)	Field test (MI, US)	F4 derived (153)	4.8% plot yield	E07080	Tan et al. (2019)
		*SDS13-6, qFDS003-03, qFDS004-04*	ss107929602—ss107925487 ss107930961—ss107912561	32.8*–*39.2 cM/34.5*–*39.8 cM	Greenhouse test/isolate *Mont1*	F6:13 (50)	3.2–4.7% FDS	PI 438489B	Abdelmajid et al. (2012)
*qRfv06-03*	MLG C2 (Chr. 6)	*SDS13-14, qFDS003-05, qFDS004-03*	ss107917031—ss107912977	16.9*–*32.8 cM (12,273,659*–*17,424,199 a1)	Greenhouse test/isolate *Mont1*	F6:13 (50)	2.1–2.4%	PI 438489B	Abdelmajid et al. (2012)
		*cqRfs4*	BARC-028177–05,786	(13,551,218 a1)	Greenhouse test/isolate *Somerset #1A*	Ancestral lines, advanced breeding lines, cultivars, and landraces (282)	*–*	*–*	Bao et al. (2015)
		*Fusarium root rot 1–1*	Gm06_13621986_A_C to Gm06_15571070_T_C	(13,621,986–15,571,070 a1)	Greenhouse test/isolate *LL0009* (NE305)	F2:3 (200)	18.0% Root rot severity	MN1606SP	Luckew et al. (2017)
*qRfv08-01*	MLG A2 (Chr. 8)	*SDS13-13, qFDS003-06*	ss107915722—ss107918074	15.0–28.0 cM (4,646,825–9,489,665 a1)	Greenhouse test/isolate *Mont1*	F6:13 (50)	17.40%	PI 438489B	Abdelmajid et al. (2012)
		*SDS15-2*	BARC-031701–07,215—BARC-016685–03,321	14.99–51.86 cM (3,060,343–8,204,715 a2)	Growth chamber/isolates *Clinton 1B, Scott F2II 1a* and *Scott B2*	F7 derived RIL (200)	8.4% Stem cut	A95-684043	Swaminathan et al. (2016)
		*SDS15-3*	BARC-016685–03,321—BARC-038631–07,266	51.86–58.43 cM (8,204,715–10,215,938 a2)	Growth chamber/isolates *Clinton 1B, Scott F2II 1a* and *Scott B2*	F7 derived RIL (200)	5.8% Stem cut	A95-684043	Swaminathan et al. (2016)
		*SDS disease incidence 20–2, qSDS-8*	ss246481149—ss246509551	70.15–84.97 cM (7.8–10.8 Mb a1)	Field test (MI, US)	F4 derived (129)	5.2–8.5% DI	LD01-5907	Tan et al. (2018)
*qRfv09-01sss*	MLG K (Chr. 9)	*SDS14-7, qDX009*	ss246870684—ss246865400	0.40–0.50 cM	Field test (IL, US)	F5:7 (94)	0.5% DX	*–*	Anderson et al. (2015), Chang et al. (2018)
		*SDS16-1*	BARC-056323–14,257—BARC-010353–00,615	45.74–50.93 cM (22,251,525–38,869,688 a2)	Growth chamber/isolates *Clinton 1B, Scott F2II 1a* and *Scott B2*	F7 derived RIL (200)	13% Stem cut	LS98-0582	Swaminathan et al. (2016), Chang et al. (2018)
		*qRfs18*	Satt381	(12,849,836 a2)	–	–	–	–	Lightfoot et al. (2015), Chang et al. (2018)
		*qSDS9-1*	ss246827311—ss246949164	(7.00–34.27 Mb a2)	Field test (MI, US)	F4 derived (153)	9.0–11.7%	U01-390489	Tan et al. (2019)
		*SDS18-3, SDS-3*	BARC-058901–15,494—BARC-050815–09,887; Satt552–Satg002	(11,775,727–33,502,306 a2)	Growth chamber/isolates *Clinton 1B* and *Scott F2II 1a*	F7:8 (200)	4.6%	LS94-3207	Swaminathan et al. (2018)
*qRfv13-01*	MLG F (Chr. 13)	*dx1, QRfs9*	Satt510	27.3 cM (31,802,559 a2)	Field test (IL, US)	RIL (100)	10.2% DX	Forrest	Abdelmajid et al. (2007), Chang et al. (2018)
		*SDS disease incidence 21–1*	Gm13_26749514_T_C—Gm13_42027425_G_T	(27,943,258–43,467,121 a2)	Greenhouse test/isolate *LL0009* (NE305)	F2:3 (200)	11.4% DI	Spencer	Luckew et al. (2017), Chang et al. (2018)
		*SDS15-1*	BARC-065495–19,507—BARC-030899–06,963	72.97–78.05 cM (29,074,011–30,510,485 a2)	Growth chamber/isolates *Clinton 1B, Scott F2II 1a* and *Scott B2*	F7 derived RIL (200)	16% Stem cut	A95-684043	Swaminathan et al. (2016), Chang et al. (2018)
		*SDS16-8*	BARC-010501–00,676—BARC-042515–08,280	74.12–78.05 cM (29,598,124–30,174,729 a1)	Growth chamber/isolates *Clinton 1B, Scott F2II 1a* and *Scott B2*	F7 derived RIL (200)	12% Root feeding	LS98-0582	Swaminathan et al. (2016), Chang et al. (2018)
		*–*	ss248117124	(34,867,303 a2)	Field test (MI, US)	Advanced breeding lines (300)	5.7% DX	–	Wen et al. (2014), Chang et al. (2018)
		*–*	ss715614656_G_A	(28,548,247 a2)	Greenhouse test	Germplasm (214)	6% DAI20	–	Zhang et al. (2015a, b), Chang et al. (2018)
*qRfv13-02*	MLG F (Chr. 13)	*–*	SGM13_13250813		Growth chamber	F2 lines (135)	12.6% foliar necrosis	PI 243518	Chang et al. (2020)
		*ds1, QRfs8*	Satt160—Satt252	(16,454,986–17,875,691 a2)	Field test (IL, US)	RIL (100); F2:3 (321)	16.9% DS, 11.20% DX	Forrest	Abdelmajid et al. (2007), Luckew et al. (2013) Chang et al. (2018)
		*ds3, qRfs12*	Satt160—Satt252	(16,454,986–17,875,691 a2)	Field test (IL, US)	RIL (94); F2:3 (321)	6.3% DS	Essex	Kassem et al. (2006), Luckew et al. (2013), Chang et al. (2018)
		*–*	Gm13-4,584,015	(17,285,679 a2)	Field test (MI, US)	Cultivars (392)	7.2% DI	–	Wen et al. (2014)
		*SDS17-2, SDS-5*	BARC-900926–00,961—BARC-041237–07,944; Sat_298–Satt423	(16,825,744–21,179,508 a2)	Growth chamber/isolates *Clinton 1B* and *Scott F2II 1a*	F7:8 (200)	9%	LS98-0582	Swaminathan et al. (2018)
		*–*	Sat_039—Satt160	–	Field test (IL, US)	F5:14 (92)	20% DS, 19% DX	Forrest	Yuan et al. (2012)
*qRfv14-01*	MLG B2 (Chr. 14)	*SDS14-10, qDX012*	ss248293401—ss248275088	1.40–4.10 cM	Field test (IL, US)	F5:7 (94)	0.03% DX	–	Anderson et al. (2015)
		*qDX013*	ss248293401—ss248275088	12.90–18.20 cM	Field test (IL, US)	F5:7 (94)	0.03% DX	–	Anderson et al. (2015)
		*SDS14-11, qDX014*	ss248293401—ss248275088	10.30–13.00 cM	Field test (IL, US)	F5:7 (94)	6.4% DX	–	Anderson et al. (2015)
		*SDS disease index 21–1*	Gm14_7195140_G_A—Gm14_27937142_C_T	(7,302,532–32,105,943 a2)	Greenhouse test/isolate *LL0009* (NE305)	F2:3 (200)	34.4% DX	MN1606SP	Luckew et al. (2017)
		*–*	ss715617333_C_T	(9,890,873 a2)	Growth chamber/isolates *Mont-1, Scott F2I11a* and *Clinton 1B*	PI lines (254)	8.6%	–	Swaminathan et al. (2019)
*qRfv15-01*	MLG E (Chr. 15)	*qDX016*	ss248604753—ss248616287	1.40–2.70 cM	Field test (IL, US)	F5:7 (94)	0.6% DX	–	Anderson et al. (2015)
		*SDS disease incidence 21–2*	Gm15_9733870_T_C—Gm15_15746095_G_A	(9,887,588–15,771,590 a2)	Greenhouse test/isolate *LL0009* (NE305)/	F2:3 (200)	9.7% DI	MN1606SP	Luckew et al. (2017)
*qRfv15-02*	MLG E (Chr. 15)	*qDX015*	ss248604753—ss248616287	1.50–3.00 cM	Field test (IL, US)	F5:7 (94)	0.05% DX	–	Anderson et al. (2015)
		*SDS disease incidence 21–2*	Gm15_13651090_G_A—Gm15_47871831_A_C	(13,666,730–48,664,536 a2)	Greenhouse test/isolate *LL0009* (NE305)/	F2:3 (200)	13.0% DX	MN1606SP	Luckew et al. (2017)
		*–*	ss248698930	(20,239,752 a1)	Field test (MI, US)	Cultivars (392)	7.7% DX	–	Wen et al. (2014)
*qRfv16-01*	MLG J (Chr. 16)	*SDS15-7*	BARC-016775–02,320—BARC-014745–01,638	27.99–38.70 cM (4,273,014–7,026,287 a2)	Growth chamber/isolates *Clinton 1B, Scott F2II 1a* and *Scott B2*	F7 derived RIL (200)	5.2% Stem cut	LS94-3207	Swaminathan et al. (2016), Chang et al. (2018)
		*SDS14-12, qDX017*	ss248983974—ss248977568	11.50–14.00 cM	Field test (IL, US)	F5:7 (94)	0.9% DX	–	Anderson et al. (2015), Chang et al. (2018)
		*–*	Satt285a—Satt132	(2,827,903–9,934,104 a2)	Field test (IL, US)	F5:14 (92)	39% DI	–	Yuan et al. (2012), Chang et al. (2018)
*qRfv17-01*	MLG D2 (Chr. 17)	*SDS11-2, cqRfs11, qRfs7s*	Satt574, Sat_001	(31,915,278 a1, 36,455,269 a2)	Field test (IL, US)	F5:14 (92); F2:3 (321)	10.2–25.2% IS	Flyer	Kazi et al. (2008), Luckew et al. (2013), Chang et al. (2018)
		*–*	BARC-059487–15,840	(34,725,321 a2)	Greenhouse test/isolate *Somerset #1A*	Ancestral lines, advanced breeding lines, cultivars, and landraces (282)	*–*	*–*	Bao et al. (2015), Chang et al. (2018)
		*–*	BARC-061049–17,016	(35,707,915 a2)	Greenhouse test/isolate *Somerset #1A*	Ancestral lines, advanced breeding lines, cultivars, and landraces (282)	*–*	*–*	Bao et al. (2015) Chang et al. (2018)
		*–*	Sat_001	(36,455,269 a2)	*–*				Lightfoot et al. (2015), Chang et al. (2018)
*qRfv17-02*	MLG D2 (Chr. 17)	*-*	BARC-051665–11,191	(14,613,850 a2)	Greenhouse test/isolate *Somerset #1A*	Ancestral lines, advanced breeding lines, cultivars, and landraces (282)	*–*	*–*	Bao et al. (2015)
		*SDS19-2*	Satt389	(13,771,699 a2)	Field tests (IA/MI/IL, US)	F5 derived (91)	17.9% DX	Ripley	Brzostowski et al. (2018)
		*qRfs7*	Satt222—Satt389	–	Greenhouse test/isolate *FSG-1*	F4 derived (96)	11%	PI 567374	de Farias Neto et al. (2007)
*qRfv18-01*	MLG (Chr. 18)	*SDS4-3, SDS6-1, rfs1*	Bng122D/Bng122_1	–	Field test (IL, US)	F5:13 (100)	16–18% DX, 38–73% IS ^j^	Forrest	Njiti et al. (1998), Meksem et al. (1999) Chang et al. (2018)
		*G-QTL-3*	Satt130, Satt356, Satt570	(4,639,971/3,172,879 a2)	Field test (IL, US)	RIL (100)	11.9–19.2% DI, 8.6–13.9%	Forrest	Abdelmajid et al. (2007), Chang et al. (2018)
		*G-QTL-2, Rfs2*	Satt309, Satt594, Satt217, OI03-P4	(1,736,832/22,375,695/ 4,713,265 a2)	Field test (IL, US)	RIL (100); F2:3 (321)	12.5–17.7% DI, 10.1–13.3% DX	Forrest	Abdelmajid et al. (2007), Luckew et al. (2013), Chang et al. (2018)
		*SDS2-1, SDS3-1, SDS QTL 1G*	OG13_490_	–	Field test (IL, US)	F5:11 (100)	17% DI, 10% DS	Forrest	Chang et al. (1996), Chang et al. (2018)
		*SDS2-2, SDS3-2, SDS QTL 1G*	OI03_450_	–	Field test (IL, US)	F5:11 (100)	20% DI, 12% DS	Forrest	Chang et al. (1996), Chang et al. (2018)
		*rfs1*	OI03_450_	–	Field test (IL, US)	F5 derived (199)	4–38% DX, 39–47% IS	Forrest	Meksem et al. (1999), Chang et al. (2018)
		*SDS7-1*	Satt214	–	Field test (IL, US)	F5:13 (100)	24.1% DI	Forrest	Iqbal. et al. (2001), Chang et al. (2018)
		*SDS4-1, SDS4-3*	OI03_512_	–	Field test (IL, US)	F:13 (100)	21–47% DX, IS	Forrest'	Njiti et al. (1998), Chang et al. (2018)
		*SDS7-3*	Satt570	(3,172,879 a2)	Field test (IL, US)	F5:13 (100)	19.2% DI	Forrest	Iqbal. et al. (2001), Chang et al. (2018)
		*SDS7-2, Rfs2*	Satt309	(1,736,692 a2)	Field test (IL, US)	F5:13 (100); F2:3 (321)	16.3% DI	Forrest	Iqbal. et al. (2001), Luckew et al. (2013) Chang et al. (2018)
		*SDS11-3, cqRfs1*	Satt038_2	(1,344,090 a2)	Field test (IL, US)	F5:14 (92)	28.1% IS	Hartwig	Kazi et al. (2008), Chang et al. (2018)
		*SDS11-4, cqRfs13*	Satt130	(4,639,971 a2)	Field test (IL, US)	F5:14 (92)	12.9% DX	Hartwig	Kazi et al. (2008), Chang et al. (2018)
		*SDS6-2, Rfs2*	Satt309	(1,736,692 a2)	Field test (IL, US)	F5:13 (100); F2:3 (321)	14–63%, 8%-9% IS	Forrest	Meksem et al. (1999), Luckew et al. (2013)
		*-*	Satt309	(1,736,692 a2)	Field test (IL, US)	F5:14 (92)	18% DI, 12% DS, 14% DX	Forrest	Yuan et al. (2012), Chang et al. (2018)
		*SDS8-1, qRfs3*	Satt163	–	Field test (IL, US)	F6 derived (90); F2:3 (321)	16.0% DI	Pyramid	Njiti et al. (2002), Luckew et al. (2013), Chang et al. (2018)
		*Rfs2*	Satt309	(1,736,692 a2)	Field test (IL, US)	F6 derived (90); F2:3 (321)	8.5% DI	Pyramid	Njiti et al. (2002), Luckew et al. (2013) Chang et al. (2018)
		*SDS5-1, Rfs1*	Satt038	(1,344,090 a2)	Field test (IL, US)	F5 derived (94)	0.5% R6, 28% R8 IS	Hartwig	Prabhu et al. (1999), Chang et al. (2018)
		*Rfs2*	GmRLK18-1 (Glyma18g02680)	(1,711,924—1,714,468 a1)	Greenhouse test/strain *Mont-1*	-	-	-	Srour et al. (2012), Chang et al. (2018)
		*qRfs2*	Satt309, TMD, SIUC-Sat1	(1,736,692 a2)	Field test (IL, US)	F5 derived (100); F2:3 (321)	-	Forrest	Triwitayakorn et al. (2005), Luckew et al. (2013), Chang et al. (2018)
		*–*	ss249511029	(1,611,921 a1)	Field test (MI, US)	Advanced breeding lines (300)	9.3% DX	-	Wen et al. (2014), Chang et al. (2018)
		*–*	Gm18-1,709,751	(1,709,751 a1)	Field test (MI, US)	Cultivars (392)	10.6% DX	–	Wen et al. (2014)
		*–*	ss249517154	(2,113,196 a1)	Field test (MI, US)	Advanced breeding lines (300)	8.3% DI	–	Wen et al. (2014)
		*G-QTL-1*	Satt214, Satt275	1,239,847 (a2)	Field test (IL, US)	RIL (100)	11.4–24.2% DI, 23.0% DS, 10.0–25.5% DX	Forrest	Abdelmajid et al. (2007), Chang et al. (2018)
		*dx2*	Satt214—Satt275	1,239,847 (a2)	Field test (IL, US)	RIL (94)	6.92% DX	Essex	Kassem et al. (2006)
		*di2*	Satt214—Satt275	1,239,847 (a2)	Field test (IL, US)	RIL (94)	7.45% DI	Essex	Kassem et al. (2006)
		*ds5*	ACC230—Satt214	103.7–110.5 cM	Field test (IL, US)	RIL (94)	17.30% DS	Essex	Kassem et al. (2006)
		*–*	Satt038	(1,344,090 a2)	Field test (IL, US)	F6 derived (90)	12.3% DI	Pyramid	Njiti et al. (2002)
*qRfv18-02*	MLG G (Chr. 18)	*SDS2-4, SDS3-3, SDS QTL 2G*	OE02_1000_	–	Field test (IL, US)	F5:11 (100)	10% DI, 12% DS	Forrest	Chang et al. (1996), Chang et al. (2018)
		*SDS7-4*	OEO2_1000_	–	Field test (IL, US)	F5:13 (100)	12.6% DI	Forrest	Iqbal. et al. (2001), Chang et al. (2018)
		*SDS13-9, SDS13-12, qFDS004-02, qRRS001-03*	ss4969823—ss107924619	24.4–28.1 cM (1,710,074–61,041,603 a1)	Greenhouse test/isolate *Mont1*	F6:13 (50)	8.8% FDS, 2.3–33.3% RRS	PI 438489B	Abdelmajid et al. (2012), Chang et al. (2018)
		*SDS2-3, SDS3-4, SDS QTL 2G*	OE04_450_	–	Field test (IL, US)	F5:11 (100)	16% DI, 20% DS	Forrest	Chang et al. (1996), Chang et al. (2018)
		*G-QTL-4*	Satt010, Satt324, OEO2_1000_	(5,927,346 a2)	Field test (IL, US)	RIL (100)	11.5–24.3% DI, 23.3% DS, 10.4–28.4% DX	Forrest	Abdelmajid et al. (2007), Chang et al. (2018)
		*-*	Satt324—Satt594	(5,927,346–22,375,830 a2)	Field test (IL, US)	F5:14 (92)	35% DI	Forrest	Yuan et al. (2012)
*qRfv18-03*	MLG G (Chr. 18)	*SDS14-2*	BARC-024251–04,812	(59,472,567 a1)	Greenhouse test/isolate *Somerset #1A*	Ancestral lines, advanced breeding lines, cultivars, and landraces (282)	–	–	Bao et al. (2015)
		*qSDS18-1*	ss249931277—ss249984976	(58.29–61.89 Mb a2)	Field test (MI, US)	F4 derived (153)	7.9–15.8%	U01-390489	Tan et al. (2019)
		*qPY18-1*	ss249931277—ss249984976	(58.29–61.89 Mb a2)	Field test (MI, US)	F4 derived (153)	26.71% plot yield	E07080	Tan et al. (2019)
		*qFvC18-1*	ss249931277—ss249953873	(58.29–59.83 Mb a2)	Field test (MI, US)	F4 derived (153)	8.42% pathogen content in root	U01-390489	Tan et al. (2019)
		*qRDW18-1*	ss249942583—ss249953873	(59.07–59.82 Mb a2)	Field test (MI, US)	F4 derived (153)	17.9–20.9% root dry weight	E07080	Tan et al. (2019)
*qRfv19-01*	MLG L (Chr. 19)	*SDS9-1*	Sat_099	(43,727,029 a2)	Greenhouse test/Strain *ST-90*	F7:14 (284)	7% DS	Minsoy'	Njiti and Lightfoot (2006)
		*SDS18-2, SDS-2*	BARC-047496–12,943—BARC-029419–06,181; Satt678–Satt664	(43,023,466–46,730,376 a2)	Growth chamber/isolates *Clinton 1B* and *Scott F2II 1a*	F7:8 (200)	16%	LS94-3207	Swaminathan et al. (2018)
		*SDS13-3, qFDS002-03*	ss107913933—ss107929955	42.0–49.9 cM (41,343,324—47,114,567 a1)	Greenhouse test/isolate *Mont1*	F6:13 (50)	6–17.7% FDS	PI 438489B	Abdelmajid et al. (2012)
		*SDS14-13, qDX018*	ss250232030—ss250233870	0.0–0.70 cM	Field test (IL, US)	F5:7 (94)	0.01% DX	-	Anderson et al. (2015)
		*-*	Satt166—Satt448	(42,119,600–42,616,473 a2)	Field test (IL, US)	F5 derived (91)	14%	Ripley	de Farias Neto et al. (2007)
*qRfv20-01*	MLG I (Chr. 20)	*qRfs5*	Satt354, Satt270	(35,362,576 a2)	Greenhouse test/isolates *Clinton1b* and *Scott*	F2:3 (321)	8.2–11.5% DI, 12.8% DS, 12.4% DX	Essex	Luckew et al. (2013)
		*SDS7-6, qRfs5*	Satt354	–	Field test (IL, US)	F5:13 (100); F2:3 (321)	11.5% DI	Essex	Iqbal. et al. (2001), Luckew et al. (2013)
		*SDS15-9*	BARC-020245–04,514—BARC-038869–07,364	50.11–63.33 cM (35,312,272—37,595,955 a2)	Growth chamber/isolates *Clinton 1B, Scott F2II 1a* and *Scott B2*	F7 derived RIL (200)	6.3% Root feeding	LS94-3207	Swaminathan et al. (2016)
*qRfv20-02*	MLG I (Chr. 20)	*SDS16-4*	BARC-052017–11,314—BARC-057793–14,926	22.84–35.34 cM (2,103,067 -28,472,273 a2)	Growth chamber/isolates *Clinton 1B, Scott F2II 1a* and *Scott B2*	F7 derived RIL (200)	15% Root feeding	LS98-0582	Swaminathan et al. (2016)
		*qSDS20-1*	ss250304625—ss250327854	(1.06–3.76 Mb a1)	Field test (MI, US)	F4 derived (153)	9.8%	E07080	Tan et al. (2019)
		*SDS disease index 21–3*	Gm20_2954372_G_T—Gm20_30048849_G_T	(2,947,656—31,195,048 a2)	Greenhouse test/isolate *LL0009* (NE305)	F2:3 (200)	20.0% DX	Spencer	Luckew et al. (2017)
		*SDS18-1, SDS-1*	BARC-054889–12,193—BARC-041129–07,912; Satt700–Satt496	(12,117,394—33,590,931 a2)	Growth chamber/isolates *Clinton 1B* and *Scott F2II 1a*	F7:8 (200)	11%	A95-684043	Swaminathan et al. (2018)
		*SDS17-1, SDS-4*	BARC-057793–14,926, Satt127—Sat_268	(12,169,135–35,176,184 a1)	Growth chamber/isolates *Clinton 1B* and *Scott F2II 1a*	F7:8 (200)	7.6%	LS98-0582	Swaminathan et al. (2018)
*qRfv20-3*	MLG I (Chr. 20)	*-*	Sat_299	(43,634,534 a2)	Greenhouse test/isolate *FSG-1*	F4 derived (96)	11%	PI 567374	de Farias Neto et al. (2007)
		*SDS15-5*	BARC-038869–07,364—BARC-059937–16,229	63.33–113.76 cM (37,595,955—45,134,969 a2)	Growth chamber/isolates *Clinton 1B, Scott F2II 1a* and *Scott B2*	F7 derived RIL (200)	5.9% Stem cut	LS94-3207	Swaminathan et al. (2016)

^a^Locus name given in this study, if the physical positions of QTLs overlap each other in at least two independent studies. For example, *qRfv02-01* means the 1st (01) validated quantitative (q) resistance (R) to *Fusarium virguliforme (fv)* on Chr. 2 (02)

^b^Marker position (bp) based on the *Glycine max* genome assembly version *Gmax1.01* (a1), or *Gmax2.0* (a2), only starting position is shown for SSR markers

^c^Phenotypic variations explained by the molecular markers

^d^FDS: foliar disease severity

^e^DI: disease incidence

^f^DAI: days after inoculation

^g^RRS: root rot severity

^h^DX: disease index

^i^DS: disease severity

^j^IS: infection severity (root)

In addition to SDS, other *Fusarium* spp. pathogens (such as *F. redolens*, *F. proliferatum*, *F. oxysporum*, *F. equiseti*, *F. acuminatum*, *F. moniliforme*, *F. graminearum*, *F. semitectum*, *F. chlamydosporum*, *F. compactum*, *F. merimoides*, *F. roseum*, *F. tricinctum, F. avenaceum,* and *F. sporotrichioides*) can also infect soybean, causing wilt, damping-off, and root rot (Arias et al. 2013). Of these *Fusarium* spp., *F. graminearum* was highly aggressive (root rot severity > 90%), causing seed rot and seedling damping-off in South America, Canada, and the USA (Pioli et al. 2004; Broders et al. 2007; Xue et al. 2007; Ellis et al. 2013a; Arias et al. 2013; Cheng et al. 2017a). Horizontal resistance is the only type of resistance so far identified for *F. graminearum*. Since the first report of five QTLs from ‘Conrad’ and ‘Sloan’, a total of thirty QTLs have been identified, accounting for 3.1–40.2% of phenotypic variations on 13 soybean chromosomes (Table 9). Based on the physical locations of the tightly linked or flanking markers, five loci can be validated from two or more QTL mapping or GWA studies, including *qRfg08-01* (17.2–47.4 Mb) and *qRfg08-02* (4.0–9.2 Mb) on Chr. 8, *qRfg13-01* (11.1–39.3 Mb) on Chr. 13, and *qRfg19-01* (47.5–47.8 Mb) and *qRfg19-02* (9.2–41.3 Mb) on Chr. 19 (Table 9). These QTLs can be of higher interest to develop resistant soybean varieties against *F*. *graminearum.*

**Table 9:** Soybean loci conferring resistance to *Fusarium graminearum*

MLG (Chr.)	Locus name^a^	Other name	Tightly linked/flanking markers	Marker position cM (bp)^b^	Testing methods/Resistance spectrum	Population type (size)	PVE^c^	Donor source/allele	References
MLG D1b (Chr. 2)	–	–	rs33907639	(33,907,639 a2)	Rolled towel assay/isolate *L09*	Landraces and elite cultivars from the Chinese National Soybean GeneBank(314)	14%	Allele C	Zhang et al. (2019)
	–	*qFG-4*	Satt600–Satt611	(28,903,021–29,355,267 a2)	Rolled towel assay/isolate *L09*	F2:14 (140)	7.7%	Conrad	Zhang et al. (2020)
MLG C1 (Chr. 4)	–	–	rs52044814	(52,044,814 a2)	Rolled towel assay/isolate *L09*	Landraces and elite cultivars from the Chinese National Soybean GeneBank(314)	14%	Allele T	Zhang et al. (2019)
	–	–	rs658576	(658,576 a2)	Rolled towel assay/isolate *L09*	Landraces and elite cultivars from the Chinese National Soybean GeneBank(314)	6%	Allele T	Zhang et al. (2019)
MLG A1 (Chr. 5)	–	–	rs29240006	(29,240,006 a2)	Rolled towel assay/isolate *L09*	Landraces and elite cultivars from the Chinese National Soybean GeneBank(314)	14%	Allele C	Zhang et al. (2019)
	–	–	rs5676224	(5,676,224 a2)	Rolled towel assay/isolate *L09*	Landraces and elite cultivars from the Chinese National Soybean GeneBank(314)	14%	Allele A	Zhang et al. (2019)
MLG C2 (Chr. 6)	–	–	ss715593740–ss715593784	(17,401,316–18,230,296) a2)	Rolled towel assay/isolate *Fay11*	F7:10 (184)	8.1%	PI 567301B	Acharya et al. (2015)
	–	*qRfg_Gm06*	BARC-042161–08,193	(19,857,954–20,280,838 a2)	Rolled towel assay/isolate *Fay11*	F6:7 (241)	40.2%	PI 567516C	Cheng et al. (2017a)
	–	–	rs9479021	(9,479,021 a2)	Rolled towel assay/isolate *L09*	Landraces and elite cultivars from the Chinese National Soybean GeneBank(314)	15%	Allele G	Zhang et al. (2019)
	–	*qFG-1*	Satt134–Satt365	111.68–112.83 cM*	Rolled towel assay/isolate *L09*	F2:14 (140)	5.4%	Hefeng25	Zhang et al. (2020)
MLG M (Chr. 7)	–	–	rs42503759	(42,503,759 a2)	Rolled towel assay/isolate *L09*	Landraces and elite cultivars from the Chinese National Soybean GeneBank(314)	13%	Allele C	Zhang et al. (2019)
MLG A2 (Chr. 8)	*qRfg08-01*	–	BARC_051847_11270	0.0–10.8 cM (35,856,368 a1)	Rolled towel assay/isolate *Fay11*	F6:8 (262)	9.2%	Conrad	Ellis et al. (2013a)
		*qFG-3*	Satt233 ‐ Satt538	(17,232,172–47,395,378 a2)	Rolled towel assay/isolate *L09*	F2:14 (140)	10.9%	Hefeng25	Zhang et al. (2020)
	*qRfg08-02*	–	Sat_157–ss715602786	(8,353,754—8,657,875 a2)	Rolled towel assay/isolate *Fay11*	F7:10 (184)	38.5%	PI 567301B	Acharya et al. (2015), Million et al. (2019)
		*qFG-2*	Sat_215 ‐ Sat_406	(3,993,698—9,204,446 a2)	Rolled towel assay/isolate *L09*	F2:14 (140)	24.5%	Hefeng25	Zhang et al. (2020)
MLG O (Chr. 10)	–	–	rs13411695	(13,411,695 a2)	Rolled towel assay/isolate *L09*	Landraces and elite cultivars from the Chinese National Soybean GeneBank(314)	12%	Allele G	Zhang et al. (2019)
MLG F (Chr. 13)	–	–	FLOWER_COLOR W1/w1 locus	21.0–23.8 cM	Rolled towel assay/isolate *Fay11*	F6:8 (262)	5.1%	Conrad	Ellis et al. (2013a)
	*qRfg13-01*	–	BARC_2.0_Gm13_16926707	(16,926,707 a2)	Rolled towel assay/isolate *Fay11*	F9:11 (316)	3.1%	Conrad	Stasko et al (2016)
		*qFG-5*	Satt554–Sat_387	(11,101,819–39,252,658 a2)	Rolled towel assay/isolate *L09*	F2:14 (140)	11.4%	Conrad	Zhang et al. (2020)
MLG B2 (Chr. 14)	–	–	BARC_2.0_Gm14_2523881	(2,523,881 a2)	Rolled towel assay/isolate *Fay11*	F9:11 (316)	4.8%	Sloan	Stasko et al (2016)
MLG E (Chr. 15)	–	–	BARC_025663_049888	0.0–19.0 cM	Rolled towel assay/isolate *Fay11*	F6:8 (262)	7.2%	Conrad	Ellis et al. (2013a)
MLG J (Chr. 16)	–	–	Satt693	12.2–21.9 cM (6,325,509 a2)	Rolled towel assay/isolate *Fay11*	F6:8 (262)	5.2%	Conrad	Ellis et al. (2013a)
	–	*qFG-6*	Satt380–Satt183	(25,456,677–26,823,650 a2)	Rolled towel assay/isolate *L09*	F2:14 (140)	5.0%	Conrad	Zhang et al. (2020)
MLG D2 (Chr. 17)	–	–	rs21473423	(21,473,423 a2)	Rolled towel assay /isolate *L09*	Landraces and elite cultivars from the Chinese National Soybean GeneBank(314)	13%	Allele G	Zhang et al. (2019)
	–	–	rs7363671	(7,363,671 a2)	Rolled towel assay/isolate *L09*	Landraces and elite cultivars from the Chinese National Soybean GeneBank(314)	15%	Allele G	Zhang et al. (2019)
MLG L (Chr. 19)	*qRfg19-01*	–	BARCSOYSSR_19_1452	31.7–40.3 cM (47,528,116 a2)	Rolled towel assay/isolate *Fay11*	F6:8 (262)	3.6%	Sloan	Ellis et al. (2013a)
		*QTL 19–2*	BARC_2.0_Gm19_47784141	(47,784,141 a2)	Rolled towel assay/isolate *Fay11*	F9:11 (316)	8.6%	Sloan	Stasko et al (2016)
	*qRfg19-02*	–	rs38240023	(38,240,023 a2)	Rolled towel assay/isolate *L09*	Landraces and elite cultivars from the Chinese National Soybean GeneBank(314)	13%	Allele G	Zhang et al. (2019)
		*qFG-7*	Sct_010–Satt652	(9,175,726–41,380,304 a2)	Rolled towel assay/isolate *L09*	F2:14 (140)	8.7%	Conrad	Zhang et al. (2020)
	–	–	rs42918129	(42,918,129 a2)	Rolled towel assay/isolate *L09*	Landraces and elite cultivars from the Chinese National Soybean GeneBank(314)	13%	Allele C	Zhang et al. (2019)

^a^Locus name given in this study, if the physical positions of QTLs overlap each other in at least two independent studies. For example, *Rfg08-02* means the 2nd (02) validated quantitative (*q*) resistance (R) to *Fusarium graminearum (fg)* on Chr. 8

^b^Marker position (bp) based on the *Glycine max* genome assembly version *Gmax1.01* (a1), or *Gmax2.0* (a2), only starting position is shown for SSR markers

^c^Phenotypic variations explained by the molecular markers

## Stem canker/Phomopsis seed decay

The *Diaporthe/Phomopsis* complex, the genus *Diaporthe* Nitschke (asexual morph *Phomopsis*) (Sacc.) comprises several species of fungi causing important diseases in soybean: northern and southern stem canker, *Diaporthe* seed decay, and pod and stem blight (Santos et al. 2011). This complex is dispersed worldwide resulting in greater yield losses in soybean than any other single fungal pathogen (Sinclair 1993). Phomopsis seed decay (PSD) is mainly caused by *Phomopsis longicolla* (*D. longicolla*), while soybean stem canker (SSC) is primarily caused by two different species, *D. aspalathi* (E. Jansen, Castl. & Crous) (syn. *Diaporthe phaseolorum* var. *meridionalis*) and *D. caulivora* (Athow & Caldwell) J.M. Santos, Vrandecic & A.J.L. Phillips (syn. *Diaporthe phaseolorum* var. *caulivora*) (Fernández et al. 1999; Pioli et al. 2003; Santos et al. 2011; Udayanga et al. 2015) and *D. sojae* is the cause of pod and stem blight (Udayanga et al. 2015). Recently*, D. gulyae, D. bacilloides,* and *D. ueckerae* have also been associated with soybean diseases (Mathew et al. 2018; Petrović et al. 2021).

Northern stem canker (caused by *D. caulivora*) was first observed in the late 1940s in the northern USA (Athow and Caldwell 1954) and resulted in severe yield losses in the mid-1950s. Hildebrand (1956) developed a greenhouse assay for stem canker which involved growing the fungus on sterilized wooden toothpicks and inserting the toothpicks into the soybean stems. Susceptible cultivars develop a canker and die, while resistant cultivars do not develop a canker symptom. Hildebrand noted that seedlings of ‘Hawkeye’ and ‘Blackhawk’ appeared resistant when inoculated, became susceptible at mid-stage, and then grew increasingly resistant as the plants matured. In the late 1990s, northern stem canker emerged as an important disease in the northern USA and Ontario, Canada (Wrather et al. 2003a). Thickett et al. (2007) developed a cut stem assay by placing inoculum on the cut surface of seedling stems which were severed above the unifoliate leaves. After two weeks, the length of the lesions was longer on the susceptible cultivars, and results agreed with field observations. To date, little has been done to elucidate the genetic resistance to *D. caulivora*.

Southern stem canker (caused by *D. aspalathi*) was first reported in the 1970s causing an estimated loss of $37 million in 1983 (Backman et al. 1985; Weaver et al. 1988). Initially identified as *D. phaseolorum* var*. caulivora*, southern isolates were noticeably different from northern isolates in culture (McGee and Biddle 1987). The name of the fungus was changed to *D. phaseolorum* var. *meridionalis* and is now *D. aspalathi* (Rensburg et al. 2006; Santos et al. 2011). Southern stem canker begins as a canker on the lower stem during mid-reproductive development (Weaver et al. 1988; Rupe 2016). The canker grows on one side of the stem but does not girdle the stem producing a toxin that results in distinctive foliar symptoms before prematurely killing the plant. Consistent cultivar reactions to southern stem canker were observed in the field, but the occurrence of the disease varied from year to year. Keeling (1985) reported that cultivar responses to inoculating 10-day-old seedlings with infested toothpicks were in good agreement with field ratings. The toothpick inoculation method was later used on 60-day-old field plants and compared to inoculating the plant with ascospores. Both methods consistently produced stem canker symptoms and were able to identify cultivar responses from very susceptible to very resistant (Keeling 1988). Single dominant resistance genes to southern stem canker were reported from the cultivar ‘Tracy-M’, *Rdc1* and *Rdc2* (later renamed *Rdm1* and *Rdm2*, respectively) (Kilen and Hartwig 1987), in ‘Crockett’, *Rdc3* (later renamed *Rdm3*), and in ‘Dowling’, *Rdc4* (later renamed *Rdm4*) (Bowers et al. 1993) (Table 10). *Rdc4* was also found in the cultivar ‘Hutcheson’ (Tyler 1996). Initially, all these genes appeared to be equally effective against all isolates of *D. aspalathi* (Keeling, 1988), but a report from Argentina isolates of *D. aspalathi* were found virulent on one or more of each of these genes (Pioli et al. 2003). Interestingly, they found a number of isolates of *D. aspalathi* that were virulent on lines with *Rdc1* and lines with *Rdc2* but were avirulent on Tracy-M which has both *Rdc1* and *Rdc2*. Moderate levels of resistance to southern stem canker have been reported from the field and greenhouse inoculations, but the genetic nature of that resistance has not been explored.

**Table 10:** Soybean loci conferring resistance to stem canker (caused by *Diaporthe aspalathi* and *D. caulivora*) and Phomopsis seed decay (caused by *D. longicolla*)

Causal agent	Locus name	MLG (Chr.)	Tightly linked/flanking markers	Marker position cM (bp)^a^	Testing methods/Resistance spectrum	Population type (size)	PVE^b^	Donor source	References
*Diaporthe aspalathi*	*Rdc1* (*Rdm1*)	–	–	–	Field test	F3 (40)	–	Tracy-M	Kilen and Hartwig (1987)
	*Rdc2* (*Rdm2*)	–	–	–	Field test	F3 (40)	–	Tracy-M	Kilen and Hartwig (1987)
	*Rdc3* (*Rdm3*)	–	–	–	Greenhouse test	F2 (200–600)	–	Crockett and Dowling	Bowers et al. (1993)
	*Rdc4* (*Rdm4*)	MLG A2 (Chr. 8)	–	–	Greenhouse test	F2 (200–600)	–	Hutcheson and Dowling	Bowers et al. (1993), Tyler (1996)
	*Rdm5*	MLG A2 (Chr. 8)	–	12.4 cM	Greenhouse test	F2:3 (105)	–	Hutcheson	Chiesa et al. (2009)
	*Rdm*_*MJ19RR*_	MLG C2 (Chr. 6)	Satt433	13.3 cM (47,516,523)	Greenhouse test	F2 (147)	–	MJ19RR	Gilli et al. (2020)
	GBSRdm370	MLG B2 (Chr. 14)	–	(1,744,370)	Greenhouse test	Accessions	–	–	Dos Santos et al. (2019)
	GBSRdm556	MLG B2 (Chr. 14)	–	(1,725,556)	Greenhouse test	Accessions	–	–	Dos Santos et al. (2019)
	GBSRdm287	MLG B2 (Chr. 14)	–	(1,710,287)	Greenhouse test	Accessions	–	–	Dos Santos et al. (2019)
	GBSRdm224	MLG B2 (Chr. 14)	–	(1,986,224)	Greenhouse test	Accessions	–	–	Dos Santos et al. (2019)
	GBSRdm562	MLG B2 (Chr. 14)	–	(1,740,562)	Greenhouse test	Accessions	–	–	Dos Santos et al. (2019)
	GBSRdm793	MLG B2 (Chr. 14)	–	(1,768,793)	Greenhouse test	Accessions	–	–	Dos Santos et al. (2019)
	GBSRdm339	MLG B2 (Chr. 14)	–	(1,921,339)	Greenhouse test	Accessions	–	–	Dos Santos et al. (2019)
	GBSRdm374	MLG B2 (Chr. 14)	–	(1,921,374)	Greenhouse test	Accessions	–	–	Dos Santos et al. (2019)
	GBSRdm219	MLG B2 (Chr. 14)	–	(1,795,219)	Greenhouse test	Accessions	–	–	Dos Santos et al. (2019)
	GBSRdm204	MLG B2 (Chr. 14)	–	(1,751,204)	Greenhouse test	Accessions	–	–	Dos Santos et al. (2019)
	GBSRdm516	MLG B2 (Chr. 14)	–	(1,612,516)	Greenhouse test	Accessions	–	–	Dos Santos et al. (2019)
	GBSRdm964	MLG B2 (Chr. 14)	–	(1,850,964)	Greenhouse test	Accessions	–	–	Dos Santos et al. (2019)
	GBSRdm114	MLG B2 (Chr. 14)	–	(1,851,114)	Greenhouse test	Accessions	–	–	Dos Santos et al. (2019)
	GBSRdm450	MLG B2 (Chr. 14)	–	(1,612,450)	Greenhouse test	Accessions	–	–	Dos Santos et al. (2019)
	GBSRdm397	MLG B2 (Chr. 14)	–	(1,612,397)	Greenhouse test	Accessions	–	–	Dos Santos et al. (2019)
	GBSRdm518	MLG B2 (Chr. 14)	–	(1,744,518)	Greenhouse test	Accessions	–	–	Dos Santos et al. (2019)
	GBSRdm120	MLG B2 (Chr. 14)	–	(1,741,120)	Greenhouse test	Accessions	–	–	Dos Santos et al. (2019)
	GBSRdm712	MLG B2 (Chr. 14)	–	(1,581,712)	Greenhouse test	Accessions	–	–	Dos Santos et al. (2019)
	GBSRdm875	MLG B2 (Chr. 14)	–	(1,581,875)	Greenhouse teste	Accessions	–	–	Dos Santos et al. (2019)
*Diaporthe longicolla*	*PSD 6–1*	MLG C2 (Chr. 6)	Satt100—Satt460	110.8 cM (31,490,622–44,049,891)	Greenhouse test	F8 (124)	46.3%	Taekwangkong	Sun et al. (2013)
	*PSD-10–2*	MLG O (Chr. 10)	Sat_038—Satt243	85.8 cM (46,052,103–46,657,863)	Greenhouse test	F8 (124)	14.1%	SS2-2	Sun et al. (2013)
	–	MLG F (Chr. 13)	Sat_317 and Sat_120	5.9–12.7 cM (32,196,800)	Field test	F2 (140)	–	MO/PSD-0259	Roy and Abney (1988), Ploper et al. (1992), Minor et al. (1993), Zimmerman and Minor (1993), Jackson et al. (2004)
	–	MLG B2 (Chr. 14)	Sat_177 and Sat_342	4.3–15.8 cM (971,657–2,956,930)	Field test	F2 (140)	–	PI 80837	Roy and Abney (1988), Ploper et al. (1992), Minor et al. (1993), Zimmerman and Minor (1993), Jackson (2004)

^a^Marker position (bp) based on the *Glycine max* genome assembly version *Gmax2.0*, only starting position is shown for SSR markers

^b^Phenotypic variations explained bsy the molecular markers

## Phomopsis seed decay

Phomopsis seed decay (PSD) of soybean is the major cause of poor seed quality and significant yield loss in most soybean-growing regions (Sinclair, 1993). PSD is favored by hot and humid environmental conditions and is usually worse with early maturing cultivars planted early in the season. Severe symptoms are shriveled, elongated, or cracked, chalky appearance, but seed infection is usually symptomless. These symptomless infections can result in pre- and post-emergence damping-off (Sinclair 1993; Kulik and Sinclair 1999; Koenning 2010). Resistance to PSD has been reported in PI 82264 (Walters and Caviness 1973), PI 181550 (Athow 1987), the cultivar ‘Delmar’ (Crittenden and Cole 1967; Brown et al. 1987), PI 200501, and ‘Arksoy’ (Ross 1986), and in PI 80837, PI 417479, and PI 360841 (Brown et al. 1987) (Table 10). PI 417,479 was reported to have two dominant genes for resistance to PSD, one located on linkage group F and one on linkage group H (Zimmerman and Minor 1993). The PSD resistant line, ‘MO/PSD-0259’ was developed from PI 417479 (Elmore et al. 1998; Minor et al. 1993). MO/PSD-0259 was used to develop two PSD resistant lines, ‘SS 93–6012’ and ‘SS 93–6181’ (Wrather et al. 2003b). The resistance in PI 80837 was determined to be conferred by a single dominant gene that is different from the one in MO/PSD-0259 (Jackson et al. 2005). A genetic study using a greenhouse inoculation method with progenies derived from a cross between the resistant cultivar ‘Taekwangkong’ and the susceptible cultivar ‘SS2-2’ reported two QTLs associated with PSD resistance which were tightly linked with genes for maturity (Sun et al. 2013). Many PIs in maturity groups III, IV, and V were identified as resistant to PSD across three states (Li et al. 2010a). Resistance to PSD was identified in six commercial cultivars in inoculated and non-inoculated tests (Li et al. 2017a). In a study evaluating the response of PIs to purple seed stain (PSS), nine PIs with resistance to PSS were also resistant to PSD (Li et al. 2019). PI 80837 also has resistance to both PSS and PSD (Jackson et al. 2005, 2006). A cut stem seedling assay similar to that described for inoculations with *D. caulivora* by Thickett et al. (2007) was used with *D. longicolla* (Li 2018). This method gave similar results as field tests. A draft genome sequence for *D. longicolla* has been published (Li et al. 2015a, 2017a), and the glycoside hydrolase subnetwork appears to be important in pathogeneses (Li et al. 2018).

Numerous management practices can be applied to control PSD, including deep tillage, crop rotation with non-legume crops, treating seeds with fungicides, and applying fungicides during pod-fill. To date, the most effective management option is the use of resistant cultivars (Park 1991; Roy et al. 1994; Jackson et al. 2005; Pathan et al. 2009; Mengistu et al. 2010). A report by Sun et al. (2013) identified two QTLs for PSD resistance associated with days to maturity in soybean (Table 10). This was an important discovery because early maturing soybean genotypes are often highly susceptible to PSD due to the weather conditions during pod and seed development. Several screening methods have been used to identify sources of resistance, including those mentioned above for stem canker, seed plate assay (Li et al. 2011), and cut-stem inoculation method.

## Sclerotinia stem rot

Sclerotinia stem rot (or white mold), caused by *Sclerotinia sclerotiorum* (Lib.), can cause significant yield losses in soybean and overall reduction of seed quality in North Central USA and northeastern China under conducive cool and wet weather conditions (Hoffman et al. 1998; Kurle et al. 2001; Peltier et al. 2012; Sun et al. 2020). For example, in 2004 and 2009, Sclerotinia stem rot caused yield losses of 1.63 and 1.61 million Mt, respectively, in the USA alone (Peltier et al. 2012). More recently, over 1.08 million Mt of production losses were recorded in 2014 in the North Central USA and Ontario, Canada (Allen et al. 2017). The disease steadily ranked among the top 10 most destructive diseases associated with yield losses in the northern USA and Ontario, Canada (Allen et al. 2017).

Horizontal resistance is the only type of soybean resistance identified for Sclerotinia stem rot. The first report for horizontal resistance identified three minor QTLs (explaining 6.5–9.6% of phenotypic variations) on linkage groups M, K, and C2 using a bi-parental population of 152 F3 derived RILs (Kim and Diers 2000). More recently, the assembly of the soybean reference genome and advancements in GWA have enabled more accurate dissection of genomic regions associated with resistance to Sclerotinia stem rot (Schmutz et al. 2010). For example, Bastien et al. (2014) identified four significant markers for resistance, which were located at chromosomes 1, 15, 19, and 20, explaining 6.3–14.5% of phenotypic variations. The locus on Chr. 15 (renamed *qRss15-01* in this review) was further validated in an F4:5 RIL population where significantly shorter lesions were observed for 24 resistant genotypes. In another GWA study, a major locus was identified and validated on Chr. 13 (*Qswm13-1*, and renamed *qRss13-01* in this review), which explained 23.33% of phenotypic variations (Zhao et al. 2015). From 2014 to 2021, a total of nine GWA studies have been published (Bastien et al. 2014; Iquira et al. 2015; Zhao et al. 2015; Wei et al. 2017; Wen et al. 2018; Boudhrioua et al. 2020; Sun et al. 2020; Jing et al. 2021; Zou et al. 2021). Combining the studies of QTL mapping and GWA, 14 loci have been validated from at least two mapping studies (Table 11). The 14 loci were distributed at 11 chromosomes (1, 4, 6, 8, 9, 10, 12, 13, 15, 17, and 19) and contributed as high as 32% of the phenotypic variations. These validated loci may be of high priority for soybean breeders to use for improving partial resistance to Sclerotinia stem rot. In addition to the validated QTLs, more than 200 QTLs have also been identified and may be validated in the future (Supplementary Table 3),

**Table 11:** Validated soybean loci associated with quantitative resistance to Sclerotinia stem rot (caused by *Sclerotinia sclerotiorum*)

MLG (Chr.)	Locus name^a^	Other name	Tightly linked/flanking markers	Marker position cM (bp)^b^	Testing methods/Resistance spectrum	Population type (size)	PVE^c^	Donor	References
MLG D1a (Chr. 1)	*qRss01-01*	–	–	(5,594,597 a2)	Cotton pad method/strain *NB-5*	Canada breeding lines (127)	32%	Allele T	Boudhrioua et al. (2020)
		–	–	(5,594,597 a2)	Cotton pad method/strain *NB-5*	F6:8 (47)	–	Maple Donovan	Boudhrioua et al. (2020)
	*qRss01-02*	–	S1_36,783,951, S1_36,497,505, S1_35,474,053, S1_35,045,463, S1_36,006,734, S1_36,045,483, S1_35,152,187	(35,045,463—36,783,951 a1)	Greenhouse test/isolate *Jatai*	Brazil breeding lines (275)	2.3–4.0%	–	Wei et al. (2017)
		*Qswm1-1*	BARCSOYSSR01_0884, BARCSOYSSR01_1102	89.21–91.19 cM (35,860,562–43,694,885 a2)	Greenhouse test	F5:10 (128)	7.9%	–	Zhao et al. (2015)
MLG C1 (Chr. 4)	*qRss04-01*	*Locus3*	ss715587925 and ss715588278	(42,372,944–46,104,694 a2)	Field test (MI, US), greenhouse test/isolate *105HT*	Improved lines (962)	5.5–5.6%	–	Wen et al. (2018)
		–	Gm04:43,486,259	(43,486,259 a2)	Cut stem method	China accessions (185)	8%	Allele T	Jing et al. (2021)
MLG C2 (Chr. 6)	*qRss06-01*	*Sclero 7–1*	Sat_238 and Satt708	(40,461,941—43,990,660 a2)	Field inoculation (QC, Canada)/strain *NB-5*	F4 derived RILs (180)	18.9–23.6%	Maple Donovan	Huynh et al. (2010)
		–	Gm06:43,719,111	(43,719,111 a2)	Cut stem method	China accessions (185)	8–9%	Allele A	Jing et al. (2021)
MLG A2 (Chr. 8)	*qRss08-01*	–	Sat_129, Satt329 (Patent)	(14,660,743–21,117,799 a2)	Greenhouse	–	12.0%	P^S^	Han et al. (2007)
		*Sclero 10–2*	Sat_199	70.95 cM (15,071,890 a2)	Field inoculation (IA and WI, US) and greenhouse test	PI (66), breeding lines (35), F4:6 (392)	15.8%	–	Kandel et al. (2018)
		*Qsp-3*	Satt525 and Satt233	(17,010,941–17,232,172 a2)	Greenhouse test	F5:6 (149)	8.0%	MapleArrow	Li et al. (2010e)
		*QTL3, Sclero 2–3*	Satt233	(17,232,172 a2)	Detached leaf method/isolate *143*	F5 (100)	4–10%	Corsoy79	Arahana et al. (2001)
		*Sclero 9–1*	Satt233-Satt327	(17,232,172–20,468,620 a2)	Field inoculation (MI, US) and greenhouse inoculation/*isolate HT105*	F2:3 (94), F2:4 (94), F2:5 (94)	10.4%	IA2053	Guo et al. (2008)
		–	ss715599948	(17,490,619 a2)	Field inoculation (IA, US)	USDA germplasm collection (474)	–	–	Moellers et al. (2017)
		*Qswm8-1*	BARCSOYSSR08_1160, BARCSOYSSR08_1127	55.89–59.74 cM (20,468,640–21,633,276 a2)	Greenhouse test	F5:10 (128)	11.3%	–	Zhao et al. (2015)
MLG K (Chr. 9)	*qRss09-01*	*QTL19, Sclero 2–16; Sclero 3–11; Sclero 4–7; Sclero 5–10*	Satt273	(38,799,271 a2)	Detached leaf method/isolate *143*	F5 (400)	4–10%	Corsoy79, Dassel, S19-90, Williams82	Arahana et al. (2001)
		*Sclero 8–3*	Satt273	(38,799,271 a2)	Greenhouse test/isolate *105HT*	F4:5 (155)	5.5%	PI 194639	Vuong et al. (2008)
MLG O (Chr. 10)	*qRss10-01*	*QTL26, Sclero 2–22; Sclero 3–17; Sclero 5–14; Sclero 6–11*	Satt478	(39,108,281 a2)	Detached leaf method/isolate *143*	F5 (400)	4–10%	Corsoy79, Dassel, S19-90, Vinton81	Arahana et al. (2001)
		*Sclero 10–3*	Satt478	66.01 cM (39,108,281a2)	Field inoculation (IA and WI, US) and greenhouse test	PI (66), breeding lines (35), F4:6 (392)	2.5%	–	Kandel et al. (2018)
	*qRss10-02*	*Sclero 10–5*	Satt243	107.30 cM (46,657,863 a2)	Field inoculation (IA and WI, US) and greenhouse test	PI (66), breeding lines (35), F4:6 (392)	2.0%	–	Kandel et al. (2018)
		–	ss715607699	(47,626,066 a2)	Greenhouse test	USDA germplasm collection (474)	–	–	Moellers et al. (2017)
		*QTL28, Sclero 2–24; Sclero 3–19; Sclero 4–11; Sclero 5–16; Sclero 6–13*	Satt243, Sat_108, and Sat_109	(46,657,863–48,199,089 a2)	Detached leaf method/isolate *143*	F5 (500)	4–10%	Corsoy79, Dassel, S19-90, Vinton81, Williams82	Arahana et al. (2001)
MLG H (Chr. 12)	*qRss12-01*	*qLLS12-1*	Block2877-Block2897	1.90 cM (35,766,547–38,843,925 a2)	Greenhouse test	F5:20 (149)	5.2%	Maple Arrow	Zou et al. (2021)
		–	Gm12:36,426,007	(36,426,007 a2)	Detached leaf method	China landraces (38), elite cultivars (147)	7.0–8.8%	Allele C	Sun et al. (2020)
MLG F (Chr. 13)	*qRss13-01*	*Qswm13-1*	BARCSOYSSR13_0114-BARCSOYSSR13_0197	124.11–127.86 cM (2,195,422–3,999,858 a1)	Greenhouse test	F5:10 (128)	23.3–23.6%	–	Zhao et al. (2015)
		–	rs3296245	(3,296,245 a1)	Greenhouse test	China core germplasm collection (330)	9.5–22.6%	Allele A	Zhao et al. (2015)
		–	rs3296576	(3,296,576 a1)	Greenhouse test	China core germplasm collection (330)	9.5–23.6%	Allele C	Zhao et al. (2015)
		–	rs3446126	(3,446,126 a1)	Greenhouse test	China core germplasm collection (330)	7.0–17.2%	Allele G	Zhao et al. (2015)
	*qRss13-02*	*qLLS13-1*	Block2994-Block2997	174.00 cM (17,233,137–17,548,123 a2)	Greenhouse test	F5:20 (149)	5.3%	Maple Arrow	Zou et al. (2021)
		*qDRS13-1*	Block2996-Block2997	174.00 cM (17,417,745–17,548,123 a2)	Greenhouse test	F5:20 (149)	21.1%	Maple Arrow	Zou et al. (2021)
		–	–	(17,472,342 a2)	Greenhouse test	CNSGB germplasm (261)	–	Allele G	Zou et al. (2021)
MLG E (Chr. 15)	*qRss15-01*	–	–	(13,339,206–13,929,317 a1)	Greenhouse test/strain *NB-5*	Breeding lines (130)	14.5%	–	Bastien et al. (2014)
		–	–	(13,339,206–13,929,317 a1)	Greenhouse test/strain *NB-5*	F4:6 (48)	–	PR918827	Bastien et al. (2014)
		–	–	(13,665,369 a2)	Cotton pad method/strain *NB-5*	Canada breeding lines (127)	15%	Allele A	Boudhrioua et al. (2020)
MLG D2 (Chr. 17)	*qRss17-01*	*QTL10, Sclero 3–6*	Satt154	(9,576,644 a2)	Detached leaf method/isolate *143*	F5 (200)	4–10%	Williams82	Arahana et al. (2001)
		*Sclero 10–8*	Satt154	46.76 cM (9,576,644 a2)	Field inoculation (IA and WI, US) and greenhouse test	PI (66), breeding lines (35), F4:6 (392)	4.0%	–	Kandel et al. (2018)
MLG L (Chr. 19)	*qRss19-01*	–	Satt523, SLS2C.F20 (Patent)	(7,127,430 a2)	Greenhouse test	–	16.0%	–	Han et al. (2007)
		*Sclero 10–10*	Satt523	25.56 cM (7,127,430 a2)	Field inoculation (IA and WI, US) and greenhouse test	PI (66), breeding lines (35), F4:6 (392)	8.5%	–	Kandel et al. (2018)

^a^Locus name given in this study, if the physical positions of QTLs overlap each other in at least two independent studies. For example, *qRss10-02* means the 2nd (02) validated quantitative (q) resistance (R) to *Sclerotinia sclerotiorum (ss)* on Chr. 10

^b^Marker position (bp) based on the *Glycine max* genome assembly version *Gmax1.01* (a1), or *Gmax2.0* (a2), only starting position is shown for SSR markers

^c^Phenotypic variations explained by the molecular markers

## Soybean rust

Asian soybean rust (ASR) caused by *Phakopsora pachyrhizi* (Sydow. & Sydow.) is one of the most destructive diseases in soybean. When environmental conditions are conducive for disease development, ASR spreads fast, causing severe crop damage, leading to significant seed quality reduction and yield losses of as much as 80% (Yorinori et al. 2005). Losses vary upon weather conditions, genotype, and the maturity stage at the time of infection (Wang and Hartman 1992) and are mainly attributed to premature leaf fall, reduced green leaf area in the canopy, reduced dry matter accumulation and reduced harvest index (Kumudini et al. 2008). Soybean rust can also be caused by *P. meibomiae*, which resembles *P. pachyrhizi* in both symptoms and spore appearance. Yet the rust caused by *P. meibomiae* occurs mainly in South and Central America and causes little damage on soybean. This review will be focused on ASR.

ASR is primarily diagnosed with a magnifying glass or microscope, but the polymerase chain reaction (PCR) reaction is also useful when sporulating pustules are not visible (Frederick et al. 2002). The key feature of ASR is the appearance of uredinia and urediniospores. Therefore, it is recommended that infected leaf samples be incubated in a humid chamber and left overnight to enhance rust development and sporulation for accurate diagnosis.

Many management strategies have been proposed to control ASR, including cultural practices, nutrition management, biological and fungicide applications, and host genetic resistance (Tadesse 2019). The application of fungicides is the preferred management tool used by farmers in regions where ASR is prevalent, but it increases production costs and environmental footprint. Since host plant resistance appears as an affordable method for managing ASR, considerable efforts have been directed toward screening soybean germplasm for resistance to *P. pachyrhizi* and the development of resistant cultivars.

### Resistance to ASR

Screening for reaction to ASR can be carried out in the field, in locations where the presence of inoculum and environmental conditions are appropriate for disease development, or in the greenhouse with controlled inoculations and incubation at high relative humidity (Childs et al. 2018a). In the latter case, it is necessary to collect and maintain the *P. pachyrhizi* isolates to be used in the inoculations. Spores can be stored in sub-zero freezers, but, as an obligate parasite, inoculum must be produced on living soybean seedlings.

Resistance to ASR in soybean plants is evaluated based on the presence or absence of lesions, color of the lesions, number of uredinia per lesion, and level of sporulation (Bromfield 1984). More recent studies have evaluated resistance using quantitative traits (Bonde et al. 2006; Walker et al. 2011; 2014). During a compatible interaction in a susceptible soybean plant, abundant sporulation and tan lesions occur, whereas in incompatible interactions (resistance), lesions are reddish-brown (RB) with less sporulation. Immune reactions (IM) have also been observed without visible lesions (Bromfield, 1984).

However, it has been pointed out that the number of uredinia per lesion and the level of sporulation are not necessarily correlated with the color of the lesion (Yamanaka et al. 2015a). Yamanaka et al. (2010) analyzed five traits including lesion color, the number of uredinia per lesion, frequency of lesions that had uredinia, frequency of open uredinia, and level of sporulation, and observed high correlations between all the traits except the color of the lesion. In this sense, Yamanaka et al. (2016) selected the number of uredinia per lesion, the frequency of lesions that had uredinia, and the level of sporulation to assess the degree of resistance.

Resistance or susceptibility studies focus on understanding the defensive response. To date, eight major resistance genes (*Rpp1-7*, *Rpp1-b*) have been mapped (Table 12) (Childs et al. 2018b; Hossain 2019). But these *Rpp* gene-mediated resistances against ASR have been overcome in nature several times. For example, the soybean resistance provided by *Rpp1* and *Rpp3* was defeated by the *P. pachyrhizi* MT isolate only two years after ASR was first detected in Brazil (Pierozzi et al. 2008).

**Table 12:** Soybean loci conferring resistance to soybean rust (caused by *Phakopsora pachyrhizi*)

MLG (Chr.)	Locus/allele name	Tightly linked/flanking markers	Marker position cM (bp)^a^	Testing methods/Resistance spectrum	Population type (size)	PVE^b^	Donor source	References
MLG N (Chr. 03)	*Rpp5*	Sat_275 and Sat_280	40.81–43.45 cM (29,862,641–32,670,432 a2)	Growth chamber/isolate *BRSMS Bacuri*	F2:3 (173)	R gene	PI 200456	Garcia et al. (2008
		Sat_275 and Sat_280	40.81–43.45 cM (29,862,641–32,670,432 a2)	Growth chamber/isolate *BRSMS Bacuri*	F2:3 (177)	R gene	PI 200526	Garcia et al. 2008
		Sat_275 and Sat_280	40.81–43.45 cM (29,862,641–32,670,432 a2)	Growth chamber/isolate *BRSMS Bacuri*	F2:3 (174)	R gene	PI 471904	Garcia et al. (2008)
MLG C2 (Chr. 06)	*Rpp3*	Sat_263 and Sat_238	118.67–117.45 cM (44,738,585–43,990,660 a2)	Growth chamber/isolate *Japanese T1-2*	F2 (86)	70%	PI 416764	Hossain et al. (2015)
		Satt460 and Sat_263	117.76–118.67 cM (44,049,891–44,738,585 a2)	Isolates *AL04-1 (USA), AU79-1 (Australia), BZ01-1 (Brazil), HW94-1 (USA), IN73-1 (India), LA04-1 (USA), PG01-2 (Paraguay), SA01-1 (South Africa), TW72-1 (Taiwan) AND TW80-2 (Taiwan)*	F2:3 (110)	R gene	PI 462312	Hyten et al. (2009)
		Satt079 and Satt307	117.87–121.26 cM (44,503,658 – 46,820,673 a2)	Local isolate (Brzil)	F2:4 (116)	R gene	FT-2 (Brazil)	Brogin (2005)
		Satt 460 and Staga001	117,76–119.84 cM (44,049,891–45,427,175 a2)	Field test/local field isolates	F2:3 (91 and 68)	R gene	PI 567099A	Ray et al. (2011)
		Satt460 and Satt307	117.76–121.26 cM (44,049,891–46,820,673 a2)	Field and Greenhouse test/local field isolate	F6:7 RILs (117)	15–14%	Hyuuga	Monteros et al. (2007)
	–	BARC-023517–05,442 and BARC-040475–07,751	(21,986,774–28,804,685 a2)	Field inoculation (Ha Noi, Vietnam)	F6:7 (250)	11.7%	DT2000 (PI 635999)	Vuong et al. (2016)
	–	BARC-040475–07,751 and BARC-051071–10,973	(21,986,774–22,185,687 a2)	Field tests (FL, US)	F6:7 (250)	8.6%	DT2000 (PI 635999)	Vuong et al. (2016)
	–	Sat_312 and BARC-203517–05,442	(27,940,542–36,131,665 a2)	Field tests (FL, US)	F6:7 (250)	8.4%	DT2000' (PI 635999)	Vuong et al. (2016)
MLG A2 (Chr. 08)	*QTL Asian Soybean Rust 2–1*	Satt409 and Satt429	145.57–162.02 cM (45,106,638—47,217,842 a2)	Greenhouse test/local isolate (Georgia, USA)	F6 (240)	10%	Benning (PI 595645)	Harris et al. (2015)
MLG K (Chr. 09)	*QTL Asian Soybean Rust 2–2*	Satt326 and Sat_363	49,52–50,58 cM (29,967,163–36,143,707 a2)	Greenhouse test/local isolate (Georgia, USA)	F6 (240)	5%	PI 416937	Harris et al. (2015)
MLG F (Chr. 13)	*QTL Asian Soybean Rust 2–3*	Satt490 and Satt554	97,97–111,88 cM (36,699,189–39,252,658 a2)	Greenhouse test/local isolate (Georgia, USA)	F6 (240)	9%	Benning	Harris et al. (2015)
MLG E (Chr. 15)	*QTL Asian Soybean Rust 2–4*	Sat_124 and Satt369	15,86–56,27 cM (11,099,721 a1–49,011,265 a2)	Greenhouse test/local isolate (Georgia, USA)	F6 (240)	17%	PI 416937	Harris et al. (2015)
MLG J (Chr. 16)	*Rpp2*	Satt215 and Sat_361	44.08–44.49 cM (28,944,536–30,478,500 a2)	Growth chamber/isolate *BRSMS Bacuri*	F2:3 (174)	R gene	PI 224270	Garcia et al. (2008)
		Satt620 and Sat_366	52.84 cM (29,205,413–30.404.629 a2)	Growth chamber/isolate *E1-4–12*	F2 (143)	70%	Iyodaizu	Yamanaka et al. (2015a)
MLG G (Chr. 18)	*Rpp1*	Sct_187 and Sat_064	107.11–108.69 cM (60,463,057 a1—56,333,703 a2)	Greenhouse test/isolate *India 73–1*	BC6 F2:3 (126)	R gene	PI 200492	Hyten et al. (2007)
		–	–	Field tests (USA)	Germplasm (576)	R gene	PI 547875 (L85-2378)	Walker et al. (2011)
		Satt191 and Sat_117	96.57–100 cM (58,722,811–58,879,539 a1)	Greenhouse test/local isolates (Brazil)	F2 (160)	R gene	PI 594760B	Garcia et al. (2011)
		Satt191 and Sat_064	96.57–108.69 cM (54,450,956–56,333,703 a2)	Growth chamber/isolate *Japanese T1-2*	F2 (90)	50%	Xiao Jing Huang	Yamanaka et al. (2015a)
		Sct_187 and Sat_064	107.11–108.69 cM (60,463,057 a1–56,333,703 a2)	Growth chamber/isolate *E1-4–12*	F2 (120)	65%	PI 594177 (Himeshirazu)	Yamanaka et al. (2015a)
		Sat_064 and SSR66	108.69 cM (56,333,703 a2)	Growth chamber/isolate *Japanese T1-2*	F2 (117)	60%	PI 587905	Hossain et al. (2015)
		Sat_064	108.69 cM (56,333,703 a2)	Growth chamber/isolate *Japanese T1-2*	F2 (82)	56%	PI 594767A	Hossain et al. (2015)
	*Rpp1? Rpp1-b?*	Satt191 and Sat_064	96.57–108.69 cM (54,450,956–56,333,703 a2)	Field test/isolates *TW72-1 (Taiwan), ZM01-1 (Zimbabwe), IN73-1 India), HW94-1 (Hawai, USA), HW98-1 (Hawai, USA), AU79-1 (Australia), LA04-1 (Lousiana, USA), AL04-3 (Alabama, USA)*	F2:3 (186)	R gene	PI 587886	Ray et al. (2009)
		Satt191 and Sat_372	96.57–107.75 cM (54,450,956 a2)	*Field test/isolates TW72-1 (Taiwan), ZM01-1 (Zimbabwe), IN73-1 India), HW94-1 (Hawai, USA), HW98-1 (Hawai, USA), AU79-1 (Australia), LA04-1 (Lousiana, USA), AL04-3 (Alabama, USA)*	F2:3 (164)	R gene	PI 587880A	Ray et al. (2009)
	*Rpp1-b?*	Sat_064 and AF162283	108.69–87.94 cM (56,333,703 a2–57,436,765 a1)	Growth chamber/isolate *E1-4–12*	F2 (106)	65–67%	PI 587855	Yamanaka et al. (2016)
	*rpp1*	Sat_117 and Sct_187	100–107.11 cM (58,879,539—60,463,057 a1)	Greenhouse test/local isolates (Brazil)	F2 (105)	R gene	PI 594760B	Garcia et al. (2011)
	*Rpp1-b*	Sat_064 Between: BARC-010495–00,656 BARC-014379–01,337	108.69 cM (56,333,703 a2)	Greenhouse test/isolate *ZM01-1*	F3:4 (98)	70%	PI 594538A	Chakraborty et al. (2009)
	*Rpp4*	Satt288 and Satt191	76.76–96.57 cM (51,127,425–54,450,956 a2)	Growth chamber/isolate *BRSMS Bacuri*	F2:3 (175)	R gene	PI 459025	Garcia et al. (2008)
		Satt288 and AF162283	76.76–87.94 cM (51,127,425 a2–57,436,765 a1)	Field collection	F2:3 (80)		PI 459025	Silva et al. (2008)
	*QTL Asian Soybean Rust 1–1*	SSR50 and SSR1859	(60,518,978–60,613,084 a1)	Greenhouse test/isolate *ZM01-1*	F2:3 (100)	70%	PI 561356	Kim et al. (2012)
	*Rpp6*	Satt324	33.25 cM (5,927,346 a2)	Growth chamber/isolates *MS06-1, LA04-1*	F2:3 (104)	R gene	PI 567102B	Li et al. (2012)
		GSM0374 and GSM0427	(5,998,461 to 6,160,481 a1)	Greenhouse test/isolate *GA12*	F5:6 (184)	R gene	PI 567068A	King et al. (2016)
	–	BARC-016867–02,359 and BARC-048761–10,703	(51,814,496–52,157,617a2)	Field inoculation (Ha Noi, Vietnam)	F6:7 (250)	12.5%	DT2000 (PI 635999)	Vuong et al. (2016)
		Satt288_BARC-024489–04,936	(51,127,425–55,000,817a2	Field inoculation (Ha Noi, Vietnam)	F6:7 (250)	9.6%	DT2000' (PI 635999)	Vuong et al. (2016)
MLG L (Chr. 19)	*Rpp7*	W82 x PI between the markers GSM0546 and GSM0463;	(39,462,291 to 39,616,643 a1)	*AU79-1 (Australia), CO04-2 (Armenia, Columbia), GA12-1 (Georgia, USA),* *HW98-1 (Hawai), IN73-1 (India), LA04-1(Lousiana, USA), TW72-1 (Taiwan), VT05-1 (Vietnam), ZM01-1 (Zimbabwe)*	F2:3 (90/100)	R gene	PI 605823	Childs et al. (2018b)
		5601 T x PI between markers GSM0461 and GSM0468	(39,462,291 to 39,616,643 a1)	*AU79-1 (Australia), CO04-2 (Armenia, Columbia), GA12-1 (Georgia, USA),* *HW98-1 (Hawai), IN73-1 (India), LA04-1(Lousiana, USA), TW72-1 (Taiwan), VT05-1 (Vietnam), ZM01-1 (Zimbabwe)*	F4:5 (114)	R gene	PI 605823	Childs et al. (2018b)

^a^Marker position (bp) based on the *Glycine max* genome assembly version *Gmax1.01* (a1), or *Gmax2.0* (a2), only starting position is shown for SSR markers

^b^Phenotypic variations explained by the molecular markers

The improvement effort to know the physical location of the *Rpp* genes (resistance to *P. pachyrhizi*) is a great challenge today. However, despite the publication of the soybean genome (Schmutz et al. 2010), no *Rpp* gene has yet been cloned. For this reason, other authors have tried to identify the candidate genes linked to the *Rpp3* gene through a massive transcriptomic approach, using NILs populations. These genes are mostly related to phenylpropanoid branch isoflavonoid pathway-specific phytoalexin, glyceollin biosynthesis (Hossain 2019).

The presence of multiple virulence genes in the pathogen population and the lack of multiple resistance genes in the host give the soybean rust pathogen a competitive advantage. Therefore, the deployment of specific single genes for resistance is unlikely to be a successful strategy (Jarvie 2009).

Although varieties with pathotype-specific resistance genes were released, the stability of this resistance is uncertain since the large number of races of this fungus already described demonstrates the great variability of the pathogen. Understanding the molecular mechanisms involved in defense responses is of primary importance to plan strategies to control stress and, consequently, to increase the adaptation of plants to limiting conditions. Molecular markers have been considered tools for a large number of applications ranging from the location of a gene to the improvement of plant varieties through MAS. Also, the analysis of the soybean genome has generated a large amount of information and several databases with molecular markers are being generated that could be used for genetic improvement (Vuong et al. 2016; Tadesse 2019).

### Strategies for ASR resistance

The introgression of vertical resistance through classical breeding followed by MAS allows the development of resistant varieties and their use as an efficient and cost-effective method to control soybean rust (Tadesse 2019). An example to highlight is the pyramiding of several *Rpps* genes in a single line. Yamanaka et al. (2015a, b) managed to develop highly resistant experimental lines with stacks of three genes: *Rpp2* + *Rpp3* + *Rpp4* and *Rpp2* + *Rpp4* + *Rpp5*.

Pathotype-specific resistance genes and molecular markers are known to facilitate selections. However, the resistance provided by major genes tend to be broken rapidly; thus, research should be focused on the role of quantitative minor genes (QTLs) which are more likely to provide durable resistance to this highly variable pathogen.

To date, only one attempt to enhance resistance ASR based on transgenic technology has been recorded (Soto et al. 2020). In this study, constitutive expression of the *NmDef02* gene from *Nicotiana magalosiphon* demonstrated significantly increased resistance in soybean against *Phakopsora pachyrhizi* in field experiments.

The most recent and novel attempt to control this disease is the treatment of liquid suspension of cellulose nanofibers (CNF) to plants before inoculation with the pathogen. The authors suggest that this application changes the hydrophobicity of the leaf surface, suppressing *P. pachyrhizi* CHSs (chitin synthases) expression related to chitin formation, which are associated with reduced formation of pre-infection structures (Saito et al. 2021).

## Frogeye leaf spot, Cercospora leaf blight and purple seed stain

There are three soybean diseases caused by *Cercospora* spp.: frogeye leaf spot (FLS), Cercospora leaf blight (CLB), and purple seed stain (PSS). FLS, caused by *C. sojina* Hara, is an important foliar disease in soybean in the USA, Brazil, and China (Laviolette et al. 1970; Bernaux 1979; Dashiell and Akem 1991; Akem and Dashiell 1994; Ma 1994; Mian et al. 1998). Symptoms start on leaves as small, light brown circular spots which develop into a darkish brown to reddish margin (Dashiell 1991). In addition to foliar symptoms, *C. sojina* can cause lesions on pods and infect soybean seeds. FLS is favored by warm temperatures and frequent rainfalls (Phillips 1999) and remains active throughout the growing season (Laviolette et al. 1970; Kim et al. 2013), which make FLS a major disease in the southern USA as well as in some regions of the Midwestern USA (Yang et al. 2001; Mengistu et al. 2002; Mian et al. 2008). Yield losses can range from 10 to 60% mainly due to the reduction in photosynthesis and leaf area by necrotic lesions and/or premature defoliation (Laviolette et al. 1970; Bernaux 1979; Dashiell and Akem 1991; Akem and Dashiell 1994; Ma 1994; Mian et al. 1998). Screening methods for FLS include field evaluations with natural inoculum or with inoculations, and greenhouse inoculations of seedlings (Mian et al. 2008; Mengistu et al. 2012). Mian et al. (2008) proposed a set of 12 differential cultivars to determine races of *C. sojina*. With these differentials, they described 11 races from a collection of 93 *C. sojina* isolates collected in the USA. Three resistance genes (*Rcs*, Resistant to *C. sojina*) have been identified including *Rcs1*, *Rcs2,* and *Rcs3* (Table 13) (Athow and Probst, 1952; Athow et al. 1962; Phillips and Boerma 1982). *Rcs3* appears to confer resistance to all known races of *C. sojina* in the USA. *Rcs3* was further fine mapped on Chr. 16 (MLG J) (Mian et al. 1999; Missaoui et al. 2007a, b). In recent years, *Rcs*(PI 594891) and *Rcs*(PI 594774) were fine mapped and approved by the Soybean Genetic Committee as QTL that confers resistance to FLS (Hoskin 2011; Pham et al. 2015); In addition, two major QTLs were mapped on chromosomes 6 and 8, respectively, conferring resistance to *C. sojina* race 2 (ATCC 44,531) (Sharma and Lightfoot 2014); *Rcs15-02* was mapped on Chr. 6 (MLG C2); the ss715594329—ss715594474 interval was mapped on chromosome 6 (MLG C2) (Smith 2021); the ss715610717—ss715610843 interval was mapped on chromosome 11 (MLG B1)(Smith 2021); the ss715614578—ss715615158 interval was mapped on chromosome 13 (MLG F) (McAllister et al. 2021); and *Rcs15-01* was mapped on Chr. 19 (MLG L) (Lee 2021).

**Table 13:** Soybean loci conferring resistance to frogeye leaf spot (caused by *Cercospora sojina*) and Cercospora leaf blight/purple seed stain

Disease name	Causal agent	MLG (Chr.)	Locus name	Tightly linked/flanking markers	Marker position cM (bp)^a^	Testing methods/Resistance spectrum	Population type (size)	PVE^b^	Donor source	References
Frogeye leaf spot	*Cercospora sojina*	–	*Rcs1*	–	–	race *1*	F2	R gene	Lincoln	Athow and Probst (1952), Pham et al. (2015)
		–	*Rcs2*	–	–	race *2*	–	R gene	Kent	Athow et al. (1962), Pham et al. (2015)
		–	–	–	–	Greenhouse test	F2	R gene	Ransom, Lee, and Stonewall	Pace et al (1993)
		MLG A1 (Chr. 5)	–	Satt276	5,158,623	Greenhouse test/race *2*	F5:14 (94)	13%	Forrest	Sharma and Lightfoot (2014)
		MLG C2 (Chr. 6)	–	ss715594329–ss715594474	39,188,086—43,688,393	–	–	–	–	Smith (2021)
			*-*	Satt319—Satt079	38,049,354—44,503,658	Greenhouse test/race *2*	F5:14 (94)	52%	Essex	Sharma and Lightfoot (2014)
			*Rcs15-02*	–	–	–	–	–	–	Smith (2021)
		MLG M (Chr. 7)	–	Satt323	10,465,123	Greenhouse test/race *2*	F5:14 (94)	4%	Essex	Sharma and Lightfoot (2014)
			–	B35H07	–	Greenhouse test/race *2*	F5:14 (94)	5%	Essex	Sharma and Lightfoot (2014)
		MLG A2 (Chr. 8)	–	Satt589	5,182,879	Greenhouse test/race *2*	F5:14 (94)	11%	Forrest	Sharma and Lightfoot (2014)
			–	Satt632—A2D8	8,223,512	Greenhouse test/race *2*	F5:14 (94)	15%	Essex	Sharma and Lightfoot (2014)
		MLG K (Chr. 9)	–	Satt555	8,020,345	Greenhouse test/race *2*	F5:14 (94)	10%	Forrest	Sharma and Lightfoot (2014)
			–	Sat_116	–	Greenhouse test/race *2*	F5:14 (94)	11%	Forrest	Sharma and Lightfoot (2014)
		MLG O (Chr. 10)	–	Satt259	–	Greenhouse test/race *2*	F5:14 (94)	4%	Essex	Sharma and Lightfoot (2014)
		MLG B1 (Chr. 11)	–	ss715610717—ss715610843	4,338,907—5,248,257	–	–	–	–	Smith (2021)
			–	Satt444	29,759,281	Greenhouse test/race *2*	F5:14 (94)	6–12%	Forrest	Sharma and Lightfoot (2014)
		MLG H (Chr. 12)	–	Satt293	36,036,485	Greenhouse test/race *2*	F5:14 (94)	6%	Essex	Sharma and Lightfoot (2014)
		MLG F (Chr. 13)	*Rcs(PI 594774)*	Satt663–Satt114	25,936,631–28,912,864	Greenhouse test	F2:3 (195)	R gene	PI 594774	Hoskins (2011)
			–	ss715614578–ss715615158	28,207,736–31,449,060	–	–	–	–	Smith (2021)
			*Rcs(PI 594891)*	Satt114–Sct_033	28,912,864	Greenhouse test	F2:3 (110)	R gene	PI 594891	Hoskins (2011)
			–	CFR2	–	Greenhouse test/race *2*	F5:14 (94)	9%	Forrest	Sharma and Lightfoot (2014)
		MLG J (Chr. 16)	–	Satt249	1,149,373	Greenhouse test/race *2*	F5:14 (94)	8%	Essex	Sharma and Lightfoot (2014)
			*Rcs3*	Satt244—Satt547; AZ573TA150 and AZ573CA393	33,818,897–34,035,180	Greenhouse test/all known races	F2:3 (123)	R gene	Davis	Phillips and Boerma (1982), Boerma and Phillips, (1983), Mian et al. (1999), Missaoui et al. (2007a, b), Pham et al. (2015)
		MLG G (Chr. 18)	–	CGG-SCAR	–	Greenhouse test/race *2*	F5:14 (94)	6%	Forrest	Sharma and Lightfoot (2014)
		MLG L (Chr. 19)	–	Satt446	1,678,377	Greenhouse test/race *2*	F5:14 (94)	4–5%	Forrest	Sharma and Lightfoot (2014)
			*Rcs15-01*	–	–	–	–	–	–	Lee (2021)
		MLG I (Chr. 20)	–	Satt440	46,787,225	Greenhouse test/race *2*	F5:14 (94)	15%	Essex	Sharma and Lightfoot (2014)
Cercospora leaf blight/Purple seed stain	*Cercospora kikuchii*	MLG G (Chr. 18)	*Rpss1*	Sat_308 and Satt594	6.6 and 11.6 cM (11,426,775–22,375,695)	Field test	F2 (148)	R gene	PI 80837	Jackson et al. (2008)

^a^Marker position (bp) based on the *Glycine max* genome assembly version *Gmax2.0*

^b^Phenotypic variations explained by the molecular markers

CLB and PSS are two closely related diseases caused by the same or similar pathogens. The causal agent of both CLB and PSS was identified as *Cercospora kikuchii* (Matsumoto & Tomoyasu) M. W. Gardner (Matsumoto and Tomoyasu 1925; Walters 1980); however, recent studies have found *C. flagellaris* and *C. sigsbeckiae* were the primary species associated with both diseases in the southern USA. CLB begins as a purpling of the upper leaves starting during seed development. This purpling can cover the entire leaf surface. Symptoms can advance to blighting where the entire leaf becomes chlorotic and necrotic with the leaflets falling off leaving the petioles attached. The pathogen produces a toxin, ‘cercosporin’, whose production requires light exposure. As a result, CLB symptoms begin at the upper end top of the plant and progress to the lower leaves. In severe cases, the whole plant may be defoliated. Yield losses for PSS have been estimated at 0.12–0.28 million Mt (Allen et al. 2017) whereas CLB causes an estimated yield loss of 23% in the USA (Wrather et al. 1997). On seed, infection causes a purpling of the seed coat. Seed infection is usually not associated with yield loss but can reduce seed germination and may lead to infected seedlings. Although both CLB and PSS are favored by high moisture and warm temperatures during early pod development (Jones 1968; Schuh 1990), the occurrence of these diseases appears to be independent of each other (Orth and Schuh, 1994; Walters 1985).

Based on natural field inoculum, Srisombun and Supapornhemin (1993) reported resistance to PSS in the soybean cultivar ‘SJ2’ and that this resistance may be due to a single dominant gene. Resistance to PSS was also reported in PI 80837, PI 417274, PI 417460, and the cultivar ‘Gnome’ (Wilcox et al. 1975; Ploper et al. 1992). The resistance in PI 80837 was attributed to a single gene on linkage group G, *Rpss1* (Jackson et al. 2006, 2008) (Table 13). Additional PIs were identified as resistant sources to both CLB and PSS (Alloatti et al. 2015) or only to PSS (Li et al. 2019). Several studies of population genetics have found differences in genetic structure among populations and pathogenicity of groups throughout the Americas (Almeida et al. 2005; Cai et al.^2009^; Lurá et al. 2011). It is unknown if the reactions of these soybean lines to CLB and PSS will remain consistent with the new species of *Cercospora* associated with these diseases.

## Charcoal rot

The worldwide distributed charcoal rot disease of soybean is caused by *Macrophomina phaseolina* (Tassi) Goid (Smith and Wyllie 1999). *M. phaseolina* is a soilborne plant pathogen causing disease infection in more than 500 plant species (Su et al. 2001; Mengistu et al. 2007). Charcoal rot is one of the primary diseases of soybean in the USA and Canada (Bandara et al. 2020; Roth et al. 2020) resulting estimated yield losses between 0.73 and 2.0 million Mt from 2010 to 2014 (Allen et al. 2017). Disease severity is favored by the increase in soil and air temperature (28–35 °C) (Mengistu et al. 2014), and symptoms include stunted growth, leaf chlorosis, premature yellowing and early maturation, or incomplete pod filling (Gupta et al. 2012; Mengistu et al. 2016). Management strategies include crop rotation with non-host crops, such as cotton, wheat, and barley that can lower inoculum load in the soil, and avoidance of water stress especially during the reproductive stage of soybeans. (Almeida et al. 2003; García‐Olivares et al. 2012; Vibha 2016). Biological control with Trichoderma isolates has been proposed by researchers as a possible alternative to control charcoal rot (Khalili et al. 2016; Orojnia et al. 2021). However, host plant resistance is the most viable method to control the disease (Mengistu et al. 2011; Coser et al. 2017). Little is known regarding the genetics and heritability of the pathogen and there is a lack of reliable and efficient screening method for this disease (Mengistu et al. 2008). Until 2018, no soybean genotype having a high level of resistance to *M. phaseolina* had been identified (Mengistu et al. 2018). Recently, a report by Nataraj et al. (2019), summarized eleven soybean genotypes identified as moderately resistant to charcoal rot along with pedigree information. Reznikov et al. (2019) found that cv. ‘Munasqa RR’ carried superior resistance to *M. phaseolina.* In addition, the University of Missouri-Fisher Delta Research Center has released varieties showing superior resistance to charcoal rot (Chen et al. 2020, 2021b). Based on field research studies conducted over the last several years, over 2,000 soybean genotypes have been screened for CR resistance, and of these genotypes, approximately 25 have been identified as having moderate resistance against charcoal rot (Mengistu et al. 2007, 2011, 2013). Recently, Mengistu et al. (2021) screened a set of 120 soybean accessions known to have resistance to one or more races of SCN. Twelve of these accessions have been identified to have moderate charcoal rot resistance combined with resistance to SCN. These accessions are archived and will be available through the Germplasm Resources Information Network (GRIN) system of the USDA. Even though moderately resistant cultivars have been identified, the lack of identifying a complete resistance has delayed the progress to better understanding the genetics of resistance. Most of those genotypes were screened using at least one of the six screening methods for the disease assessment including: colony-forming unit index (CFUI); root stem severity (RSS); percent height of stem discoloration (PHSD); foliar symptoms (FS); cut-stem inoculation method; and seed plate assay (SPA) (Mengistu et al. 2007; Twizeyimana et al. 2012; da Silva et al. 2019). Of all these methods, CFUI and RSS have been the stay methods for charcoal rot assessment currently used in the field.

Recently, QTL mapping and GWA studies were reported on multiple genomic regions harboring horizontal resistance to charcoal rot in soybean, which may be used to facilitate breeding and MAS against this pathogen (Table 14) (Coser et al. 2017; da Silva et al. 2019, 2020; Ghorbanipour et al. 2019). More efforts are needed to identify complete resistant sources and develop tightly linked molecular markers to facilitate breeding resistant varieties.

**Table 14:** Soybean loci conferring resistance to charcoal rot (caused by *Macrophomina phaseolina*)

MLG (Chr.)	Linked/flanking markers	Marker position/bp	Testing methods/Resistance spectrum	Population type (size)	PVE^b^	Donor source	References
MLG D1b (Chr. 2)	Sat_169	37,813,855 a2	Field inoculation/isolate S_8_	Maturity group I-V (130)	10% number of microsclerotia in stem, 13% amount of charcoal rot disease, 5% severity of charcoal rot disease	–	Ghorbanipour et al. (2019)
	Satt644	38,221,027 a2	Field inoculation/isolate S_8_	Maturity group I-V (130)	12% 100 grain weight	–	Ghorbanipour et al. (2019)
MLG C1 (Chr. 4)	ss715588228	4,307,731 a2	Field test and cut-stem inoculation technique/isolate from Iowa soybean field	USDA PI lines (459)	–	–	Coser et al. (2017)
	Satt607	8,165,631 a2	Field inoculation/isolate S_8_	Maturity group I-V (130)	9% 100 grain weight	–	Ghorbanipour et al. (2019)
	Sat_404	13,613,713 a2	Field inoculation/isolate S_8_	Maturity group I-V (130)	8% pod weight	–	Ghorbanipour et al. (2019)
	Satt190	16,738,759 a2	Field inoculation/isolate S_8_	Maturity group I-V (130)	9% number of microsclerotia in stem, 7% amount of charcoal rot disease, 7% severity of charcoal rot disease	–	Ghorbanipour et al. (2019)
	Satt361	32,617,784 a2	Field inoculation/isolate S_8_	Maturity group I-V (130)	9% pod weight	–	Ghorbanipour et al. (2019)
	Sat_357	33,970,110 a2	Field inoculation/isolate S_8_	Maturity group I-V (130)	10% 100 grain weight	–	Ghorbanipour et al. (2019)
	Sat_416	40,624,709 a2	Field inoculation/isolate S_8_	Maturity group I-V (130)	11% number of microsclerotia in stem, 12% amount of charcoal rot disease	–	Ghorbanipour et al. (2019)
MLG A1 (Chr. 5)	–	25,338,390 a2	Cut-stem inoculation technique/isolate *Conway*	F2:3 (140)	–	PI 567562A	da Silva et al. (2020)
MLG C2 (Chr. 6)	ss715593307	14,918,492 a2	Field test and cut-stem inoculation technique/isolate from Iowa soybean field	USDA PI lines (459)	–	–	Coser et al. (2017)
	Sat_238	43,990,660 a2	Field inoculation/isolate S_8_	Maturity group I-V (130)	8% pod weight, 13% grain weight, 8% grain yield	–	Ghorbanipour et al. (2019)
	Satt460	44,049,891 a2	Field inoculation/isolate S_8_	Maturity group I-V (130)	9% grain weight, 8% number of microsclerotia in stem, 10% severity of charcoal rot disease	–	Ghorbanipour et al. (2019)
	Satt079	44,503,658 a2	Field inoculation/isolate S_8_	Maturity group I-V (130)	11% grain weight, 8% 100 grain weight	–	Ghorbanipour et al. (2019)
	Sct_028	46,273,196 a1	Field inoculation/isolate S_8_	Maturity group I-V (130)	6% grain weight	–	Ghorbanipour et al. (2019)
	Sat_252	48,211,009 a2	Field inoculation/isolate S_8_	Maturity group I-V (130)	11% pod weight, 12% 100 grain weight, 10% amount of charcoal rot disease	–	Ghorbanipour et al. (2019)
MLG A2 (Chr. 8)	–	7,511,708 a2	Cut-stem inoculation technique/isolate *Conway*	F2:3 (140)	–	PI 567562A	da Silva et al. (2020)
	ss715601990	42,490,418 a2	Field test and cut-stem inoculation technique/isolate from Iowa soybean field	USDA PI lines (459)	–	–	Coser et al. (2017)
	ss715602087	43,618,993 a2	Field test and cut-stem inoculation technique/isolate from Iowa soybean field	USDA PI lines (459)	–	–	Coser et al. (2017)
MLG K (Chr. 9)	ss715604575	45,369,206 a2	Field test and cut-stem inoculation technique/isolate from Iowa soybean field	USDA PI lines (459)	–	–	Coser et al. (2017)
MLG B1 (Chr. 11)	Satt359	32,411,307 a2	Field inoculation/isolate S_8_	Maturity group I-V (130)	10% number of microsclerotia in stem, 9% amount of charcoal rot disease, 11% severity of charcoal rot disease	–	Ghorbanipour et al. (2019)
MLG H (Chr. 12)	ss715613120	492,020 a2	Field test and cut-stem inoculation technique/isolate from Iowa soybean field	USDA PI lines (459)	–	–	Coser et al. (2017)
	ss715612760	37,527,844 a2	Field test and cut-stem inoculation technique/isolate from Iowa soybean field	USDA PI lines (459)	–	–	Coser et al. (2017)
MLG B2 (Chr. 14)	ss715618004	219,725 a2	Field test and cut-stem inoculation technique/isolate from Iowa soybean field	USDA PI lines (459)	–	–	Coser et al. (2017)
	–	2,442,086 a2	Cut-stem inoculation technique/isolate *Conway*	F2:3 (140)	–	PI 567562A	da Silva et al. (2020)
MLG E (Chr. 15)	Gm15_01842053 and Gm15_03051337	1,842,060 a2	Cut-stem inoculation technique/isolate *Conway*	F2:3 (140)	29.4%	PI 567562A	da Silva et al. (2019)
MLG J (Chr. 16)	Gm16_28961127 and Gm16_30493887	29,328,591–30,862,012 a2	Cut-stem inoculation technique/isolate *Conway*	F2:3 (140)	25.4%	PI 567562A	da Silva et al. (2019)
	Gm16_35973543 and Gm16_37078478	36,476,386–37,570,986 a2	Cut-stem inoculation technique/isolate *Conway*	F2:3 (140)	8.84%	PI 567562A	da Silva et al. (2019)
MLG G (Chr. 18)	ss715631726	51,751,797 a2	Field test and cut-stem inoculation technique/isolate from Iowa soybean field	USDA PI lines (459)	–	–	Coser et al. (2017)
	ss715631906	53,502,168 a2	Field test and cut-stem inoculation technique/isolate from Iowa soybean field	USDA PI lines (459)	–	–	Coser et al. (2017)
	ss715632099	54,829,750 a2	Field test and cut-stem inoculation technique/isolate from Iowa soybean field	USDA PI lines (459)	–	–	Coser et al. (2017)
MLG L (Chr. 19)	Sat_124	50,728,020 a2	Field inoculation/isolate S_8_	Maturity group I-V (130)	11% grain weight, 11% 100 grain weight, 11% grain yield	–	Ghorbanipour et al. (2019)
MLG I (Chr. 20)	ss715638424	43,471,723 a2	Field test and cut-stem inoculation technique/isolate from Iowa soybean field	USDA PI lines (459)	–	–	Coser et al. (2017)
-	Satt512	–	Field inoculation/isolate S_8_	Maturity group I-V (130)	8% pod weight, 10% 100 grain weight	–	Ghorbanipour et al. (2019)
	S63880-CB	–	Field inoculation/isolate S_8_	Maturity group I-V (130)	11% grain weight, 7% grain yield	–	Ghorbanipour et al. (2019)

^a^Marker position (bp) based on the *Glycine max* genome assembly version *Gmax1.01* (a1), or *Gmax2.0* (a2), only starting position is shown for SSR markers

^b^Phenotypic variations explained by the molecular markers

## Brown stem rot

Brown stem rot (BSR) is a devastating soybean disease caused by a soilborne fungus, *Phialophora gregata* (syn. *Cadophora gregata*), which was first discovered in central Illinois in 1944 (Allington and Chamberlain 1948; Harrington and McNew 2003). There are two different types of *P. gregata* pathogen identified (Type I and II): Type I causes pith browning and interveinal chlorosis and necrosis of leaves, but Type II only causes pith browning (Gray 1972; Harrington et al. 2003). The disease caused annual yield loss of 0.35 million Mt in the Northern USA (Allen et al. 2017; Klos et al. 2000), and yield reduction can reach as high as 38% (Bachman et al. 2001). The most effective strategy to control BSR is the introgression of resistance genes into soybean cultivars (Klos et al. 2000; McCabe and Graham 2020). From previous studies, three genes (*Rbs*_*1*_, *Rbs*_*2*_, and *Rbs*_*3*_) for BSR resistance in soybean have been identified through allelism tests (Table 15) (Hanson et al. 1988; Willmot and Nickell 1989). Later, it was determined that all three genetic loci were in an overlapping region of Chr. 16 (28.9–36.2 Mb) (Lewers et al. 1999; Bachman et al. 2001). Recently, Rincker et al. (2016a) concluded that all three loci for BSR resistance were located in the same region, and that the resistance was conferred by a single gene based on their fine mapping (Rincker et al. 2016a) and GWA studies (Rincker et al. 2016b). To evaluate BSR resistance in soybean, Sebastian et al. (1983) established a greenhouse root-dip method, which has been modified and refined by further studies (Hanson et al. 1988; Willmot and Nickell 1989; Lewers et al. 1999; Bachman et al. 2001). Soybean PIs that have BSR resistance include PI 84946–2, PI 86150, PI 90238, PI 95769, PI 88820, PI 424285A, PI 424353, PI 424611A, PI 437833, and PI 437970 (Chamberlain and Bernard 1968; Tachibana and Card 1972; Hanson et al. 1988; Nelson et al. 1989; Wilmot and Nickell 1989).

**Table 15:** Soybean loci conferring resistance to brown stem rot (caused by *Phialophora gregata*)

MLG (Chr.)	Locus name	Tightly linked/flanking markers	Marker position cM (bp)^a^	Testing methods	Population type (size)	PVE^b^	Donor source	References
MLG J (Chr. 16)	*Rbs*_*1*_	Satt215	(28,944,536–28,944,665)	Greenhouse assay	F2:3 (73)	28%	L78-4094	Bachman et al. (2001)
		Satt431	(36,221,174–36,221,397)	Greenhouse assay	F2:3 (73)	74%	L78-4094	Bachman et al. (2001)
	*Rbs*_*2*_	Satt244	(33,818,897–33,819,094)	Greenhouse assay	F2:3 (77)	67%	PI 437833	Bachman et al. (2001)
		Satt431	(36,221,174–36,221,397)	Greenhouse assay	F2:3 (77)	46%	PI 437833	Bachman et al. (2001)
	*Rbs*_*3*_	K375	67.3–69.3 cM*	Greenhouse assay	F6:7 (320)	62%	PI 84946–2	Lewers et al. (1999)
		B122	53.8–55.8 cM*	Greenhouse assay	F6:7 (320)	45%	PI 84946–2	Lewers et al. (1999)

*GmComposite2003 genetic position (www.soybase.org)

^a^Marker position (bp) based on the *Glycine max* genome assembly version *Gmax2.0*

^b^Phenotypic variations explained by the molecular markers

## Rhizoctonia damping-off and root rot

Rhizoctonia damping-off and root rot is an important disease in soybean and can cause pre- and postemergence damping-off, seed rot, root rot, hypocotyl lesions, and web blight (Dorrance et al. 2003; Rahman et al. 2020). The causal agent, *Rhizoctonia solani* Kuhn, is a soilborne necrotrophic complex species that can host corn, soybean, and other crops such as wheat and potato, suggesting that management of Rhizoctonia root rot by rotations between these crops may not be effective (Ajayi-Oyetunde and Bradley 2017, 2018). The isolates of *R. solani* can be classified into 14 anastomosis groups (AGs) and more subgroups based on their genetic similarity. Different AGs may incite different symptoms of disease on soybean. For example, AG-2-2IIIB, AG-4 and AG-5 can cause seed rot, pre- and post-emergence damping-off, hypocotyl and root rot, and foliar blight on soybean, while AG-3, AG-7, and AG-11 cause very little damage (Ajayi-Oyetunde and Bradley 2018). The management of Rhizoctonia root rot may include clean seeds, tillage, fungicides, and deployment of resistant cultivars if possible. Unfortunately, currently there is no commercial resistant cultivars available to the market, and the genetic research against Rhizoctonia root rot is inadequate. Only three SSR markers, Satt281, Satt177, and Satt245 (Table 16) have been found associated with partial resistance to AG-4 isolate (Zhao et al. 2005), although more germplasm lines and soybean varieties have been identified as potential sources of resistance (Muyolo et al. 1993; Bradley et al. 2001; Sharma 2020).

**Table 16:** soybean loci conferring resistance to Rhizoctonia damping off and root rot (caused by *Rhizoctonia solani*)

MLG (Chr.)	Locus name	Tightly linked / flanking markers	Marker position cM (bp)^a^	Testing methods / Resistance spectrum	Population type (size)	PVE^b^	Donor source	Reference
MLG C2 (Chr. 6)	-	Satt281	6,529,270	Greenhouse test / AG-4	F2(189), F4:5(23), F4:5(32)	11-39%	PI 442031	Zhao et al. (2005)
MLG M (Chr. 7)	-	Satt245	9,357,717	Greenhouse test / AG-4	F2(189), F4:5(23), F4:5(32)	6.8-14%	PI 442031	Zhao et al. (2005)
MLG A2 (Chr. 8)	-	Satt177	36.77cM *	Greenhouse test / AG-4	F2(189), F4:5(23), F4:5(32)	7-23%	PI 442031	Zhao et al. (2005)

^a^ Marker position (bp) based on the Glycine max genome assembly version Gmax2.0

^b^ Phenotypic variations explained by the molecular markers.

^*^ GmComposite2003 genetic position (www.soybase.org)

## Other fungal diseases

### Taproot decline

Taproot decline is a disease caused by *Xylaria necrophora* sp. nov. (Garcia-Aroca et al. 2021)*,* a recently identified pathogen that was overlooked since some of the symptoms were similar to other soybean root diseases including SDS and charcoal rot. This soilborne pathogen can affect seedlings; however, the symptoms in the field develop later in the season producing interveinal chlorosis followed by necrosis. It has been noted that *X. necrophora* will affect the root to the point that pulling plants from the ground causes the root system to break with black stroma visible on the root tissue (Allen et al. 2017). The disease is mostly managed with cultural practices, but cultivar trials are ongoing. The cv. ‘Osage’ (PI 648270) has tolerance to this pathogen (Purvis 2019). Osage was developed in Arkansas and also has resistance to SDS, stem canker, and frogeye leaf spot (Chen et al. 2007).

## Red leaf blotch

Red leaf blotch affects soybean plants in several Eastern, Central, and Southern African countries. The disease (also known as Pyrenochaeta leaf spot or blotch, and Dactuliophora leaf spot) can cause yield losses of up to 50% (Hartman et al. 1987, 2016). The causal agent is *Coniothyrium glycines* (R.B. Stewart) Verkley & Gruyter, a fungus previously named *Phoma glycinicola*, *Dactuliochaeta glycines*, *Dactuliophora glycines*, and *Pyrenochaeta glycines*. The disease affects foliage, petioles, pods, and stems, and may cause severe leaf blotching, defoliation, and premature senescence. Because of the potential negative consequences of this disease to US agriculture if introduced, *C. glycines* is listed as a select agent by the Federal Select Agent Program (Tooley 2017).

Since the 1980s, soybean germplasm has been evaluated under field conditions in African countries for reaction to red leaf blotch. Despite this extensive field testing, no sources of resistance have yet been identified among US soybean commercial cultivars, local lines, or exotic soybean lines. These evaluations were carried out in regions where red leaf blotch is endemic (Sinclair 1989). A field method to assess the infection of soybean by the pathogen was developed and used to evaluate cultivar reaction and efficacy of chemical control (Levy et al. 1990).

A seedling inoculation method has also been proposed which allows optimal infection in less space over a shorter period than field trials and without relying on the occurrence of natural inoculum and disease conducive environmental conditions. Soybean genotypes that represent nearly 90% of the genes present in US soybean were evaluated and found to be susceptible, which is consistent with previous field evaluations (Tooley 2017).

Studies are necessary to evaluate genetic variability within the pathogen population from different countries, and to assess potential interactions with soybean genotypes. With limited genomic information of the pathogens known, there are no molecular genotyping or detection methods available. Recently, the draft genome sequences of three *C. glycines* isolates were reported, enhancing the knowledge of this species (Blagden et al. 2019).

## Section IV Soybean resistance to bacterial diseases

### Bacterial blight

Soybean bacterial blight caused by *Pseudomonas savastanoi* pv. *Glycinea* Coerper (formerly *Pseudomonas syringae* pv. *glycinea*) is a widespread soybean disease. Although bacterial blight is not a major suppressor of soybean yield in the USA (Williams and Nyvall 1980; Hwang and Lim 1992), the interaction between soybean and the pathogen was well known as a model system to study gene-for-gene host-parasite relationships (Huynh et al. 1989). Five resistance genes/alleles have been identified named *Rpg1-b*, *Rpg1-r*, *Rpg2*, *Rpg3*, and *Rpg4*, conferring resistance to the corresponding *Psg* avirulence factors AvrB, AvrRpm1, AvrA, AvrC, and AvrD, respectively (Staskawicz et al. 1987; Keen and Buzzell. 1991; Ashfield et al. 1998; Khan et al. 2011; Whitham et al. 2016). The *Rpg1-b* and *Rpg1-r* genes were located on MLG F (Chr. 13) (Ashfield et al. 1998) and have been cloned in 2004 and 2014, respectively (Ashfield et al. 2004, 2014). *Rpg2* is loosely linked with *Rpg1*, and *Rpg3* is linked with *Rpg4* at 40.5 ± 3.2 recombination units (Table 17) (Keen and Buzzel. 1991).

**Table 17:** Soybean genes/loci conferring resistance to bacteria diseases

Disease Name	Causal agent	MLG (Chr.)	Locus name	Tightly linked/flanking markers	Marker position cM (bp)^a^	Testing methods/Resistance spectrum	PVE^b^	Donor source	References
Bacterial blight	*Pseudomonas syringae* pv. *glycinea*	MLG F (Chr. 13)	*Rpg1-b* (RGA-84B)	Flanked by K644 and B212. RFLP markers R45, php2265. php2385 co-segregated with *Rpg1*	67.18–69.83 cM *	AvrB	R gene	Norchief, Harosoy, PI 132207, Merit, BSR 101, Williams82,	Mukherjee et al. (1966), Staskawicz et al. (1987), Ashfield et al. (1998), Ashfield et al. (2004)
			*Rpg1-r* (P21f22_29)	Flanked by K644 and B212. RFLP markers R45, php2265. php2385 co-segregated with *Rpg1*	67.18–69.83 cM *	AvrRpm1	R gene	Flambeau, PI 96983	Ashfield et al. (1998), Ashfield et al. (2014)
			*Rpg2*	Loosely linked with *Rpg1*	–	AvrA	R gene	Merit	Keen and Buzzell (1991), Whitham et al. (2016)
		*–*	*Rpg3*	Linked with *Rpg4*	*–*	AvrC	R gene	Merit, Flambeau	Keen and Buzzell (1991), Whitham et al. (2016)
		*–*	*Rpg4*	Linked with *Rpg3*	*–*	AvrD	R gene	Flambeau	Keen and Buzzell (1991), Khan et al. (2011), Whitham et al. (2016)
Bacterial pustule	*Xanthomonas axonopodis* pv. *glycines*	MLG D1a (Chr. 1)	*–*	ss715580342	(53,136,582 a2)	*–*	*–*	*–*	Chang et al. (2016)
		MLG K (Chr. 9)	*–*	Satt137	(5,753,983 a1)	*–*	5.5%	Keunolkong	Seo et al. (2009)
		MLG O (Chr. 10)	*–*	Sat_108	(48,199,089 a2)	*–*	Single recessive gene	PI 96188	Kim et al. (2011)
		MLG B1 (Chr. 11)	*–*	ss715609404	(26,963,752 a2)	*–*	*–*	*–*	Chang et al. (2016)
		MLG B2 (Chr. 14)	*–*	Satt556	(38,859,467 a2)	*–*	7.3%	Keunolkong	Seo et al. (2009)
		MLG D2 (Chr. 17)	*rxp*	Satt014 and Satt372; Satt486; Rxp17-700; SNUSSR17_9 and SNUSNP17_12	(6,475,946—7,542,029 a2)	*–*	Single recessive gene	CNS (PI 548445), Young, Coker237	Feaster (1951), Hartwig and Lehman (1951), Bernard and Weiss (1973), Hwang and Kim (1987), Palmer et al. (1992), Narvel et al. (2001), Kim et al. (2004), Kim et al. (2010), Yang et al. (2011), Chang et al. (2016)
			*–*	Satt135 and Satt397	(6,156,526—11,724,482 a1)	*–*	20.9%	Keunolkong	Seo et al. (2009)
		MLG I (Chr. 20)	*–*	Satt496	(27,664,504 a2)	*–*	2.7%	Keunolkong	Seo et al. (2009)

*GmComposite2003 genetic position (www.soybase.org)

^a^Marker position (bp) based on the *Glycine max* genome assembly version *Gmax1.01* (a1), or *Gmax2.0* (a2), only starting position is shown for SSR markers

^b^Phenotypic variations explained by the molecular markers

## Bacterial pustule

Soybean bacterial pustule is a common disease in regions with warm and wet conditions (Bernard and Weiss 1973; Kennedy and Tachibana 1973; Matsuo et al. 2017). The causal agent, *Xanthomonas axonopodis* pv. *glycines*, can cause small, pale green spots with elevated pustules in the center of lesions, which can grow into large necrotic lesions causing premature defoliation (Kennedy and Tachibana 1973; Narvel et al. 2001). The first identified resistance gene is *rxp* from cv. ‘CNS’ and was initially mapped between Satt014 and Satt372 on MLG D2 (Chr. 17) (Feaster 1951; Hartwig and Lehman 1951; Bernard and Weiss 1973; Hwang and Kim 1987; Palmer et al. 1992; Narvel et al. 2001). Further studies narrowed the *rxp* locus down to a 33 kb genomic region between markers SNUSSR17_9 and SNUSNP17_12, with two candidate genes identified (Kim et al. 2010). In addition, another single recessive resistance gene was identified from PI 96188. The gene was located on MLG O (Chr. 10) and was closely linked with Sat_108 (Kim et al. 2011). QTLs have also been reported against bacterial pustule (Van et al. 2004; Seo et al. 2009; Chang et al. 2016). For example, Seo et al. (2009) reported four QTLs on chromosomes 9, 14, 17 and 20, explaining 2.7–20.9% of phenotypic variations (Table 17).

## Section V Soybean resistance to virus diseases

### Soybean mosaic virus

Soybean mosaic virus (SMV) is a major global viral pathogen in soybean that can compromise the soybean value chain by causing expressive yield losses of up to 90% in severe outbreaks (Ren et al. 1997a; Wang et al. 2001). SMV is widely distributed in soybean-growing countries including Brazil, Canada, China, Japan, Korea, and the USA (Cho and Goodman 1979; Li et al. 2010b, 2015b). In China, the occurrence of SMV is gradually increasing throughout the country and it currently represents the most prevalent disease in soybean with annual yield losses reaching over 50% (Zhang et al. 1980, 2015b). Typical SMV symptoms include reduced seedling viability and vigor, flower abortion, reduction of pod set, seed number, and seed size (Hill et al. 1987; Ren et al. 1997b; Gunduz et al. 2004). The severity of the symptoms is dependent on the host genotype, virus strain, plant stage at infection, as well as environmental factors (Bos 1972).

SMV is classified into strains based on its virulence and observed symptoms and differs between countries. In the USA, SMV isolates are classified into seven strains (G1–G7), where G1 is the least virulent affecting only susceptible genotypes whereas G7 is the most virulent capable of infecting both resistant and susceptible soybean genotypes (Cho and Goodman 1979). In China, SMV is classified into 21 groups (SC1–SC21) according to geographical regions and individual genotypes responses (Moon et al. 2009; Li et al. 2010b). Genetic resistance is the most efficient strategy to control SMV (Gunduz et al. 2004). To date, four independent loci for SMV resistance, *Rsv1*, *Rsv3*, *Rsv4*, and *Rsv5* have been identified (Kiihl and Hartwig 1979; Buzzell and Tu 1984; Buss et al. 1997; Li et al. 2010c; Klepadlo et al. 2017) although most of the modern commercial cultivars are susceptible to SMV, particularly to more virulent strains (Table 18) (Zheng et al. 2005a, b; Shakiba et al. 2012a).

**Table 18:** Soybean loci conferring resistance to soybean mosaic virus (SMV)

MLG (Chr.)	Locus/allele name	Tightly linked/flanking markers	Marker position (bp)^a^	Testing methods/Resistance spectrum	PVE^b^	Population Type (size)	Donor source	References
MLG D1b (Chr. 2)	*Rsv4*	–	–	Greenhouse Screening SMV Strains G1–G7	–	F2:3 (117)	PI 88788	Gunduz et al. (2004)
		Barc-011147–00,855	8,380,603	Greenhouse Screening SMV Strains G1–G7	*–*	Diverse Genotypes (47)	*–*	Shi et al. (2011)
		Barc-025955–05,182	9,689,348	Greenhouse Screening SMV Strains G1–G7	*–*	Diverse Genotypes (47)	*–*	Shi et al. (2011)
		AW307114-indel	12,585,482	Greenhouse Screening SMV Strains G1–G7	–	Diverse Genotypes (47)	–	Shi et al. (2011)
		Satt558	10,619,724	Greenhouse Screening SMV Strains G1–G7	–	F2 (255)	LR2	Hayes et al. (2000)
		Satt542	13,316,465	Greenhouse Screening SMV Strains G1–G7	–	F2 (255)	LR2	Hayes et al. (2000)
		BARCSOYSSR_02_0610	11,964,524	Greenhouse Screening SMV Strain SC8	–	F7:11 (184)	Kefeng No. 1	Wang et al. (2011)
		BARCSOYSSR_02_0616	12,070,465	Greenhouse Screening SMV Strain SC8	–	F7:11 (184)	Kefeng No. 1	Wang et al. (2011)
		Sat_254	11,168,955	Greenhouse Screening SMV Strain G7	–	F2 (561)	PI 486355	Hwang et al. (2006)
		ss244712184	11,613,852	Greenhouse Screening SMV Strains G1 and G7	–	F2 (766)	V94-5152	Klepadlo et al. (2017)
		ss244712591	11,685,678	Greenhouse Screening SMV Strains G1 and G7	–	F2 (766)	V94-5152	Klepadlo et al. (2017)
		ss244712651	11,693,196	Greenhouse Screening SMV Strains G1 and G7	–	F2 (766)	V94-5152	Klepadlo et al. (2017)
		ss244712652	11,693,604	Greenhouse Screening SMV Strains G1 and G7	–	F2 (766)	V94-5152	Klepadlo et al. (2017)
		ss244712653	11,693,900	Greenhouse Screening SMV Strains G1 and G7	–	F2 (766)	V94-5152	Klepadlo et al. (2017)
		ss244712671	11,697,977	Greenhouse Screening SMV Strains G1 and G7	–	F2 (766)	V94-5152	Klepadlo et al. (2017)
		R4at3	11,964,498	Greenhouse Screening SMV Strains G1 and G7	–	BC3F2 (309)	V94-5152	Ilut et al. (2016)
		Rat2	12,044,285	Greenhouse Screening SMV Strains G1 and G7	–	BC3F2 (309)	V94-5152	Ilut et al. (2016)
		Sms1	12,156,384	Greenhouse Screening SMV Strains G1 and G7	–	BC3F2 (309)	V94-5152	Ilut et al. (2016)
		Sm0	12,276,844	Greenhouse Screening SMV Strains G1 and G7	–	BC3F2 (309)	V94-5152	Ilut et al. (2016)
		BARC‐021,625‐04,157	12,623,066	Greenhouse Screening SMV Strain SC7	5.0%	Soybean Accessions (191) F7:16 (184)	Kefeng No. 1	Yan et al. (2015)
	*Rsv4-b*	–	–	Greenhouse Screening SMV Strains G7	–	F2 (616) F2:3 (289)	Beeson	Shakiba et al. (2013)
	*Rsv4-v*	Satt634	11,441,849	Greenhouse Screening SMV Strains G7	–	F2:3 (403)	PI 438307	Klepadlo et al. (2016)
		Satt296	12,975,935					
	*Rsc5*	Bin 352	11,300,000	Greenhouse Screening SMV Strain SC5	–	F7 (427)	Kefeng No. 1	Karthikeyan et al. (2017)
		Bin 353	11,800,000					
	*Rsc7*	Satt266	14,288,241	Greenhouse Screening SMV Strain SC7	4.1–10.6%	Soybean Accessions (191) F7:16 (184)	Kefeng No. 1	Yan et al. (2015)
		Satt634	11,778,505					
	*Rsc8*	ZL-42	12,060,386	Greenhouse Screening SMV Strain SC8	–	F2 (2122)	Kefeng No. 1	Lin et al. (2016), Zhao et al. (2016)
		ZL-52	12,091,080					
	*qSC3/7-D1b*	ss715580960–ss715581063	10,935,557–12,334,435	Greenhouse Screening SMV strains SC3 and SC7	54.2%	RIL (279)	Qihuang30	Chu et al. (2021a)
	*qSC7-D1b*	ss715581063–ss715581097	12,334,435–12,506,411	Greenhouse Screening SMV strain SC7	27.0%	RIL (279)	Qihuang30	Chu et al. (2021a)
	–	ss715583175	45,170,092	Greenhouse Screening SMV strain SC3	10.6%	Cultivars (302) and landraces (77)	–	Chu et al. (2021b)
MLG B1 (Chr. 11)	–	ss715608741	10,304,178	Greenhouse Screening SMV strain SC3	7.0%	Cultivars (302) and landraces (77)	–	Chu et al. (2021b)
MLG F (Chr. 13)	*Rsv1*	–	–	Greenhouse Screening SMV Strain SVM-1	–	F2 (1739)	PI 96983	Kiihl and Hartwig (1979)
		SoyHSP176	29,041,694	Greenhouse Screening SMV Strain G1	–	F2 (107)	PI 96983	Yu et al. (1996)
		3gG2-snp1	29,877,164	Greenhouse Screening SMV Strain G1-G7	–	Diverse Genotypes (47)	–	Shi et al. (2011)
		3gG2-snp2	30,402,642	Greenhouse Screening SMV Strain G1-G7	–	Diverse Genotypes (47)	–	Shi et al. (2011)
		N11PF-snp2	31,012,220	Greenhouse Screening SMV Strain G1-G7	–	Diverse Genotypes (47)	–	Shi et al. (2011)
		Barc-015435–01,966	32,607,605	Greenhouse Screening SMV Strain G1-G7	–	Diverse Genotypes (47)	–	Shi et al. (2011)
		BARCSOYSSR_13_1128	30,119,784	Greenhouse Screening SMV Strains SC3, SC6, SC7, SC17	–	F2 (783)	PI 96983	Yang et al. (2013)
		BARCSOYSSR_13_1136	30,464,888	Greenhouse Screening SMV Strains SC3, SC6, SC7, SC17	–	F2 (783)	PI 96983	Yang et al. (2013)
		BARCSOYSSR_13_1140	30,501,849	Greenhouse Screening SMV Strains SC3, SC6, SC7, SC17	–	F2 (783)	PI 96983	Yang et al. (2013)
		BARCSOYSSR_13_1155	30,880,128	Greenhouse Screening SMV Strains SC3, SC6, SC7, SC17	–	F2 (783)	PI 96983	Yang et al. (2013)
		Satt510	31,802,676	Greenhouse Screening SMV Strain G1	–	F2 (1056)	PI 96983	Gore et al. (2002)
	*Rsv1-h*	–	–	Greenhouse Screening SMV Strains G1, G5, G6, G7, G7A	–	F2 (794)	Suweon 97	Chen et al. (2002)
	*Rsv1-r*	–	–	Greenhouse Screening SMV Strains G1-G7	–	F2:3 (1041)	Raiden	Chen et al. (2001)
	*Rsv1-k*	–	–	Greenhouse Screening SMV Strain G1	–	F2:3 (1133)	Kwanggyo	Chen et al. (1991)
	*Rsv1-t*	–	–	Greenhouse Screening SMV Strain G1	–	F2:3 (1133)	Ogden	Chen et al. (1991)
	*Rsv1-m*	–	–	Greenhouse Screening SMV Strain G1	–	F2:3 (1133)	Marshall	Chen et al. (1991)
	*Rsv1-n*	–	–	Greenhouse Screening SMV Strains G1 and G6	–	F2:3 (239)	PI 507389	Ma et al. (2003)
	*Rsv5*	Satt114	28,912,864	Greenhouse Screening SMV Strain G1	–	F2:3 (3000)	York	Klepadlo et al. (2017)
	*Rsc3*	BARCSOYSSR_13_1128	28,919,973	Greenhouse Screening SMV Strain SC3	–	F2 (783)	PI 96983	Yang et al. (2013)
		BARCSOYSSR_13_1136	29,264,742					
	*Rsc14Q*	Satt334	29,609,521	Greenhouse Screening SMV Strain SC14	–	F7 (231)	Qihuang No. 1	Bai et al. (2009), Ma et al. (2011)
		MY750	29,594,566					
	*Rsc15ZH*	–	27,801,314	Greenhouse Screening SMV Strain SC15	–	F8 (163)	Zhonghuang24	Li et al. (2020)
		–	27,864,011					
	*qSMV13*	ss715614844	29,741,893	Greenhouse Screening SMV strain SC3	8.1%	Cultivars (302) and landraces (77)	–	Chu et al. (2021b)
		ss715614844–ss715614864	29,741,893–29,839,120	Greenhouse Screening SMV strains SC3 and SC7	71.2–76.6%	F6:8 (193)	Kennong7	Chu et al. (2021b)
MLG B2 (Chr. 14)	–	ss715617664	13,092,389	Greenhouse Screening SMV strain SC3	19.0%	Cultivars (302) and landraces (77)	–	Chu et al. (2021b)
	*Rsv3*	–	–	–	–	–	OX 686	Buzzell and Tu (1989)
		Barc-012953–00,413	45,086,977	Greenhouse Screening SMV Strains G1-G7	–	Diverse Genotypes (47)	–	Shi et al. (2011)
		A519-snp2	46,937,343	Greenhouse Screening SMV Strains G1-G7	–	Diverse Genotypes (47)	–	Shi et al. (2011)
		A519-snp4	46,937,465	Greenhouse Screening SMV Strains G1-G7	–	Diverse Genotypes (47)	–	Shi et al. (2011)
		Satt063	45,993,857	Greenhouse Screening SMV Strains G5-G7	–	F2:3 (195)	L29, Tousan 140	Jeong et al. (2002)
		A519-f/r	46,937,953	Greenhouse Screening SMV Strains G5-G7	–	F2:3 (195)	L29, Tousan 140	Jeong et al. (2002)
		M3aSatt	47,090,758	Greenhouse Screening SMV Strains G5-G7	–	F2:3 (195)	L29, Tousan 140	Jeong et al. (2002)
		BARCSOYSSR_14_1413	46,944,330	Greenhouse Screening SMV Strains SC4	–	F2 (1047)	Dabaima	Wang et al. (2011)
		BARCSOYSSR_14_1416	47,007,588	Greenhouse Screening SMV Strains SC4	–	F2 (1047)	Dabaima	Wang et al. (2011)
		-	–	Greenhouse Screening SMV Strains G6 and G7	–	F2:3 (472)	Harosoy	Gunduz et al. (2001)
	*Rsv3-n*	Satt534	45,051,723	Greenhouse Screening SMV Strains G1 and G7	–	F2 (273) F2:3 (196)	PI 61944	Cervantes-Martinez et al. (2015)
	*Rsv3-h*	Satt063	45,993,857	Greenhouse Screening SMV Strains G7	–	F2 (616) F2:3 (289)	PI 61947	Shakiba et al. (2012a, b)
	*Rsv3-c*	Satt063	45,993,857	Greenhouse Screening SMV Strains G7	–	F2 (616) F2:3 (289)	PI 399091	Shakiba et al. (2012a, b)
MLG J (Chr. 16)	-	ss715625254	6,042,142	Greenhouse Screening SMV strain SC3	6.0%	Cultivars (302) and landraces (77)	–	Chu et al. (2021b)

^a^Marker position (bp) based on the *Glycine max* genome assembly version *Gmax2.0*

^b^Phenotypic variations explained by the molecular markers

*Rsv1* is the first SMV resistance locus identified and was mapped on Chr. 13 (MLG F). It represents the most common resistance locus in soybean germplasm (Kiihl and Hartwig 1979), conferring resistance to less virulent strains (G1-G3) and susceptibility to more virulent strains (G5-G7). A total of ten unique alleles have been identified including *Rsv1, Rsv1-t, Rsv1-y, Rsv1-m, Rsv1-k, Rsv1-r, Rsv1-s, Rsv1-n*, *Rsv1-h,* and *Rsv1-c* (Kiihl and Hartwig 1979; Roane et al. 1983; Chen et al. 1991, 2001, 2002; Shakiba et al. 2013). *Rsv3* was mapped on Chr. 14 (MLG B2) and confers resistance to more virulent strains (G5-G7) while susceptible to less virulent strains (G1–G4) (Tu and Buzzell 1987). The *Rsv3* locus contains at least six alleles identified in ‘OX686’, ‘Harosoy’, ‘L29’, PI 61944, PI 61947, and PI 399091 (Buzzell and Tu 1989; Buss et al. 1999; Gunduz et al. 2001; Shakiba et al. 2012b; Cervantes-Martinez et al. 2015). *Rsv4* was mapped on Chr. 2 (MLG D1b) and confers complete resistance to all SVM strains (Buss et al. 1997; Ma et al. 2002; Gunduz et al. 2004). A total of four alleles have been identified from ‘V94-5152’, PI 88788, and ‘Beeson’ (Buss et al. 1997; Ma et al. 2002; Gunduz et al. 2004; Shakiba et al. 2013). Since the reaction (hypersensitive reaction) observed in *Rsv1* and *Rsv3* is different from that in *Rsv4*, it is suggested that *Rsv4* has unique molecular defense mechanisms (Ma et al. 2002; Gunduz et al. 2004; Saghai Maroof et al. 2008). Recently, Klepadlo et al. (2017) suggested that *Rsv1-y* should be named as an independent locus *Rsv5* because of segregation in resistance to SMV in progenies derived from PI 96983 (*Rsv1*) and ‘York’ (*Rsv1-y*).

In addition to *Rsv1*, *Rsv3*, *Rsv4*, and *Rsv5*, several other genes named *Rsc5* (Karthikeyan et al. 2017)*, Rsc7* (Yan et al. 2015)*,* and *Rsc8* (Zhao et al. 2016) have been mapped on Chr. 2 (MLG D1b), and *Rsc3* (Yang et al. 2013)*, Rsc14Q* (Ma et al. 2011) and *Rsc15ZH* (Li et al. 2020) on Chr. 13 (LG F) for resistance to Chinese SMV strains. Due to differences in SMV strain classification systems between USA and China, likely *Rsc3*, *Rsc14Q* and *Rsc15ZH* share the same locus of *Rsv1* whereas *Rsc5, Rsc7, Rsc8* share the same locus as *Rsv4* (Table 18)*.* Although rare, the combination of the four resistance loci is naturally available in soybean genotypes and can be achieved through gene pyramiding. Combining multiple resistance genes may provide more effective and durable resistance and minimize the occurrence of resistance-breaking emerging populations.

## Alfalfa mosaic virus

Alfalfa mosaic virus (AMV) is a member of the genus *Alfamovirus* in the family Bromoviridae. It has a worldwide distribution and infects more than 600 species in 22 dicotyledonous families, including agriculturally valuable crops such as alfalfa, tomato, lettuce, potato, soybean, and common bean. AMV is transmitted by more than 15 species of aphids, including the soybean aphid [*Aphis glycines* Matsumura (Hemiptera: Aphididae)], in a nonpersistent manner. It is also transmitted by mechanical inoculation and in some species, such as alfalfa and in reduced values in soybean, through the seed (Truol et al. 1985; Clark and Perry 2002; Hartman et al. 2016). Seed transmissibility was proven to be virus strain and host genotype-dependent in soybean (He et al. 2010).

AMV is known as a very complex virus which has four bacilliform particles, elongated with rounded ends. The particles are 18 nm in diameter and 30, 34, 43, and 56 nm in length. The viral genome consists of three single strands of RNA (2.0, 2.6, and 3.6 kb in length) and a fourth sub-genomic RNA, known as RNA 4 encoding the coat protein (Hartman et al. 2016; Loesch-Fries 2021).

Symptoms caused by AMV in soybean range from mosaic to mottle patterns of contrasting mixes of bright yellow and dark. It is often referred to as a calico or flashy mosaic. Leaf malformation, stunting, reduced pod set, and seed coat mottling have also been mentioned. Depending upon soybean genotype, environmental conditions and strain of the virus involved, symptoms can either persist or disappear in the new tissues of infected plants (Mueller et al. 2007; Hartman et al. 2016).

Synergism between AMV and SMV has been reported. AMV symptoms are more severe and persist throughout the season in plants infected by both viruses. The observation that co-infection of AMV and SMV results in disease synergism suggests enhancement of potential that AMV may become a serious viral disease of soybean (Malapi-Nelson et al. 2009).

Recommended management strategies include selection of resistant cultivars and the use of clean virus-free seed. Resistance to AMV in the Brazilian cultivars ‘Pérola’ and ‘Planalto’ and their common ancestor ‘Hood’ was reported to be controlled by a single dominant gene (Almeida et al. 1982). Two cultivars, ‘Wuyuezha’ and ‘Baimaodou’, were described as tolerant in China (Che et al. 2020). In the USA, resistance to AMV was found in PI 153282. Genetic studies revealed the existence of one dominant gene, which was named *Rav1*, and DNA marker analysis allowed its location on a genetic map (Kopisch-Obuch et al. 2008) (Table 19).

**Table 19:** Soybean loci conferring resistance to other soybean viruses

Causal agent	MLG (Chr.)	Locus name	Tightly linked markers	Marker position cM (bp)^a^	Testing methods/Resistance spectrum	Population type (size)	PVE^b^	Donor source	References
*Alfalfa mosaic virus* (AMV)	MLG J (Chr. 16)	*Rav1*	Sat_228	23,91 cM (3,049,971)	Greenhouse test/AMV-C field isolate, Wiconsin, USA (2001)	F4:7 (174)	79%	PI 153282	Kopisch-Obuch et al. (2008)
Soybean dwarf virus (SbDV)	MLG A1 (Chr.5)	*Rsdv1*	Sat_11 and Sct_13	–	Greenhouse and field tests	F6 (289)	79%	Wilis	Uchibori et al. (2009), Yamashita et al. (2013)
	MLG N (Chr.3)	*Raso1*	Gm03-11 and Gm03-12	(4,661,084–4,724,159)	*Raso1* confers resistance to foxglove aphid, but require additional genes for tolerance to SbDV	F2 (669, 576)	32% to aphid	Adams (PI 548502)	Ohnishi et al. (2012)

^a^Marker position (bp) based on the *Glycine max* genome assembly version *Gmax1.01*

^b^Phenotypic variations explained by the molecular markers

## Bean pod mottle virus

Bean pod mottle virus (BPMV), a member of genus *Comovirus* in the family *Comoviridae*, is a major viral pathogen of soybean first identified in Arkansas in 1951 (Walters 1958). The adult bean leaf beetle, *Cerotoma trifurcate* Forster (Coleoptera: Chrysomelidae), has been known as a main vector of BPMV, but it is also a destructive insect feeding on leaves, stems, and pods in soybean production regions in the USA (Pedigo and Zeiss 1996; Giesler et al. 2002). Plant responses to this pathogen can range from mild chlorotic mottling to severe mosaic on younger soybean leaves co-occurring with green stem symptoms (Giesler et al. 2002; Zheng et al. 2005b; Rodriguez and Thiessen 2020). BPMV can also cause plant stunting, leaf distortion, wilting, and reduced pods per plant and seed size and quality under severe infection (Myhre et al. 1973; Schwenk and Nickell 1980; Giesler et al. 2002). Soybean yield reductions resulting from BPMV infection have been reported as high as 52% (Hopkins and Mueller 1984; Gergerich 1999), and it can be maximized by the infection before V6 stage (Fehr et al. 1971) or the co-infections with soybean mosaic virus (Ross 1968; Rodriguez and Thiessen, 2020). Although Ross (1986) developed and released four BPMV-resistant soybean germplasm lines, these lines showed mild symptoms with systemic infections, and there is still no commercial soybean variety with BPMV resistance (Zheng et al. 2005b; Rodriguez and Thiessen 2020). Genetic loci for BPMV resistance have not been thoroughly investigated in soybean, but several studies have successfully engineered BPMV resistance in transgenic soybean plants by overexpressing ds-specific ribonuclease gene *PAC1* (RNase III family) from *Schizosaccharomyces pombe* and RNAi-based strategies (Reddy et al. 2001; Zhang et al. 2011; Yang et al. 2019). However, with previously identified 15 *G. soja* and 12 *G. tomentella* lines showing tolerance with mild symptoms or no systematic infection to BPMV, soybean breeders may want to incorporate those useful genetic sources into *G. max* by interspecific crosses for further investigation in specific loci and molecular marker development in soybean (Zheng et al. 2005b). The virus infection assay for BPMV was well described by Zheng et al. (2005b) using four diverse isolates (*K-G7*, *K-Ha1*, *K-Ho1*, and *AR*) and enzyme-linked immunosorbent assay (ELISA).

## Soybean vein necrotic virus

Soybean vein necrosis virus (SVNV) was first reported in Arkansas and Tennessee in 2008 (Tzanetakis et al. 2009) and is now found in 22 states in the USA as well as in Canada and Egypt (Zhou 2012; Ali and Abdalla 2013; Conner et al. 2013; Han et al. 2013; Jacobs et al. 2013; Smith et al. 2013; Kleczewski 2016; Abd El-Wahab and El-Shazly 2017; Escalante et al. 2018). It is now the most prevalent virus in North America (Zhou and Tzanetakis 2013). Symptoms caused by SVNV begin as clearing of the main leaflet veins that progressively become necrotic. When severe, these symptoms can expand to encompass the entire leaflet (Tzanetakis et al. 2009). Seeds of plants infected by SVNV can have lower oil and protein content (Groves et al. 2016; Anderson et al. 2017), with higher levels of linoleic acid and lower levels of oleic acid (Anderson et al. 2017). It is not known if the virus reduces overall yields. There is evidence of seed transmission (Groves et al. 2016), but SVNV is vectored primarily by thrips which transmit SVNV in a persistent and propagative manner (Zhou et al. 2013). The primary thrips vector is *Neohydatothrips variabilis*, but the thrips *Frankliniella tirtici* and *F. fusca* also transmit the virus at lower rates (Zhou et al. 2018).

Two studies have identified resistance related to SVNV. Zhou et al. (2020) compared the feeding preferences of *N. variabilis* on 11 soybean accessions and suggested breeders consider PI 547422 as a source of resistance. In a more recent study, seven soybean genotypes were inoculated under controlled conditions using SVNV-infected thirps (*N. variabilis*), and their results suggested that the genotypes ‘51–23’, ‘91–38’, and ‘SSR51-70’ were resistant to SVNV and 51–23 was tolerant (some symptom development, but very low virus titer) (Zambrana-Echevarria 2021).

An alternative mechanism to control SVNV is blocking the vector-virus interaction via synthetic glycopeptides that compete with SVNV glycopeptides to reduce transmission of SVNV by *N. variabilis* (Zhou and Tzanetakis 2020). These peptides reduced the transmission of SVNV by at least 50% (Zhou and Tzanetakis 2020).

## Soybean dwarf virus

Soybean dwarf virus (SbDV) was first noticed in Hokkaido in 1969 and remains a major soybean yield suppressor in northern Japan (Tamada et al. 1969; Harrison et al. 2005). The symptoms of SbDV include dwarfing (stunting), downward curling, rugosity, and interveinal yellowing of the leaves. *Rsdv1* is the only gene known to confer major resistance to SbDV (Uchibori et al. 2009; Yamashita et al. 2013). Another gene, *Raso1*, was found conferring resistance to foxglove aphid, a transmission vector of SbDV, but a further study indicated that *Raso1* needs at least one additional gene for resistance to SbDV (Table 19) (Ohnishi et al. 2012).

## Conclusions and future perspectives

With the identification and implementation of molecular markers tightly linked with resistance genes, the introgression of vertical resistance through MAS became a practice routinely performed by public and private soybean breeding programs. Efforts to understand minor genes with small but accumulative effects for horizontal resistance will also be needed. What’s more, to expand the sources of resistance and discover resistance genes and QTLs to ensure the sustainability of soybean production, continuous efforts are needed to screen diverse germplasm lines. For example, the USDA Germplasm Collection (GRIN) provides more than 20,000 soybeans accessions worldwide and more resistance sources can be expected to be identified. Germplasm lines and elite soybean cultivars with resistance to multiple diseases combined with high-yielding potential and desired agronomic traits are being developed. In addition, interaction among resistance loci, allelic and copy number variations, their interactions with environment, and impact on virulence of pathogens and disease development deserve close attention in future research.

Advances in genomics facilitated the introduction of next generation sequencing (NGS)-based high-density molecular markers which are quickly evolved and became available at an accessible cost for both public and private breeding programs (Song et al. 2013, 2020). Genome-wide studies revealed many novel regions of the soybean genome significantly associated with resistance to different pathogens, and traits that were often considered qualitative in nature evolved to some extent into quantitative traits with major and minor alleles with small effects contributing to the observed phenotypes. The rise of digitally smart-agriculture and the application of machine learning and artificial intelligence for characterizing the response of breeding lines to specific diseases represented another breakthrough in breeding for genetic resistance. Disease assessment screening protocols often reported on categorical scales based on subjective ratings are gradually being replaced by precise quantitative metrics representing the observed phenotypes (Gazala et al. 2013; Khalili et al. 2020; Gui et al. 2021; Liu et al. 2021). In combination with advanced predictive analytics and mega environmental data, one can predict the response of soybean breeding lines to specific or multiple diseases in diverse environments, which can be a powerful tool to anticipate the deployment of resistant cultivars to potential disease outbreaks and extreme environmental conditions.

Throughout this review, the impact of pathogens in global soybean production and their respective yield losses have been discussed. Substantial yearly production losses in the order of billions of dollars due to diseases have been repeatedly reported in the literature for decades (Wrather et al. 1997; 2001; Allen et al. 2017; Savary et al. 2019; Bandara et al. 2020). Genetic resistance is the most effective and sustainable approach for the disease management in soybean globally, representing a critical pillar bolstering the global soybean value chain and food security. Although hundreds of significant genomic regions conferring resistance to multiple pathogens have been reported in this review, there are many components of genetic resistance still to be enlightened and continuously investigated. For instance, limited advancements have been achieved in understanding the pathogen infectious dynamics and underlying genetic regulations. The substantial shift and emergence of novel and/or resistance-breaking strains and emergence of pathogen races impose a threat to previously validated resistance genes. In addition, the pleiotropic effect of resistance genes and the interaction among those in terms of durable broad-based resistance levels, yield penalty, as well as environmental interactions are now becoming critically important due to the availability of big genomic data and emergence of advanced analytical algorithms (Patil et al. 2019).

Whole genome resequencing facilitated the characterization of diverse lines with superior haplotypes or alleles among unexplored germplasm which could be used to deploy durable resistance in plant breeding program. The future breeding era is likely to be genomics-assisted breeding (GAB) including marker-assisted recurrent selection (MARS), marker-assisted backcrossing (MABC), haplotype-based breeding, and genomic selection (GS) (Varshney et al. 2021). Trait-associated genes would be mapped with NGS-based trait mapping and system biology approach. Future genetic variations can be estimated by targeting induced local lesions in genomes (TILLING), Eco-TILLING populations, and multiparent advanced generation intercross (MAGIC) or can be created through genome/gene editing (GE). GE has been emerged at an unprecedented speed and probably become a primary technique for translating genomic information to improvement of the crop in the field. However, the success of the development of CRISPR/Cas9 transformants is subject to effective genetic transformation system. Unfortunately, soybean is a recalcitrant crop for plant transformation technology and most of the GE studies are in primary phase of development. Although a few studies have successfully show the introduction of Cas12a-RNP in soybean protoplast (Kim et al. 2017), enormous efforts may be needed to implement these tools into soybean.

All in all, the early establishment of the soybean research field, the vast availability of unexplored genetic diversity through soybean accessions, the breakthrough advancements in genomics and analytics, and the dynamism of the environment, pathogens, and host genetic background will significantly improve the efficiency and accuracy of global soybean breeding in the next decades, ensuring the sustainability and growth of soybean production worldwide.

## Supplementary Information

Below is the link to the electronic supplementary material.

Supplementary file1 (DOCX 63 kb)

Supplementary file2 (DOCX 51 kb)

Supplementary file3 (DOCX 40 kb)

## References

[CR1] Abad P, Gouzy J, Aury JM (2008). Genome sequence of the metazoan plant-parasitic nematode *Meloidogyne incognita*. Nat Biotechnol.

[CR2] Abd El-Wahab AS, El-Shazly MA (2017). Identification and characterization of soybean vein necrosis virus (SVNV): a newly isolated thrips-borne tospovirus in Egypt. J Virol Sci.

[CR3] Abdelmajid KM, Meksem K, Wood AJ, Lightfoot DA (2007). Loci underlying SDS and SCN resistance mapped in the ‘Essex’by ‘Forrest’ soybean recombinant inbred lines. Rev Biol Biotechnol.

[CR4] Abdelmajid KM, Ramos L, Leandro L (2012). The ‘PI 438489B’by ‘Hamilton’SNP-based genetic linkage map of soybean [Glycine max (L.) Merr.] identified quantitative trait loci that underlie seedling SDS resistance. J Plant Genome Sci.

[CR5] Abdelmajid KM, Ramos L, Hyten DL, Bond J (2014). Quantitative trait loci (QTL) that underlie SCN resistance in soybean [Glycine max (L.) Merr.] PI438489B by ‘Hamilton’recombinant inbred line (RIL) population. Atlas J Plant Biol.

[CR6] Abeysekara NS, Matthiesen RL, Cianzio SR (2016). Novel sources of partial resistance against *Phytophthora sojae* in soybean PI 399036. Crop Sci.

[CR7] Acharya B, Lee S, Mian MR (2015). Identification and mapping of quantitative trait loci (QTL) conferring resistance to *Fusarium graminearum* from soybean PI 567301B. Theor Appl Genet.

[CR8] Ajayi-Oyetunde OO, Bradley CA (2017). Identification and characterization of *Rhizoctonia* species associated with soybean seedling disease. Plant Dis.

[CR9] Ajayi-Oyetunde OO, Bradley CA (2018). *Rhizoctonia solani*: taxonomy, population biology and management of rhizoctonia seedling disease of soybean. Plant Pathol.

[CR10] Akem CN, Dashiell KE (1994). Effect of planting date on severity of frogeye leaf spot and grain yield of soybeans. Crop Prot.

[CR12] Ali A, Abdalla OA (2013). First report of Soybean vein necrosis virus in soybean fields of Oklahoma. Plant Dis.

[CR13] Allen TW, Bradley CA, Sisson AJ (2017). Soybean yield loss estimates due to diseases in the United States and Ontario, Canada, from 2010 to 2014. Plant Health Prog.

[CR14] Allen TW, Bissonnette K, Bradley CA, et al (2020) Southern United States soybean disease loss estimates for 2019. Southern Soybean Disease Workers (SSDW). https://www.mssoy.org/uploads/files/allen-dis-loss-survey-2019.pdf

[CR15] Allington WB, Chamberlain DW (1948). Brown stem rot of soybean. Phytopathology.

[CR16] Alloatti J, Li S, Chen P (2015). Screening a diverse soybean germplasm collection for reaction to purple seed stain caused by *Cercospora kikuchii*. Plant Dis.

[CR17] Almeida AMR, Kiihl RAS, Almeida LA (1982). Calico mosaic of soybean: Sources of resistance and inheritance of reaction. Soybean Genet Newsl.

[CR705] Almeida ÁM, Amorim L, Bergamin Filho A (2003). Progress of soybean charcoal rot under tillage and no-tillage systems in Brazil. Fitopatol Bras.

[CR18] Almeida AMR, Piuga FF, Marin SRR (2005). Pathogenicity, molecular characterization, and cercosporin content of Brazilian isolates of *Cercospora kikuchii*. Fitopatol Bras.

[CR19] Anand SC, Rao-Arelli AP (1989). Genetic analyses of soybean genotypes resistant to soybean cyst nematode race 5. Crop Sci.

[CR20] Anderson J, Akond M, Kassem MA (2015). Quantitative trait loci underlying resistance to sudden death syndrome (SDS) in MD96–5722 by ‘Spencer’ recombinant inbred line population of soybean. 3 Biotech.

[CR21] Anderson NR, Irizarry MD, Bloomingdale CA (2017). Effect of soybean vein necrosis on yield and seed quality of soybean. Can J Plant Pathol.

[CR22] Anderson TR, Welacky TW, Olechowski HT (1988). First report of *Heterodera glycines* on soybeans in Ontario. Canada Plant Dis.

[CR23] Anderson TR, Buzzell RI (1992). Inheritance and linkage of the *Rps7* gene for resistance to Phytophthora rot of soybean. Plant Dis.

[CR24] Anderson TR, Tenuta AU (1998). First report of Fusarium solani f. sp. glycines causing sudden death syndrome of soybean in Canada. Plant Dis.

[CR25] Aoki T, O’Donnell K, Homma Y, Lattanzi AR (2003). Sudden-death syndrome of soybean is caused by two morphologically and phylogenetically distinct species within the *Fusarium solani* species complex—*F. virguliforme* in North America and *F. tucumaniae* in South America. Mycologia.

[CR26] Aoki T, O’Donnell K, Scandiani MM (2005). Sudden death syndrome of soybean in South America is caused by four species of *Fusarium*: *Fusarium brasiliense* sp. nov., *F. cuneirostrum* sp. nov., *F. tucumaniae*, and *F. virguliforme*. Mycoscience.

[CR27] Aoki T, Scandiani MM, O’Donnell K (2012). Phenotypic, molecular phylogenetic, and pathogenetic characterization of *Fusarium crassistipitatum* sp. nov., a novel soybean sudden death syndrome pathogen from Argentina and Brazil. Mycoscience.

[CR28] Arahana VS, Graef GL, Specht JE (2001). Identification of QTLs for resistance to *Sclerotinia sclerotiorum* in soybean. Crop Sci.

[CR29] Arias MMD, Leandro LF, Munkvold GP (2013). Aggressiveness of *Fusarium* species and impact of root infection on growth and yield of soybeans. Phytopathology.

[CR30] Ashfield T, Danzer JR, Held D (1998). *Rpg1*, a soybean gene effective against races of bacterial blight, maps to a cluster of previously identified disease resistance genes. Theor Appl Genet.

[CR31] Ashfield T, Ong LE, Nobuta K (2004). Convergent evolution of disease resistance gene specificity in two flowering plant families. Plant Cell.

[CR32] Ashfield T, Redditt T, Russell A (2014). Evolutionary relationship of disease resistance genes in soybean and Arabidopsis specific for the *Pseudomonas syringae* effectors AvrB and AvrRpm1. Plant Physiol.

[CR33] Astudillo GE, Birchfield W (1980). Pathology of *Hoplolaimus columbus* on sugarcane. Phytopathology.

[CR34] Athow K, Probst AH (1952). The inheritance of resistance to frog-eye leaf spot of soybeans. Phytopathology.

[CR35] Athow KL, Caldwell RM (1954). A comparative study of Diaporthe stem canker and pod and stem blight of soybean. Phytopathology.

[CR36] Athow KL, Probst AH, Kurtzman CP, Laviolette FA (1962). A newly identified physiological race of *Cercospora sojina* on soybean. Phytopathology.

[CR37] Athow KL, Laviolette FA, Mueller EH, Wilcox JR (1980). A new major gene for resistance to *Phytophthora megasperma var. sojae* in soybean. Phytopathology.

[CR38] Athow KL, Laviolette FA (1982). *Rps6*, a major gene for resistance to *Phytophthora megasperma f. sp. glycinea* in soybean. Phytopathology.

[CR39] Athow KL, Laviolette FA, Layton Hahn AC, Ploper LD (1986). United States: Genes for resistance to Phytophthora megasperma f. sp. glycinea in PI 273483D, PI 64747, PI 274212, PI 82312N, and PI 340046. Soybean Genet Newsl.

[CR40] Athow KL, Wilcox JR (1987). Fungal diseases. Soybeans: improvement, production, and uses.

[CR41] Bachman MS, Tamulonis JP, Nickell CD, Bent AF (2001). Molecular markers linked to brown stem rot resistance genes, *Rbs1* and *Rbs2*, in soybean. Crop Sci.

[CR42] Backman PA, Weaver DB, Morgan-Jones G (1985). Soybean stem canker: an emerging disease problem. Plant Dis.

[CR43] Bai L, Li HC, Ma Y (2009). Inheritance and gene mapping of resistance to soybean mosaic virus strain *SC11* in soybean. Soybean Sci.

[CR44] Bandara AY, Weerasooriya DK, Bradley CA (2020). Dissecting the economic impact of soybean diseases in the United States over two decades. PLoS ONE.

[CR45] Bao Y, Vuong T, Meinhardt C (2014). Potential of association mapping and genomic selection to explore PI 88788 derived soybean cyst nematode resistance. Plant Genome.

[CR46] Bao Y, Kurle JE, Anderson G, Young ND (2015). Association mapping and genomic prediction for resistance to sudden death syndrome in early maturing soybean germplasm. Mol Breed.

[CR47] Bastien M, Sonah H, Belzile F (2014). Genome wide association mapping of Sclerotinia sclerotiorum resistance in soybean with a genotyping-by-sequencing approach. Plant Genome.

[CR48] Bates GD, Rothrock CS, Rupe JC, Chen P (2004). Resistance in soybean cultivars to Pythium damping-off and root rot. Phytopathology.

[CR49] Bates GD, Rothrock CS, Rupe JC (2008). Resistance of the soybean cultivar Archer to Pythium damping-off and root rot caused by several *Pythium* spp. Plant Dis.

[CR50] Bernard RL, Smith PE, Kaufmann MJ, Schmitthenner AF (1957). Inheritance of resistance to Phytophthora root and stem rot in the soybean. Agron J.

[CR51] Bernard RL, Cremeens CR (1971). A gene for general resistance to downy mildew of soybeans. J Hered.

[CR52] Bernard RL, Weiss MG, Caldwell BE (1973). Qualitative genetics. Soybeans: improvement, production, and uses.

[CR53] Bernard RL, Cremeens CR (1981). An allele at the *rps1* locus from the variety ‘Kingwa’. Soybean Genet Newsl.

[CR54] Bernaux P (1979). Identification of some soybean diseases in Cameroon. Agron Trop.

[CR55] Bhattacharyya MK, Narayanan NN, Gao H (2005). Identification of a large cluster of coiled coil-nucleotide binding site–leucine rich repeat-type genes from the *Rps1* region containing Phytophthora resistance genes in soybean. Theor Appl Genet.

[CR56] Bienapfl JC, Malvick DK, Percich JA (2011). Specific molecular detection of *Phytophthora sojae* using conventional and real-time PCR. Fungal Biol.

[CR57] Blagden T, Espindola A, Cardwell K (2019). Draft genome sequences of three isolates of *Coniothyrium glycines*, causal agent of red leaf blotch of soybean. Microbiol Resour Announc.

[CR58] Boerma HR, Phillips DV (1983). Genetic implications of the susceptibility of Kent soybean to *Cercospora sojina*. Phytopathology.

[CR59] Bonde MR, Nester SE, Austin CN (2006). Evaluation of virulence of Phakopsora pachyrhizi and *P. meibomiae* isolates. Plant Dis.

[CR60] Bos L (1972) Soybean mosaic virus. CMI/AAB Descriptions of plant viruses 93

[CR61] Boudhrioua C, Bastien M, Torkamaneh D, Belzile F (2020). Genome-wide association mapping of *Sclerotinia sclerotiorum* resistance in soybean using whole-genome resequencing data. BMC Plant Biol.

[CR62] Bowers TC, Hartwig EE (1987). Identification of single genes controlling resistance to stem canker in Soybean 1. Crop Sci.

[CR63] Bowers GR, Ngeleka K, Smith O (1993). Inheritance of stem canker resistance in soybean cultivars Crockett and Dowling. Crop Sci.

[CR64] Bradley CA, Hartman GL, Nelson RL (2001). Response of ancestral soybean lines and commercial cultivars to Rhizoctonia root and hypocotyl rot. Plant Dis.

[CR65] Bradley CA, Allen T, Sisson AJ (2021). Soybean yield loss estimates due to diseases in the United States and Ontario, Canada from 2015–2019. Plant Health Prog.

[CR66] Broders KD, Lipps PE, Paul PA, Dorrance AE (2007). Evaluation of *Fusarium graminearum* associated with corn and soybean seed and seedling disease in Ohio. Plant Dis.

[CR67] Brogin RL (2005) Mapping resistance genes to soybean rust and QTLs involved in brown spot resistance in soybean. Tese - Escola Superior de Agricultura Luiz de Queiroz 93

[CR68] Bromfield KR (1984) Soybean rust, No. 11 monograph, American Phytopathological Society, St. Paul, Minnesota, pp 65

[CR69] Brown EA, Minor HC, Calvert OH (1987). A soybean genotype resistant to Phomopsis seed decay. Crop Sci.

[CR70] Brucker E, Carlson S, Wright E (2005). *Rhg1* alleles from soybean PI 437654 and PI 88788 respond differentially to isolates of *Heterodera glycines* in the greenhouse. Theor Appl Genet.

[CR71] Brzostowski LF, Pruski TI, Hartman GL (2018). Field evaluation of three sources of genetic resistance to sudden death syndrome of soybean. Theor Appl Genet.

[CR72] Burnham KD, Dorrance AE, Francis DM (2003). *Rps8*, a new locus in soybean for resistance to *Phytophthora sojae*. Crop Sci.

[CR73] Burnham KD, Dorrance AE, VanToai T, St Martin S (2003). Quantitative trait loci for partial resistance to *Phytophthora sojae* in soybean. Crop Sci.

[CR74] Bushnev A, Babenko S, Bushneva N (2020) The application efficiency of organic fungicides against soybean diseases in the central zone of the Krasnodar region of the Russian Federation. In: E3S Web of Conferences 222: p. 02020, EDP Sciences

[CR75] Buss GR, Ma G, Chen P, Tolin SA (1997). Registration of V94–5152 Soybean germplasm resistant to *Soybean Mosaic Potyvirus*. Crop Sci.

[CR76] Buss GR, Ma G, Kristipati S, et al (1999) A new allele at the *Rsv3* locus for resistance to soybean mosaic virus. In: Proceedings of World Soybean Res. Conf. VI, Chicago, Illinois. Champaign, Illinois: Superior Printing, pp 4–7

[CR77] Buzzell RI, Anderson TR (1981). Another major gene for resistance to *Phytophthora megasperma var. sojae* in soybean. Soybean Genet Newsl.

[CR78] Buzzell RI, Tu JC (1984). Inheritance of soybean resistance to soybean mosaic virus. J Hered.

[CR79] Buzzell RI, Tu JC (1989). Inheritance of a soybean stem-tip necrosis reaction to soybean mosaic virus. J Hered.

[CR80] Buzzell RI, Anderson TR (1992). Inheritance and race reaction of a new soybean *Rps1* allele. Plant Dis.

[CR81] Cai G, Schneider RW, Padgett GB (2009). Assessment of lineages of *Cercospora kikuchii* in Louisiana for aggressiveness and screening soybean cultivars for resistance to Cercospora leaf blight. Plant Dis.

[CR82] Caldwell BE, Brim CA, Ross JP (1960). Inheritance of resistance of soybeans to the cyst nematode, *Heterodera Glycines* 1. Agron J.

[CR83] Cervantes-Martinez I, Chen P, Orazaly M, Klepadlo M (2015). Identification of a New Allele at the *Rsv3* locus for resistance to soybean mosaic virus in PI 61944 Soybean Accession. Crop Sci.

[CR84] Chakraborty N, Curley J, Frederick RD (2009). Mapping and confirmation of a new allele at *rpp1* from soybean PI594538a conferring RB lesion-type resistance to soybean rust. Crop Sci.

[CR85] Chamberlain DW, Bernard RL (1968). Resistance to brown stem rot in soybeans. Crop Sci.

[CR86] Chang SJC, Doubler TW, Kilo V (1996). Two additional loci underlying durable field resistance to soybean sudden death syndrome (SDS). Crop Sci.

[CR87] Chang HX, Lipka AE, Domier LL, Hartman GL (2016). Characterization of disease resistance loci in the USDA soybean germplasm collection using genome-wide association studies. Phytopathology.

[CR88] Chang HX, Roth MG, Wang D (2018). Integration of sudden death syndrome resistance loci in the soybean genome. Theor Appl Genet.

[CR89] Chang HX, Wen Z, Tan R (2020). Linkage mapping for foliar necrosis of soybean sudden death syndrome. Phytopathology.

[CR90] Che X, Jiang X, Liu X (2020). First report of alfalfa mosaic virus on soybean in Heilongjiang. China Plant Dis.

[CR91] Chehri K, Salleh B, Zakaria L (2014). *Fusarium virguliforme*, a soybean sudden death syndrome fungus in Malaysian soil. Australas Plant Dis Notes.

[CR92] Chen K, Wang Y, Zhang R (2019). CRISPR/Cas genome editing and precision plant breeding in agriculture. Annu Rev Plant Biol.

[CR93] Chen L, Wang W, Ping J (2021). Identification and molecular mapping of *Rps14*, a gene conferring broad-spectrum resistance to *Phytophthora sojae* in soybean. Theor Appl Genet.

[CR94] Chen P, Buss GR, Roane CW, Tolin SA (1991). Allelism among genes for resistance to soybean mosaic virus in strain-differential soybean cultivars. Crop Sci.

[CR95] Chen P, Ma G, Buss GR (2001). Inheritance and allelism tests of Raiden soybean for resistance to soybean mosaic virus. J Hered.

[CR96] Chen P, Buss GR, Tolin SA (2002). A valuable gene in Suweon 97 soybean for resistance to soybean mosaic virus. Crop Sci.

[CR97] Chen P, Sneller CH, Mozzoni LA, Rupe JC (2007). Registration of ‘Osage’soybean. J Plant Regist.

[CR98] Chen P, Shannon G, Scaboo A (2020). Registration of ‘S14-15146GT’soybean, a high-yielding RR1 cultivar with high oil content and broad disease resistance and adaptation. J Plant Regist.

[CR99] Chen P, Shannon G, Scaboo A (2021). Registration of ‘S13-2743C’as a conventional soybean cultivar with high oil content, broad disease resistance, and high yield potential. J Plant Regist.

[CR100] Cheng P, Gedling CR, Patil G (2017). Genetic mapping and haplotype analysis of a locus for quantitative resistance to *Fusarium graminearum* in soybean accession PI 567516C. Theor Appl Genet.

[CR101] Cheng Y, Ma Q, Ren H (2017). Fine mapping of a Phytophthora-resistance gene *RpsWY* in soybean (*Glycine max* L.) by high-throughput genome-wide sequencing. Theor Appl Genet.

[CR102] Chiesa MA, Pioli RN, Morandi EN (2009). Specific resistance to soybean stem canker conferred by the *Rdm4* locus. Plant Pathol.

[CR103] Childs SP, Buck JW, Li Z (2018). Breeding soybeans with resistance to soybean rust (*Phakopsora pachyrhizi*). Plant Breed.

[CR104] Childs SP, King ZR, Walker DR (2018). Discovery of a seventh *Rpp* soybean rust resistance locus in soybean accession PI 605823. Theor Appl Genet.

[CR105] Cho E-K, Goodman RM (1979). Strains of soybean mosaic virus: Classification based on virulence in resistant soybean cultivars. Phytopathology.

[CR106] Chowdhury AK, Srinives P, Saksoong P, Tongpamnak P (2002). RAPD markers linked to resistance to downy mildew disease in soybean. Euphytica.

[CR107] Chu J, Li W, Piao D (2021). Mining of a major QTL and novel genes conferring resistance to *SC3* and *SC7* strains in soybean. Plant Breed.

[CR108] Chu J, Li W, Piao D (2021). Identification of a major QTL related to resistance to soybean mosaic virus in diverse soybean genetic populations. Euphytica.

[CR109] Cianzio SR, Shultz SP, Fehr WR, Tachibana H (1991). Registration of ‘Archer’ soybean. Crop Sci.

[CR110] Clark AJ, Perry KL (2002). Transmissibility of field isolates of soybean viruses by *Aphis glycines*. Plant Dis.

[CR111] Clevinger EM, Biyashev R, Lerch-Olson E (2021). Identification of quantitative disease resistance loci toward four *Pythium* species in soybean. Front Plant Sci.

[CR112] Concibido VC, Denny RL, Boutin SR (1994). DNA marker analysis of loci underlying resistance to soybean cyst nematode (*Heterodera glycines* Ichinohe). Crop Sci.

[CR113] Concibido VC, Lange DA, Denny RL (1997). Genome mapping of soybean cyst nematode resistance genes in Peking, PI 90763, and PI 88788 using DNA markers. Crop Sci.

[CR114] Concibido VC, Diers BW, Arelli PR (2004). A decade of QTL mapping for cyst nematode resistance in soybean. Crop Sci.

[CR115] Conner K, Sikora EJ, Zhang L, Burmester C (2013). First report of *Soybean vein necrosis-associated virus* affecting soybeans in Alabama. Plant Health Prog.

[CR116] Cook CG, Robinson AF, Namken LN (1997). Tolerance to *Rotylenchulus reniformis* and resistance to *Meloidogyne incognita* race 3 in high-yielding breeding lines of upland cotton. J Nematol.

[CR117] Cook DE, Lee TG, Guo X (2012). Copy number variation of multiple genes at *Rhg1* mediates nematode resistance in soybean. Science.

[CR118] Cook DE, Bayless AM, Wang K (2014). Distinct copy number, coding sequence, and locus methylation patterns underlie *Rhg1*-mediated soybean resistance to soybean cyst nematode. Plant Physiol.

[CR119] Coser SM, Chowda Reddy RV, Zhang J (2017). Genetic architecture of charcoal rot (*Macrophomina phaseolina*) resistance in soybean revealed using a diverse panel. Front Plant Sci.

[CR120] Cregan PB, Jarvik T, Bush AL (1999). An integrated genetic linkage map of the soybean genome. Crop Sci.

[CR121] Crittenden HW, Cole RH (1967). Registration of Delmar and Bethel soybeans. Crop Sci.

[CR122] Cummings JA, Meyer KL, Bergstorm GC (2018). First report of sudden death syndrome of soybean caused by *Fusarium virguliforme* in New York. Plant Dis.

[CR123] da Silva MP, Klepadlo M, Gbur EE (2019). QTL mapping of charcoal rot resistance in PI 567562A soybean accession. Crop Sci.

[CR124] da Silva MP, Zaccaron AZ, Bluhm BH (2020). Bulked segregant analysis using next-generation sequencing for identification of genetic loci for charcoal rot resistance in soybean. Physiol Mol Plant Pathol.

[CR125] Dashiell KE, Akem CN (1991). Yield losses in soybeans from frogeye leaf spot caused by *Cercospora sojina*. Crop Prot.

[CR126] Davis CL (2017) Identification, validation, and mapping of *Phytophthora sojae* and soybean mosaic virus resistance genes in soybean. Dissertation, Virginia Tech

[CR127] Davis E, Koenning SR, Burton JW, Barker KR (1996). Greenhouse evaluation of selected soybean germplasm for resistance to North Carolina populations of *Heterodera glycines, Rotylenchulus reniformis*, and *Meloidogyne species*. J Nematol.

[CR128] Davis EL, Tylka GL (2000). Soybean cyst nematode disease. Plant Pathol Microbiol Pub.

[CR129] de Farias Neto AL, Hashmi R, Schmidt M (2007). Mapping and confirmation of a new sudden death syndrome resistance QTL on linkage group D2 from the soybean genotypes PI 567374 and ‘Ripley’. Mol Breed.

[CR130] de Ronne M, Labbé C, Lebreton A (2020). Integrated QTL mapping, gene expression and nucleotide variation analyses to investigate complex quantitative traits: a case study with the soybean–*Phytophthora sojae* interaction. Plant Biotechnol J.

[CR131] de Ronne M, Santhanam P, Cinget B (2021). Mapping of partial resistance to *Phytophthora sojae* in soybean PIs using whole-genome sequencing reveals a major QTL. Plant Genome.

[CR132] Demirbas A, Rector B, Lohnes D (2001). Simple sequence repeat markers linked to the soybean Rps genes for Phytophthora resistance. Crop Sci.

[CR133] Diers BW, Mansur L, Imsande J, Shoemaker RC (1992). Mapping of Phytophthora resistance loci in soybean with restriction fragment length polymorphism markers. Crop Sci.

[CR134] Dorrance AE, Kleinhenz MD, McClure SA, Tuttle NT (2003). Temperature, moisture, and seed treatment effects on *Rhizoctonia solani* root rot of soybean. Plant Dis.

[CR135] Dorrance AE, Jia H, Abney TS (2004). Evaluation of soybean differentials for their interaction with *Phytophthora sojae*. Plant Health Prog.

[CR136] Dorrance AE, Berry SA, Anderson TR, Meharg C (2008). Isolation, storage, pathotype characterization, and evaluation of resistance for *Phytophthora sojae* in soybean. Plant Health Prog.

[CR137] Dorrance AE, Robertson AE, Cianzo S (2009). Integrated management strategies for *Phytophthora sojae* combining host resistance and seed treatments. Plant Dis.

[CR138] Dorrance AE, Kurle J, Robertson AE (2016). Pathotype diversity of *Phytophthora sojae* in eleven states in the United States. Plant Dis.

[CR139] Dorrance AE (2018). Management of *Phytophthora sojae* of soybean: a review and future perspectives. Can J Plant Pathol.

[CR140] Dos Santos JVM, Ferreira EGC, de Lima Passianotto AL (2019). Association mapping of a locus that confers southern stem canker resistance in soybean and SNP marker development. BMC Genom.

[CR141] Dunleavy JM (1987). Yield reduction in soyabeans caused by downy mildew. Plant Dis.

[CR142] Ellis ML, Arias MMD, Jimenez DRC (2013). First report of *Fusarium commune* causing damping-off, seed rot, and seedling root rot on soybean (*Glycine max*) in the United States. Plant Dis.

[CR143] Ellis ML, McHale LK, Paul PA (2013). Soybean germplasm resistant to *Pythium irregulare* and molecular mapping of resistance quantitative trait loci derived from the soybean accession PI 424354. Crop Sci.

[CR144] Elmore RW, Minor HC, Doupnik BL (1998). Soybean genetic resistance and benomyl for Phomopsis seed decay control. Seed Technol.

[CR145] Erwin DC, Ribeiro OK (1996). Phytophthora diseases worldwide.

[CR146] Escalante C, Bollich P, Valverde R (2018). Soybean vein necrosis virus naturally infecting yard-long bean (*Vigna unguiculata* ssp. sesquipedalis) and soybean (Glycine max) in Louisiana. Plant Dis.

[CR147] Fan A, Wang X, Fang X (2009). Molecular identification of *Phytophthora* resistance gene in soybean cultivar Yudou 25. Acta Agron Sin.

[CR148] Feaster CV (1951) Bacterial pustule disease in soybeans: artificial inoculation, varietal resistance, and inheritance of resistance. Research Bulletin 487, University of Missouri

[CR149] Fehr WR, Caviness CE, Burmood DT, Pennington JS (1971). Stage of development descriptions for soybeans, *Glycine max* (L.) Merrill. Crop Sci.

[CR150] Ferdous SA, Watanabe S, Suzuki-Orihara C (2006). QTL analysis of resistance to soybean cyst nematode race 3 in soybean cultivar Toyomusume. Breed Sci.

[CR151] Fernández FA, Philips DV, Russin JS, Rupe JC, Hartman GL, Sinclair JB, Rupe JC (1999). *Diaporthe-Phomopsis* complex. Compendium of soybean diseases.

[CR152] Fourie H, Mienie CMS, Mc Donald AH, de Waele D (2008). Identification and validation of genetic markers associated with *Meloidogyne incognita* race 2 resistance in soybean, *Glycine max* (L.) Merr. Nematol.

[CR153] Frederick RD, Snyder CL, Peterson GL, Bonde MR (2002). Polymerase chain reaction assays for the detection and discrimination of the soybean rust pathogens *Phakopsora pachyrhizi* and *P. meibomiae*. Phytopathology.

[CR154] Ganji S, Wubben MJ, Jenkins JN (2013). Two simple methods for the collection of individual life stages of reniform nematode, *Rotylenchulus reniformis*. J Nematol.

[CR155] Gao H, Narayanan NN, Ellison L, Bhattacharyya MK (2005). Two classes of highly similar coiled coil-nucleotide binding-leucine rich repeat genes isolated from the *Rps1-k* locus encode *Phytophthora* resistance in soybean. Mol Plant Microbe Interact.

[CR156] Gao H, Bhattacharyya MK (2008). The soybean-Phytophthora resistance locus *Rps1-k* encompasses coiled coil-nucleotide binding-leucine rich repeat-like genes and repetitive sequences. BMC Plant Biol.

[CR157] Gaur HS, Perry RN (1991). The role of the moulted cuticles in the desiccation survival of adults of *Rotylenchulus reniformis*. Revue Nématol.

[CR158] Garcia A, Calvo ÉS, de Souza Kiihl RA (2008). Molecular mapping of soybean rust (*Phakopsora pachyrhizi*) resistance genes: discovery of a novel locus and alleles. Theor Appl Genet.

[CR159] Garcia A, Calvo ÉS, de Souza Kiihl RA, de Souto ER (2011). Evidence of a susceptible allele inverting the dominance of rust resistance in soybean. Crop Sci.

[CR706] García-Olivares JG, López-Salinas E, Cumpián-Gutiérrez J (2012). Grain yield and charcoal rot resistance stability in common beans under terminal drought conditions. J Phytopathol.

[CR160] Garcia-Aroca T, Price PP, Tomaso-Peterson M (2021). *Xylaria necrophora*, sp. nov., is an emerging root-associated pathogen responsible for taproot decline of soybean in the southern United States. Mycologia.

[CR161] Gavilano L, Baum T, Parrott W, et al. (2013) Identification and characterization of a CLE domain-containing protein from *Rotylenchulus reniformis*. Proceedings Society of Nematologists, July 14–17, 2013, Knoxville, Tennessee

[CR162] Gazala IFS, Sahoo RN, Pandey R (2013). Spectral reflectance pattern in soybean for assessing yellow mosaic disease. Indian J Virol.

[CR163] Geeseman GE (1950). Physiologic races of *Peronospora manshurica* on soybeans. Agron J.

[CR164] Geeseman GE (1950). Inheritance of resistance of soybeans to *Peronospora manshurica*. Agron J.

[CR165] Gergerich RC, Hartman GL, Sinclair JB, Rupe JC (1999). Comoviruses: Bean pod mottle comovirus. Compendium of soybean diseases.

[CR166] Ghorbanipour A, Rabiei B, Rahmanpour S, Khodaparast SA (2019). Association analysis of charcoal rot disease resistance in soybean. Plant Pathol.

[CR167] Giesler LJ, Ghabrial SA, Hunt TE, Hill JH (2002). Bean pod mottle virus: a threat to US soybean production. Plant Dis.

[CR168] Gilli JR, Vellicce GR, Bernardi CN (2020). SSR markers linked to stem canker resistance in soybean *Glycine max*. Revista De La Facultad De Ciencias Agrarias UNCuyo.

[CR169] Gordon SG, St Martin SK, Dorrance AE (2006). *Rps8* maps to a resistance gene rich region on soybean molecular linkage group F. Crop Sci.

[CR170] Gordon SG, Kowitwanich K, Pipatpongpinyo W (2007). Molecular marker analysis of soybean plant introductions with resistance to *Phytophthora sojae*. Phytopathol.

[CR171] Gore MA, Hayes AJ, Jeong SC (2002). Mapping tightly linked genes controlling potyvirus infection at the *Rsv1* and *Rpv1* region in soybean. Genome.

[CR172] Grau CR, Dorrance AE, Bond J, Russin JS, Shibles RM, Harper JE, Wilson RF, Shoemaker RC (2004). Fungal diseases. Soybeans: improvement, production and uses.

[CR173] Gray LE (1972). Effect of *Cephalosporium gregatum* on soybean yield. Plant Dis Rep.

[CR174] Groves C, German T, Dasgupta R (2016). Seed transmission of soybean vein necrosis virus: the first *Tospovirus* implicated in seed transmission. PLoS ONE.

[CR175] Gui J, Fei J, Wu Z (2021). Grading method of soybean mosaic disease based on hyperspectral imaging technology. Inf Process Agric.

[CR176] Gunadi A (2012) Characterization of *Rps8* and *Rps3* resistance genes to *Phytophthora sojae* through genetic fine mapping and physical mapping of soybean chromosome 13. Dissertation, Ohio State University

[CR177] Gunduz I, Buss GR, Ma G (2001). Genetic analysis of resistance to soybean mosaic virus in OX670 and Harosoy soybean. Crop Sci.

[CR178] Gunduz I, Buss GR, Chen P, Tolin SA (2004). Genetic and phenotypic analysis of soybean mosaic virus resistance in PI 88788 soybean. Phytopathology.

[CR179] Guo B, Sleper DA, Arelli PR (2005). Identification of QTLs associated with resistance to soybean cyst nematode races 2, 3 and 5 in soybean PI 90763. Theor Appl Genet.

[CR180] Guo W, Chen JS, Zhang F (2020). Characterization of Pingliang xiaoheidou (ZDD 11047), a soybean variety with resistance to soybean cyst nematode *Heterodera glycines*. Plant Mol Biol.

[CR181] Guo X, Wang D, Gordon SG (2008). Genetic mapping of QTLs underlying partial resistance to *Sclerotinia sclerotiorum* in soybean PI 391589A and PI 391589B. Crop Sci.

[CR182] Gupta GK, Sharma SK, Ramteke R (2012). Biology, epidemiology and management of the pathogenic fungus *Macrophomina phaseolina* (Tassi) Goid with special reference to charcoal rot of soybean (*Glycine max* (L.) Merrill). Phytopathology.

[CR183] Ha BK, Robbins RT, Han F (2007). SSR mapping and confirmation of soybean QTL from PI437654 conditioning resistance to reniform nematode. Crop Sci.

[CR184] Han F, Katt M, Schuh W, et al. (2007) QTL controlling Sclerotinia stem rot resistance in soybean. U.S. Patent 7,250,552

[CR185] Han J, Domier LL, Dorrance AE, Qu F (2013). First report of *Soybean vein necrosis-associated virus* in Ohio soybean fields. Plant Dis.

[CR186] Han Y, Teng W, Yu K (2008). Mapping QTL tolerance to Phytophthora root rot in soybean using microsatellite and RAPD/SCAR derived markers. Euphytica.

[CR187] Hanson PM, Nickell CD, Gray LE, Sebastian SA (1988). Identification of two dominant genes conditioning brown stem rot resistance in soybean. Crop Sci.

[CR188] Harrington TC, McNew DL (2003). Phylogenetic analysis places the Phialophora-like anamorph genus *Cadophora* in the Helotiales. Mycotaxon.

[CR189] Harrington TC, Steimel J, Workneh F, Yang XB (2003). Characterization and distribution of two races of *Phialophora gregata* in the northcentral United States. Phytopathol.

[CR190] Harris DK, Boerma HR, Hussey RS, Finnerty SL (2002). Additional sources of soybean germplasm resistant to two secies of root-knot nematode. Crop Sci.

[CR191] Harris DK, Abdel-Haleem H, Buck JW (2015). Soybean quantitative trait loci conditioning soybean rust-induced canopy damage. Crop Sci.

[CR192] Harrison B, Steinlage TA, Domier LL, D'Arcy CJ (2005). Incidence of soybean dwarf virus and identification of potential vectors in Illinois. Plant Dis.

[CR193] Hartman GL, Datnoff LE, Levy C (1987). Red leaf blotch of soybeans. Plant Dis.

[CR194] Hartman GL, Rupe JC, Sikora EJ (2016). Compendium of soybean diseases.

[CR195] Hartman GL, Murithi HM (2019). Soybean diseases: unique situations in Africa. African J Food Agric Nutr Dev.

[CR196] Hartwig EE, Lehman SG (1951). Inheritance of resistance to the bacterial pustule disease in Soybean. Agron J.

[CR197] Hayes AJ, Ma G, Buss GR, Maroof MS (2000). Molecular marker mapping of *Rsv4*, a gene conferring resistance to all known strains of soybean mosaic virus. Crop Sci.

[CR198] He B, Fajolu OL, Wen RH, Hajimorad MR (2010). Seed transmissibility of alfalfa mosaic virus in soybean. Plant Health Prog.

[CR199] Herald CM, Thames WH (1982) The reniform nematode, *Rotylenchulus reniformis*. In: Riggs RD (ed) Nematology in the southern region of the United States, Southern Cooperative Series Bulletin 276. Arkansas Agricultural Experiment Station, Fayetteville, pp 139–143

[CR200] Herald CM, Robinson AF (1990). Survey of current distribution of *Rotylenchulus reniformis* in the United States. J Nematol (suppl).

[CR201] Herman M, Hussey RS, Boerma HR (1990). Response of resistant soybean plant introductions to *Meloidogyne incognita* in field microplots. J Nematol.

[CR202] Hildebrand AA (1956). Observations on stem canker and pod and stem blight of soybeans in Ontario. Can J Bot.

[CR203] Hill JH, Bailey TB, Benner HI (1987). Soybean mosaic virus: Effects of primary disease incidence on yield and seed quality. Plant Dis.

[CR204] Hnetkovsky N, Chang SJC, Doubler TW (1996). Genetic mapping of loci underlying field resistance to soybean sudden death syndrome (SDS). Crop Sci.

[CR205] Hoffman DD, Hartman GL, Mueller DS (1998). Yield and seed quality of soybean cultivars infected with *Sclerotinia sclerotiorum*. Plant Dis.

[CR206] Holguin CM, Ma X, Mueller JD, Agudelo P (2016). Distribution of *Hoplolaimus* species in soybean fields in South Carolina and North Carolina. Plant Dis.

[CR207] Hopkins JD, Mueller AJ (1984). Effect of bean pod mottle virus on soybean yield. J Econ Entomol.

[CR208] Hoskin A (2011) Genetic mapping of soybean resistance genes to frogeye leaf spot in five Chinese plant introductions and efficiency of early generation selection for low phytate soybean lines. Dissertation, University of Georgia

[CR209] Hossain MM, Akamatsu H, Morishita M (2015). Molecular mapping of Asian soybean rust resistance in soybean landraces PI 594767A, PI 587905 and PI 416764. Plant Pathol.

[CR210] Hossain Z (2019) Study of host resistance of soybean against *Phakopsora pachyrhizi* the causal agent of soybean rust using *Rpp* near isogenic lines. Dissertation, University of Tsukuba

[CR211] Huang J, Guo N, Li Y (2016). Phenotypic evaluation and genetic dissection of resistance to *Phytophthora sojae* in the Chinese soybean mini core collection. BMC Genet.

[CR212] Huynh TV, Dahlbeck D, Staskawicz BJ (1989). Bacterial blight of soybean: Regulation of a pathogen gene determining host cultivar specificity. Science.

[CR213] Huynh TT, Bastien M, Iquira E (2010). Identification of QTLs associated with partial resistance to white mold in soybean using field-based inoculation. Crop Sci.

[CR214] Hwang I, Kim SM (1987). Pathogenic variation in soybeans of by *Xanthomonas campestris* pv. glycines. Phytopathology.

[CR215] Hwang I, Lim SM (1992). Effects of individual and multiple infections with three bacterial pathogens on disease severity and yield of soybeans. Plant Dis.

[CR216] Hwang TY, Moon JK, Yu S (2006). Application of comparative genomics in developing molecular markers tightly linked to the virus resistance gene *Rsv 4* in soybean. Genome.

[CR217] Hyten DL, Hartman GL, Nelson RL (2007). Map location of the *Rpp1* locus that confers resistance to soybean rust in soybean. Crop Sci.

[CR218] Hyten DL, Smith JR, Frederick RD (2009). Bulked segregant analysis using the goldengate assay to locate the *Rpp3* locus that confers resistance to soybean rust in soybean. Crop Sci.

[CR219] Ilut DC, Lipka AE, Jeong N (2016). Identification of haplotypes at the *Rsv4* genomic region in soybean associated with durable resistance to soybean mosaic virus. Theor Appl Genet.

[CR220] Iqbal MJ, Meksem K, Njiti VN (2001). Microsatellite markers identify three additional quantitative trait loci for resistance to soybean sudden-death syndrome (SDS) in Essex× Forrest RILs. Theor Appl Genet.

[CR221] Iquira E, Humira S, François B (2015). Association mapping of QTLs for Sclerotinia stem rot resistance in a collection of soybean plant introductions using a genotyping by sequencing (GBS) approach. BMC Plant Biol.

[CR222] Islam S (2015). Molecular characterization of genetic resistance to soybean cyst nematode in soybean line SS97-6946. J Animal Plant Sci.

[CR223] Jackson EW (2004) Resistance to Phomopsis seed decay and purple seed stain in soybean and virulence differences among *Phomopsis* spp. causing seed decay. Dissertation, University of Arkansas, Fayetteville

[CR224] Jackson EW, Fenn P, Chen P (2005). Inheritance of resistance to Phomopsis seed decay in soybean PI 80837 and MO/PSD-0259 (PI 562694). Crop Sci.

[CR225] Jackson EW, Fenn P, Chen P (2006). Inheritance of resistance to purple seed stain caused by *Cercospora kikuchii* in PI 80837 soybean. Crop Sci.

[CR226] Jackson EW, Feng C, Fenn P, Chen P (2008). Genetic mapping of resistance to purple seed stain in PI 80837 soybean. J Hered.

[CR227] Jacobs JL, Chilvers MI (2013). First report of *Soybean vein necrosis virus* on soybeans in Michigan. Plant Dis.

[CR228] Jang IH, Kang IJ, Kim JM (2020). Genetic mapping of a resistance locus to *Phytophthora sojae* in the Korean soybean cultivar Daewon. Plant Pathol.

[CR229] Jang IH, Lee S (2020). A review and perspective on soybean (*Glycine max* L.) breeding for the resistance to *Phytophthora sojae* in Korea. Plant Breed Biotechnol.

[CR230] Jarvie JA (2009). A review of soybean rust from a South African perspective. S Afr J Sci.

[CR231] Jeong SC, Kristipati S, Hayes AJ (2002). Genetic and sequence analysis of markers tightly linked to the soybean mosaic virus resistance gene, *Rsv3*. Crop Sci.

[CR232] Jiang B, Cheng Y, Cai Z (2020). Fine mapping of a Phytophthora-resistance locus *RpsGZ* in soybean using genotyping-by-sequencing. BMC Genom.

[CR233] Jiao Y, Vuong TD, Liu Y (2015). Identification of quantitative trait loci underlying resistance to southern root-knot and reniform nematodes in soybean accession PI 567516C. Mol Breed.

[CR234] Jing Y, Teng W, Qiu L (2021). Genetic dissection of soybean partial resistance to sclerotinia stem rot through genome wide association study and high throughout single nucleotide polymorphisms. Genomics.

[CR235] Jones JP (1968). Survival of *Cercospora kikuchii* on soybean stems in the field. Plant Dis Rep.

[CR236] Jones JT, Haegeman A, Danchin EGJ (2013). Top 10 plant-parasitic nematodes in molecular plant pathology. Mol Plant Pathol.

[CR237] Jones RA (2021). Global plant virus disease pandemics and epidemics. Plants.

[CR238] Kadam S, Vuong TD, Qiu D (2016). Genomic-assisted phylogenetic analysis and marker development for next generation soybean cyst nematode resistance breeding. Plant Sci.

[CR239] Kandel R, Chen CY, Grau CR (2018). Soybean resistance to white mold: evaluation of soybean germplasm under different conditions and validation of QTL. Front Plant Sci.

[CR240] Kandoth PK, Liu S, Prenger E (2017). Systematic mutagenesis of serine hydroxymethyltransferase reveals an essential role in nematode resistance. Plant Physiol.

[CR241] Karhoff S, Lee S, Mian R (2019). Phenotypic characterization of a major quantitative disease resistance locus for partial resistance to *Phytophthora sojae*. Crop Sci.

[CR242] Karl TR, Melillo JM, Peterson TC, Hassol SJ (2009). Global climate change impacts in the United States.

[CR243] Karthikeyan A, Li K, Jiang H (2017). Inheritance, fine-mapping, and candidate gene analyses of resistance to soybean mosaic virus strain *SC5* in soybean. Mol Genet Genom.

[CR244] Kassem MA, Shultz J, Meksem K (2006). An updated ‘Essex’ by ‘Forrest’ linkage map and first composite interval map of QTL underlying six soybean traits. Theor Appl Genet.

[CR245] Kasuga T, Salimath SS, Shi J (1997). High resolution genetic and physical mapping of molecular markers linked to the Phytophthora resistance gene *Rps1-k* in soybean. Mol Plant Microbe Interact.

[CR246] Kaufmann MJ, Gerdemann JW (1958). Root and stem rot of soybean caused by *Phytophthora sojae* n.sp. Phytopathology.

[CR247] Kazi S, Shultz J, Afzal J (2008). Separate loci underlie resistance to root infection and leaf scorch during soybean sudden death syndrome. Theor Appl Genet.

[CR248] Keeling BL (1985). Soybean cultivar reactions to soybean stem canker caused by *Diaporthe phaseolorum* var. caulivora and pathogenic variation among isolates. Plant Dis.

[CR249] Keeling BL (1988). Influence of temperature on growth and pathogenicity of geographic isolates of *Diaporthe phaseolorum var. caulivora*. Plant Dis.

[CR250] Keen NT, Buzzell RI (1991). New disease resistance genes in soybean against *Pseudomonas syringae pv glycinea*: evidence that one of them interacts with a bacterial elicitor. Theor Appl Genet.

[CR251] Kennedy BW, Tachibana H, Caldwell BE (1973). Bacterial diseases. Soybeans: improvement, production, and uses.

[CR708] Khalili E, Javed MA, Huyop F (2016). Evaluation of Trichoderma isolates as potential biological control agent against soybean charcoal rot disease caused by Macrophomina phaseolina. Biotechnol. Equip..

[CR252] Khalili E, Kouchaki S, Ramazi S, Ghanati F (2020). Machine learning techniques for soybean charcoal rot disease prediction. Front Plant Sci.

[CR253] Khan MJ (2011). Genetic analysis of race-specificity of *Pseudomonas syringae* pv. *glycinea*. Pak J Bot.

[CR254] Kiihl RAS, Hartwig EE (1979). Inheritance of reaction to soybean mosaic virus in soybeans. Crop Sci.

[CR255] Kilen TC, Hartwig EE, Keeling BL (1974). Inheritance of a second major gene for resistance to Phytophthora rot in soybeans. Crop Sci.

[CR700] Kilen TC, Hartwig EE (1987). Identification of Single Genes Controlling Resistance to Stem Canker in Soybean. Crop Sci.

[CR256] Kim DH, Kim KH, Van K (2010). Fine mapping of a resistance gene to bacterial leaf pustule in soybean. Theor Appl Genet.

[CR257] Kim H, Newell AD, Cota-Sieckmeyer RG (2013). Mating-type distribution and genetic diversity of *Cercospora sojina* populations on soybean from Arkansas: evidence for potential sexual reproduction. Phytopathology.

[CR258] Kim H, Kim S-T, Ryu J (2017). CRISPR/Cpf1-mediated DNA-free plant genome editing. Nat Commun.

[CR259] Kim HS, Diers BW (2000). Inheritance of partial resistance to Sclerotinia stem rot in soybean. Crop Sci.

[CR260] Kim KH, Park JH, Kim MY (2011). Genetic mapping of novel symptom in response to soybean bacterial leaf pustule in PI 96188. J Crop Sci Biotechnol.

[CR261] Kim KS, Kim MY, Lee SH (2004). Development of molecular markers for *Xanthomonas axonopodis* resistance in soybean. Korean J Crop Sci.

[CR262] Kim KS, Unfried JR, Hyten DL (2012). Molecular mapping of soybean rust resistance in soybean accession PI 561356 and SNP haplotype analysis of the *Rpp1* region in diverse germplasm. Theor Appl Genet.

[CR263] Kim KS, Vuong TD, Qiu D (2016). Advancements in breeding, genetics, and genomics for resistance to three nematode species in soybean. Theor Appl Genet.

[CR264] King ZR, Harris DK, Pedley KF (2016). A novel *Phakopsora pachyrhizi* resistance allele (*Rpp*) contributed by PI 567068A. Theor Appl Genet.

[CR265] Kinloch RA, Barker KR, Pederson GA, Windham GL, Bartels JM (1998). Soybean. Plant and nematodes interactions.

[CR266] Kinloch RA, Hiebsch CK, Peacock HA (1984). Comparative root-knot galling and yield responses of soybean cultivars to *Meloidogyne incognita*. Plant Dis.

[CR267] Kirkpatrick MT, Rothrock CS, Rupe JC, Gbur EE (2006). The effect of *Pythium ultimum* and soil flooding on two soybean cultivars. Plant Dis.

[CR268] Kleczewski N (2016) Research updates on soybean vein necrosis virus. Farms.com. https://m.farms.com/news/research-updates-on-soybean-vein-necrosis-virus-102825.aspx. Accessed 7 Sept 2018

[CR269] Klepadlo M, Chen P, Wu C (2016). Genetic analysis of resistance to soybean mosaic virus in PI 438307 soybean accession. Crop Sci.

[CR270] Klepadlo M, Chen P, Shi A (2017). Two tightly linked genes for soybean mosaic virus resistance in soybean. Crop Sci.

[CR271] Klepadlo M, Balk CS, Vuong TD (2019). Molecular characterization of genomic regions for resistance to *Pythium ultimum var. ultimum* in the soybean cultivar Magellan. Theor Appl Genet.

[CR272] Klos KL, Paz MM, Marek LF, Cregan PB, Shoemaker RC (2000). Molecular markers useful for detecting resistance to brown stem rot in soybean. Crop Sci.

[CR273] Koenning SR, Overstreet C, Noling JW (1999). Survey of crop losses in response to phytoparasitic nematodes in the United States for 1994. J Nematol (suppl).

[CR274] Koenning SR (2010) Southern United States soybean disease loss estimate for 2009. Proceedings of the Southern Soybean Disease Workers, the 37th Annual Meeting

[CR275] Koenning SR, Wrather JA (2010). Suppression of soybean yield potential in the continental United States by plant diseases from 2006 to 2009. Plant Health Prog.

[CR276] Kofsky J, Zhang H, Song BH (2021). Novel resistance strategies to soybean cyst nematode (SCN) in wild soybean. Sci Rep.

[CR277] Kopisch-Obuch FJ, NKoval NC, Mueller EM, (2008). Inheritance of resistance to alfalfa mosaic virus in soybean PI 153282. Crop Sci.

[CR278] Kulik MM, Sinclair JB, Hartman GL, Sinclair JB, Rupe JC (1999). Phomopsis seed decay. Compendium of Soybean diseases.

[CR279] Kulik MM, Sinclair JB, Hartman GL, Hartman GL, Sinclair JB, Rupe JC (1999). Pod and stem blight. Compendium of Soybean diseases.

[CR280] Kumudini S, Godoy CV, Board JE (2008). Mechanisms involved in soybean rust-induced yield reduction. Crop Sci.

[CR281] Kurle JE, Grau CR, Oplinger ES, Mengistu A (2001). Tillage, crop sequence, and cultivar effects on Sclerotinia stem rot incidence and yield in soybean. Agron J.

[CR282] Kyrychenko AM, Kraeva GV, Kovalenko OG (2012). Biological characteristic and identification of soybean viruses isolated from different Ukraine regions. Miкpoбioлoгiчний Жypнaл.

[CR283] Lakhssassi N, Liu S, Bekal S (2017). Characterization of the soluble NSF attachment protein gene family identifies two members involved in additive resistance to a plant pathogen. Sci Rep.

[CR284] Langenbach C, Campe R, Beyer SF (2016). Fighting Asian soybean rust. Front Plant Sci.

[CR285] Laviolette FA, Athow KL, Probst AH (1970). Effect of bacterial pustule and frogeye leaf spot on yield of Clark soybean. Crop Sci.

[CR286] Lebreton A, Labbé C, De Ronne M (2018). Development of a simple hydroponic assay to study vertical and horizontal resistance of soybean and pathotypes of *Phytophthora sojae*. Plant Dis.

[CR287] Lee JD, Kim HK, Robbins RT (2015). Reaction of soybean cyst nematode resistant plant introductions to root-knot and reniform nematodes. Plant Breed Biotech.

[CR288] Lee S, Rouf Mian MR, McHale LK (2013). Novel quantitative trait loci for partial resistance to *Phytophthora sojae* in soybean PI 398841. Theor Appl Genet.

[CR289] Lee S, Rouf Mian MA, McHale LK (2013). Identification of quantitative trait loci conditioning partial resistance to *Phytophthora sojae* in soybean PI 407861A. Crop Sci.

[CR290] Lee S, Mian MR, Sneller CH (2014). Joint linkage QTL analyses for partial resistance to *Phytophthora sojae* in soybean using six nested inbred populations with heterogeneous conditions. Theor Appl Genet.

[CR291] Lee YC, Lightfoot DA, Anderson J (2016). QTL underlying reniform nematode resistance in soybean cultivar Hartwig. Atlas J Biol.

[CR292] Lee YC (2021) Evaluation of soybean diseases and pests using two advanced breeding population. Dissertation, Southern Illinois University Carbondale

[CR710] Levy C, Mahuku GS, Tattersfield JR (1990). Method of assessment of red leaf blotch on soybeans used to evaluate cultivar susceptibility and chemical control. Crop Prot.

[CR293] Lewers KS, Crane EH, Bronson CR (1999). Detection of linked QTL for soybean brown stem rot resistance in ‘BSR 101’ as expressed in a growth chamber environment. Mol Breed.

[CR294] Lewis SA, Fassuliotis G, Riggs RD (1982). Lance nematodes, *Hoplolaimus spp.*, in the Southern United States. Nematology in the Southern region of the United States.

[CR295] Lewis SA, Sinclair IB, Blackmon PA (1989). Lance nematodes. Compendium of Soybean diseases.

[CR296] Li D, Chen P, Alloatti J (2010). Identification of new alleles for resistance to soybean mosaic virus in soybean. Crop Sci.

[CR297] Li D, Sun M, Han Y (2010). Identification of QTL underlying soluble pigment content in soybean stems related to resistance to soybean white mold (*Sclerotinia sclerotiorum*). Euphytica.

[CR298] Li K, Yang QH, Zhi HJ, Gai JY (2010). Identification and distribution of soybean mosaic virus strains in southern China. Plant Dis.

[CR299] Li K, Ren R, Adhimoolam K (2015). Genetic analysis and identification of two soybean mosaic virus resistance genes in soybean [*Glycine max* (L.) Merr.]. Plant Breed.

[CR300] Li K, Ren R, Wang T (2017). Genetic analysis and mapping of soybean mosaic virus resistance genes to *SC18* in soybean. Soybean Sci.

[CR301] Li L, Lin F, Wang W (2016). Fine mapping and candidate gene analysis of two loci conferring resistance to *Phytophthora sojae* in soybean. Theor Appl Genet.

[CR302] Li L, Guo N, Niu J (2016). Loci and candidate gene identification for resistance to *Phytophthora sojae* via association analysis in soybean [*Glycine max* (L.) Merr.]. Mol Gen Genom.

[CR303] Li S, Bradley CA, Hartman GL, Pedersen WL (2001). First report of *Phomopsis longicolla* from velvetleaf causing stem lesions on inoculated soybean and velvetleaf plants. Plant Dis.

[CR304] Li S, Rupe J, Chen P, Wrather A (2010a) Reaction of maturity group IV soybean plant introductions to Phomopsis seed decay in Arkansas, Mississippi, and Missouri, 2009. Plant Dis Manag Rep 4

[CR305] Li X, Han Y, Teng W (2010). Pyramided QTL underlying tolerance to Phytophthora root rot in mega-environments from soybean cultivars ‘Conrad’ and ‘Hefeng 25’. Theor Appl Genet.

[CR306] Li M, Liu N, Ma Q (2020). Fine mapping and analyses of the *RSC15ZH* resistance candidate gene for the soybean mosaic virus. Euphytica.

[CR307] Li S (2018). Development of a seedling inoculation technique for rapid evaluation of soybean for resistance to *Phomopsis longicolla* under controlled conditions. Plant Methods.

[CR308] Li S, Darwish O, Alkharouf N (2015). Draft genome sequence of *Phomopsis longicolla* isolate MSPL 10–6. Genom Data.

[CR309] Li S, Darwish O, Alkharouf NW, Musungu B, Matthews BF (2017). Analysis of the genome sequence of *Phomopsis longicolla*: A fungal pathogen causing Phomopsis seed decay in soybean. BMC Genom.

[CR310] Li S, Musungu B, Lightfoot D, Ji P (2018). The interactomic analysis reveals pathogenic protein networks in *Phomopsis longicolla* underlying seed decay of soybean. Front Genet.

[CR311] Li S, Sciumbato G, Rupe J (2017). Evaluation of commercial soybean cultivars for reaction to Phomopsis seed decay. Plant Dis.

[CR312] Li S, Sciumbato G, Boykin D (2019). Evaluation of soybean genotypes for reaction to natural field infection by *Cercospora* species causing purple seed stain. PLoS ONE.

[CR313] Li S, Smith J, Nelson R (2011). Resistance to Phomopsis seed decay identified in maturity group V soybean plant introductions. Crop Sci.

[CR314] Li S, Smith JR, Ray JD, Frederick RD (2012). Identification of a new soybean rust resistance gene in PI 567102B. Theor Appl Genet.

[CR315] Li YH, Shi XH, Li H (2016). Dissecting the genetic basis of resistance to soybean cyst nematode combining linkage and association mapping. Plant Genome.

[CR316] Li Y, Sun S, Zhong C (2017). Genetic mapping and development of co-segregating markers of *RpsQ*, which provides resistance to *Phytophthora sojae* in soybean. Theor Appl Genet.

[CR317] Li Z, Jakkula L, Hussey RS (2001). SSR mapping and confirmation of the QTL from PI96354 conditioning soybean resistance to southern root-knot nematode. Theor Appl Genet.

[CR318] Lightfoot DA (2015). Two decades of molecular marker-assisted breeding for resistance to soybean sudden death syndrome. Crop Sci.

[CR319] Lim SM, Bernard RL, Nickell CD, Gray LE (1984). New physiological race of *Peronospora manshurica* virulent to the gene *Rpm* in soybeans. Plant Dis.

[CR320] Lim SM (1989). Inheritance of resistance of *Peronospora manshurica* races 2 and 33 in soybean. Phytopathology.

[CR321] Lima FS, Correa VR, Nogueira SR, Santos PR, Kasai M (2017). Nematodes affecting soybean and sustainable practices for their management. Soybean, The basis of yield, biomass and productivity.

[CR322] Lin F, Zhao M, Ping J (2013). Molecular mapping of two genes conferring resistance to *Phytophthora sojae* in a soybean landrace PI 567139B. Theor Appl Genet.

[CR323] Lin F, Zhao M, Baumann DD (2014). Molecular response to the pathogen *Phytophthora sojae* among ten soybean near isogenic lines revealed by comparative transcriptomics. BMC Genom.

[CR324] Lin F, Wani SH, Collins PJ (2018). Mapping quantitative trait loci for tolerance to *Pythium irregulare* in soybean (*Glycine max* L.). G3.

[CR325] Lin F, Wani SH, Collins PJ (2020). QTL mapping and GWAS for identification of loci conferring partial resistance to Pythium sylvaticum in soybean (*Glycine max* (L.) Merr). Mol Breed.

[CR326] Lin F, Li W, McCoy AG (2021). Molecular mapping of quantitative disease resistance loci for soybean partial resistance to *Phytophthora sansomeana*. Theor Appl Genet.

[CR327] Lin Z, Wang D, Zhang H (2016). Fine mapping of the *RSC8* locus and expression analysis of candidate SMV resistance genes in soybean. Plant Breed.

[CR328] Linford MB, Oliveira JM (1940). *Rotylenchulus reniformis*, nov. gen., n., sp., a nematode parasite of roots. Proc Helminthol Soc Wash.

[CR329] Liu S, Ge F, Huang W, Lightfoot DA, Peng D (2019). Effective identification of soybean candidate genes involved in resistance to soybean cyst nematode via direct whole genome re-sequencing of two segregating mutants. Theor Appl Genet.

[CR330] Liu S, Kandoth PK, Warren SD (2012). A soybean cyst nematode resistance gene points to a new mechanism of plant resistance to pathogens. Nature.

[CR331] Liu S, Kandoth PK, Lakhssassi N (2017). The soybean GmSNAP18 gene underlies two types of resistance to soybean cyst nematode. Nat Commun.

[CR332] Liu S, Yu H, Sui Y (2021). Classification of soybean frogeye leaf spot disease using leaf hyperspectral reflectance. PLoS ONE.

[CR333] Loesch-Fries LS, Bamford DH, Zuckerman M (2021). Alfalfa mosaic virus (*Bromoviridae*). Encyclopedia of virology.

[CR334] Lohnes DG, Schmitthenner AF (1997). Position of the *Phytophthora* resistance gene *Rps7* on the soybean molecular map. Crop Sci.

[CR335] Luckew A, Cianzio SR, Leandro LF (2012). Screening method for distinguishing soybean resistance to *Fusarium virguliforme* in resistant × resistant crosses. Crop Sci.

[CR336] Luckew AS, Leandro LF, Bhattacharyya MK (2013). Usefulness of 10 genomic regions in soybean associated with sudden death syndrome resistance. Theor Appl Genet.

[CR337] Luckew AS, Swaminathan S, Leandro LF (2017). ‘MN1606SP’ by ‘Spencer’ filial soybean population reveals novel quantitative trait loci and interactions among loci conditioning SDS resistance. Theor Appl Genet.

[CR338] Ludke WH, Schuster I, Silva FL (2019). SNP markers associated with soybean partial resistance to *Phytophthora sojae*. Crop Breed Appl Biotechnol.

[CR339] Lurá MC, Latorre MG, Vaccari RMC (2011). Genetic diversity of *Cercospora kikuchii* isolates from soybean cultured in Argentina as revealed by molecular markers and cercosporin production. Mycopathologia.

[CR340] Luzzi BM, Boerma HR, Hussey RS (1987). Resistance to three species of root-knot nematode in soybean. Crop Sci.

[CR341] Luzzi BM, Boerma HR, Hussey RS (1994). A gene for resistance to the southern root-knot nematode in soybean. J Hered.

[CR342] Luzzi BM, Boerma HR, Hussey RS (1994). Inheritance of resistance to the southern root-knot nematode in soybean. Crop Sci.

[CR343] Ma GZ (1994). Review and forecast of study on frogeye leaf spot. Soybean J.

[CR344] Ma G, Chen P, Buss GR, Tolin SA (2002). Complementary action of two independent dominant genes in Columbia soybean for resistance to soybean mosaic virus. J Hered.

[CR345] Ma G, Chen P, Buss GR, Tolin SA (2003). Genetic study of a lethal necrosis to soybean mosaic virus in PI 507389 soybean. J Hered.

[CR346] Ma Y, Wang DG, Li HC (2011). Fine mapping of the *R-SC14Q* locus for resistance to soybean mosaic virus in soybean. Euphytica.

[CR347] Malapi-Nelson M, Wen RH, Ownley BH, Hajimorad MR (2009). Co-infection of soybean with soybean mosaic virus and alfalfa mosaic virus results in disease synergism and alteration in accumulation level of both viruses. Plant Dis.

[CR348] Mathew FM, Gulya TJ, Jordahl JG, Markell SG (2018). First report of stem disease of soybean (*Glycine max*) caused by *Diaporthe gulyae* in North Dakota. Plant Dis.

[CR349] Matsumoto T, Tomoyasu R (1925). Studies on the purple speck of soybean seed. Ann Phytopathol Soc Jpn.

[CR350] Matsuo E, Ferreira PA, Sediyama T, Borem A, Sediyama T, Ludke W (2017). Resistance to diseases. Soybean breeding.

[CR351] Matsuoka JI, Takahashi M, Yamada T (2021). Identification of three closely linked loci conferring broad-spectrum *Phytophthora sojae* resistance in soybean variety Tosan-231. Theor Appl Genet.

[CR352] Matthiesen RL, Schmidt C, Garnica VC (2021). Comparison of *Phytophthora sojae* populations in Iowa and Nebraska to identify effective *Rps* genes for Phytophthora stem and root rot management. Plant Heal Prog.

[CR353] McAllister KR, Lee YC, Kantartzi SK (2021). QTL mapping for resistance to *Cercospora sojina* in ‘Essex’ × ‘Forrest’ soybean (*Glycine max* L.) lines. J Plant Breed Crop Sci.

[CR354] McCabe CE, Graham MA (2020). New tools for characterizing early brown stem rot disease resistance signaling in soybean. Plant Genome.

[CR355] McCoy AG, Jacobs JL, Chilvers M (2018). *Phytophthora sansomeana* host characterization in Michigan field crops. Phytopathology.

[CR356] McCoy AG, Noel ZA, Jacobs JL (2021). *Phytophthora sojae* pathotype distribution and fungicide sensitivity in Michigan. Plant Dis.

[CR357] McGee DC, Biddle JA (1987). Seedborne *Diaporthe phaseolorum* var. caulivora in Iowa and its relationship to soybean stem canker in the Southern United States. Plant Dis.

[CR358] McCaghey M, Willbur J, Ranjan A (2017). Development and evaluation of *Glycine max* germplasm lines with quantitative resistance to *Sclerotinia sclerotiorum*. Front Plant Sci.

[CR359] Meksem K, Doubler TW, Chancharoenchai K (1999). Clustering among loci underlying soybean resistance to *Fusarium solani*, SDS and SCN in near-isogenic lines. Theor Appl Genet.

[CR360] Meksem K, Pantazopoulos P, Njiti VN (2001). ’Forrest’resistance to the soybean cyst nematode is bigenic: saturation mapping of the Rhg1and Rhg4 loci. Theor Appl Genet.

[CR361] Mengistu A, Kurtzweil NC, Grau CR (2002). First report of Frogeye Leaf Spot (*Cercospora sojina*) in Wisconsin. Plant Dis.

[CR362] Mengistu A, Ray JD, Smith JR, Paris RL (2007). Charcoal rot disease assessment of soybean genotypes using a colony-forming unit index. Crop Sci.

[CR363] Mengistu A, Smith JR, Bellaloui N (2010). Irrigation and time of harvest effects on evaluation of selected soybean accessions against *Phomopsis longicolla*. Crop Sci.

[CR364] Mengistu A, Wrather A, Little CR (2011). Evaluation of soybean genotypes for resistance to charcoal rot. Plant Health Prog.

[CR365] Mengistu A, Bond J, Mian R (2012). Resistance to frogeye leaf spot in selected soybean accessions in MG I through MG VI. Plant Health Prog.

[CR366] Mengistu A, Arelli PR, Bond J (2013). Identification of soybean accessions resistant to *Macrophomina phaseolina* by field screening and laboratory validation. Plant Health Prog.

[CR367] Mengistu A, Ray JD, Smith JR (2014). Maturity effects on colony-forming units of *Macrophomina phaseolina* infection as measured using near-isogenic lines of soybeans. J Crop Improv.

[CR368] Mengistu A, Rupe JC, Wrather JA, Hartman GL, Rupe JC, Sikora EJ, Domier LL, Steffey KL, Davis JA (2016). Charcoal rot. Compendium of Soybean diseases and pests.

[CR369] Mengistu A, Ray JD, Smith JR (2018). Effect of charcoal rot on selected putative drought tolerant soybean genotypes and yield. Crop Prot.

[CR370] Mengistu A, Arelli PR, Bellaloui N (2021). Resistance to charcoal rot identified within soybean cyst nematode resistant accessions. Plant Health Progress.

[CR371] Mian MAR, Boerma HR, Phillips DV (1998). Performance of frogeye leaf spot resistant and susceptible near isolines of soybean. Plant Dis.

[CR372] Mian MA, Wang T, Phillips DV (1999). Molecular mapping of the *Rcs3* gene for resistance to frogeye leaf spot in soybean. Crop Sci.

[CR702] Mian MA, Missaoui AM, Walker DR, Phillips DV (2008). Boerma HR (2008) Frogeye leaf spot of soybean: A review and proposed race designations for isolates of Cercospora sojina Hara. Crop Sci.

[CR373] Mikhaylov A, Moiseev N, Aleshin K, Burkhardt T (2020). Global climate change and greenhouse effect. Entrep Sustain Issues.

[CR374] Million CR, Wijeratne S, Cassone BJ (2019). Hybrid genome assembly of a major quantitative disease resistance locus in soybean toward *fusarium graminearum*. Plant Genome.

[CR375] Mimee B, Peng H, Popovic V (2015). First report of soybean cyst nematode (*Heterodera glycines* Ichinohe) on soybean in the Province of Quebec, Canada. Nematol.

[CR376] Minor HC, Brown EA, Doupnik B (1993). Registration of Phomopsis seed decay resistant soybean germplasm MO/PSD-0259. Crop Sci.

[CR377] Mishchenko LT, Dunich AA, Shevchenko TP (2017). Detection of Soybean mosaic virus in some left-bank forest-steppe regions of Ukraine. Miкpoбioлoгiчний Жypнaл.

[CR378] Missaoui AM, Ha BK, Phillips DV (2007). Single nucleotide polymorphism detection of the *Rcs3* gene for resistance to frogeye leaf spot in soybean. Crop Sci.

[CR379] Missaoui AM, Phillips DV, Boerma HR (2007). DNA marker analysis of 'Davis' soybean and its descendants for the *Rcs3* gene conferring resistance to *Cercospora sojina*. Crop Sci.

[CR380] Moellers TC, Singh A, Zhang J (2017). Main and epistatic loci studies in soybean for *Sclerotinia sclerotiorum* resistance reveal multiple modes of resistance in multi-environments. Sci Rep.

[CR381] Monteros MJ, Missaoui AM, Phillips DV (2007). Mapping and confirmation of the “Hyuuga” red-brown lesion resistance gene for Asian soybean rust. Crop Sci.

[CR382] Moon JK, Jeong SC, Van K (2009). Marker-assisted identification of resistance genes to soybean mosaic virus in soybean lines. Euphytica.

[CR383] Morse WJ, Cartter JL, Williams LF (1949). Soybeans: culture and varieties.

[CR384] Mueller EH, Athow KL, Laviolette FA (1978). Inheritance of resistance to four physiologic races of *Phytophthora megasperma* var sojae. Phytopathology.

[CR385] Mueller JD, Sanders GB (1987). Control of *Hoplolaimus columbus* on late-planted soybean with aldicarb. J Nematol (suppl).

[CR386] Mueller EE, Grau CR (2007). Seasonal progression, symptom development, and yield effects of alfalfa mosaic virus epidemics on soybean in Wisconsin. Plant Dis.

[CR387] Mueller DS, Wise KA, Sisson AJ, et al (2016) A Farmer’s Guide to Soybean Diseases. St. Paul, Minnesota

[CR388] Mukherjee D, Lambert JW, Cooper RL, Kennedy BW (1966). Inheritance of resistance to bacterial blight (*Pseudomonas glycinea Coerper*) in soybeans (*Glycine* max L.). Crop Sci.

[CR389] Mundt CC (2014). Durable resistance: a key to sustainable management of pathogens and pests. Infect Genet Evol.

[CR390] Murithi HM, Beed F, Tukamuhabwa P (2016). Soybean production in eastern and southern Africa and threat of yield loss due to soybean rust caused by *Phakopsora pachyrhizi*. Plant Pathol.

[CR391] Muyolo NG, Lipps PE, Schmitthenner AF (1993). Reactions of dry bean, lima bean, and soybean cultivars to Rhizoctonia root and hypocotyl rot and web blight. Plant Dis.

[CR392] Myhre DL, Pitre HN, Haridasan M, Hesketh JD (1973). Effect of bean pod mottle virus on yield components and morphology of soybeans in relation to soil water regimes: a preliminary study. Plant Dis Rep.

[CR393] Nakajima T, Mitsueda T, Charchar MJD (1996). First occurrence of sudden death syndrome of soybean in Brazil. Japan Agric Res Q.

[CR394] Narvel JM, Jakkula LR, Phillips DV (2001). Molecular mapping of *Rxp* conditioning reaction to bacterial pustule in soybean. J Hered.

[CR395] Nataraj V, Wani S (2019). Charcoal rot resistance in Soybean: current understanding and future perspectives. Disease resistance in crop plants.

[CR396] Nelson RL, Nickell CD, Orf JH (1989). Evaluating soybean germplasm for brown stem rot resistance. Plant Dis.

[CR397] Nelson R, Wiesner-Hanks T, Wisser R, Balint-Kurti P (2018). Navigating complexity to breed disease-resistant crops. Nat Rev Genet.

[CR398] Nguyen V, Vuong T, VanToai T (2012). Mapping of quantitative trait loci associated with resistance to *Phytophthora sojae* and flooding tolerance in soybean. Crop Sci.

[CR399] Niblack TL, Lambert KN, Tylka GL (2006). A model plant pathogen from the kingdom animalia: *Heterodera glycines*, the soybean cyst nematode. Annu Rev Phytopathol.

[CR400] Niu J, Guo N, Sun J (2017). Fine mapping of a resistance gene *RpsHN* that controls *Phytophthora sojae* using recombinant inbred lines and secondary populations. Front Plant Sci.

[CR401] Niu J, Guo N, Zhang Z (2018). Genome-wide SNP-based association mapping of resistance to Phytophthora sojae in soybean (*Glycine max* (L.) Merr.). Euphytica.

[CR402] Njiti VN, Doubler TW, Suttner RJ (1998). Resistance to soybean sudden death syndrome and root colonization by *Fusarium solani* f. sp. *glycine* in near-isogenic lines. Crop Sci.

[CR403] Njiti VN, Meksem K, Iqbal MJ (2002). Common loci underlie field resistance to soybean sudden death syndrome in Forrest, Pyramid, Essex, and Douglas. Theor Appl Genet.

[CR404] Njiti VN, Lightfoot DA (2006). Genetic analysis infers *Dt* loci underlie resistance to *Fusarium solani* f. sp. *glycines* in indeterminate soybeans. Can J Plant Sci.

[CR405] Noe JP (1993). Damage functions and population changes of *Hoplolaimus columbus* on cotton and soybean. J Nematol.

[CR406] Ohnishi S, Miyake N, Takeuchi T (2012). Fine mapping of foxglove aphid (*Aulacorthum solani*) resistance gene *Raso1* in soybean and its effect on tolerance to soybean dwarf virus transmitted by foxglove aphid. Breed Sci.

[CR709] Orojnia S, Habibi D, Shahbazi S (2021). Investigation of biological control of trichoderma formulations and its mutant type related to chemical treatments in the control of soybean charcoal rots. Rom Agric Res.

[CR407] Orth CE, Schuh W (1994). Resistance of 17 soybean cultivars to foliar, latent, and seed infection by *Cercospora kikuchii*. Plant Dis.

[CR408] Ortiz-Bobea A, Ault TR, Carrillo CM (2021). Anthropogenic climate change has slowed global agricultural productivity growth. Nat Clim Chang.

[CR409] Pace PF, Weaver DB, Ploper LD (1993). Additional genes for resistance to frogeye leaf spot race 5 in soybean. Crop Sci.

[CR410] Palmer RG, Lim SM, Hedges BR (1992). Testing for linkage between the *Rxp* locus and nine isozyme loci in soybean. Crop Sci.

[CR411] Palmer RG, Pfeifer TW, Buss GR, Kilen TC, Boerma HR, Specht JE (2004). Qualitative genetics. Soybeans: improvement, production, and uses.

[CR412] Park EW (1991). Studies on effective control for cyst nematodes and Phomopsis seed decay of soybean. Korea Soybean Dig.

[CR413] de Passianotto AL, L, Sonah H, Dias WP, (2017). Genome-wide association study for resistance to the southern root-knot nematode (*Meloidogyne incognita*) in soybean. Mol Breed.

[CR414] Pathan MS, Clark KM, Wrather JA (2009). Registration of soybean germplasm SS93-6012 and SS93-6181 resistant to Phomopsis seed decay. J Plant Regist.

[CR415] Patil GB, Lakhssassi N, Wan J (2019). Whole-genome re-sequencing reveals the impact of the interaction of copy number variants of the *rhg1* and *Rhg4* genes on broad-based resistance to soybean cyst nematode. Plant Biotechnol J.

[CR416] Pedigo LP, Zeiss MR (1996). Effect of soybean planting date on bean leaf beetle (Coleoptera: Chrysomeli-dae) abundance and pod injury. J Econ Entomol.

[CR417] Peltier AJ, Bradley CA, Chilvers MI (2012). Biology, yield loss and control of Sclerotinia stem rot of soybean. J Integr Pest Manag.

[CR418] Peng DL, Peng H, Wu DQ (2016). First report of soybean cyst nematode (*Heterodera glycines*) on soybean from Gansu and Ningxia China. Plant Dis.

[CR419] Petrović K, Skaltsas D, Castlebury LA (2021). Diaporthe seed decay of soybean [*Glycine max* (L.) Merr.] is endemic in the United States, but new fungi are involved. Plant Dis.

[CR420] Pham AT, McNally K, Abdel-Haleem H (2013). Fine mapping and identification of candidate genes controlling the resistance to southern root-knot nematode in PI 96354. Theor Appl Genet.

[CR421] Pham A, Harris D, Buck J (2015). Fine mapping and characterization of candidate genes that control resistance to Cercospora sojina K. Hara in two soybean germplasm accessions. PLoS ONE.

[CR422] Phillips D, Boerma H (1982). Two genes for resistance to race 5 of *Cercospora sojina* in soybeans. Phytopathology.

[CR423] Phillips D, Hartman GL, Sinclair JB, Rupe JC (1999). Frogeye leaf spot. Compendium of Soybean diseases.

[CR424] Pierozzi PHB, Ribeiro AS, Moreira JUV (2008). New soybean (*Glycine max* Fabales, Fabaceae) sources of qualitative genetic resistance to Asian soybean rust caused by *Phakopsora pachyrhizi* (Uredinales, Phakopsoraceae). Genet Mol Biol.

[CR426] Pioli RN, Morandi EN, Martínez MC (2003). Morphologic, molecular, and pathogenic characterization of *Diaphorte phaseolorum* variabillity in the core soybean-producing area of Argentina. Phytopathology.

[CR704] Pioli RN, Mozzoni L, Morandi EN (2004). First report of pathogenic association between Fusarium graminearum and soybean. Plant Disease.

[CR425] Ping J, Fitzgerald JC, Zhang C (2016). Identification and molecular mapping of *Rps11*, a novel gene conferring resistance to *Phytophthora sojae* in soybean. Theor Appl Genet.

[CR427] Ploper LD, Athow KL, Laviolette FA (1985). A new allele at the *Rps3* locus for resistance to *Phytophthora megasperma* f. sp. glycinea in soybean. Phytopathology.

[CR428] Ploper LD, Abney TS, Roy KW (1992). Influence of soybean genotype on rate of seed maturation and its impact on seedborne fungi. Plant Dis.

[CR429] Ploper LD (1993). Síndrome de la muerte súbita: nueva enfermedad de la soja en el noroeste argentino. Av Agroindustrial Ano.

[CR430] Ploper LD (2003) Importancia de las enfermedades de la soja en el Mercosur. In: Actas Simposio Internacional sobre Soja, XI Congreso de AAPRESID, Rosario, Argentina, August 26–29, 2003, pp 163–174

[CR431] Polzin KM, Lohnes DG, Nickell CD, Shoemaker RC (1994). Integration of *Rps2*, *Rmd*, and *Rj2* into linkage group J of the soybean molecular map. J Hered.

[CR432] Prabhu RR, Njiti VN, Bell-Johnson B (1999). Selecting soybean cultivars for dual resistance to soybean cyst nematode and sudden death syndrome using two DNA markers. Crop Sci.

[CR433] Prein AF, Mearns LO (2021). US extreme precipitation weather types increased in frequency during the 20th century. J Geophys Res Atmos.

[CR434] Purvis M (2019) Developing management strategies for taproot decline, *Xylaria* sp. Soybean LSU Master’s Theses 4982

[CR435] Qiu BX, Arelli PR, Sleper DA (1999). RFLP markers associated with soybean cyst nematode resistance and seed composition in a 'Peking' x 'Essex' population. Theor Appl Genet.

[CR436] Rahman MT, Rubayet MT, Bhuiyan MKA (2020). Integrated management of rhizoctonia root rot disease of soybean caused by *Rhizoctonia solani*. Nipp J Environ Sci.

[CR437] Ray JD, Morel W, Smith JR (2009). Genetics and mapping of adult plant rust resistance in soybean PI 587886 and PI 587880A. Theor Appl Genet.

[CR438] Ray JD, Smith JR, Morel W (2011). Genetic resistance to soybean rust in PI567099A is at or near the *Rpp3* locus. J Crop Improv.

[CR439] Rebois RV, Johnson WC, Cairns EJ (1968). Resistance in soybeans, *Glycine max* (L.) Merr. to the reniform nematode. Crop Sci.

[CR440] Rebois RV, Epps JM, Hartwig EE (1970). Correlation of resistance in soybeans to *Heterodera glycines* and *Rotylenchulus reniformis*. Phytopathology.

[CR441] Reddy MSS, Ghabrial SA, Redmond CT (2001). Resistance to bean pod mottle virus in transgenic soybean lines expressing the capsid polyprotein. Phytopathology.

[CR442] Ren Q, Pfeiffer TW, Ghabrial SA (1997). Soybean mosaic virus incidence level and infection time: Interaction effects on soybean. Crop Sci.

[CR443] Ren Q, Pfeiffer TW, Ghabrial SA (1997). Soybean mosaic virus resistance improves productivity of double-cropped soybean. Crop Sci.

[CR444] Rensburg JC, Lamprecht SC, Groenewald JZ (2006). Characterisation of *Phomopsis* spp. associated with die-back of rooibos (*Aspalathus linearis*) in South Africa. Stud Mycol.

[CR445] Reznikov S, Chiesa MA, Pardo EM (2019). Soybean-*Macrophomina phaseolina*-specific interactions and identification of a novel source of resistance. Phytopathol.

[CR446] Ribaut JM, Hoisington D (1998). Marker-assisted selection: new tools and strategies. Trends Plant Sci.

[CR447] Riggs RD, Schmitt DP, Wrather JA, Riggs RD (2004). History and distribution. Biology and management of soybean cyst nematode.

[CR448] Rincker K, Lipka AE, Diers BW (2016). Genome-wide association study of brown stem rot resistance in soybean across multiple populations. Plant Genome.

[CR449] Rincker K, Hartman GL, Diers BW (2016). Fine mapping of resistance genes from five brown stem rot resistance sources in soybean. Plant Genome.

[CR450] Rivera YR, Thiessen L (2020) Soybean disease information: Reniform nematode of soybean. North Carolina State Extension Publications. https://content.ces.ncsu.edu/reniform-nematode-of-soybean

[CR451] Roane CW, Tolin SA, Buss GR (1983). Inheritance of reaction to two viruses in the soybean cross ‘York’ × ‘Lee 68’. J Hered.

[CR452] Robbins RT (1982). Description of *Hoplolaimus magnistylus* n. sp. (Nematoda: *Hoplolaimidae*). J Nematol.

[CR453] Robbins RT, Rakes L, Elkins CR (1994). Reproduction of the reniform nematode on thirty soybean cultivars. J Nematol.

[CR454] Robbins RT, Rakes L, Elkins CR (1994). Reniform nematode reproduction and soybean yield of four soybean cultivars in Arkansas. J Nematol (suppl).

[CR455] Robbins RT, Rakes L (1996). Resistance to the reniform nematode in selected soybean cultivars and germplasm lines. J Nematol.

[CR456] Robbins RT, Rakes L, Jackson LE, Dombek DG (1999). Reniform nematode resistance in selected soybean cultivars. J Nematol.

[CR457] Robbins RT (2013) Reniform nematode, a southern problem. Soybean Breeder’s Workshop. February 11–13, St. Louis, Missouri, USA

[CR458] Robinson AF, Inserra RN, Caswell-Chen EP (1997). *Rotylenchulus* species: identification, distribution, host ranges, and crop plant resistance. Nematropica.

[CR459] Rodriguez RG, Thiessen L (2020) Soybean disease information: bean pod mottle virus. NC state extension publications. Accessed 2 Sept 2020. https://content.ces.ncsu.edu/bean-pod-mottle-virus

[CR460] Rojas-Flechas JA (2016) Diversity of oomycetes associated with soybean seedling diseases. Dissertation, Michigan State University

[CR703] Rojas JA, Jacobs JL, Napieralski S (2017). Oomycete species associated with soybean seedlings in North America—Part I: Identification and pathogenicity characterization. Phytopathology.

[CR461] Rolling W, Lake R, Dorrance AE, McHale LK (2020). Genome-wide association analyses of quantitative disease resistance in diverse sets of soybean [*Glycine max* (L.) Merr.] plant introductions. PLoS ONE.

[CR462] Ross JP, Brim CA (1957). Resistance of soybeans to the soybean cyst nematode as determined by a double-row method. Plant Dis Rep.

[CR463] Ross JP (1968). Effect of single and double infections of soybean mosaic and bean pod mottle viruses on soybean yield and seed characters. Plant Dis Rep.

[CR464] Ross JP (1977). Effect of aphid-transmitted soybean mosaic virus on yields of closely related resistant and susceptible soybean lines. Crop Sci.

[CR465] Ross JP (1986). Registration of four soybean germplasm lines resistant to BPMV. Crop Sci.

[CR466] Rosso ML, Rupe JC, Chen P, Mozzoni LA (2008). Inheritance and genetic mapping of resistance to Pythium damping-off caused by *Pythium aphanidermatum* in ‘Archer’soybean. Crop Sci.

[CR467] Roth MG, Webster RW, Mueller DS (2020). Integrated management of important soybean pathogens of the United States in changing climate. J Integr Pest Manag.

[CR468] Roy KW, Abney TS (1988). Colonization of pods and infection of seeds by *Phomopsis longicolla* in susceptible and resistant soybean lines inoculated in the greenhouse. Can J Plant Pathol.

[CR469] Roy KW, Keith BC, Andrews CH (1994). Resistance of hard seeded soybean lines to seed infection by *Phomopsis*, other fungi and soybean mosaic virus. Can J Plant Pathol.

[CR470] Roy KW, Hershman DE, Rupe JC, Abney TS (1997). Sudden death syndrome of soybean. Plant Dis.

[CR471] Rupe JC, Weidemann GJ (1986). Pathogenicity of a *Fusarium* sp isolated from soybean plants with sudden death syndrome. Phytopathol.

[CR472] Rupe JC (1989). Frequency and pathogenicity of *Fusarium solani* recovered from soybeans with sudden death syndrome. Plant Dis.

[CR473] Rupe JC, Rothrock CS, Bates G, et al (2011) Resistance to *Pythium* seedling disease in soybean. In: Sudaric A (ed) Soybean: Molecular aspects of breeding. InTech, Rijeka, Croatia, pp 262–275 http://www.intechopen.com/books/soybeanmolecular-aspects-of-breeding/resistance-to-pythium-seedling-disease-in-soybean

[CR474] Rupe JC, Hartman GL, Rupe JC, Sikora EJ, Domier LL, Steffey KL, Davis JA (2016). Stem canker. Compendium of Soybean diseases and pests.

[CR475] Ryley M (2013) Disease threats to the Australian soybean industry. Summer Grains Conference. June 17–19, Gold Coast, Queensland, Australia

[CR476] Saghai Maroof MA, Jeong SC, Gunduz I (2008). Pyramiding of soybean mosaic virus resistance genes by marker-assisted selection. Crop Sci.

[CR477] Sahoo DK, Abeysekara NS, Cianzio SR (2017). A novel *Phytophthora sojae* resistance *Rps12* gene mapped to a genomic region that contains several *Rps* genes. PLoS ONE.

[CR478] Sahoo DK, Das A, Huang X (2021). Tightly linked *Rps12* and *Rps13* genes provide broad-spectrum *Phytophthora* resistance in soybean. Sci Rep.

[CR479] Saito H, Yamashita Y, Sakata N (2021). Covering soybean leaves with cellulose nanofiber changes leaf surface hydrophobicity and confers resistance against *Phakopsora pachyrhizi*. Front Plant Sci.

[CR480] Sandhu D, Gao H, Cianzio S, Bhattacharyya MK (2004). Deletion of a disease resistance nucleotide-binding-site leucine-rich repeat-like sequence is associated with the loss of the Phytophthora resistance gene *Rps4* in soybean. Genetics.

[CR481] Sandhu D, Schallock KG, Rivera-Velez N (2005). Soybean Phytophthora resistance gene *Rps8* maps closely to the *Rps3* region. J of Hered.

[CR482] Sanitchon J, Vanavichit A, Chanprame S (2004). Identification of simple sequence repeat markers linked to sudden death syndrome resistance in soybean. Sci Asia.

[CR483] Santos JM, Vrandečić K, Cosić J (2011). Resolving the *Diaporthe* species occurring on soybean in Croatia. Persoonia.

[CR484] Savary S, Willocquet L, Pethybridge SJ (2019). The global burden of pathogens and pests on major food crops. Nat Ecol Evol.

[CR485] Scandiani M, Ruberti D, O’Donnell K (2004). Recent outbreak of soybean sudden death syndrome caused by *Fusarium virguliforme* and *F. tucumaniae* in Argentina. Plant Dis.

[CR486] Schmutz J, Cannon SB, Schlueter J (2010). Genome sequence of the palaeopolyploid soybean. Nature.

[CR487] Schneider R, Rolling W, Song Q (2016). Genome-wide association mapping of partial resistance to *Phytophthora sojae* in soybean plant introductions from the Republic of Korea. BMC Genom.

[CR488] Schuh W, Hartman GL, Sinclair JB, Rupe JC (1990). Cercospora blight, leaf spot, and purple seed stain. Compendium of soybean diseases.

[CR489] Schuster I, Abdelnoor RV, Marin SRR (2001). Identification of a new major QTL associated with resistance to soybean cyst nematode (*Heterodera glycines*). Theor Appl Genet.

[CR490] Schwenk FW, Nickell CD (1980). Soybean green stem caused by bean pod mottle virus. Plant Dis.

[CR491] Scott K, Balk C, Veney D (2019). Quantitative disease resistance loci towards *Phytophthora sojae* and three species of *Pythium* in six soybean nested association mapping populations. Crop Sci.

[CR492] Sebastian SA, Nickell CD, Gray LE (1983). Sequential screening of soybean plants for resistance to Phytophthora rot and brown stem rot 1. Crop Sci.

[CR493] Seo M, Kang ST, Moon JK (2009). Identification of quantitative trait loci associated with resistance to bacterial pustule (*Xanthomonas axonopodis* pv. glycines) in soybean. Korean J Breed Sci.

[CR494] Sergiienko V, Shyta O, Khudolii A (2021). The effect of fungicides on the development of diseases and soybean yield in the Forest-steppe of Ukraine. Quar Plant Protect.

[CR495] Shakiba E, Chen P, Gergerich R (2012). Reactions of commercial soybean cultivars from the mid-South to soybean mosaic virus. Crop Sci.

[CR496] Shakiba E, Chen P, Shi A (2012). Two novel alleles at the *Rsv3* locus for resistance to soybean mosaic virus in PI 399091 and PI 61947 soybeans. Crop Sci.

[CR497] Shakiba E, Chen P, Shi A (2013). Inheritance and allelic relationships of resistance genes for soybean mosaic virus in ‘Corsica’ and ‘Beeson’ soybean. Crop Sci.

[CR498] Sharma H, Lightfoot DA (2014). Quantitative trait loci underlying partial resistance to *Cerco spora sojina* race 2 detected in soybean seedlings in greenhouse assays. Atlas J Biol.

[CR499] Sharma P (2020) Evaluation of resistance to *Rhizoctonia solani* in soybean and assessment of fungicide sensitivity in isolates from sugar beet and soybean. Dissertation, University of Minnesota

[CR500] Sher SA (1963). Revision of the Hoplolaiminae (Nematoda) II. *Hoplolaimus* Daday, 1905 and *Aorolaimus* n. gen. Nematologica.

[CR501] Shearin ZP, Finnerty SL, Wood ED (2009). A southern root-knot nematode resistance QTL linked to the T-locus in soybean. Crop Sci.

[CR502] Shi A, Chen P, Vierling R (2011). Multiplex single nucleotide polymorphism (SNP) assay for detection of soybean mosaic virus resistance genes in soybean. Theor Appl Genet.

[CR503] Shi Z, Liu S, Noe J (2015). SNP identification and marker assay development for high-throughput selection of soybean cyst nematode resistance. BMC Genom.

[CR504] Silva MFD, Schuster I, Silva JFVD (2007). Validation of microsatellite markers for assisted selection of soybean resistance to cyst nematode races 3 and 14. Pesq Agrop Brasil.

[CR505] Silva DC, Yamanaka N, Brogin RL (2008). Molecular mapping of two loci that confer resistance to Asian rust in soybean. Theor Appl Genet.

[CR506] Sinclair JB (1989). Threats to soybean production in the tropics: red leaf blotch and leaf rust. Plant Dis.

[CR507] Sinclair JB (1993). Control of seedborne pathogens and diseases of soybean seeds and seedlings. Pesticide Sci.

[CR508] Sinegovskaya VT (2021). Scientific provision of an effective development of soybean breeding and seed production in the Russian Far East. Vavilov J Genet Breed.

[CR509] Singh RJ, Hymowitz T (1999). Soybean genetic resources and crop improvement. Genome.

[CR510] Smith AL (1940). Distribution and relation of meadow nematode, *Pratylenchus pratensis* to Fusarium wilt of cotton in Georgia. Phytopathol.

[CR511] Smith DL, Fritz C, Watson Q (2013). First report of Soybean vein necrosis disease caused by *Soybean vein necrosis-associated virus* in Wisconsin and Iowa. Plant Dis.

[CR512] Smith GS, Wyllie TD, Hartman GL, Sinclair JB, Rupe JC (1999). Charcoal rot. Compendium of Soybean diseases.

[CR513] Smith K (2021) Identifying frogeye leaf spot resistance two elite soybean populations and analysis of agronomic traits in resistant lines. Dissertation, Southern Illinois University Carbondale

[CR514] Song Q, Hyten DL, Jia G (2013). Development and evaluation of SoySNP50K, a high-density genotyping array for soybean. PLoS ONE.

[CR515] Song Q, Yan L, Quigley C (2020). Soybean BARCSoySNP6K: an assay for soybean genetics and breeding research. Plant J.

[CR516] Soto N, Hernández Y, Delgado C (2020). Field resistance to *Phakopsora pachyrhizi* and *Colletotrichum truncatum* of transgenic soybean expressing the NmDef02 plant defensin gene. Front Plant Sci.

[CR517] Srisombun S, Supapornhemin P (1993). Inheritance of soybean resistance to purple seed stain. Soybean Genet Newsl.

[CR518] Srour A, Afzal AJ, Blahut-Beatty L (2012). The receptor like kinase at *Rhg1-a/Rfs2* caused pleiotropic resistance to sudden death syndrome and soybean cyst nematode as a transgene by altering signaling responses. BMC Genom.

[CR519] St.Clair DA (2010). Quantitative disease resistance and quantitative resistance loci in breeding. Annu Rev Phytopathol.

[CR520] Staskawicz B, Dahlbeck D, Keen N, Napoli C (1987). Molecular characterization of cloned avirulence genes from race 0 and race 1 of *Pseudomonas syringae* pv. *glycinea*. J Bacteriol.

[CR521] Stasko AK, Wickramasinghe D, Nauth BJ (2016). High-density mapping of resistance QTL toward *Phytophthora sojae*, *Pythium irregulare*, and *Fusarium graminearum* in the same soybean population. Crop Sci.

[CR522] Stewart S, Robertson AE (2012). A modified method to screen for partial resistance to *Phytophthora sojae* in soybean. Crop Sci.

[CR523] Su G, Suh SO, Schneider RW (2001). Host specialization in the charcoal rot fungus, *Macrophomina phaseolina*. Phytopathology.

[CR524] Sugimoto T, Yoshida S, Watanabe K (2007). Identification of SSR markers linked to the Phytophthora resistance gene *Rps1-d* in soybean. Plant Breed.

[CR525] Sugimoto T, Yoshida S, Kaga A (2011). Genetic analysis and identification of DNA markers linked to a novel *Phytophthora sojae* resistance gene in the Japanese soybean cultivar Waseshiroge. Euphytica.

[CR526] Sugimoto T, Kato M, Yoshida S (2012). Pathogenic diversity of *Phytophthora sojae* and breeding strategies to develop Phytophthora-resistant soybeans. Breed Sci.

[CR527] Sun S, Wu X, Zhao J (2011). Characterization and mapping of *RpsYu25*, a novel resistance gene to *Phytophthora sojae*. Plant Breed.

[CR528] Sun S, Kim MY, Van K (2013). QTLs for resistance to Phomopsis seed decay are associated with days to maturity in soybean (*Glycine max*). Theor Appl Genet.

[CR529] Sun J, Li L, Zhao J (2014). Genetic analysis and fine mapping of *RpsJS*, a novel resistance gene to *Phytophthora sojae* in soybean [*Glycine max* (L.) Merr.]. Theor Appl Genet.

[CR530] Sun J, Guo N, Lei J (2014). Association mapping for partial resistance to *Phytophthora sojae* in soybean (*Glycine max* (L.) Merr.). J Genet.

[CR531] Sun M, Jing Y, Zhao X (2020). Genome-wide association study of partial resistance to Sclerotinia stem rot of cultivated soybean based on the detached leaf method. PLoS ONE.

[CR532] Swaminathan S, Abeysekara NS, Liu M (2016). Quantitative trait loci underlying host responses of soybean to *Fusarium virguliforme* toxins that cause foliar sudden death syndrome. Theor Appl Genet.

[CR533] Swaminathan S, Abeysekara NS, Knight JM (2018). Mapping of new quantitative trait loci for sudden death syndrome and soybean cyst nematode resistance in two soybean populations. Theor Appl Genet.

[CR534] Swaminathan S, Das A, Assefa T (2019). Genome wide association study identifies novel single nucleotide polymorphic loci and candidate genes involved in soybean sudden death syndrome resistance. PLoS ONE.

[CR535] Tachibana H, Card LC (1972). Brown stem rot of soybean and its modification by soybean mosaic virus in soybeans. Phytopathology.

[CR536] Tadesse M (2019). Soybean (*Glycine Max* (L.) Merr.) Breeding and management for soybean rust: a review. Int J Novel Res Life Sci.

[CR537] Taguchi-Shiobara F, Fujii K, Sayama T (2019). Mapping versatile QTL for soybean downy mildew resistance. Theor Appl Genet.

[CR538] Tamada T, Goto T, Chiba I, Suwa T (1969). Soybean dwarf, a new virus disease. Jpn J Phytopathol.

[CR539] Tamulonis JP, Luzzi BM, Hussey RS (1997). RFLP mapping of resistance to southern root-knot nematode in soybean. Crop Sci.

[CR540] Tan R, Serven B, Collins PJ (2018). QTL mapping and epistatic interaction analysis of field resistance to sudden death syndrome (*Fusarium virguliforme*) in soybean. Theor Appl Genet.

[CR541] Tan R, Collins PJ, Wang J (2019). Different loci associated with root and foliar resistance to sudden death syndrome (*Fusarium virguliforme*) in soybean. Theor Appl Genet.

[CR542] Tande C, Hadi B, Chowdhury R (2014). First report of sudden death syndrome of soybean caused by *Fusarium virguliforme* in South Dakota. Phytopahology.

[CR543] Tebaldi C, Hayhoe K, Arblaster JM, Meehl GA (2006). Going to the extremes. Clim Change.

[CR544] Tewoldemedhin YT, Lamprecht SC, Geldenhuys JJ, Kloppers FJ (2014). First report of soybean sudden death syndrome caused by *Fusarium virguliforme* in South Africa. Plant Dis.

[CR545] Thickett K, VanDerWal J, Lovett-Doust L, Anderson TR (2007). A method for screening soybean seedlings for resistance to northern stem canker caused by *Diaporthe phaseolorum* var. caulivora. Can J Plant Sci.

[CR546] Thorne G (1935). The sugar beet nematode and other indigenous nemic parasites of shadscale. J Agric Res.

[CR547] Tian Y, Liu B, Shi X (2019). Deep genotyping of the gene *GmSNAP* facilitates pyramiding resistance to cyst nematode in soybean. Crop J.

[CR548] Timper P, Fribourg HA, Hannaway DB, West CP (2009). Nematode. Tall fescue for the twenty-first century.

[CR549] Tooley PW (2017). Development of an inoculation technique and the evaluation of soybean genotypes for resistance to *Coniothyrium glycines*. Plant Dis.

[CR550] Tran DT, Steketee CJ, Boehm JD (2019). Genome-wide association analysis pinpoints additional major genomic regions conferring resistance to soybean cyst nematode (*Heterodera glycines* Ichinohe). Front Plant Sci.

[CR551] Triwitayakorn K, Njiti VN, Iqbal MJ (2005). Genomic analysis of a region encompassing *QRfs1* and *QRfs2*: genes that underlie soybean resistance to sudden death syndrome. Genome.

[CR552] Trudgill DL, Blok VC (2001). Apomictic polyphagous root-knot nematodes: exceptionally successful and damaging biotrophic root pathogens. Annu Rev Phytopathol.

[CR553] Truol G, Laguna IG, Nome SF, Rodríguez Pardina P (1985). Alfalfa mosaic virus (AMV) en cultivos de soja *Glycine max* (L) Merr. IDIA.

[CR554] Tu JC, Buzzell RI (1987). Stem-tip necrosis: a hypersensitive, temperature dependent, dominant gene reaction of soybean to infection by soybean mosaic virus. Can J Plant Sci.

[CR555] Tucker DM, Saghai Maroof MA, Mideros S (2010). Mapping quantitative trait loci for partial resistance to *Phytophthora sojae* in a soybean interspecific cross. Crop Sci.

[CR556] Twizeyimana M, Hill CB, Pawlowski M (2012). A cut-stem inoculation technique to evaluate soybean for resistance to *Macrophomina phaseolina*. Plant Dis.

[CR557] Tyler JM (1996). Characterization of stem canker resistance in ‘Hutcheson’soybean. Crop Sci.

[CR558] Tzanetakis I, We R, Newman M, Hajimorad R (2009). Soybean vein necrosis virus: a new threat to soybean production in Southeastern United States. Phytopathol.

[CR559] Uchibori A, Sasaki J, Takeuchi T (2009). QTL analysis for resistance to soybean dwarf virus in Indonesian soybean cultivar Wilis. Mol Breed.

[CR560] Udayanga D, Castlebury LA, Rossman AY (2015). The *Diaporthe sojae* species complex: phylogenetic re-assessment of pathogens associated with soybean, cucurbits and other field crops. Fungal Biol.

[CR561] Urrea K, Rupe J, Chen P, Rothrock CS (2017). Characterization of seed rot resistance to *Pythium aphanidermatum* in soybean. Crop Sci.

[CR562] Usovsky M, Lakhssassi N, Patil GB (2021). Dissecting nematode resistance regions in soybean revealed pleiotropic effect of soybean cyst and reniform nematode resistance genes. Plant Genome.

[CR563] Van K, Ha BK, Kim MY (2004). SSR mapping of genes conditioning soybean resistance to six isolates of *Xanthomonas axonopodis* pv. *glycines*. Korean J Genet.

[CR564] Van K, Rolling W, Biyashev RM (2020). Mining germplasm panels and phenotypic datasets to identify loci for resistance to *Phytophthora sojae* in soybean. Plant Genome.

[CR565] Varshney RK, Bohra A, Yu J (2021). Designing future crops: genomics-assisted breeding comes of age. Trends Plant Sci.

[CR707] Vibha (2016) Macrophomina phaseolina. The most destructive soybean fungal pathogen of global concern. In: Kumar P, Gupta V, Tiwari A, Kamle M (eds) Current trends in plant disease diagnostics and management practices fungal biology. Springer, New York. 10.1007/978-3-319-27312-9_8

[CR566] Vieira CC, Chen P, Usovsky M (2021). A major quantitative trait locus resistant to southern root-knot nematode sustains soybean yield under nematode pressure. Crop Sci.

[CR567] Vieira CC, Chen P (2021). The numbers game of soybean breeding in the United States. Crop Breed Appl Biotechnol.

[CR568] Vierling RA, Faghihi J, Ferris VR, Ferris JM (1996). Association of RFLP markers with loci conferring broad-based resistance to the soybean cyst nematode (*Heterodera glycines*). Theor Appl Genet.

[CR569] Vuong TD, Diers BW, Hartman GL (2008). Identification of QTL for resistance to *Sclerotinia stem rot* in soybean plant introduction 194639. Crop Sci.

[CR570] Vuong TD, Sleper DA, Shannon JG, Nguyen HT (2010). Novel quantitative trait loci for broad-based resistance to soybean cyst nematode (*Heterodera glycines* Ichinohe) in soybean PI 567516C. Theor Appl Genet.

[CR571] Vuong TD, Sleper DA, Shannon JG (2011). Confirmation of quantitative trait loci for resistance to multiple-HG types of soybean cyst nematode (*Heterodera glycines* Ichinohe). Euphytica.

[CR572] Vuong TD, Sonah H, Meinhardt CG (2015). Genetic architecture of cyst nematode resistance revealed by genome-wide association study in soybean. BMC Genom.

[CR573] Vuong TD, Sonah H, Patil G (2021). Identification of genomic loci conferring broad-spectrum resistance to multiple nematode species in exotic soybean accession PI 567305. Theor Appl Genet.

[CR574] Vuong TD, Walker DR, Nguyen BT (2016). Molecular characterization of resistance to soybean rust (*Phakopsora pachyrhizi* Syd. & Syd.) in soybean cultivar DT 2000 (PI 635999). PLoS ONE.

[CR575] Walker DR, Boerma HR, Phillips DV (2011). Evaluation of USDA soybean germplasm accessions for resistance to soybean rust in the southern United States. Crop Sci.

[CR576] Walker DR, Harris DK, King ZR (2014). Evaluation of soybean germplasm accessions for resistance to *Phakopsora pachyrhizi* populations in the southeastern United States, 2009–2012. Crop Sci.

[CR577] Walters HJ (1958). A virus disease complex in soybeans in Arkansas. (abstr.) Phytopahology.

[CR578] Walters HJ (1980). Soybean leaf blight caused by *Cercospora kikuchii*. Plant Dis.

[CR579] Walters HJ, Shibles R (1985). Purple seed stain and Cercosporin leaf blight. World soybean research conference III: proceedings.

[CR580] Walters HJ, Caviness CE (1973). Breeding for improved soybean seed quality. Arkansas Farm Res.

[CR581] Wang D, Ma Y, Liu N (2011). Fine mapping and identification of the soybean *R*_*SC4*_ resistance candidate gene to soybean mosaic virus. Plant Breed.

[CR582] Wang H, StMartin SK, Dorrance AE (2012). Comparison of phenotypic methods and yield contributions of quantitative trait loci for partial resistance to *Phytophthora sojae* in soybean. Crop Sci.

[CR583] Wang H, Waller L, Tripathy S (2010). Analysis of genes underlying soybean quantitative trait loci conferring partial resistance to *Phytophthora sojae*. Plant Genome.

[CR584] Wang J (2016) Molecular diagnostics, epidemiology, and population genetics of the soybean sudden death syndrome pathogen, *Fusarium virguliforme*. Dissertation, Michigan State University.

[CR585] Wang J, Jacobs JL, Roth MG, Chilvers MI (2019). Temporal dynamics of Fusarium virguliforme colonization of soybean roots. Plant Dis.

[CR586] Wang TC, Hartman GL (1992). Epidemiology of soybean rust and breeding for host resistance. Plant Protect Bull.

[CR587] Wang W, Chen L, Fengler K (2021). A giant NLR gene confers broad-spectrum resistance to *Phytophthora sojae* in soybean. Nat Commun.

[CR588] Wang X, Eggenberger AL, Nutter FW, Hill JH (2001). Pathogen-derived transgenic resistance to soybean mosaic virus in soybean. Mol Breed.

[CR589] Weaver DB, Sedhom SA, Smith EF, Backman PA (1988). Field and greenhouse evaluations of stem canker resistance in soybean. Crop Sci.

[CR590] Webb DM (1995) Quantitative trait loci associated with cyst nematode resistance and uses thereof. US Patent Application, US20110083224 A1

[CR591] Wei W, Mesquita ACO, Figueiró ADA (2017). Genome-wide association mapping of resistance to a Brazilian isolate of *Sclerotinia sclerotiorum* in soybean genotypes mostly from Brazil. BMC Genom.

[CR592] Wen Z, Tan R, Yuan J (2014). Genome-wide association mapping of quantitative resistance to sudden death syndrome in soybean. BMC Genom.

[CR593] Wen Z, Tan R, Zhang S (2018). Integrating GWAS and gene expression data for functional characterization of resistance to white mould in soya bean. Plant Biotechnol J.

[CR594] Weng C, Yu K, Anderson TR, Poysa V (2001). Mapping genes conferring resistance to Phytophthora root rot of soybean, *Rps1a* and *Rps7*. J Hered.

[CR595] Weng C, Yu K, Anderson TR, Poysa V (2007). A quantitative trait locus influencing tolerance to Phytophthora root rot in the soybean cultivar ‘Conrad’. Euphytica.

[CR596] Whitham SA, Qi M, Innes RW (2016). Molecular soybean-pathogen interactions. Annu Rev Phytopathol.

[CR597] Wilcox JR, Laviolette FA, Martin RJ (1975). Heritability of purple seed stain resistance in soybeans. Crop Sci.

[CR598] Wilkes J, Saski C, Klepadlo M (2020). Quantitative trait loci associated with *Rotylenchulus reniformis* host suitability in soybean. Phytopathology.

[CR599] Williams DJ, Nyvall RF (1980). Leaf infection and yield losses caused by brown spot and bacterial blight diseases of soybean. Phytopathology.

[CR600] Willmot DB, Nickell C (1989). Genetic analysis of brown stem rot resistance in soybean. Crop Sci.

[CR601] Winstead NN, Skotland CB, Sasser JN (1955). Soybean cyst nematode in North Carolina. Plant Dis Rep.

[CR602] Wrather JA, Chambers AY, Fox JA (1995). Soybean disease loss estimates for the southern United States, 1974 to 1994. Plant Dis.

[CR603] Wrather JA, Anderson TR, Arsyad DM (1997). Soybean disease loss estimates for the top 10 soybean producing countries in 1994. Plant Dis.

[CR604] Wrather JA, Anderson TR, Arsyad DM (2001). Soybean disease loss estimates for the top ten soybean-producing countries in 1998. Can J Plant Pathol.

[CR605] Wrather JA, Koenning SR, Anderson TR (2003). Effect of diseases on soybean yields in the United States and Ontario (1999 to 2002). Plant Heal Prog.

[CR606] Wrather JA, Sleper DA, Stevens WE (2003). Planting date and cultivar effects on soybean yield, seed quality, and *Phomopsis* sp. seed infection. Plant Dis.

[CR607] Wrather JA, Koenning SR (2009) Effects of diseases on soybean yields in the United States 1996 to 2007. Plant Health Prog. https://www.plantmanagementnetwork.org/pub/php/research/2009/yields/PMC2586459 19259444

[CR608] Wrather A, Shannon G, Balardin R (2010). Effect of diseases on soybean yield in the top eight producing countries in 2006. Plant Health Prog.

[CR609] Wu X, Zhang B, Shi S (2011). Identification, genetic analysis and mapping of resistance to *Phytophthora sojae* of *Pm28* in soybean. Agric Sci China.

[CR610] Wu X, Zhou B, Sun S (2011). Genetic analysis and mapping of resistance to *Phytophthora sojae* of *Pm14* in soybean. Sci Agricult Sin.

[CR611] Wu X, Zhou B, Zhao J (2011). Identification of quantitative trait loci for partial resistance to *Phytophthora sojae* in soybean. Plant Breed.

[CR612] Xu X, Zeng L, Tao Y (2013). Pinpointing genes underlying the quantitative trait loci for root-knot nematode resistance in palaeopolyploid soybean by whole genome resequencing. PNAS.

[CR613] Xue AG, Cober E, Voldeng HD (2007). Evaluation of the pathogenicity of *Fusarium graminearum* and *Fusarium pseudograminearum* on soybean seedlings under controlled conditions. Can J Plant Pathol.

[CR614] Yamanaka N, Fuentes FH, Gilli JR (2006). Identification of quantitative trait loci for resistance against soybean sudden death syndrome caused by *Fusarium tucumaniae*. Pesq Agrop Brasil.

[CR615] Yamanaka N, Yamaoka Y, Kato M (2010). Development of classification criteria for resistance to soybean rust and differences in virulence among Japanese and Brazilian rust populations. Trop Plant Pathol.

[CR616] Yamanaka N, Hossain MM, Yamaoka Y (2015). Molecular mapping of Asian soybean rust resistance in Chinese and Japanese soybean lines, Xiao Jing Huang, Himeshirazu, and Iyodaizu B. Euphytica.

[CR617] Yamanaka N, Morishita M, Mori T (2015). Multiple *Rpp*-gene pyramiding confers resistance to Asian soybean rust isolates that are virulent on each of the pyramided genes. Trop Plant Pathol.

[CR618] Yamanaka N, Morishita M, Mori T (2016). The locus for resistance to Asian soybean rust in PI 587855. Plant Breed.

[CR619] Yamashita Y, Takeuchi T, Ohnishi S (2013). Fine mapping of the major soybean dwarf virus resistance gene *Rsdv1* of the soybean cultivar ‘Wilis’. Breed Sci.

[CR620] Yan GP, Plaisance A, Huang D, Handoo ZA (2016). First report of the lance nematode *Hoplolaimus stephanus* from a soybean field in North Dakota. Plant Dis.

[CR621] Yan H, Wang H, Cheng H (2015). Detection and fine-mapping of soybean mosaic virus resistance genes via linkage and association analysis in soybean. J Integr Plant Biol.

[CR622] Yang K, Lee YH, Ko JM (2011). Development of molecular markers conferring bacterial leaf pustule resistance gene, *rxp*, using resistant and susceptible cultivars in soybean. Korean J Breed Sci.

[CR623] Yang X, Uphoff M, Sanogo S (2001). Outbreaks of soybean frogeye leaf spot in Iowa. Plant Dis.

[CR624] Yang X, Niu L, Zhang W (2019). Increased multiple virus resistance in transgenic soybean overexpressing the double-strand RNA-specific ribonuclease gene PAC1. Transgenic Res.

[CR625] Yang Y, Zheng G, Han L (2013). Genetic analysis and mapping of genes for resistance to multiple strains of soybean mosaic virus in a single resistant soybean accession PI 96983. Theor Appl Genet.

[CR626] Yao HY, Wang XM, Wu XF (2010). Molecular mapping of *Phytophthora* resistance gene in soybean cultivar Zaoshu18. J Plant Genet Res.

[CR627] Yorinori JT (1999) Situação atual das enfermidades de soja na Bolivia, Brasil e Paraguai. Proceedings, Mercosoja 99, Rosario, Argentina, June 21–25

[CR628] Yorinori JT (2002) Situacao atual das doencas potenciais no cone sur. In: Proceedings, II Brazilian Soybean Congress-Mercosoja 2002, Foz do Iguazu, Brazil, June 3–6

[CR629] Yorinori JT, Paiva WM, Frederick RD (2005). Epidemics of soybean rust (*Phakopsora pachyrhizi*) in Brazil and Paraguay from 2001 to 2003. Plant Dis.

[CR630] Young LD (1998). *Heterodera glycines* populations selected for reproduction on Hartwig soybean. J Nematol.

[CR701] Yu YG, Maroof MA, Buss GR (1996). Divergence and allelomorphic relationship of a soybean virus resistance gene based on tightly linked DNA microsatellite and RFLP markers. Theor Appl Genet.

[CR631] Yu A, Xu P, Wang J (2010). Genetic analysis and SSR mapping of gene resistance to *Phytophthora sojae* race 1 in soybean cv Suinong 10. Chin J Oil Crop Sci.

[CR632] Yu C, Miao R, Khanna M (2021). Maladaptation of US corn and soybeans to a changing climate. Sci Rep.

[CR633] Yu N, Lee TG, Rosa DP (2016). Impact of *Rhg1* copy number, type, and interaction with *Rhg4* on resistance to *Heterodera glycines* in soybean. Theor Appl Genet.

[CR634] Yuan J, Bashir R, Salas G (2012). New approaches to selecting resistance or tolerance to SDS and Fusarium root rot. Plant Genet Genom Biotechnol.

[CR635] Yue P, Arelli PR, Sleper DA (2001). Molecular characterization of resistance to *Heterodera glycines* in soybean PI 438489B. Theor Appl Genet.

[CR636] Yue P, Sleper DA, Arelli PR (2001). Mapping resistance to multiple races of *Heterodera glycines* in soybean PI 89772. Crop Sci.

[CR637] Zambrana-Echevarria C (2021) Development of tools for the management of soybean vein necrosis orthotospovirus and tobacco streak illavirus in soybean (*Glycine max* (L.) Merr.). Dissertation, University of Wisconsin

[CR638] Zhang BQ, Chen WD, Yang XB (1998). Occurrence of *Pythium* species in long-term maize and soybean monoculture and maize/soybean rotation. Mycol Res.

[CR639] Zhang BQ, Yang XB (2000). Pathogenicity of *Pythium* populations from corn-soybean rotation fields. Plant Dis.

[CR640] Zhang C, Han Y, Qu Y (2020). Identification of quantitative trait loci underlying resistance of soybean to *Fusarium graminearum*. Plant Breed.

[CR641] Zhang C, Zhao X, Qu Y (2019). Loci and candidate genes in soybean that confer resistance to *Fusarium graminearum*. Theor Appl Genet.

[CR642] Zhang J, Singh A, Mueller DS, Singh AK (2015). Genome-wide association and epistasis studies unravel the genetic architecture of sudden death syndrome resistance in soybean. Plant J.

[CR643] Zhang J, Wen Z, Li W (2017). Genome-wide association study for soybean cyst nematode resistance in Chinese elite soybean cultivars. Mol Breed.

[CR644] Zhang J, Xia C, Duan C (2013). Identification and candidate gene analysis of a novel Phytophthora resistance gene *Rps10* in a Chinese soybean cultivar. PLoS ONE.

[CR645] Zhang J, Xia C, Wang X (2013). Genetic characterization and fine mapping of the novel Phytophthora resistance gene in a Chinese soybean cultivar. Theor Appl Genet.

[CR646] Zhang K, Ren R, Wang Y (2015). The symptom types in soybean leaves caused by soybean mosaic virus. Soybean Sci.

[CR647] Zhang MH, Lyu WQ, Zhong ZX (1980). Types and identification of the pathogen of soybean virus diseases. Acta Phytopathol Sin.

[CR648] Zhang X, Sato S, Ye X (2011). Robust RNAi-based resistance to mixed infection of three viruses in soybean plants expressing separate short hairpins from a single transgene. Phytopathology.

[CR649] Zhao G, Ablett GR, Anderson TR (2005). Inheritance and genetic mapping of resistance to Rhizoctonia root and hypocotyl rot in soybean. Crop Sci.

[CR650] Zhao L, Wang D, Zhang H (2016). Fine mapping of the *R*_*SC8*_ locus and expression analysis of candidate SMV resistance genes in soybean. Plant Breed.

[CR651] Zhao X, Bao D, Wang W (2020). Loci and candidate gene identification for soybean resistance to Phytophthora root rot race 1 in combination with association and linkage mapping. Mol Breed.

[CR652] Zhao X, Han Y, Li Y (2015). Loci and candidate gene identification for resistance to *Sclerotinia sclerotiorum* in soybean (*Glycine max L.* Merr.) via association and linkage maps. Plant J.

[CR653] Zhao X, Teng W, Li Y (2017). Loci and candidate genes conferring resistance to soybean cyst nematode HG type 2.5.7. BMC Genom.

[CR654] Zheng C, Chen P, Gergerich R (2005). Characterization of resistance to soybean mosaic virus in diverse soybean germplasm. Crop Sci.

[CR655] Zheng C, Chen P, Hymowitz T (2005). Evaluation of Glycine species for resistance to bean pod mottle virus. Crop Prot.

[CR656] Zhu Z, Huo Y, Wang X (2007). Molecular identification of a novel Phytophthora resistance gene in soybean. Acta Agron Sin.

[CR657] Zhong C, Li Y, Sun S (2019). Genetic mapping and molecular characterization of a broad-spectrum *Phytophthora sojae* resistance gene in Chinese soybean. Int J Mol Sci.

[CR658] Zhong C, Sun S, Li Y (2018). Next-generation sequencing to identify candidate genes and develop diagnostic markers for a novel Phytophthora resistance gene, *RpsHC18*, in soybean. Theor Appl Genet.

[CR659] Zhong C, Sun S, Yao L (2018). Fine mapping and identification of a novel Phytophthora root rot resistance locus *RpsZS18* on chromosome 2 in soybean. Front Plant Sci.

[CR660] Zhong C, Sun S, Zhang X (2020). Fine mapping, candidate gene identification and co-segregating marker development for the Phytophthora root rot resistance gene *RpsYD25*. Front Genet.

[CR661] Zhou J (2012) Characterization and epidemiology of Soybean vein necrosis associated virus. Dissertation, University of Arkansas

[CR662] Zhou J, Aboughanem-Sabanadzovic N, Sabanadzovic S, Tzanetakis IE (2018). First report of soybean vein necrosis virus infecting kudzu (*Pueraria montana*) in the United States of America. Plant Dis.

[CR663] Zhou J, Tzanetakis IE (2013). Epidemiology of Soybean vein necrosis-associated virus. Phytopathology.

[CR664] Zhou J, Tzanetakis IE (2020). Transmission blockage of an orthotospovirus using synthetic peptides. J Gen Virol.

[CR665] Zimmerman MS, Minor HC (1993). Inheritance of Phomopsis seed decay resistance in soybean PI 417479. Crop Sci.

[CR666] Zou J, Li W, Zhang Y (2021). Identification of glutathione transferase gene associated with partial resistance to Sclerotinia stem rot of soybean using genome-wide association and linkage mapping. Theor Appl Genet.

