# Correction to: Breeding for disease resistance in soybean: a global perspective

**DOI:** 10.1007/s00122-022-04226-5

**Published:** 2022-10-31

**Authors:** Feng Lin, Sushil Satish Chhapekar, Caio Canella Vieira, Marcos Paulo Da Silva, Alejandro Rojas, Dongho Lee, Nianxi Liu, Esteban Mariano Pardo, Yi-Chen Lee, Zhimin Dong, Jose Baldin Pinheiro, Leonardo Daniel Ploper, John Rupe, Pengyin Chen, Dechun Wang, Henry T. Nguyen

**Affiliations:** 1 Department of Plant, Soil and Microbial Sciences, Michigan State University, East Lansing, MI 48824 USA; 2 Division of Plant Sciences and National Center for Soybean Biotechnology, University of Missouri-Columbia, Columbia, MO 65211 USA; 3 Fisher Delta Research Center, University of Missouri, Portageville, MO 63873 USA; 4 Department of Entomology and Plant Pathology, University of Arkansas, Fayetteville, AR 72701 USA; 5 Soybean Research Institute, Jilin Academy of Agricultural Sciences, Changchun,, 130033 Jilin China; 6 Instituto de Tecnología Agroindustrial del Noroeste Argentino (ITANOA) [Estación Experimental Agroindustrial Obispo Colombres (EEAOC) – Consejo Nacional de Investigaciones Científicas y Técnicas (CONICET)], Av. William Cross 3150, C.P. T4101XAC, Las Talitas, Tucumán, Argentina; 7 Departamento de Genética, Escola Superior de Agricultura “Luiz de Queiroz” (ESALQ/USP), PO Box 9, Piracicaba, SP 13418-900 Brazil

## Correction to: Theoretical and Applied Genetics 10.1007/s00122-022-04101-3

Due to the publisher’s error, Table 16 was repeatedly used for ‘Rhizoctonia damping off and root rot’ (Page 69) and ‘Bacteria diseases’ (Page 70), while the table for ‘Rhizoctonia damping off and root rot’ was missing. Please see the following for the correct table for Rhizoctonia damping off and root rot.

In addition, the Plant Introduction (PI) names and soybean germplasm names in the text and tables should not contain comma (for example, PI 96354 should be corrected as PI 96354; S06-13640 should be corrected as S06-13640). Although the author team pointed this out during the proof reading process, unfortunately, these errors were not corrected in the published text. We apologize for any inconvenience caused by misunderstanding of the germplasm names.

The original article has been corrected.

**Table 16:** Soybean loci conferring resistance to Rhizoctonia damping off and root rot (caused by *Rhizoctonia solani*)

MLG (Chr.)	Locus name	Tightly linked/flanking markers	Marker position cM (bp)^a^	Testing methods/resistance spectrum	Population type (size)	PVE^b^	Donor source	Reference
MLG C2 (Chr. 6)	–	Satt281	6,529,270	Greenhouse test/AG-4	F2(189), F4:5(23), F4:5(32)	11–39%	PI 442031	Zhao et al. (2005)
MLG M (Chr. 7)	–	Satt245	9,357,717	Greenhouse test/AG-4	F2(189), F4:5(23), F4:5(32)	6.8–14%	PI 442031	Zhao et al. (2005)
MLG A2 (Chr. 8)	–	Satt177	36.77cM*	Greenhouse test/AG-4	F2(189), F4:5(23), F4:5(32)	7–23%	PI 442031	Zhao et al. (2005)

*GmComposite2003 genetic position (www.soybase.org)

^a^Marker position (bp) based on the *Glycine max* genome assembly version *Gmax2.0*

^b^Phenotypic variations explained by the molecular markers

