# Supplementary material for: Breeding for disease resistance in soybean: a global perspective
Source: Theor Appl Genet. 2022 Jul 5;135(11):3773–872. doi: 10.1007/s00122-022-04101-3 (PMC9729162; doi:10.1007/s00122-022-04101-3)
Supplement: Supplementary file 1 — Supplementary file1 (DOCX 63 kb) [file 122_2022_4101_MOESM1_ESM.docx]

**Supplementary Table 3** Additional soybean loci conferring resistance to Sclerotinia stem rot (caused by *Sclerotinia sclerotiorum*)

| **MLG (Chr.)** | **Locus name** | **Tightly linked / flanking markers** | **Marker position cM (bp) ^a^** | **Testing methods / Resistance spectrum** | **Population type (size)** | **PVE ^b^** | **Donor source** | **References** |
| --- | --- | --- | --- | --- | --- | --- | --- | --- |
| MLG D1a (Chr. 1) | - | ss715578907 | (2,915,019 a2) | Greenhouse test | USDA germplasm collection (474) | - | - | Moellers et al. 2017 |
|  | - | ss715579141 | (3,597,388 a2) | Greenhouse test | USDA germplasm collection (474) | - | - | Moellers et al. 2017 |
|  | *Qsp-1* | Satt502 and Sat_159 | (24,851,104 a2 - 8,541,268 a1) | Greenhouse test | F5:6 (149) | 10.4-12.6% | MapleArrow | Li et al. 2010 |
|  | - | Gm01:28271068 | (28,271,068 a2) | Detached leaf method | China landraces (38), elite cultivars (147) | 6.7-7.6% | Allele G | Sun et al. 2020 |
|  | - | - | (29,185,984 a1) | Greenhouse test / strain *NB-5* | Breeding lines (130) | 7.3% | - | Bastien et al. 2014 |
|  | *QTL6, Sclero 2-5; Sclero 3-4; Sclero 4-1; Sclero 5-3* | Satt147 and Satt129 | (54,828,484 - 55,166,202 a2) | Detached leaf method / isolate *143* | F5 (400) | 4-10% | Corsoy79, Dassel, DSR173, S19-90 | Arahana et al. 2001 |
| MLG D1b (Chr. 2) | - | Gm02:360091 | (360,091 a2) | Cut stem method | China accessions (185) | 8% | Allele T | Jing et al. 2021 |
|  | - | ss715581133 | (1,272,176 a2) | Greenhouse test | USDA germplasm collection (474) | - | - | Moellers et al. 2017 |
|  | - | ss715581558 | (2,358,875 a2) | Greenhouse test | USDA germplasm collection (474) | - | - | Moellers et al. 2017 |
|  | - | ss715583594 | (5,417,832 a2) | Field inoculation (IA, US) | USDA germplasm collection (474) | - | - | Moellers et al. 2017 |
|  | *Locus1* | ss715583735 | (6,447,172 a2) | Field test (MI, US), greenhouse test / isolate *105HT* | Improved lines (962) | 5.3% | - | Wen et al. 2018 |
|  | - | Gm02:25872635 | (25,872,635 a2) | Cut stem method | China accessions (185) | 6-8% | Allele A | Jing et al. 2021 |
|  | - | Gm02:29817148 | (29,817,148 a2) | Detached leaf method | China landraces (38), elite cultivars (147) | 5.8-10.0% | Allele T | Sun et al. 2020 |
|  | *QTL7, Sclero 2-6* | Satt172 | (43,443,481 a2) | Detached leaf method / isolate *143* | F5 (100) | 4-10% | Williams82 | Arahana et al. 2001 |
|  | *QTL8, Sclero 2-7; Sclero 3-5; Sclero 5-4; Sclero 6-3* | Satt459 | (45,311,085 a2) | Detached leaf method / isolate *143* | F5 (400) | 4-10% | Williams82, S19-90, Vinton81 | Arahana et al. 2001 |
|  | - | Gm02:46172174 | (46,172,174 a2) | Cut stem method | China accessions (185) | 7-8% | Allele C | Jing et al. 2021 |
| MLG N (Chr. 3) | - | ss715584553 | (164,189 a2) | Greenhouse test | USDA germplasm collection (474) | - | - | Moellers et al. 2017 |
|  | *qLLS3-1* | Block456-Block455 | 104.70cM (2,173,694-2,334,790 a2) | Greenhouse test | F5:20 (149) | 1.1% | Maple Arrow | Zou et al. 2021 |
|  | *QTL24, Sclero 2-21; Sclero 3-15; Sclero 4-9; Sclero 5-13; Sclero 6-10* | Satt009 | (3,931,955 a2) | Detached leaf method / isolate *143* | F5 (400) | 4-10% | Dassel, DSR173, S19-90, Vinton81, Williams82 | Arahana et al. 2001 |
|  | - | - | (5,216,761 a2) | Greenhouse test | CNSGB germplasm (261) | - | Allele A | Zou et al. 2021 |
|  | - | Gm03:27991148 | (27,991,148 a2) | Detached leaf method | China landraces (38), elite cultivars (147) | 8.2-8.6% | Allele C | Sun et al. 2020 |
|  | - | - | (29,889,328 a2) | Greenhouse test | CNSGB germplasm (261) | - | Allele A | Zou et al. 2021 |
|  | *QTL25, Sclero 3-16* | Satt387 | (34,554,705 a2) | Detached leaf method / isolate *143* | F5 (100) | 4-10% | Williams82 | Arahana et al. 2001 |
|  | - | Gm03:40558623 | (40,558,623 a2) | Cut stem method | China accessions (185) | 7-8% | Allele A | Jing et al. 2021 |
|  | - | - | (44,735,630 a1) | Greenhouse test | Germplasm and elite lines (101) | 21% | - | Iquira et al. 2015 |
| MLG C1 (Chr. 4) | *Locus2* | ss715587841ss715587850 ss715587866 | (3,732,457 / 3,752,035 / 3,797,774 a2) | Field test (MI, US), greenhouse test / isolate *105HT* | Improved lines (962) | 5.1-5.3% | - | Wen et al. 2018 |
|  | - | S4_7,210,961 | (7,210,961 a1) | Greenhouse test / isolate *Jatai* | Brazil breeding lines (275) | 2.3% | - | Wei et al. 2017 |
|  | - | ss715588567 | (48,208,640 a2) | Greenhouse test | USDA germplasm collection (474) | - | - | Moellers et al. 2017 |
|  | - | - | (51,367,426 a2) | Greenhouse test | CNSGB germplasm (261) | - | Allele A | Zou et al. 2021 |
|  | - | SATT391 patent | 50.0cM | - |  | - | - | Sebastian et al. 2010 |
| MLG A1 (Chr. 5) | *Locus4* | ss715590176 | (3,924,139 a2) | Field test (MI, US), greenhouse test / isolate *105HT* | Improved lines (962) | 6.9% | - | Wen et al. 2018 |
|  | - | Gm05:14834789 | (14,834,789 a2) | Detached leaf method | China landraces (38), elite cultivars (147) | 3.4% - 10.7% | Allele G | Sun et al. 2020 |
|  | - | Satt155, SLS1C.L24 (Patent) | (29,529,125 a2) | greenhouse | - | 14.0% | P^S^ | Han et al. 2007 |
|  | *Locus9, Sclero 2-1* | ss715590828 | (33,208,876 a2) | Field test (MI, US), greenhouse test / isolate *105HT* | USDA germplasm collection (405) | 5.2% | - | Wen et al. 2018 |
|  | - | ss715590908 | (33,836,740 a2) | Field inoculation (IA, US) | USDA germplasm collection (474) | - | - | Moellers et al. 2017 |
|  | *QTL1, Sclero 2-1; Sclero 3-1; Sclero 6-1* | Satt545 | (36,463,025 a2) | Detached leaf method / isolate *143* | F5 (300) | 4-10% | Corsoy79, Williams82, Vinton81 | Arahana et al. 2001 |
| MLG C2 (Chr. 6) | - | rs2707217 | (2,707,217 a1) | Greenhouse test | China core germplasm collection (330) | 7.4-8.1% | Allele G | Zhao et al. 2015 |
|  | - | Gm06:789641 | (789,641 a2) | Cut stem method | China accessions (185) | 7-8% | Allele T | Jing et al. 2021 |
|  | *Sclero 7-2* | Satt363 and Satt658 | (20,754,844 a2) | Field inoculation (QC, Canada) / strain *NB-5* | F4 derived RILs (180) | 5.7-15.4% | Maple Donovan | Huynh et al. 2010 |
|  | *Sclero 7-1* | Gm06:39027181 | (39,027,181 a2) | Detached leaf method | China landraces (38), elite cultivars (147) | 6.7-7.2% | Allele G | Sun et al. 2020 |
|  | - | ss715595168 | (50,255,578 a2) | Greenhouse test | USDA germplasm collection (474) | - | - | Moellers et al. 2017 |
|  | *Sclero 1-4* | OAA09_600 | - | Field test | F3 derived RILs (252) | 7.8% | S19-90 | Kim and Diers 2000 |
| MLG M (Chr. 7) | - | Gm07:529324 | (529,324 a2) | Detached leaf method | China landraces (38), elite cultivars (147) | 4.8-8.4% | Allele T | Sun et al. 2020 |
|  | - | Satt540-Satt323 | (5,010,696 - 10,465,123 a2) | Infested oat seed cotyledon assay and cut stem assay | BC1F4:5 (230) | 9.0% | Kottman | Guo et al. 2008 |
|  | *Locus10, Sclero 1-2* | ss715596286 | (10,514,582 a2) | Field test (MI, US), greenhouse test / isolate *105HT* | USDA germplasm collection (405) | 5.3% | - | Wen et al. 2018 |
|  | *Sclero 10-1* | Satt175 | 61.93cM (15,394,119 a2) | Field inoculation (IA and WI, US) and greenhouse test | PI (66), breeding lines (35), F4:6 (392) | 7.0% | - | Kandel et al. 2018; soybase |
|  | - | ss715596502 | (16,084,952 a2) | Greenhouse test | USDA germplasm collection (474) | - | - | Moellers et al. 2017 |
|  | - | ss715596530 | (16,235,287 a2) | Field inoculation (IA, US) | USDA germplasm collection (474) | - | - | Moellers et al. 2017 |
|  | - | Gm07:33815183 | (33,815,183 a2) | Detached leaf method | China landraces (38), elite cultivars (147) | 4.5-5.9% | Allele T | Sun et al. 2020 |
|  | *Sclero 1-2* | laSU-A226H-1/A226_1 | 71.09cM * | Field test (MI, US) | F3 derived RILs (252) | 9.2% | S19-90 | Kim and Diers 2000 |
|  | - | SAG1048 patent | 84.2cM | - |  | - | - | Sebastian et al. 2010 |
| MLG A2 (Chr. 8) | - | ss715601247 | (2,677,309 a2) | Greenhouse test | USDA germplasm collection (474) | - | - | Moellers et al. 2017 |
|  | *Locus5* | ss715601283 | (2,789,107 a2) | Field test (MI, US), greenhouse test / isolate *105HT* | Improved lines (962) | 5.1% | - | Wen et al. 2018 |
|  | - | - | (3,477,233 a2) | Greenhouse test | CNSGB germplasm (261) | - | Allele G | Zou et al. 2021 |
|  | - | Gm08:3493511 | (3,493,511 a2) | Cut stem method | China accessions (185) | 7-11% | Allele A | Jing et al. 2021 |
|  | - | - | (7,606,596 - 7,650,317 a1) | Greenhouse test | Germplasm and elite lines (101) | 16% | - | Iquira et al. 2015 |
|  | *QTL2, Sclero 2-2; Sclero 3-2; Sclero 5-1; Sclero 6-2* | Satt424 | (10,633,645 a2) | Detached leaf method / isolate *143* | F5 (400) | 4-10% | Corsoy79, Dassel, Wi lliams82 | Arahana et al. 2001 |
|  | - | Gm08:23254864 | (23,254,864 a2) | Cut stem method | China accessions (185) | 8% | Allele G | Jing et al. 2021 |
|  | *Sclero 8-1* | Sat_138 | (41,292,087 a2) | Greenhouse test / isolate *105HT* | F4:5 (155) | 12.1% | PI 194639 | Vuong et al. 2008 |
|  | *Sclero 9-2* | Satt209 | (42,190,891 a2) | Field inoculations (MI, US) / *isolate HT105* | F2:3 (94), F2:4 (94), F2:5 (94) | 12.4% | IA2053 | Guo et al. 2008 |
| MLG K (Chr. 9) | - | ss715604491 | (4,195,294 a2) | Greenhouse test | USDA germplasm collection (474) | - | - | Moellers et al. 2017 |
|  | - | ss715604590 | (4,266,665 a2) | Greenhouse test | USDA germplasm collection (474) | - | - | Moellers et al. 2017 |
|  | - | ss715604731 | (4,409,676 a2) | Greenhouse test | USDA germplasm collection (474) | - | - | Moellers et al. 2017 |
|  | - | ss715604981 | (4,653,871 a2) | Greenhouse test | USDA germplasm collection (474) | - | - | Moellers et al. 2017 |
|  | - | ss715605054 | (4,743,286 a2) | Greenhouse test | USDA germplasm collection (474) | - | - | Moellers et al. 2017 |
|  | - | Gm09:5469571 | (5,469,571 a2) | Detached leaf method | China landraces (38), elite cultivars (147) | 4.8-12.3% | Allele C | Sun et al. 2020 |
|  | - | Gm09:26950566 | (26,950,566 a2) | Cut stem method | China accessions (185) | 7-8% | Allele A | Jing et al. 2021 |
|  | - | ss715603406 | (28,637,128 a2) | Greenhouse test | USDA germplasm collection (474) | - | - | Moellers et al. 2017 |
|  | - | ss715603408 | (28,739,753 a2) | Greenhouse test | USDA germplasm collection (474) | - | - | Moellers et al. 2017 |
|  | - | ss715603485 | (31,230,369 a2) | Greenhouse test | USDA germplasm collection (474) | - | - | Moellers et al. 2017 |
|  | - | ss715603491 | (31,459,532 a2) | Greenhouse test | USDA germplasm collection (474) | - | - | Moellers et al. 2017 |
|  | - | ss715603503 | (31,828,736 a2) | Greenhouse test | USDA germplasm collection (474) | - | - | Moellers et al. 2017 |
|  | - | S9_32,113,409 | (32,113,409 a1) | Greenhouse test / isolate *Jatai* | Brazil breeding lines (275) | 2.3% | - | Wei et al. 2017 |
|  | - | Gm09:33075095 | (33,075,095 a2) | Cut stem method | China accessions (185) | 7-8% | Allele G | Jing et al. 2021 |
|  | *QTL20, Sclero 2-17; Sclero 3-12* | Satt260 | (42,407,962 a2) | Detached leaf method / isolate *143* | F5 (200) | 4-10% | Corsoy79, Dassel | Arahana et al. 2001 |
|  | *QTL21, Sclero 2-18* | Satt588 | (48,619,194 a2) | Detached leaf method / isolate *143* | F5 (100) | 4-10% | Corsoy79 | Arahana et al. 2001 |
|  | *Locus6, Sclero 2-18* | ss715605011 and ss715605026 | (49,559,911 - 49,749,681 a2) | Field test (MI, US), greenhouse test / isolate *105HT* | Improved lines (962) | 5.3-8.2% | - | Wen et al. 2018 |
|  | - | S60222-TB patent | 170.0cM | - |  | - | - | Sebastian et al. 2010 |
|  | *Sclero 1-1; Sclero 1-3; Sclero 1-6* | OW13_900 | 47.3cM * | Field test | F3 derived RILs (252) | 9.6% | S19-90 | Kim and Diers 2000 |
| MLG O (Chr. 10) | - | ss715608353 | (6,005,807 a2) | Greenhouse test | USDA germplasm collection (474) | - | - | Moellers et al. 2017 |
|  | - | rs15929904 | (15,929,904 a1) | Greenhouse test | China core germplasm collection (330) | 8.4-11.1% | Allele C | Zhao et al. 2015 |
|  | - | Gm10:38740659 | (38,740,659 a2) | Cut stem method | China accessions (185) | 8-10% | Allele A | Jing et al. 2021 |
|  | *QTL27, Sclero 2-23; Sclero 3-18; Sclero 4-10; Sclero 5-15; Sclero 6-12* | Satt477 and Satt123 | (40,306,793 - 40,832,257 a2) | Detached leaf method / isolate *143* | F5 (500) | 4-10% | Corsoy79, Dassel, DSR173, S19-90, Vinton81 | Arahana et al. 2001 |
|  | *Locus11* | ss715607488 | (45,331,299 a2) | Field test (MI, US), greenhouse test / isolate *105HT* | USDA germplasm collection (405) | 5.8% | - | Wen et al. 2018 |
|  | *Sclero 10-4* | Satt153 | 106.32cM (45,959,176 a1) | Field inoculation (IA and WI, US) and greenhouse test | PI (66), breeding lines (35), F4:6 (392) | 3.2% | - | Kandel et al. 2018; soybase |
|  | - | SATT243 patent | 157.0cM | - |  | - | - | Sebastian et al. 2010 |
| MLG B1 (Chr. 11) | - | ss715608756 | (104,375 a2) | Greenhouse test | USDA germplasm collection (474) | - | - | Moellers et al. 2017 |
|  | - | ss715611260 | (879,577 a2) | Greenhouse test | USDA germplasm collection (474) | - | - | Moellers et al. 2017 |
|  | - | rs1808219 | (1,808,219 a1) | Greenhouse test | China core germplasm collection (330) | 7.3-9.0% | Allele T | Zhao et al. 2015 |
|  | - | S11_6,493,121 | (6,493,121 a1) | Greenhouse test / isolate *Jatai* | Brazil breeding lines (275) | 2.4-3.7% | - | Wei et al. 2017 |
|  | *Qsp-2* | Sat_156 and Satt251 | 35-36.48cM * (6,906,465 a2) | Greenhouse test | F5:6 (149) | 6.3-8.9% | MapleArrow | Li et al. 2010 |
|  | *QTL4, Sclero 3-3* | Satt197 | (8,898,878 a2) | Detached leaf method / isolate *143* | F5 (100) | 4-10% | Williams82 | Arahana et al. 2001 |
|  | *Qswm11-1* | Sat_095, BARCSOYSSR11_1208 | 124.11-127.86cM (16,478,273 - 28,813,368 a2) | Greenhouse test | F5:10 (128) | 7.7% | - | Zhao et al. 2015 |
|  | - | ss715610499 | (34,528,210 a2) | Field inoculation (IA, US) | USDA germplasm collection (474) | - | - | Moellers et al. 2017 |
|  | - | SAG1032 patent | 65.6cM | - |  | - | - | Sebastian et al. 2010 |
|  | - | SATT597 patent | 68.1cM | - |  | - | - | Sebastian et al. 2010 |
|  | - | SCT_026 patent | 71.6cM | - |  | - | - | Sebastian et al. 2010 |
| MLG H (Chr. 12) | - | Gm12:7079865 | (7,079,865 a2) | Detached leaf method | China landraces (38), elite cultivars (147) | 6.7-7.8% | Allele T | Sun et al. 2020 |
|  | - | ss715612173 | (32,434,240 a2) | Field inoculation (IA, US) | USDA germplasm collection (474) | - | - | Moellers et al. 2017 |
|  | - | ss715612209 | (32,727,465 a2) | Greenhouse test | USDA germplasm collection (474) | - | - | Moellers et al. 2017 |
| MLG F (Chr. 13) | - | rs4244040 | (4,244,040 a1) | Greenhouse test | China core germplasm collection (330) | 8.5-18.8% | Allele T | Zhao et al. 2015 |
|  | - | ss715613722 | (11,498,741 a2) | Field inoculation (IA, US) | USDA germplasm collection (474) | - | - | Moellers et al. 2017 |
|  | *Sclero 9-5* | AW186493-Satt149 | 18.12-21.04cM* (16,855,019 a2) | Field inoculation (MI, US) and greenhouse inoculation / *isolate HT105* | F2:3 (94), F2:4 (94), F2:5 (94) | 9.6% | PI 391589B | Guo et al. 2008 |
|  | - | ss715615422 | (18,644,670 a2) | Field inoculation (IA, US) | USDA germplasm collection (474) | - | - | Moellers et al. 2017 |
|  | - | ss715614675 | (28,642,102 a2) | Greenhouse test | USDA germplasm collection (474) | - | - | Moellers et al. 2017 |
|  | *QTL14, Sclero 2-12; Sclero 3-9; Sclero 4-4; Sclero 5-6; Sclero 6-5* | Satt114 | (28,912,864 a2) | Detached leaf method / isolate *143* | F5 (500) | 4-10% | Corsoy79, Dassel, DSR173, S19-90, Vinton81 | Arahana et al. 2001 |
|  | *QTL15, Sclero 5-7; Sclero 6-6* | Satt510 and Satt335 | (31,802,559 - 32,721,481 a2) | Detached leaf method / isolate *143* | F5 (200) | 4-10% | S19-90, Vinton81 | Arahana et al. 2001 |
|  | - | ss715616351 | (42,324,129 a2) | Field inoculation (IA, US) | USDA germplasm collection (474) | - | - | Moellers et al. 2017 |
|  | - | Gm13:44279860 | (44,279,860 a2) | Cut stem method | China accessions (185) | 7-8% | Allele T | Jing et al. 2021 |
|  | - | S60817-TB patent | 30.0cM | - |  | - | - | Sebastian et al. 2010 |
| MLG B2 (Chr. 14) | *Sclero 10-6* | Sat_342 | 15.5cM (2,956,930 a2) | Field inoculation (IA and WI, US) and greenhouse test | PI (66), breeding lines (35), F4:6 (392) | 1.8% | - | Kandel et al. 2018; soybase |
|  | - | ss715618320 | (3,198,128 a2) | Field inoculation (IA, US) | USDA germplasm collection (474) | - | - | Moellers et al. 2017 |
|  | - | ss715618415 | (3,419,976 a2) | Field inoculation (IA, US) | USDA germplasm collection (474) | - | - | Moellers et al. 2017 |
|  | *Locus12, Sclero 8-2* | ss715618590, ss715618599, ss715618604 | (3,852,549 - 3,885,274 a2) | Field test (MI, US), greenhouse test / isolate *105HT* | USDA germplasm collection (405) | 5.9-6.8% | - | Wen et al. 2018 |
|  | *Sclero 8-2* | Satt126 | (5,020,200 a2) | Greenhouse test / isolate *105HT* | F4:5 (155) | 11.2% | Merit | Vuong et al. 2008 |
|  | - | Gm14:12784029 | (12,784,029 a2) | Cut stem method | China accessions (185) | 7-8% | Allele T | Jing et al. 2021 |
|  | *QTL5, Sclero 2-4; Sclero 5-2* | Satt438, Satt070 | (17,406,101 a2) | Detached leaf method / isolate *143* | F5 (200) | 4-10% | Williams82, S19-90 | Arahana et al. 2001 |
|  | - | Satt556, P1694 (Patent) | (38,859,467 a2) | greenhouse | - | 12.0% | P^R^ | Han et al. 2007 |
|  | - | ss715618815 | (42,618,496 a2) | Greenhouse test | USDA germplasm collection (474) | - | - | Moellers et al. 2017 |
|  | - | ss715619067 | (45,176,960 a2) | Greenhouse test | USDA germplasm collection (474) | - | - | Moellers et al. 2017 |
|  | - | ss715619085 | (45,287,060 a2) | Greenhouse test | USDA germplasm collection (474) | - | - | Moellers et al. 2017 |
| MLG E (Chr. 15) | *Sclero 9-3* | Satt212-Satt720 | (4,154,674 - 5,240,509 a2) | Field inoculation (MI, US) and greenhouse inoculation / *isolate HT105* | F2:3 (94), F2:4 (94), F2:5 (94) | 9.9% | PI 391589B | Guo et al. 2008 |
|  | - | Satt212-Satt268 | (5,240,509 - 22,969,031 a2) | Infested oat seed cotyledon assay and cut stem assay | BC1F4:5 (230) | 6.0% | PI 391589A | Guo et al. 2008 |
|  | - | Gm15:12149616 | (12,149,616 a2) | Cut stem method | China accessions (185) | 7-9% | Allele T | Jing et al. 2021 |
|  | *Locus13* | ss715620418, ss715620421 | (12,264,951 - 12,278,417 a2) | Field test (MI, US), greenhouse test / isolate *105HT* | USDA germplasm collection (405) | 5.2-5.4% | - | Wen et al. 2018 |
|  | *qLLS15-1* | Block3520-Block3519 | 213.60cM (12,677,751-12,778,261 a2) | Greenhouse test | F5:20 (149) | 13.2% | Maple Arrow | Zou et al. 2021 |
|  | *Sclero 9-4* | Satt185-Satt263 | (28,489,658 - 30,238,139 a2) | Field inoculation (MI, US) and greenhouse inoculation / *isolate HT105* | F2:3 (94), F2:4 (94), F2:5 (94) | 15.7% | IA2053 | Guo et al. 2008 |
|  | - | ss715621805 | (40,083,805 a2) | Field inoculation (IA, US) | USDA germplasm collection (474) | - | - | Moellers et al. 2017 |
|  | - | BARCSOYSSR_15_1382 | (47,656,624 a2) | Cut petiole technique / isolate *25* | F7 derived RILs (109) | - | W04-1002 | McCaghey et al. 2017 |
|  | - | Gm15:47922300 | (47,922,300 a2) | Detached leaf method | China landraces (38), elite cultivars (147) | 3.5-5.9% | Allele A | Sun et al. 2020 |
|  | - | BARCSOYSSR_15_1400 | (48,070,447 a2) | Cut petiole technique / isolate *25* | F7 derived RILs (109) | - | W04-571 | McCaghey et al. 2017 |
|  | - | PHP10118C, Satt231 (Patent) | (51,294,894 a2) | greenhouse | - | 10.0% | P^S^ | Han et al. 2007 |
|  | *QTL13, Sclero 2-11; Sclero 3-8; Sclero 4-3; Sclero 5-5* | OP_m12, OP_M12b | 22.84cM * | Detached leaf method / isolate *143* | F5 (400) | 4-10% | Corsoy79, Dassel, DSR173, Williams82 | Arahana et al. 2001 |
| MLG J (Chr. 16) | - | ss715623502 | (1,576,261 a2) | Field inoculation (IA, US) | USDA germplasm collection (474) | - | - | Moellers et al. 2017 |
|  | *QTL18, Sclero 2-15* | Satt451 | (1,683,957 a2) | Detached leaf method / isolate *143* | F5 (100) | 4-10% | Williams82 | Arahana et al. 2001 |
|  | - | S16_3,111,366 | (3,111,366 a1) | Greenhouse test / isolate *Jatai* | Brazil breeding lines (275) | 2.1% | - | Wei et al. 2017 |
|  | - | BARCSOYSSR_16_0290 | (4,716,256 a2) | Cut petiole technique / isolate *25* | F7 derived RILs (250) | - | W04-1002 | McCaghey et al. 2017 |
|  | - | ss715625404 | (7,244,545 a2) | Field inoculation (IA, US) | USDA germplasm collection (474) | - | - | Moellers et al. 2017 |
|  | - | Gm16:21589672 | (21,589,672 a2) | Detached leaf method | China landraces (38), elite cultivars (147) | 4.7-5.0% | Allele T | Sun et al. 2020 |
|  | *Locus14* | ss715624027, ss715624030, ss715624031 | (29,081,835 - 29,095,909 a2) | Field test (MI, US), greenhouse test / isolate *105HT* | USDA germplasm collection (405) | 5.2-7.7% | - | Wen et al. 2018 |
|  | *Locus7* | ss715624465 and ss715624900 | (31,667,215 - 31,915,854 a2) | Field test (MI, US), greenhouse test / isolate *105HT* | Improved lines (962) | 5.2-6.1% | - | Wen et al. 2018 |
|  | - | - | (34,207,580 a2) | Greenhouse test | CNSGB germplasm (261) | - | Allele A | Zou et al. 2021 |
|  | - | ss715624738 | (35,186,332 a2) | Greenhouse test | USDA germplasm collection (474) | - | - | Moellers et al. 2017 |
|  | - | P1047, A724_1 (Patent) | 84.89cM * | greenhouse | - | 6% | - | Han et al. 2007 |
| MLG D2 (Chr. 17) | *QTL9, Sclero 2-8* | Satt458 | (5,788,551 a2) | Detached leaf method / isolate *143* | F5 (100) | 4-10% | Corsoy79 | Arahana et al. 2001 |
|  | *Sclero 10-7* | Satt135 | 25.48cM (5,891,979 a2) | Field inoculation (IA and WI, US) and greenhouse test | PI (66), breeding lines (35), F4:6 (392) | 11.3% | - | Kandel et al. 2018; soybase |
|  | - | ss715628199 | (7,740,564 a2) | Greenhouse test | USDA germplasm collection (474) | - | - | Moellers et al. 2017 |
|  | - | BARCSOYSSR_17_0460 | (7,841,443 a2) | Cut petiole technique / isolate *25* | F7 derived RILs (224) | - | W04-1002 | McCaghey et al. 2017 |
|  | - | BARCSOYSSR_17_0471 | (8,064,099 a2) | Cut petiole technique / isolate *25* | F7 derived RILs (224) | - | W04-1002 | McCaghey et al. 2017 |
|  | - | BARCSOYSSR_17_0476 | (8,281,145 a2) | Cut petiole technique / isolate *25* | F7 derived RILs (224) | - | W04-1002 | McCaghey et al. 2017 |
|  | - | BARCSOYSSR_17_0500 | (8,706,906 a2) | Cut petiole technique / isolate *25* | F7 derived RILs (224) | - | W04-1002 | McCaghey et al. 2017 |
|  | - | BARCSOYSSR_17_0507 (Satt154) | (8,799,234 a2) | Cut petiole technique / isolate *25* | F7 derived RILs (224) | - | W04-1002 | McCaghey et al. 2017 |
|  | - | PHP8701R, Satt311 (Patent) | (18,065,453 a2) | greenhouse | - | 14.0% | P^R^ | Han et al. 2007 |
|  | *QTL11, Sclero 2-9; Sclero 4-2* | Satt543 and Satt301 | (30,341,859 - 36,718,722 a2) | Detached leaf method / isolate *143* | F5 (200) | 4-10% | Corsoy79, DSR173 | Arahana et al. 2001 |
|  | *Sclero 10-9* | Satt186 | 92.22cM (38,763,354 a2) | Field inoculation (IA and WI, US) and greenhouse test | PI (66), breeding lines (35), F4:6 (392) | 14.2% | - | Kandel et al. 2018; soybase |
|  | *QTL12, Sclero 2-10; Sclero 3-7; Sclero 6-4* | Satt256 | (40,525,673 a2) | Detached leaf method / isolate *143* | F5 (300) | 4-10% | Corsoy79, Dassel, Vinton81 | Arahana et al. 2001 |
| MLG G (Chr. 18) | - | Gm18:949979 | (949,979 a2) | Detached leaf method | China landraces (38), elite cultivars (147) | 7.6-8.4% | Allele G | Sun et al. 2020 |
|  | - | BARCSOYSSR_18_0105 | (1,808,801 a2) | Cut petiole technique / isolate *25* | F7 derived RILs (224) | - | L84-5873 | McCaghey et al. 2017 |
|  | - | ss715630238 | (3,184,755 a2) | Greenhouse test | USDA germplasm collection (474) | - | - | Moellers et al. 2017 |
|  | - | ss715630264 | (3,253,599 a2) | Greenhouse test | USDA germplasm collection (474) | - | - | Moellers et al. 2017 |
|  | - | Gm18:6720660 | (6,720,660 a2) | Detached leaf method | China landraces (38), elite cultivars (147) | 5.2-6.4% | Allele C | Sun et al. 2020 |
|  | - | ss715632705 | (7,929,040 a2) | Greenhouse test | USDA germplasm collection (474) | - | - | Moellers et al. 2017 |
|  | - | S18_14,327,556, S18_14,327,607, S18_14,517,407, S18_14,517,362, S18_14,282,760, S18_14,282,812, S18_14,282,806, S18_14,324,249, S18_14,324,245, S18_14,334,212, | (14,282,760 - 14,517,407 a1) | Greenhouse test / isolate *Jatai* | Brazil breeding lines (275) | 3.2-4.4% | - | Wei et al. 2017 |
|  | *Locus8* | ss715630705 | (43,030,373 a2) | Field test (MI, US), greenhouse test / isolate *105HT* | Improved lines (962) | 6.0% | - | Wen et al. 2018 |
|  | - | ss715631537 | (49,816,209 a2) | Greenhouse test | USDA germplasm collection (474) | - | - | Moellers et al. 2017 |
|  | *QTL17, Sclero 2-14; Sclero 3-10; Sclero 4-6; Sclero 5-9; Sclero 6-7* | Satt472 and Satt191 | (53,866,536 - 54,450,956 a2) | Detached leaf method / isolate *143* | F5 (500) | 4-10% | Corsoy79, Dassel, DSR173, Williams82 | Arahana et al. 2001 |
|  | - | S60239-TB patent | 0.0cM | - |  | - | - | Sebastian et al. 2010 |
|  | - | P10646A-1 patent | 2.0cM | - |  | - | - | Sebastian et al. 2010 |
|  | - | P7659A-2 patent | 4.0cM | - |  | - | - | Sebastian et al. 2010 |
|  | *QTL16, Sclero 2-13; Sclero 4-5; Sclero 5-8* | Satt394 | 43.38cM * | Detached leaf method / isolate *143* | F5 (300) | 4-10% | Corsoy79, S19-90, Williams82 | Arahana et al. 2001 |
|  | - | SATT570 patent | 7.7cM | - |  | - | - | Sebastian et al. 2010 |
|  | - | SATT356 patent | 8.5cM | - |  | - | - | Sebastian et al. 2010 |
| MLG L (Chr. 19) | *QTL22, Sclero 2-19; Sclero 3-13; Sclero 4-8; Sclero 5-11; Sclero 6-8* | Satt143 and Satt_134 | 30.19 - 112.83cM | Detached leaf method / isolate *143* | F5 (500) | 4-10% | Corsoy79, Dassel, DSR173, Williams82, Vinton81 | Arahana et al. 2001 |
|  | *Locus15* | ss715636086 | (579,512 a2) | Field test (MI, US), greenhouse test / isolate *105HT* | USDA germplasm collection (405) | 5.2% | - | Wen et al. 2018 |
|  | - | S19_1,289,850 | (1,289,850 a1) | Greenhouse test / isolate *Jatai* | Brazil breeding lines (275) | 3.3% | - | Wei et al. 2017 |
|  | *Sclero 8-4* | Satt182 | (2,059,590 a2) | Greenhouse test / isolate *105HT* | F4:5 (155) | 5.5% | PI 194639 | Vuong et al. 2008 |
|  | *Locus16* | ss715634194 | (3,498,043 a2) | Field test (MI, US), greenhouse test / isolate *105HT* | USDA germplasm collection (405) | 5.1% | - | Wen et al. 2018 |
|  | - | Gm19:3989466 | (3,989,466 a2) | Cut stem method | China accessions (185) | 7-9% | Allele A | Jing et al. 2021 |
|  | - | ss715633818 | (32,622,933 a2) | Greenhouse test | USDA germplasm collection (474) | - | - | Moellers et al. 2017 |
|  | - | Gm19:36122367 | (36,122,367 a2) | Detached leaf method | China landraces (38), elite cultivars (147) | 4.1-4.8% | Allele A | Sun et al. 2020 |
|  | - | ss715634536 | (37,017,795 a2) | Greenhouse test | USDA germplasm collection (474) | - | - | Moellers et al. 2017 |
|  | - | BARCSOYSSR_19_0908 | (37,423,905 a2) | Cut petiole technique / isolate *25* | F7 derived RILs (250) | - | W04-1002 | McCaghey et al. 2017 |
|  | - | ss715634826 | (39,505,778 a2) | Greenhouse test | USDA germplasm collection (474) | - | - | Moellers et al. 2017 |
|  | - | - | (39,698,515 a1) | Greenhouse test / strain *NB-5* | Breeding lines (130) | 6.3% | - | Bastien et al. 2014 |
|  | *QTL23, Sclero 2-20; Sclero 3-14; Sclero 5-12; Sclero 6-9* | Satt481 | (40,319,984 a2) | Detached leaf method / isolate *143* | F5 (300) | 4-10% | Dassel, S19-90, Vinton81, Williams82 | Arahana et al. 2001 |
|  | - | BARCSOYSSR_19_1314 | (44,933,476 a2) | Cut petiole technique / isolate *25* | F7 derived RILs (117) | - | W04-1002 | McCaghey et al. 2017 |
|  | - | BARCSOYSSR_19_1367 | (45,777,597 a2) | Cut petiole technique / isolate *25* | F7 derived RILs (117) | - | W04-1002 | McCaghey et al. 2017 |
|  | - | BARCSOYSSR_19_1424 (Satt166) | (47,118,641 a2) | Cut petiole technique / isolate *25* | F7 derived RILs (117) | - | W04-1002 | McCaghey et al. 2017 |
|  | - | ss715635935 | (49,462,811 a2) | Field inoculation (IA, US) | USDA germplasm collection (474) | - | - | Moellers et al. 2017 |
|  | - | Gm19:49923093 | (49,923,093 a2) | Cut stem method | China accessions (185) | 7-8% | Allele T | Jing et al. 2021 |
|  | - | SATT166 patent | 77.1cM | - |  | - | - | Sebastian et al. 2010 |
|  | - | SATT448 patent | 78.0cM | - |  | - | - | Sebastian et al. 2010 |
| MLG I (Chr. 20) | *Sclero 10-11* | Satt571 | 14.97cM (1,287,393 a2) | Field inoculation (IA and WI, US) and greenhouse test | PI (66), breeding lines (35), F4:6 (392) | 2.3% | - | Kandel et al. 2018 |
|  | *Sclero 7-3* | Satt700, Satt614 | (3,903,416 - 25,498,552 a2) | Field inoculation (QC, Canada) / strain *NB-5* | F4 derived RILs (180) | 4-12% | Maple Donovan | Huynh et al. 2010 |
|  | - | - | (33,511,401 a1) | Greenhouse test | Germplasm and elite lines (101) | 15% | - | Iquira et al. 2015 |
|  | - | Gm20:33803317 | (33,803,317 a2) | Cut stem method | China accessions (185) | 8-11% | Allele T | Jing et al. 2021 |
|  | - | - | (34,900,979 a2) | Greenhouse test | CNSGB germplasm (261) | - | Allele T | Zou et al. 2021 |
|  | - | ss715637528 | (35,151,397 a2) | Field inoculation (IA, US) | USDA germplasm collection (474) | - | - | Moellers et al. 2017 |
|  | - | ss715638266 | (42,297,577 a2) | Greenhouse test | USDA germplasm collection (474) | - | - | Moellers et al. 2017 |
|  | - | ss715638269 | (42,350,470 a2) | Greenhouse test | USDA germplasm collection (474) | - | - | Moellers et al. 2017 |
|  | - | - | (44,170,264 a2) | Greenhouse test | CNSGB germplasm (261) | - | Allele G | Zou et al. 2021 |
|  | - | - | (50,557,054 a1) | Greenhouse test / strain *NB-5* | Breeding lines (130) | 7.2% | - | Bastien et al. 2014 |

^a^: Marker position (bp) based on the *Glycine max* genome assembly version *Gmax1.01* (a1), or *Gmax2.0* (a2), only starting position is shown for SSR markers.

^b^: Phenotypic variations explained by the molecular markers.

*: GmComposite2003 genetic position (www.soybase.org)
