# Supplementary material for: Breeding for disease resistance in soybean: a global perspective
Source: Theor Appl Genet. 2022 Jul 5;135(11):3773–872. doi: 10.1007/s00122-022-04101-3 (PMC9729162; doi:10.1007/s00122-022-04101-3)
Supplement: Supplementary file 2 — Supplementary file2 (DOCX 51 kb) [file 122_2022_4101_MOESM2_ESM.docx]

**Supplementary Table 1** Additional soybean loci conferring resistance to Phytophthora root and stem rot (caused by *P. sojae*)

| **MLG (Chr.)** | **Locus name** | **Tightly linked / flanking markers** | **Marker position**  **cM (bp) ^a^** | **Testing methods / Resistance spectrum** | **Population type (size)** | **PVE ^b^** | **Candidate gene** | **Donor source / allele** | **Reference** |
| --- | --- | --- | --- | --- | --- | --- | --- | --- | --- |
| MLG D1a (Chr. 1) | *-* | Gm01_44414453 | (44,414,453 a1) | Hypocotyl inoculation / HeN08-35 | Germplasm (337) | 6.1-11.2% | Glyma01g32800 and Glyma01g32855 | - | Niu et al. 2018 |
|  | *-* | BARC-044479-08708 | 71cM (50,885,379 a1) | Tray test / isolate *C2S1* | F7:8 (305) | 4.6% | - | PI 398841 | Lee et al. 2013a |
|  | *-* | - | 50-51cM | Tray test and layer test / isolates *C.2.S.1, OH25, OH7, 1.S.1.1, OH30* | F7:8 (305) | 6.9% | - | PI 398841 | Lee et al. 2014 |
|  | *-* | - | 58-60cM | Tray test and layer test / isolates *C.2.S.1, OH25, OH7, 1.S.1.1, OH30* | F7:8 (305) | 7.7% | - | PI 398841 | Lee et al. 2014 |
|  | *-* | - | 60-62cM | Tray test and layer test / isolates *C.2.S.1, OH25, OH7, 1.S.1.1, OH30* | F7:8 (305) | 3.5% | - | PI 398841 | Lee et al. 2014 |
|  | *-* | - | 63-65cM | Tray test and layer test / isolates *C.2.S.1, OH25, OH7, 1.S.1.1, OH30* | F7:8 (305) | 5.6% | - | PI 398841 | Lee et al. 2014 |
| MLG D1b (Chr. 2) | *qHC2-1* | ss715584058 | (8,923,676 a2) | Hypocotyl inoculation / isolate *PT2004 C2.S1* | Germplasm (460) | - | - | - | Van et al. 2020 |
|  | - | Satt634-133 | (11,778,505 a2) | Slant board assay / race *2* | China mini core collection (175) | 8.1% | - | ZDD01629 | Sun et al. 2014 |
|  | - | Satt634-149 | (11,778,505 a2) | Slant board assay / race *2* | China mini core collection (175) | 7.0% | - | ZDD03842 | Sun et al. 2014 |
|  | *-* | Satt266 (BARCSOYSSR_02_0727) and Satt579 (BARCSOYSSR_02_0855) | (14,288,241 – 19,688,108 a2) | Slant board test / isolate OH25 | F4:6 (64) | 15.9% | - | - | Burnham et al. 2003b |
|  | *OH-02-1* | ss715582345 | (38,935,468 - 39,002,760 a2) | Layer test / isolate *OH.121* | Germplasm (478) | 2.7% ΔSW^c^ | - | - | Rolling et al. 2020 |
|  | *OH-02-2* | ss715582359 | (39,090,899 - 39,126,047 a2) | Layer test / isolate *OH.121* | Germplasm (478) | 6.4% IRRS^d^ | - | - | Rolling et al. 2020 |
|  | *C2-02-1* | ss715582994 | (43,367,206 - 43,440,684 a2) | Layer test / isolate *C2.S1* | Germplasm (495) | 2.8% IRW^e^ | - | - | Rolling et al. 2020 |
|  | *QFP1/QGP3* | OPL18_800_ and Satt274 (BARCSOYSSR_02_1663) | (45,267,040 a2) | Field inoculation and greenhouse test (China and Canada) | F2:7 (112) | 9.6-21.6% | - | - | Han et al. 2008 |
|  | *-* | BARC-019805-04379 and BARC-042881-08448 | (47,449,589 - 48,163,979 a2) | Rice-based method / isolates *PT2004C2.S1, 1005-2.9, R7-2a* | F5:7 (232), F5:7 (277) | 12.1% CDRW^f^, 13.3% %DRL^g^, 14.6% %DSA^h^ | - | AR2 | Abeysekara et al. 2016 |
|  | *-* | BARC-906743-01012 and BARC-019805-04379 | (48,163,979 - 48,470,807 a2) | Rice-based method / isolates *PT2004C2.S1, 1005-2.9, R7-2a* | F5:7 (232), F5:7 (277) | 5.6% CDSW^i^ | - | AR2 | Abeysekara et al. 2016 |
| MLG N (Chr. 3) | *C2-03-1* | ss715586961 | (786,873 - 821,859 a2) | Layer test / isolate *C2.S1* | Germplasm (495) | 6.9% IRRS | - | - | Rolling et al. 2020 |
|  | *C2-03-2* | ss715586992 | (821,859 - 875,151 a2) | Layer test / isolate *C2.S1* | Germplasm (495) | 7.0% ΔRW^j^ | - | - | Rolling et al. 2020 |
|  | *qHC3-1* | ss715585067 | (3,036,784 a2) | Hypocotyl inoculation / isolate *PT2004 C2.S1* | Germplasm (460) | - | - | - | Van et al. 2020 |
|  | *-* | Gm03_3225968 | (3,254,065 a2) | Layer test / isolate ? (vir 1d, 2, 3b, 3c, 4, 5, 6, 7) | Cultivars (169) | 11.2-21.1% | Glyma03g03480 | - | Ludke et al. 2019 |
|  | *Rprr-3-2* | - | (4,410,714 a2) | Hypocotyl inoculation / race *1* | Germplasm and cultivars (225) | 31.8% | - | Allele A | Zhao et al. 2020 |
|  | *-* | Gm03_4782127 | (4,591,270 a2) | Layer test / isolate ? (vir 1d, 2, 3b, 3c, 4, 5, 6, 7) | Cultivars (169) | 12.2% | - | - | Ludke et al. 2019 |
|  | *-* | Gm03_5106459 | (5,009,334 a2) | Layer test / isolate ? (vir 1d, 2, 3b, 3c, 4, 5, 6, 7) | Cultivars (169) | 14.5% | - | - | Ludke et al. 2019 |
|  | *-* | Q-03-0059953 | (5,147,782 a1) | Hypocotyl inoculation / isolate *Pm28* | China mini core germplasm (224) | 3.4-5.7% | Glyma03g04960 | - | Huang et al. 2016 |
|  | *-* | Gm03_5217414 | (5,120,291 a2) | Layer test / isolate ? (vir 1d, 2, 3b, 3c, 4, 5, 6, 7) | Cultivars (169) | 13.9-16.8% | Glyma03g04990 | - | Ludke et al. 2019 |
|  | *-* | Gm03_5341695 | (5,244,122 a2) | Layer test / isolate ? (vir 1d, 2, 3b, 3c, 4, 5, 6, 7) | Cultivars (169) | 14.7% | Glyma03g05070 | - | Ludke et al. 2019 |
|  | *OH-03-1* | ss715586915 | (6,074,620 - 6,378,977 a2) | Layer test / isolate *OH.121* | Germplasm (478) | 4.8% IRW, 4.7% ISW | - | - | Rolling et al. 2020 |
|  | *OH-03-2* | ss715586985 | (7,872,384 - 8,252,615 a2) | Layer test / isolate *OH.121* | Germplasm (478) | 8.5% IRRS | - | - | Rolling et al. 2020 |
|  | *-* | BARC-050433-09624 and BARC-010179-00543 | (35,023,448 - 36,005,566 a2) | Rice-based method / isolates *PT2004C2.S1, 1005-2.9, R7-2a* | F5:7 (232), F5:7 (277) | 6.7% %DRL, 6.9% %DSA | - | PI 399036 | Abeysekara et al. 2016 |
|  | *-* | Q-03-0266907 | (36,634,361 a1) | Hypocotyl inoculation / isolate *P7063* | China mini core germplasm (224) | 3.2-10.4% | Glyma03g28660 | - | Huang et al. 2016 |
|  | *Qprr3-1* | Chr03-41803925 and Chr03-41822143 | (41,803,925-41,822,143 a2) | Hypocotyl inoculation / race *1* | F5:15 (109) | 5.9% | Glyma.03G033800 and Glyma.03G033700 | DongongL-28 | Zhao et al. 2020 |
|  | *OH-03-3* | ss715586306 | (42,506,684 - 42,517,511 a2) | Layer test / isolate *OH.121* | Germplasm (478) | 8.6% ΔSW | - | - | Rolling et al. 2020 |
|  | *QTL-3a* | BARC-028645-05979 to BARCSOYSSR_03_0317 | 21-27cM | Tray test / isolate *OH25* | F7:8 (157) | 3.6% | - | PI 407861A | Lee et al. 2013b |
|  | *-* | - | 28-31cM | Tray test and layer test / isolates *C.2.S.1, OH25, OH7, 1.S.1.1, OH30* | F7:8 (305) | 1.4-2.9% | - | PI 398841 | Lee et al. 2014 |
|  | *QTL-3b* | - | 63-73cM | Tray test and layer test / isolates *C.2.S.1, OH25, OH7, 1.S.1.1, OH30* | F7:8 (157) | 5.6% | - | PI 407861A | Lee et al. 2014 |
|  | *QTL-3b* | Sat_091 to Sat_125 | 65-85cM | Tray test / isolate *OH25* | F7:8 (157) | 4.6% | - | PI 407861A | Lee et al. 2013b |
|  | *QTL-3b* | - | 74-77cM | Tray test and layer test / isolates *C.2.S.1, OH25, OH7, 1.S.1.1, OH30* | F7:8 (157) | 4.4-7.4% | - | PI 407861A | Lee et al. 2014 |
| MLG C1 (Chr. 4) | *C2-04-1* | ss715589155 | (6,514,173 - 6,682,383 a2) | Layer test / isolate *C2.S1* | Germplasm (495) | 5.5% IRW | - | - | Rolling et al. 2020 |
|  | *-* | Map-0715 | (46,749,591 a1) | Hypocotyl inoculation / isolate *Pm31* | China mini core germplasm (224) | 2.4-7.4% | Glyma04g40800 | - | Huang et al. 2016 |
|  | *QTL-4b* | BARC-024445-04886 to BARC-061079-17031 | 44-54cM | Tray test / isolate *OH25* | F7:8 (157) | 2.5% | - | OX20-8 | Lee et al. 2013b |
|  | *QTL-4a* | BARC-038359-10052 to BARC-054289-12451 | 5-16cM | Tray test / isolate *OH25* | F7:8 (157) | 2.4% | - | OX20-8 | Lee et al. 2013b |
| MLG A1 (Chr. 5) | *qHC5-1* | ss715590933, ss715590944, ss715590958 | (33,948,991 / 34,007,017 / 34,066,088 a2) | Hypocotyl inoculation / isolate *PT2004 C2.S1* | Germplasm (460) | - | - | - | Van et al. 2020 |
|  | *OH-05-1* | ss715591382 | (36,972,839 - 37,035,513 a2) | Layer test / isolate *OH.121* | Germplasm (478) | 7.4% IPH^k^ | - | - | Rolling et al. 2020 |
|  | *C2-05-1* | ss715591632 | (41,780,982 - 42,090,709 a2) | Layer test / isolate *C2.S1* | Germplasm (495) | 5.7% IRW | - | - | Rolling et al. 2020 |
| MLG C2 (Chr. 6) | *-* | BARC-014527-01571 | (644,565 a1) | Hypocotyl inoculation / isolate *PNJ1* | China mini core germplasm (224) | 7.2-7.7% | Glyma06g01080 | - | Huang et al. 2016 |
|  | *QPRR-8* | Satt460 (BARCSOYSSR_06_1456) and Satt307 (BARCSOYSSR_06_1581) | (44,049,891 - 46,820,673 a2) | Field inoculation and greenhouse test (China and Canada) | F2:6 (140) | 4.2-7.8% | - | - | Li et al. 2010 |
|  | *qHM6-1* | ss715594898 | (48,466,050 a2) | Hypocotyl inoculation / isolates *PT2004 C2.S1, R7-2a, 1005-2.9* | Germplasm (448) | - | - | - | Van et al. 2020 |
|  | *OH-06-1* | ss715595238 | (50,603,738 - 50,852,296 a2) | Layer test / isolate *OH.121* | Germplasm (478) | 7.8% IPH, 8.7% IRRS | - | - | Rolling et al. 2020 |
| MLG M (Chr. 7) | *qHO7-1* | ss715598441 | (6,147,344 a2) | Hypocotyl inoculation / isolate *OH.12108.6.3 (OH.121)* | Germplasm (429) | - | - | - | Van et al. 2020 |
|  | *Rprr-7-1* | - | (40,332,063 a2) | Hypocotyl inoculation / race *1* | Germplasm and cultivars (225) | - | - | Allele T | Zhao et al. 2020 |
| MLG A2 (Chr. 8) | *qHO8-1* | ss715599604 | (1,476,014 a2) | Hypocotyl inoculation / isolate *OH.12108.6.3 (OH.121)* | Germplasm (429) | - | - | - | Van et al. 2020 |
|  | *C2-08-1* | ss715602597 | (5,472,166 - 5,709,053 a2) | Layer test / isolate *C2.S1* | Germplasm (495) | 2.8% IPH | - | - | Rolling et al. 2020 |
|  | *qHM8-1* | ss715602853 | (9,332,121 a2) | Hypocotyl inoculation / isolates *PT2004 C2.S1, R7-2a, 1005-2.9* | *G. soja* (520) | - | - | - | Van et al. 2020 |
|  | *C2-08-2* | ss715602910 | (9,877,098 - 9,898,176 a2) | Layer test / isolate *C2.S1* | Germplasm (495) | 1.2% IRRS | - | - | Rolling et al. 2020 |
|  | *QPRR-4* | Satt233 (BARCSOYSSR_08_0960) and Satt437 | (17,232,172 a2) | Field inoculation and greenhouse test (China and Canada) | F2:6 (140) | 5.0-17.0% | - | - | Li et al. 2010 |
|  | *C2-08-3* | ss715600593 | (20,295,654 - 20,975,559 a2) | Layer test / isolate *C2.S1* | Germplasm (495) | 3.2% IPH | - | - | Rolling et al. 2020 |
|  | *QTL-8* | BARC-051883-11286 to BARC-042715-08379 | 102-114cM | Tray test / isolate *OH25* | F7:8 (157) | 7.2% | - | PI 407861A | Lee et al. 2013b |
|  | *-* | - | 44-48cM | Tray test and layer test / isolates *C.2.S.1, OH25, OH7, 1.S.1.1, OH30* | F7:8 (305) | 2.0-10.5% | - | PI 398297 | Lee et al. 2014 |
| MLG K (Chr. 9) | *-* | Map-1630 | (3,157,784 a1) | Hypocotyl inoculation / isolate *Pm31* | China mini core germplasm (224) | 6.8-9.1% | Glyma09g04310 | - | Huang et al. 2016 |
|  | *qHO9-1* | ss715605368 | (6,936,878 a2) | Hypocotyl inoculation / isolate *OH.12108.6.3 (OH.121)* | Germplasm (429) | - | - | - | Van et al. 2020 |
|  |  | BARC_2.0_Gm09_15487393 | (15,487,393 a2) | Tray test / isolates *PT2004C2.S1, 1.S.1.1, OH25* | F9:11 (316) | 4.5-7.4% | - | Conrad | Stasko et al. 2016 |
|  | *-* | BARC-055533-13402 and BARC-007999-00186 | (38,300,154 – 38,383,740 a1) | Rice-based method / isolates *PT2004C2.S1, 1005-2.9, R7-2a* | F5:7 (232), F5:7 (277) | 21% CDSW | - | PI 399036 | Abeysekara et al. 2016 |
|  | *-* | BARC-017625-02635 and BARC-055533-13402 | (39,718,681 - 40,901,351 a2) | Rice-based method / isolates *PT2004C2.S1, 1005-2.9, R7-2a* | F5:7 (232), F5:7 (277) | 29.9% CDRW | - | PI 399036 | Abeysekara et al. 2016 |
| MLG O (Chr. 10) | *OH-10-1* | ss715605487 | (998,272 - 1,022,198 a2) | Layer test / isolate *OH.121* | Germplasm (478) | 4.2% IRW | - | - | Rolling et al. 2020 |
|  | *QTL-10* | BARC-060257-16508 to BARC-015925-02017 | 70-100cM | Tray test / isolate *OH25* | F7:8 (157) | 4.6% | - | PI 407861A | Lee et al. 2013b |
| MLG B1 (Chr. 11) | *OH-11-1* | ss715610747 | (4,563,815 - 4,586,936 a2) | Layer test / isolate *OH.121* | Germplasm (478) | 8.9% IRW | - | - | Rolling et al. 2020 |
|  | *OH-11-2* | ss715610923 | (5,898,743 - 5,962,759 a2) | Layer test / isolate *OH.121* | Germplasm (478) | 12.1% ΔSW | - | - | Rolling et al. 2020 |
|  | *-* | Map-1995 | (7,904,934 a1) | Hypocotyl inoculation / isolate *Pmg* | China mini core germplasm (224) | 4.7-6.2% | Glyma11g11100 | - | Huang et al. 2016 |
|  | *C2-11-1* | ss715609313 | (26,075,475 - 26,169,302 a2) | Layer test / isolate *C2.S1* | Germplasm (495) | 3.1% ΔRW | - | - | Rolling et al. 2020 |
|  | *QPRR-5* | Satt453 (BARCSOYSSR_11_1468) and Satt484 | (34,173,104 a2) | Field inoculation and greenhouse test (China and Canada) | F2:6 (140) | 5.2-14.8% | - | - | Li et al. 2010 |
| MLG H (Chr. 12) | *OH-12-1* | ss715611695 | (1,804,514 - 1,844,191 a2) | Layer test / isolate *OH.121* | Germplasm (478) | 10.7% ΔRW | - | - | Rolling et al. 2020 |
|  | *qHC12-1* | ss715613311 | (6,694,566 a2) | Hypocotyl inoculation / isolate *PT2004 C2.S1* | Germplasm (460) | - | - | - | Van et al. 2020 |
|  | *-* | - | 61-62cM, | Tray test and layer test / isolates *C.2.S.1, OH25, OH7, 1.S.1.1, OH30* | F7:8 (367) | 4.1% | - | OX20-8 | Lee et al. 2014 |
|  | *-* | - | 62-71cM | Tray test and layer test / isolates *C.2.S.1, OH25, OH7, 1.S.1.1, OH30* | F7:8 (367) | 4.0-4.1% | - | OX20-8 | Lee et al. 2014 |
|  |  | GMH_OSU31 | 37.2-63.4cM | Tray test / isolate *1.S.1.1* | F4:6 (186) | 4.6% | - | - | Wang et al. 2010 |
| MLG F (Chr. 13) | *C2-13-1* | ss715617255 | (13,389,672 - 13,550,863 a2) | Layer test / isolate *C2.S1* | Germplasm (495) | 9.2% ΔSW | - | - | Rolling et al. 2020 |
|  | *C3-13-2* | ss715616837 | (15,952,204 - 16,017,061 a2) | Layer test / isolate *C2.S1* | Germplasm (495) | 0.2% IRRS | - | - | Rolling et al. 2020 |
|  | *qHC13-1* | ss715616768 | (16,421,869 a2) | Hypocotyl inoculation / isolate *PT2004 C2.S1* | Germplasm (460) | - | - | - | Van et al. 2020 |
|  |  | Satt252 (BARCSOYSSR_13_0272, Satt423 (BARCSOYSSR_13_0264), and Satt149 (BARCSOYSSR_13_0245) | (16,454,986 - 16,855,019 a2) | Slant board test / isolate OH25 | F4:6 (66), F4:6 (79), F4:6 (64) | 21.4-35.0% | - | - | Burnham et al. 2003b |
|  | *C2-13-3* | ss715615656 | (18,315,025 - 18,531,998 a2) | Layer test / isolate *C2.S1* | Germplasm (495) | 10.4% ΔRW, 13.1% ΔSW | - | - | Rolling et al. 2020 |
|  | *C2-13-4* | ss715614099 | (19,599,094 - 19,614,217 a2) | Layer test / isolate *C2.S1* | Germplasm (495) | 5.0% IRRS | - | - | Rolling et al. 2020 |
|  |  | F424_294 | 15.7 - 24.1cM | Tray test / isolate *1.S.1.1* | F4:6 (186) | 2.0% | - | - | Wang et al. 2010 |
| MLG B2 (Chr. 14) | *C2-14-1* | ss715618005 | (2,131,853 - 2,153,133 a2) | Layer test / isolate *C2.S1* | Germplasm (495) | 4.7% IRW | - | - | Rolling et al. 2020 |
|  |  | Satt304 (BARCSOYSSR_14_0646) | 22.8 - 31.7cM (13,086,766 a2) | Tray test / isolate *1.S.1.1* | F4:6 (375) | 4.7% | - | - | Wang et al. 2010 |
|  | *Rprr-14-1* | - | (33,243,422 a2) | Hypocotyl inoculation / race *1* | Germplasm and cultivars (225) | - | - | Allele C | Zhao et al. 2020 |
|  | *qHO14-1* | ss715619417 | (47,590,507 a2) | Hypocotyl inoculation / isolate *OH.12108.6.3 (OH.121)* | Germplasm (429) | - | - | - | Van et al. 2020 |
|  | *-* | - | 12-22cM | Tray test and layer test / isolates *C.2.S.1, OH25, OH7, 1.S.1.1, OH30* | F7:8 (367), F7:8 (338) | 2.7% | - | PI 427106 and PI 427105B | Lee et al. 2014 |
| MLG E (Chr. 15) | *-* | BARC-039153-07459 | (831,324 a1) | Hypocotyl inoculation / isolate *Pm28* | China mini core germplasm (224) | 8.6-10.7% | Glyma15g01370 | - | Huang et al. 2016 |
|  | *qHC15-1* | ss715620190 | (1,017,643 a2) | Hypocotyl inoculation / isolate *PT2004 C2.S1* | Germplasm (460) | - | - | - | Van et al. 2020 |
|  |  | BARC_2.0_Gm15_3639988 and BARC_2.0_Gm15_3591774 | (3,591,774 - 3,639,988 a2) | Tray test / isolates *PT2004C2.S1, 1.S.1.1, OH25* | F9:11 (316) | 2.0% | - | Conrad | Stasko et al. 2016 |
|  | *-* | BARC-054257-12408 and BARC-028907-06042 | (4,433,953 - 5,541,487 a2) | Rice-based method / isolates *PT2004C2.S1, 1005-2.9, R7-2a* | F5:7 (232), F5:7 (277) | 7.3% %DRL, 6.7% %DSA | - | PI 399036 | Abeysekara et al. 2016 |
|  | *Rprr-15-1* | - | (6,082,725 a2) | Hypocotyl inoculation / race *1* | Germplasm and cultivars (225) | - | - | Allele A | Zhao et al. 2020 |
|  | *-* | Gm15_18422604 | (18,422,604 a1) | Layer test / isolate ? (vir 1d, 2, 3b, 3c, 4, 5, 6, 7) | Cultivars (169) | 10.6-18.4% | Glyma15g20550 | - | Ludke et al. 2019 |
|  | *-* | Gm15_19326210 | (19,326,210 a1) | Layer test / isolate ? (vir 1d, 2, 3b, 3c, 4, 5, 6, 7) | Cultivars (169) | 11.0% | Glyma15g21130 | - | Ludke et al. 2019 |
|  | *-* | Chr15:36764744 | (35835724 a2) | Hydroponic assay / isolate *Ont-7-1, Ont-42-1, Amand-1* | Germplasm (357) | 13% CDW^l^ | Glyma.15G217100 | Allele T | de Ronne et al. 2021 |
|  | *-* | Q-15-0369188 | (48,863,575 a1) | Hypocotyl inoculation / isolate *HLJ08-17* | China mini core germplasm (224) | 6.3-10.4% | Glyma15g41680 | - | Huang et al. 2016 |
| MLG J (Chr. 16) | *16-1* | BARC_2.0_Gm16_486741 and BARC_2.0_Gm16_807114 | (486,741 - 807,114 a2) | Tray test / isolates *PT2004C2.S1, 1.S.1.1, OH25* | F9:11 (316) | 5.1% | - | Conrad | Stasko et al. 2016 |
|  | *-* | BARC-014467-01559 | (3,962,328 a1) | Hypocotyl inoculation / isolate *HeN08-35* | China mini core germplasm (224) | 6.9-7.5% | Glyma16g04700 | - | Huang et al. 2016 |
|  | *Qsatt414-596* | Satt414 and Satt596 | (8,909,747 -14,155,157 a2) | Field test | F6 (62) | 13.7-21.5% | - | Conrad | Weng et al. 2007 |
|  | *-* | Map-3031 | (15,093,996 a1) | Hypocotyl inoculation / isolate *PNJ1* | China mini core germplasm (224) | 5.0-5.5% | Glyma16g14080 | - | Huang et al. 2016 |
|  | *qHM16-1* | ss715623655 | (20,565,775 a2) | Hypocotyl inoculation / isolates *PT2004 C2.S1, R7-2a, 1005-2.9* | Germplasm (448) | - | - | - | Van et al. 2020 |
|  | *C2-16-1* | ss715623885 | (27,437,538 - 27,660,360 a2) | Layer test / isolate *C2.S1* | Germplasm (495) | 7.5% IRRS | - | - | Rolling et al. 2020 |
|  | *-* | Q-16-0268535 | (33,793,393 a1) | Hypocotyl inoculation / isolate *H15* | China mini core germplasm (224) | 5.0-6.9% | Glyma16g30140 | - | Huang et al. 2016 |
|  | *-* | BARC-042413-08254 | (35,175,092 a1) | Hypocotyl inoculation / isolate *Pmg* | China mini core germplasm (224) | 8.33-9.9% | Glyma16g31930 | - | Huang et al. 2016 |
|  | *-* | Satt414, p50h212, and Satt529 | 6-13cM | Tray test / isolate C2S1 | F10 and F11 (298) | 22-42% | - | PI 407162 | Tucker et al. 2010 |
|  | *-* | - | 77-87cM, 79-87cM | Tray test and layer test / isolates *C.2.S.1, OH25, OH7, 1.S.1.1, OH30* | F7:8 (338) | 2.5-3.6% | - | PI 427105B | Lee et al. 2014 |
| MLG D2 (Chr. 17) | *-* | BARC-058841-15463 and BARC-052295-11407 | (7,487,007 - 8,088,937 a2) | Rice-based method / isolates *PT2004C2.S1, 1005-2.9, R7-2a* | F5:7 (232), F5:7 (277) | 12.7% CDRW | - | PI 399036 | Abeysekara et al. 2016 |
|  | - | Sat_222-168 | (12,887,597 a2) | Slant board assay / race *2* | China mini core collection (175) | 6.7% | - | ZDD01629 | Sun et al. 2014 |
|  | *Rprr-17-1* | - | (14,145,058 a2) | Modified slant board assay / race 2 | Germplasm and cultivars (225) | 25.7% | - | Allele A | Zhao et al. 2020 |
|  | *Rprr-17-2* | - | (14,183,268 a2) | Hypocotyl inoculation / race *1* | Germplasm and cultivars (225) | 30.5% | - | Allele T | Zhao et al. 2020 |
|  | *Rprr-17-3* | - | (14,356,285 a2) | Hypocotyl inoculation / race *1* | Germplasm and cultivars (225) | 25.4% | - | Allele A | Zhao et al. 2020 |
|  |  | BARC-062213-17705 | 94cM (18,409,823 a1) | Hypocotyl inoculation / isolate *OH17*, race *2* | F7:8 (188) | 7.5-8.8% | - | PI 408105A | Nguyen et al. 2012 |
|  |  | Satt574 (BARCSOYSSR_17_1164) | 25.2 - 39.6cM (31,915,278 a1) | Tray test / isolate *1.S.1.1* | F4:6 (375) | 7.1% | - | - | Wang et al. 2010 |
| MLG G (Chr. 18) | *C2-18-1* | ss715630573 | (433,428 a1) | Layer test / isolate *C2.S1* | Germplasm (495) | 2.2% IPH | - | - | Rolling et al. 2020 |
|  | *qHC18-1* | ss715630895 | (44,368,782 a2) | Hypocotyl inoculation / isolate *PT2004 C2.S1* | Germplasm (460) | - | - | - | Van et al. 2020 |
|  | *QTL-18* | BARC-040163-07672 to BARC-041331-07965 | 70-81cM  (54,810,869 - 57,797,198 a1) | Tray test / isolate *OH25* | F7:8 (157) | 3.5% | - | PI 407861A | Lee et al. 2013b |
|  | *OH-18-2* | ss715632090 | (54,737,619 - 54,774,006 a2) | Layer test / isolate *OH.121* | Germplasm (478) | 2.9% IRRS | - | - | Rolling et al. 2020 |
|  | *qHM18-1* | ss715632346 | (56,486,375 a2) | Hypocotyl inoculation / isolates *PT2004 C2.S1, R7-2a, 1005-2.9* | *G. soja* (520) | - | - | - | Van et al. 2020 |
|  | *-* | BARC-031343-07057 | 91cM (58,976,307 a1) | Tray test / isolate *C2S1* | F7:8 (305) | 3.6% | - | PI 398841 | Lee et al. 2013a |
|  | *QDRL-18* | - | 13-16cM | Tray test and layer test / isolates *C.2.S.1, OH25, OH7, 1.S.1.1, OH30* | F7:8 (367), F7:8 (338) | 19.4-24.7% | - | PI 427106 and PI 427105B | Lee et al. 2014 |
|  | *-* | SLP142 | 15-30cM | Tray test / isolate C2S1 | F10 and F11 (298) | 9-11% | - | PI 407162 | Tucker et al. 2010 |
|  | *-* | - | 104-107cM | Tray test and layer test / isolates *C.2.S.1, OH25, OH7, 1.S.1.1, OH30* | F7:8 (338) | 3.1-3.5% | - | PI 427105B | Lee et al. 2014 |
| MLG L (Chr. 19) | *qHO19-1* | ss715634100 | (3,415,217 a2) | Hypocotyl inoculation / isolate OH.12108.6.3 (OH.121) | Germplasm (429) | - | - | - | Van et al. 2020 |
|  | *qHM19-1* | ss715635571 | (46,329,244 a2) | Hypocotyl inoculation / isolates *PT2004 C2.S1, R7-2a, 1005-2.9* | Germplasm (448) | - | - | - | Van et al. 2020 |
|  | *-* | BARC-064609-18739 and BARC-039977-07624 | (47,232,960 - 48,733,355 a2) | Rice-based method / isolates *PT2004C2.S1, 1005-2.9, R7-2a* | F5:7 (232), F5:7 (277) | 8.5% %DRL, 8.9% %DSA | - | PI 399036 | Abeysekara et al. 2016 |
|  | *QTL 19-1* | ss715635897 | (49,121,258 a2) | Tray test / isolates *OH121* and *C2S1* | PI lines (800) | 2.5% Root rot score | - | - | Schneider et al. 2016 |
|  | *QTL 19-2* | ss715635934 | (49,461,582 a2) | Tray test / isolates *OH121* and *C2S1* | PI lines (800) | 2.5% Root rot score | - | - | Schneider et al. 2016 |
|  | *-* | GML_OSU10 | 33.4 - 36.5cM | Tray test / isolate *1.S.1.1* | F4:6 (375) | 7.1% | - | - | Wang et al. 2010 |
| MLG I (Chr. 20) | *OH-20-1* | ss715636836 | (1,724,545 - 1,897,580 a2) | Layer test / isolate *OH.121* | Germplasm (478) | 8.9% IPH | - | - | Rolling et al. 2020 |
|  | *qHC20-1* | ss715637465 | (34,663,053 a2) | Hypocotyl inoculation / isolate *PT2004 C2.S1* | Germplasm (460) | - | - | - | Van et al. 2020 |
|  | *OH-20-2* | ss715638609 | (45,279,755 - 45,458,003 a2) | Layer test / isolate *OH.121* | Germplasm (478) | 7.7% IPH | - | - | Rolling et al. 2020 |
|  | *-* | BARC-013645-01207 | (46,624,541 a1) | Hypocotyl inoculation / isolate *HeN08-35* | China mini core germplasm (224) | 6.8-7.5% | Glyma20g39240 | - | Huang et al. 2016 |
|  | *qHO20-1* | ss715638856 | (47,074,681 a2) | Hypocotyl inoculation / isolate *OH.12108.6.3 (OH.121)* | Germplasm (429) | - | - | - | Van et al. 2020 |
|  | *-* | RGA018 | 12-24cM | Tray test / isolate C2S1 | F10 and F11 (298) | 7-12% | - | V71-370 | Tucker et al. 2010 |

^a^: Marker position (bp) based on the *Glycine max* genome assembly version *Gmax1.01* (a1), or *Gmax2.0* (a2), only starting position is shown for SSR markers.

^b^: Phenotypic variations explained by the molecular markers.

^c^: ΔSW: change in shoot weight

^d^: IRRS: inoculated root rot score

^e^: IRW: inoculated shoot weight

^f^: CDRW: corrected dry root length

^g^: %DRL: percentage of diseased root length

^h^: %DSA: percentage of diseased root surface area

^i^: CDSW: corrected dry shoot length

^j^: ΔRW: change in root weight

^k^: IPH: inoculated plant height

^l^: CDW: corrected dry weight
