# Supplementary material for: Breeding for disease resistance in soybean: a global perspective
Source: Theor Appl Genet. 2022 Jul 5;135(11):3773–872. doi: 10.1007/s00122-022-04101-3 (PMC9729162; doi:10.1007/s00122-022-04101-3)
Supplement: Supplementary file 3 — Supplementary file3 (DOCX 40 kb) [file 122_2022_4101_MOESM3_ESM.docx]

**Supplementary Table 2** Additional soybean loci conferring resistance to sudden death syndrome (SDS) (caused by *Fusarium virgulifomes* and *F*. *tucumaniae*)

| **Causal agent** | **MLG (Chr.)** | **Locus name** | **Tightly linked / flanking markers** | **Marker position**  **cM (bp) ^a^** | **Testing methods / Resistance spectrum** | **Population type (size)** | **PVE ^b^** | **Donor source** | **Reference** |  |
| --- | --- | --- | --- | --- | --- | --- | --- | --- | --- | --- |
| *Fusarium virguliforme* | MLG D1a (Chr. 1) | *qDX002* | ss244562583-ss244554797 | 15.40–16.10cM | Field test (IL, US) | F5:7 (94) | 0.5% DX | - | Anderson et al. 2015 |  |
|  |  | *SDS14-1, qDX001* | ss244562583-ss244554797 | 15.60–15.90cM | Field test (IL, US) | F5:7 (94) | 0.9% DX | - | Anderson et al. 2015 |  |
|  |  | *SDS20-2* | BARC-058851-15477 | (7,443,056 a2) | greenhouse and field | F2 derived (106) | - | PI 507531 | Brzostowski et al. 2018 |  |
|  |  | - | ss715579740_A_G | (48,684,616 a2) | growth chamber / Isolates Mont-1, Scott F2I11a and Clinton 1B | PI lines (254) | 6.0% | - | Swaminathan et al. 2019 |  |
|  | MLG D1b (Chr. 2) | - | Gm02-707483 | (707,483 a1) | Field test (MI, US) | Advanced breeding lines (300) | 5.6% DX | - | Wen et al. 2014 |  |
|  |  | - | ss715582444_T_G | (39,634,936 a2) | Greenhouse test | GWA Panel (214) | 6% DAI20, 7% DAI23 | - | Zhang et al. 2015 |  |
|  |  | - | ss715584189_T_C | (9,450,450 a2) | Greenhouse test | GWA Panel (214) | 6% DAI20, 7% DAI23, 7% AUDPC | - | Zhang et al. 2015 |  |
|  | MLG N (Chr. 3) | *SDS14-1* | BARC-044643-08744 | (460,192 a2) | Somerset #1A | - | - | - | Bao et al. 2015 |  |
|  |  | *-* | ss715585711_A_C | (3,850,374 a2) | Isolates Mont-1, Scott F2I11a and Clinton 1B/ growth chamber | PI lines (254) | 7.52% | - | Swaminathan et al. 2019 |  |
|  | MLG C1 (Chr. 4) | - | Satt578 | (7,891,823 a2) | Field test (IL, US) | - | 14% | Spencer | de Farias Neto et al. 2007 |  |
|  |  | *SDS disease index 21-4* | Gm04_7957588_G_T to Gm04_38135736_T_G | (8,029,471 – 41,298,992 a2) | Isolate LL0009 (NE305)/ greenhouse | F2:3 (200) | 9.0% DX | MN1606SP | Luckew et al. 2017 |  |
|  | MLG A1 (Chr. 5) | - | ss715591951_C_T | (40,431,439 a2) | Isolates Mont-1, Scott F2I11a and Clinton 1B/ growth chamber | PI lines (254) | 6.3% | - | Swaminathan et al. 2019 |  |
|  | MLG C2 (Chr. 6) | *Fusarium root rot 1-3* | Gm06_1006528_G_A to Gm06_2325487_G_T | (1,018,033 -2,352,768 a2) | Isolate LL0009 (NE305)/ greenhouse | F2:3 (200) | 15.6% Root rot severity | MN1606SP | Luckew et al. 2017 |  |
|  |  | - | BARC-Satt316 | (48,016,485 a2) | Field test (IL, US) |  | 9.1% DI | Pyramid | Njiti et al. 2002 |  |
|  |  | *qSDS6-1* | ss245826901–ss245842048 | - | Field test (MI, US) | F4 derived (153) | 7.0% | E07080 | Tan et al. 2019 |  |
|  |  | *qSDS6-2* | ss245879277–ss245888974 | - | Field test (MI, US) | F4 derived (153) | 6.1-17.4% | E07080 | Tan et al. 2019 |  |
|  |  | - | ss245842048 | - | Field test (MI, US) | Advanced breeding lines (300) | 7.7% DX | - | Wen et al. 2014 |  |
|  | MLG M (Chr. 7) | *SDS16-2* | BARC-028517-05936 to BARC-065255-19294 | 103.98–133.83cM (39,211,962-43,709,400 a1) | Isolates Clinton 1B, Scott F2II 1a and Scott B2 / growth chamber | F7 derived RIL (200) | 12.2% Stem cut | LS98-0582 | Swaminathan et al. 2016 |  |
|  |  | - | Gm07-15654480 | (15,654,480 a1) | Field test (MI, US) | Advanced breeding lines (300) | 5.5% DX | - | Wen et al. 2014 |  |
|  |  | - | Gm07-36959086 | (36,959,086 a1) | Field test (MI, US) | Advanced breeding lines (300) | 6.5% DS | - | Wen et al. 2014 |  |
|  |  | *qSDS7-1* | ss246280747–ss246282071 | - | Field test (MI, US) | F4 derived (153) | 9.9% | E07080 | Tan et al. 2019 |  |
|  | MLG A2 (Chr. 8) | *SDS13-7, qFDS003-04* | ss107919498–ss107915722 | 2.0–13.0cM | Greenhouse test | F6:13 (50) | 9.6% FDS | PI 438489B | Abdelmajid et al. 2012 |  |
|  |  | *SDS5-2, Rfs1* | BLT65 | - | Field test | - | 0.6% R6, 1.4% R8 IS | Hartwig | Prabhu et al. 1999 |  |
|  |  | - | ss246580442 | - | Field test (MI, US) | Advanced breeding lines (300) | 10.9% DX | - | Wen et al. 2014 |  |
|  |  | - | ss246585278 | - | Field test (MI, US) | Advanced breeding lines (300) | 8.1% DX | - | Wen et al. 2014 |  |
|  |  | - | ss715599474_G_A | (1,373,179 a2) | Greenhouse test | Germplasm (214) | 7% DAI20 | - | Zhang et al. 2015 |  |
|  | MLG K (Chr. 9) | - | ss715603096 | - | Field test (IL, US) | - | - | - | Chang et al. 2016 |  |
|  |  | - | Gm09-43648118 | (43,648,118 a1) | Field test (MI, US) | Advanced breeding lines (300) | 11.6% DI | - | Wen et al. 2014 |  |
|  | MLG O (Chr. 10) | *SDS13-2, qFDS002-02* | ss107930838–ss107912519 | 13.5-15.2cM | Greenhouse test | F6:13 (50) | 19.3% FDS | PI 438489B | Abdelmajid et al. 2012 |  |
|  |  | *SDS14-8, qDX010* | ss247085505-ss247098566 | 1.30–3.30cM | Field test (IL, US) | F5:7 (94) | 0.04 % DX | - | Anderson et al. 2015 |  |
|  |  | *SDS19-1* | Satt262 | (35,511,082 a2) | greenhouse and field | F5 derived (91) | 18.7% DX | Ripley | Brzostowski et al. 2018 |  |
|  |  | *SDS16-7* | BARC-017045-02182 to BARC-060901-16948 | 51.00–53.66cM (6,431,374-10,118,500 a2) | Isolates Clinton 1B, Scott F2II 1a and Scott B2 / growth chamber | F7 derived RIL (200) | 5% Root feeding | A95-684043 | Swaminathan et al. 2016 |  |
|  |  | - | ss715608333_A_G | (5,775,923 a2) | Isolates Mont-1, Scott F2I11a and Clinton 1B/ growth chamber | PI lines (254) | 9.1% | - | Swaminathan et al. 2019 |  |
|  |  | - | ss715608329_C_T | (5,749,166 a2) | Isolates Mont-1, Scott F2I11a and Clinton 1B/ growth chamber | PI lines (254) | 8.7% | - | Swaminathan et al. 2019 |  |
|  |  | - | ss715608298_G_A | (5,537,936 a2) | Isolates Mont-1, Scott F2I11a and Clinton 1B/ growth chamber | PI lines (254) | 6.5% | - | Swaminathan et al. 2019 |  |
|  | MLG B1 (Chr. 11) | *SDS13-8, qFDS004-01* | ss107912672–ss107924081 | 5.5–17.8cM | Greenhouse test | F6:13 (50) | 3.4% FDS | PI 438489B | Abdelmajid et al. 2012 |  |
|  |  | - | Satt583–Satt415 | (27,790,963 a2) | Field test (IL, US) | - | 37% MNDI |  | Yuan et al. 2012 |  |
|  |  | - | ss715609076 | - | Field test (IL, US) | - | - | - | Chang et al. 2016 |  |
|  |  | - | Gm11-37426559 | (32,978,048 a2) | Field test (MI, US) | Advanced breeding lines (300) | 5.6% DI | - | Wen et al. 2014 |  |
|  | MLG H (Chr. 12) | *Rfs16* | Satt353 | (1,682,640 a2) | Isolate Clinton1b and Scott, greenhouse test | - | - | - | Luckew et al. 2013 |  |
|  |  | *SDS16-3* | BARC-042199-08209- BARC-044217-08646 | 14.99–67.86cM | Isolates Clinton 1B, Scott F2II 1a and Scott B2 / growth chamber | F7 derived RIL/ 200 | 10.2% Root feeding | LS98-0582 | Swaminathan et al. 2016 |  |
|  |  | - | ss715612543_C_T | (35,078,375 a2) | Isolates Mont-1, Scott F2I11a and Clinton 1B/ growth chamber | PI lines (254) | 7.1% | - | Swaminathan et al. 2019 |  |
|  |  | *SDS disease incidence 20-3, qSDS-12* | ss247654378-ss247700264 | 54.47–73.99cM (7.4–12.7Mb, a1) | Field test (MI, US) | F4 derived (129) | 4.5% DI | GD2422 | Tan et al. 2018 |  |
|  | MLG F (Chr. 13) | *SDS14-9, qDX011* | ss247942156-ss247937719 | 2.50–2.80cM | Field test (IL, US) | F5:7 (94) | 0.1% DX | - | Anderson et al. 2015 |  |
|  |  | - | ss715613738_G_T | (11,106,359 a2) | Isolates Mont-1, Scott F2I11a and Clinton 1B/ growth chamber | PI lines (254) | 8.1% | - | Swaminathan et al. 2019 |  |
|  |  | - | ss715617107_A_G | (14,610,894 a2) | Isolates Mont-1, Scott F2I11a and Clinton 1B/ growth chamber | PI lines (254) | 6.1% | - | Swaminathan et al. 2019 |  |
|  |  | - | ss715617111_A_C | (14,577,952 a2) | Isolates Mont-1, Scott F2I11a and Clinton 1B/ growth chamber | PI lines (254) | 6.3% | - | Swaminathan et al. 2019 |  |
|  |  | - | ss715617189_C_T | (14,040,386 a2) | Isolates Mont-1, Scott F2I11a and Clinton 1B/ growth chamber | PI lines (254) | 7.0% | - | Swaminathan et al. 2019 |  |
|  |  | - | ss715617218_C_T | (13,855,912 a2) | Isolates Mont-1, Scott F2I11a and Clinton 1B/ growth chamber | PI lines (254) | 5.1% | - | Swaminathan et al. 2019 |  |
|  |  | - | ss715615487_C_T | (18,567,932 a2) | Isolates Mont-1, Scott F2I11a and Clinton 1B/ growth chamber | PI lines (254) | 8.0% | - | Swaminathan et al. 2019 |  |
|  |  | *qPY13-1* | ss248065435–ss248078524 | (29.38–30.10 Mb a2) | Field test (MI, US) | F4 derived (153) | 6.3% plot yield | U01-390489 | Tan et al. 2019 |  |
|  | MLG B2 (Chr. 14) | *Fusarium root rot 1-2* | Gm14_30024382_T_C to Gm14_43417417_T_C | (34,212,172 – 42,687,702 a2) | Isolate LL0009 (NE305)/ greenhouse | F2:3 (200) | 26.8% Root rot severity | MN1606SP | Luckew et al. 2017 |  |
|  |  | - | ss715618125_A_G | (2,597,934 a1) | Isolates Mont-1, Scott F2I11a and Clinton 1B/ growth chamber | PI lines (254) | 6.9% | - | Swaminathan et al. 2019 |  |
|  |  | - | ss715619446_ C_T | (48,509,947 a2) | Isolates Mont-1, Scott F2I11a and Clinton 1B/ growth chamber | PI lines (254) | 6.1% | - | Swaminathan et al. 2019 |  |
|  |  | - | ss715619290_C_T | (47,361,927 a2) | Isolates Mont-1, Scott F2I11a and Clinton 1B/ growth chamber | PI lines (254) | 5.5% | - | Swaminathan et al. 2019 |  |
|  |  | - | Gm14-4636247 | (4,720,511 a2) | Field test | Advanced breeding lines (300) | 5.3% DI | - | Wen et al. 2014 |  |
|  | MLG E (Chr. 15) | - | ss715622696_G_T | (50,090,190 a2) | Isolates Mont-1, Scott F2I11a and Clinton 1B/ growth chamber | PI lines (254) | 5.6% | - | Swaminathan et al. 2019 |  |
|  |  | - | ss248566590 | (5,978,279 a1) | Field test (MI, US) | Advanced breeding lines (300) | 5.8% DI | - | Wen et al. 2014 |  |
|  | MLG J (Chr. 16) | *SDS disease incidence 21-3* | Gm16_31454423_G_A to Gm16_37319334_C_A | (31,822,897 – 37,811,857 a2) | Isolate LL0009 (NE305)/ greenhouse | F2:3 (200) | 11.8% DI | MN1606SP | Luckew et al. 2017 |  |
|  |  | - | Satt183, Satt456, Sct065, Sct001 | (25,103,222 a2) | Greenhouse test | - | - | GC87012-12-2B-1 | Sanitchon et al. 2004 |  |
|  |  | - | Satt285 | (2,802,624 a2) | Field test (IL, US) | - | 10.2% DS | Essex | Abdelmajid et al. 2007 |  |
|  | MLG D2 (Chr. 17) | *-* | BARC-064101-18557 | (25,852,278 a2) | Somerset #1A | - | - | - | Bao et al. 2015 |  |
|  |  | - | BARC-023721-03465 | (20,352,435 a2) | Somerset #1A | - | - | - | Bao et al. 2015 |  |
|  |  | *cqSDS-001* | Satt226 | (26,769,188 a2) | Field test (IL, US) | - | 14% | Ripley | de Farias Neto et al. 2007 |  |
|  |  | *SDS17-3, SDS-6* | BARC-020357-04569 – BARC-065705-19668; Sctt008–Sct_192 | (789,923-3,369,886 a2) | Isolates Clinton 1B, Scott F2II 1a / growth chamber | F7:8 (200) | 7.5% | LS98-0582 | Swaminathan et al. 2018 |  |
|  |  | *qSDS17-1* | ss249273026–ss249290628 | (4.65–6.82 Mb a2) | Field test (MI, US) | F4 derived (153) | 6.5% | E07080 | Tan et al. 2019 |  |
|  |  | - | ss715627896_C_A | (4,355,885 a2) | Greenhouse test | Germplasm (214) | 6% DAI20, 6% AUDPC | - | Zhang et al. 2015 |  |
|  | MLG G (Chr. 18) | *SDS20-1* | BARC-0475-12985 | - | greenhouse and field | F2 derived (106) | - | PI 507531 | Brzostowski et al. 2018 |  |
|  |  | *ds4* | OIO3-ACC230 (95.1cM-103.71cM) | - | Field test (IL, US) | - | 11.9% | Essex | Kassem et al. 2006 |  |
|  |  | *SDS11-5, cqRfs3* | Satt115, Satt427 | (10,458,453 a2) | Field test (IL, US) | - | 6.4-15% IS | Hartwig | Kazi et al. 2008 |  |
|  |  | - | ss715631294_C_T | (47,670,147 a2) | Isolates Mont-1, Scott F2I11a and Clinton 1B/ growth chamber | PI lines (254) | 6.5% | - | Swaminathan et al. 2019 |  |
|  |  | *SDS disease incidence 20-4, SDS disease index 20-2, SDS disease severity 20-1, qSDS-18* | ss249498763-ss249516114 | 0–11.62cM | Field test (MI, US) | F4 derived (129) | 22.1-35.1% DI, DS, DX | LD01-5907 | Tan et al. 2018 |  |
|  |  | *qRfs1* | OI03-P4 and CTA13-SCAR | - | Field test | - | - | Forrest | Triwitayakorn et al. 2005 |  |
|  |  | - | ss249520656 | (2,444,089 a2) | Field test (MI, US) | Advanced breeding lines (300) | 9.5% DI | - | Wen et al. 2014 |  |
|  |  | - | ss715631747_C_T | (51,968,926 a2) | Greenhouse test | Germplasm (214) | 8% DAI23, 7% DAI26, 8% AUDPC | - | Zhang et al. 2015 |  |
|  | MLG L (Chr. 19) | *SDS9-2* | Satt006 | (50,057,960 a2) | Strain ST-90, greenhouse | F7:14 (284) | 5% DS | Minsoy | Njiti and Lightfoot 2006 |  |
|  |  | - | ss715634180_G_A | (3,458,700 a2) | Greenhouse test | Germplasm (214) | 6% DAI20, 7% DAI23, 7% AUDPC | - | Zhang et al. 2015 |  |
|  |  | - | ss715634431_C_T | (35,932,013 a2) | Greenhouse test | Germplasm (214) | 8% DAI26, 6% AUDPC | - | Zhang et al. 2015 |  |
|  |  | - | ss715634448_A_G | (36,018,563 a2) | Greenhouse test | Germplasm (214) | 7% DAI26 | - | Zhang et al. 2015 |  |
|  |  | - | Gm19-34890716 | (34,890,716 a1) | Field test (MI, US) | Advanced breeding lines (300) | 5.8% DI | - | Wen et al. 2014 |  |
|  |  | - | ss715633619_C_T | (2,871,338 a2) | Greenhouse test | Germplasm (214) | 7% DAI29 | - | Zhang et al. 2015 |  |
|  | MLG I (Chr. 20) | - | ss715637220_A_G | (30,662,956 a2) | Greenhouse test | Germplasm (214) | 7% DAI20 | - | Zhang et al. 2015 |  |
|  |  | *SDS15-6* | BARC-029803-06418 to BARC-041445-07985 | 55.09–65.62cM (36,856,000 - 38,121,706 a2) | Isolates Clinton 1B, Scott F2II 1a and Scott B2 / growth chamber | F7 derived RIL (200) | 6.2% Stem cut | LS94-3207 | Swaminathan et al. 2016 |  |
|  | Unlinked | *SDS8-4* | OG01 | - | Field test (IL, US) | - | 17.2% DI | Pyramid | Njiti et al. 2002 |  |
|  | | | | | | | | | | |
| *F. tucumaniae*  (South America) | MLG D1a (Chr. 1) | *SDS12-1, SDS13-1, qFDS002-01* | ss107927723–ss107913849 | 27.2-42.8cM | Greenhouse test | F6:13 (50) | 7.5% FDS (foliar disease severity) | PI 438489B | Yamanaka et al. 2006 |  |
|  | MLG D1b (Chr. 2) | *SDS13-4, qFDS003-01* | ss107927695–ss107913858 | 19.4–21.8cM | Greenhouse test | F6:13 (50) | 9.0% FDS | PI 438489B | Yamanaka et al. 2007 |  |
|  | MLG C1 (Chr. 4) | *SDS13-11, qRRS001-02* | ss107929213–ss107929551 | 51.5–57.3cM | Greenhouse test | F6:13 (50) | 8.6% RRS | PI 438489B | Yamanaka et al. 2008 |  |
|  | MLG A1 (Chr. 5) | *RSDS1* | Satt599 | (38,647,191 a2) | *F. tucumaniae* sp. nov. MJ161 | - | 18.4% DX | Misuzudaizu | Yamanaka et al. 2006 |  |
|  | MLG K (Chr. 9) | *RSDS2* | GM055b | - | *F. tucumaniae* sp. nov. MJ161 | - | 8.7% DX | Moshidou Gong 503 | Yamanaka et al. 2006 |  |
|  | MLG I (Chr. 20) | *RSDS4* | GM222b | - | *F. tucumaniae* MJ161 | - | 6.6% DX | Moshidou Gong 503 | Yamanaka et al. 2006 |  |

^a^: Marker position (bp) based on the *Glycine max* genome assembly version *Gmax1.01* (a1), or *Gmax2.0* (a2), only starting position is shown for SSR markers.

^b^: Phenotypic variations explained by the molecular markers.
